# Spatial Assembly of Mechanically Planar Chiral Rotaxanes on Rationally Designed Cavitands

**DOI:** 10.1002/anie.202515927

**Published:** 2025-10-26

**Authors:** Liwen Xia, Wenyang Hong, Chong Tian, Ye Zhu

**Affiliations:** ^1^ Department of Chemistry Faculty of Science National University of Singapore 3 Science Drive 2 Singapore 117543 Singapore; ^2^ Department of Pharmacy and Pharmaceutical Sciences Faculty of Science National University of Singapore 18 Science Drive 4 Singapore 117559 Singapore

**Keywords:** Cavitands, Inherent chirality, Mechanical planar chirality, Rotaxanes, Stereocontrol

## Abstract

Chirality plays a central role in chemistry, biology, and pharmaceutical sciences. Although covalent chirality is widely studied, stereoselective assembly of mechanically interlocked molecules (MIMs) remains challenging. Herein, we report a spatial assembly strategy for the stereoselective synthesis of mechanically planar chiral (MPC) rotaxanes. Rationally designed inherently chiral cavitands orchestrate the spatial arrangement of macrocycle and axle components during mechanical interlocking. This approach enables high levels of stereocontrol in both active‐template and passive‐template synthesis of MPC rotaxanes (up to 93:7 e.r.). Our study demonstrates that stereoinduction of MIMs can be made predictable through rational design of chiral auxiliaries, paving the way for applications of the spatial assembly strategy in asymmetric supramolecular synthesis.

## Graphical abstract

A spatial molecular assembly strategy enables the synthesis of enantioenriched mechanically planar chiral rotaxanes. Inherently chiral cavitands are rationally designed to encode and transmit the stereochemical information. Predictable stereochemical induction is achieved by controlling the spatial arrangement of molecular components through engineering combinations of covalent bonding, noncovalent interactions, and reaction activation.

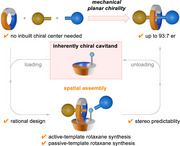

## Introduction

**Scheme 1:**
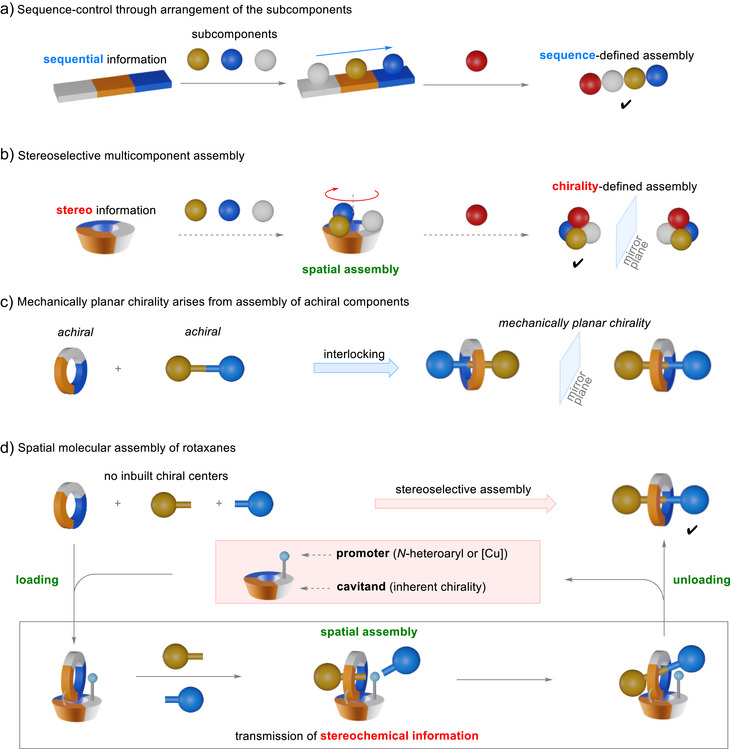
Sequence‐selective templated synthesis and proposed inherently chiral cavitands for stereoselective assembly of mechanically planar chiral rotaxanes.

Encoding and decoding structural information are foundational to life, exemplified by the translation of genetic information from DNA into peptides. Inspired by the natural processes, researchers have engineered templates^[^ ^1^, ^2^, ^3^, ^4^ ^]^ to direct the assembly of sequence‐specific^[^ ^5^, ^6^, ^7^, ^8^, ^9^ ^]^ and length‐specific polymers,^[^ ^10^, ^11^ ^]^ and size‐specific macrocycles^[^ ^12^, ^13^, ^14^ ^]^ and cages^[^ ^15^ ^]^ (Scheme 1a). In contrast, stereoselective multicomponent assembly on chiral templates remains elusive^[^ ^16^ ^]^ (Scheme 1b). This strategy could offer stereochemical predictability without relying on inbuilt stereogenic elements near the reaction sites or chiral confinement within space‐defined cages^[^ ^17^ ^]^ and capsules.^[^ ^18^ ^]^ However, its development has been hindered by the lack of enantioenriched, hetero‐functionalized concave scaffolds.

To explore this strategy, we selected mechanically planar chirality as a rigorous test. This intriguing stereogenicity of mechanically interlocked molecules (MIMs) can manifest even when neither component is chiral.^[^ ^19^, ^20^ ^]^ Specifically, mechanical interlocking of a directional macrocycle and an oriented axle affords enantiomeric mechanically planar chiral (MPC) rotaxanes (Scheme 1c). Despite their expanding applications in sensing,^[^ ^21^, ^22^ ^]^ molecular machines,^[^ ^23^ ^]^ catalysis,^[^ ^24^ ^]^ and synthesis,^[^ ^25^, ^26^ ^]^ MPC rotaxanes are typically used in racemic forms, such as in rotaxane‐crosslinked polymers, where stereoregularity becomes important.^[^ ^27^, ^28^, ^29^ ^]^ To date, effective stereocontrol over assembly of MPC rotaxanes is rare. Hirose and coworkers reported chirality transmission from prerotaxanes that relies on chiral chromatographic separation.^[^ ^30^ ^]^ Goldup and coworkers have achieved high stereoselectivity for active‐template assembly^[^ ^31^ ^]^ directed by inbuilt stereocenters.^[^ ^32^, ^33^, ^34^ ^]^ Leigh and coworkers developed an enantioselective strategy using a chiral leaving group.^[^ ^35^ ^]^ The intrinsic flexibility of mechanical bonds and the challenges in elucidating modes of stereoinduction continue to impede the rational design of asymmetric assembly strategy.^[^ ^36^, ^37^, ^38^ ^]^

Herein, we report the rational design and development of inherently chiral cavitands for spatial assembly of MPC rotaxanes (Scheme 1d). The cavitands position the macrocycle and two half‐axles through covalent bonds, attractive noncovalent interactions, and metal coordination, and the on‐rim catalytic promoters facilitate axle formation. The stereogenicity arising from the curvature of the cavitands defines the spatial arrangement of molecular components, transmitting the stereochemical information to the assembled rotaxanes in a predictable manner. Furthermore, the spatial assembly is applicable to both active‐template and passive‐template rotaxane syntheses via organocatalytic and metal‐mediated processes.

## Results and Discussion

### Design of Cavitands for Spatial Assembly

**Scheme 2:**
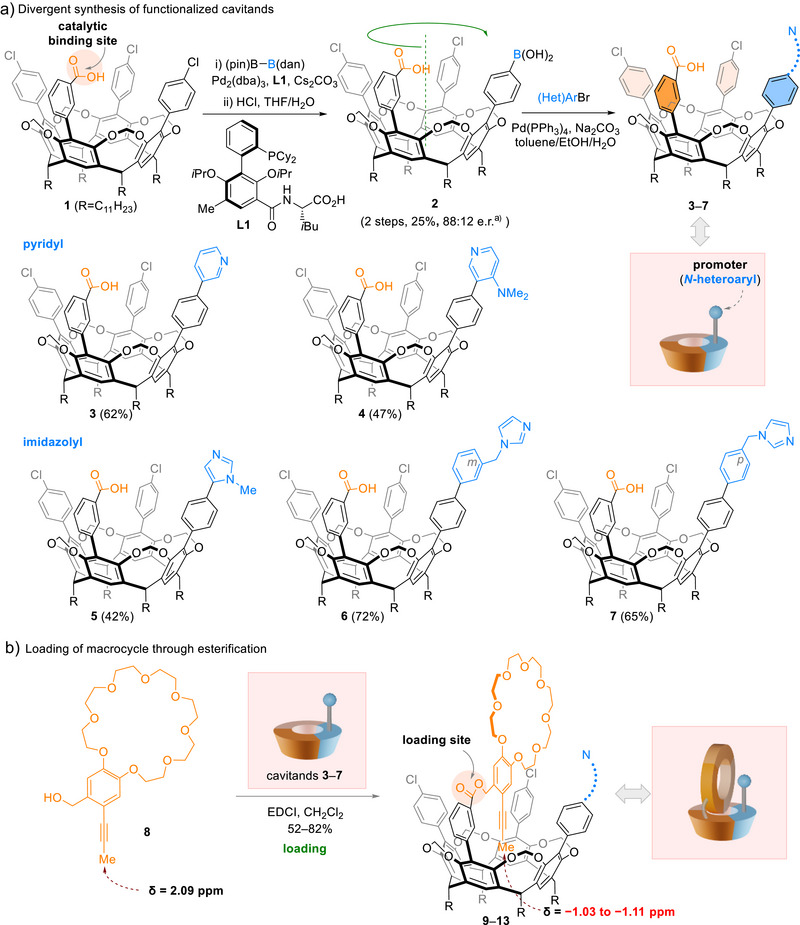
Synthesis of cavitands and loading of macrocycle. ^a)^Enantiomeric ratio (e.r.) of **2** was determined by analyzing the Suzuki coupling product with *p*‐MeOC_6_H_4_B(OH)_2_ (**S1** in Supporting Information) using chiral stationary phase HPLC. dan: naphthalene‐1,8‐diaminato; pin: pinacol; EDCI: 1‐ethyl‐3‐(3‐dimethylaminopropyl)carbodiimide.

We began our study by designing functionalized inherently chiral resorcinarene cavitands. We envisioned that N‐heterocycles incorporated on the upper rims of cavitands, such as pyridyl and imidazolyl groups, could serve as nucleophilic promoters and ligands for metal catalysts (Scheme 2a). To achieve a divergent synthesis, we postulated that a stereoselective Miyaura borylation reaction of prochiral cavitand **1** could give access to a common enantioenriched boronate intermediate for further derivatization.

Using (pin)B–B(dan) as the coupling partner, we achieved a site‐selective and stereoselective borylation reaction by repurposing a chiral catalyst (Pd–**L1**) previously developed for desymmetrizing Suzuki coupling.^[^ ^39^, ^40^ ^]^ Under basic reaction conditions, the carboxylate anion of **1** serves as a catalyst‐binding group that steers the Pd catalyst, thereby directing the borylation. Following acidic deprotection of the B(dan), boronic acid‐functionalized cavitand **2** was obtained in 88:12 e.r. Subsequently, five pyridyl‐derived and imidazolyl‐derived aryl halides were coupled with **2**, affording functionalized cavitands (**3**–**7**) in 42%–72% overall yields upon saponification.

**Scheme 3:**
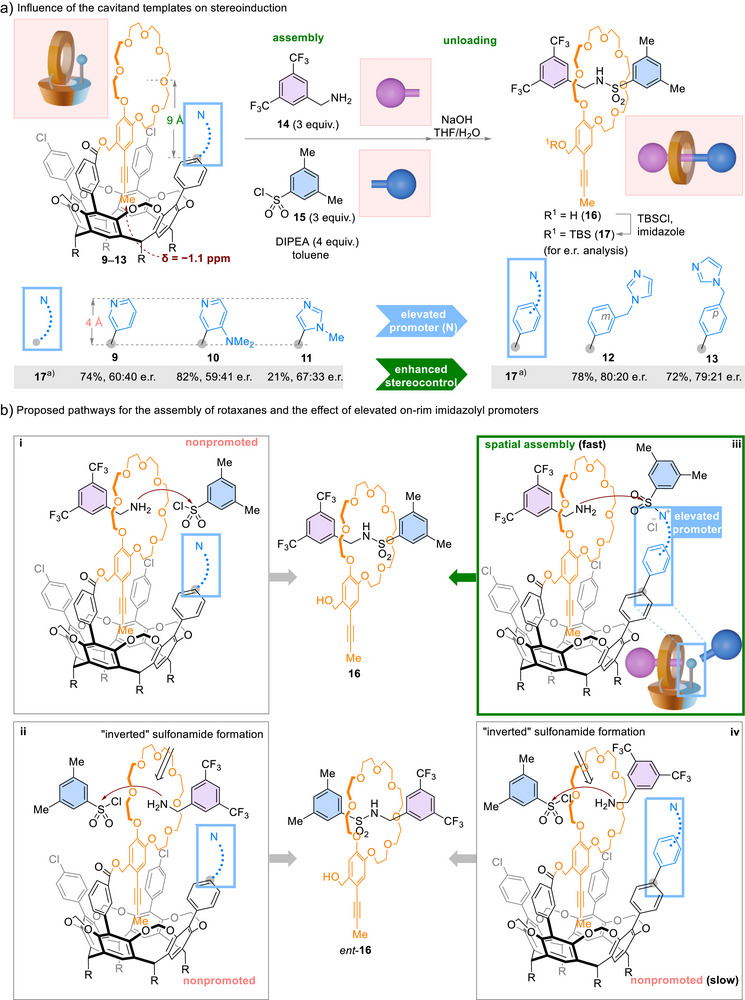
Spatial assembly of MPC rotaxane. ^a)^Overall yield for three steps starting from **9** to **13**. DIPEA: *N, N*‐diisopropylethylamine; TBS: *tert*‐butyldimethylsilyl.

With the chiral cavitands in hand, we evaluated the loading of macrocycle (Scheme 2b). The carboxyl groups of cavitands **3**–**7**, which serve as catalyst‐binding groups in the borylation step, now act as the anchors for loading of the macrocycle. Crown ether **8** was readily loaded onto the rim of the cavitands via ester linkages (Scheme 3a, **9**–**13**). The terminal methyl group of **8** is encapsulated within the cavitand cavity, as evidenced by a pronounced upfield shift observed in ^1^H NMR spectra (in CDCl_3_, CH_3_C≡C δ = −1.1 ppm, ∆δ = −3.1 ppm compared with **8**). In this orientation, the N‐heterocycle moiety of the cavitand is positioned towards a specific side of the crown ether, setting the stage for rotaxane assembly.

### Spatial Assembly of MPC Rotaxanes

We first investigated the viability of spatial assembly using pyridyl‐derived cavitand **9** through an active‐template rotaxane formation pioneered by Leigh and coworkers.^[^ ^41^, ^42^, ^43^, ^44^ ^]^ In the initial experiment, benzylamine **14** and sulfonyl chloride **15** were mixed with **9** in a 3:3:1 stoichiometry in toluene with *N,N*‐diisopropylethylamine as an acid scavenger, leading to rotaxane assembly. Subsequent saponification unloaded rotaxane **16** from the cavitand, and TBS protection afforded the rotaxane product **17** in 74% overall yield with 60:40 e.r. Gratifyingly, chirality was transmitted from the cavitand to the assembled rotaxane to a small yet measurable extent (Scheme 3a).

Encouraged by the initial result using pyridyl‐derived cavitand **9**, we evaluated the cavitands containing on‐rim dimethylaminopyridine (DMAP) and N‐methylimidazole (NMI) groups (**10** and **11**). We hypothesized that the high nucleophilic catalytic activity of DMAP and NMI might further accelerate sulfonamide formation and enhance the effectiveness of spatial assembly. However, when DMAP‐derived cavitand **10** was utilized, the enantiomeric ratio of the rotaxane product **17** (59:41 e.r.) remained similar to the result starting from **9**. When NMI‐derived **11** was employed, **17** was isolated in lower yield but with slightly improved enantiopurity (21% yield, 67:33 e.r.).

We postulated that an appropriate geometrical arrangement of the *N*‐heterocycle and the crown ether could facilitate the proposed spatial assembly. If the crown ether moiety is oriented upwards, its center is estimated to be approximately 9 Å above the tetraphenyl groups of the cavitand scaffold. The *N*‐heterocycle substituents, which are less than 4 Å in diameter, are insufficient to align the sulfonyl moiety with the center of the crown ether. This misalignment between the nucleophilic amino group and the electrophilic sulfonyl imidazolium hinders the proposed spatial assembly. The nonpromoted reaction pathways between the crown ether moiety, amino group of **14**, and sulfonyl chloride **15** without the involvement of *N*‐heterocycle contribute to the nonselective formation of both enantiomers of the rotaxane products (**16** and *ent*‐**16**, Scheme 3b, i and ii). In addition, the sulfonyl imidazolium could react with **14** outside of the crown ether to form the free axle molecule, which is nonproductive.

We hypothesized that incorporating an extended linker between the pendant *N*‐heterocycle and the cavitand scaffold would project the sulfonyl group closer to the center of the crown ether, thereby favoring the spatial assembly (Scheme 3b, iii) over the nonpromoted pathway (iv). Therefore, we investigated cavitands **6** and **7**, in which the imidazolyl groups are connected via phenylene methylene linkages at meta and para positions, respectively (Scheme 2). Despite the conformational flexibility of the *m*‐ or *p*‐phenylene methylene linking the imidazolyl, the resulting rotaxane products obtained from both cavitands exhibited noticeably improved enantiopurity (80:20 e.r. from **12** and 79:21 e.r. from **13**) as well as satisfactory yields (Scheme 3a). The similar levels of stereocontrol observed with **12** and **13** indicate that spatial assembly tolerates conformational changes to a certain degree, and the cavitands transmit stereochemical information through spatial alignment of subcomponents rather than steric confinement.

**Scheme 4:**
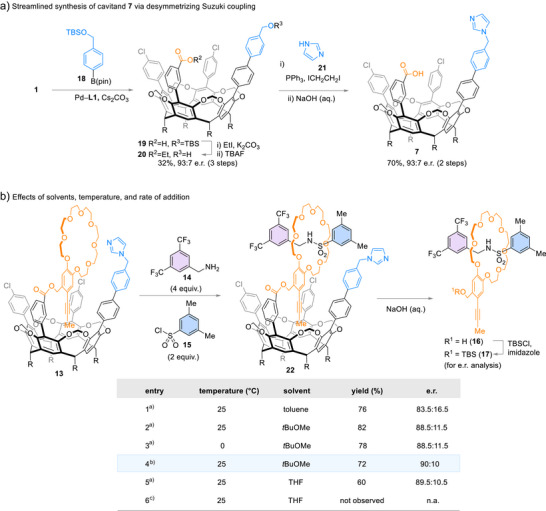
Optimization of conditions. ^a)^Aryl sulfonyl chloride was added in one portion. ^b)^A solution of **15** in *t*BuOMe was slowly added via a syringe over 4 h. 82% of cavitand **7** was recovered upon saponification of **22**. ^c)^Direct formation of rotaxane **17** was not observed in THF starting from **14**, **15**, and OTBS‐protected **8** without loading on the cavitand.

To access cavitand **7** for further studies, we simplified the synthesis via desymmetrizing Suzuki coupling (Scheme 4a, 93:7 e.r.). Following the loading–assembly–unloading sequence, rotaxane **16** was obtained with improved enantiopurity (Scheme 4b, entry 1, 83.5:16.5 e.r.). In the assembly stage, **14** and **15** were mixed with **13** in a 4:2:1 stoichiometry without additional base. Changing the solvent from toluene to *t*BuOMe further enhanced yield and stereoselectivity (entry 2, 82%, 88.5:11.5 e.r.). No change in stereocontrol was observed when the reaction was performed at 0 °C (entry 3). Slow addition of sulfonyl chloride **15** as a solution in *t*BuOMe via syringe pump to a mixture of **13** and **14** in *t*BuOMe led to slightly improved e.r. (entry 4, 90:10 e.r.). Presumably, the slow addition of **15** reduces the concentration of free electrophile in excess relative to the imidazole moiety of **13**, thereby suppressing the nonpromoted reactions (Scheme 3b, iv). Furthermore, cavitand **7** was recovered (82%) through chromatography after saponification of **22**.

The active‐template rotaxane assembly relies on pre‐complexation between crown ether and benzylamine through hydrogen bonds.^[^ ^33^, ^45^ ^]^ The detrimental effect of polar solvents is evident: the formation of rotaxanes was not observed by reacting **14**, **15**, and TBS‐protected **8** (entry 6) or benzo‐24‐crown‐8 in THF. In contrast, when the macrocycle was loaded onto the cavitand (**13**, 93:7 e.r.), rotaxane was formed even when the reaction was carried out in THF, and transmission of chirality remained effective (entry 5, 60% yield, 89.5:10.5 e.r.). It is possible that the preorganization of crown ether and electrophilic sulfonyl group by the cavitand renders the rotaxane assembly less susceptible to disruption of the hydrogen bonds between amino group of **14** and the crown ether moiety. The robustness of spatial assembly against disruption of intercomponent interactions is advantageous, because competing nonpromoted pathways (Scheme 3b) might be suppressed.

### Active‐Template MPC Rotaxane Assembly

**Scheme 5:**
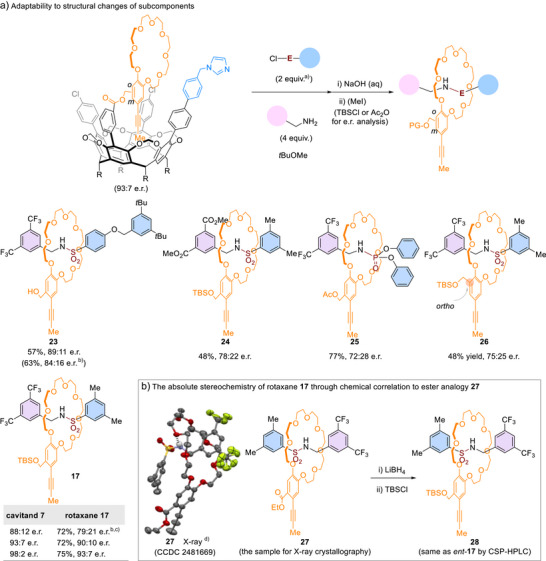
Active‐template MPC rotaxane assembly. ^a)^A solution of aryl sulfonyl chloride or diphenyl chlorophosphate in *t*BuOMe was slowly added via a syringe over 4 h. ^b)^The aryl sulfonyl chloride was added in one portion. ^c)^Reaction in toluene. ^d)^Carbon, gray; hydrogen, ivory; fluorine, green‐yellow; nitrogen, light purple; oxygen, red; sulfur, yellow. Solvent molecules and other hydrogen atoms omitted for clarity. Hydrogen bonding interactions NH⋯O were indicated using a dashed line. The disorders of trifluoromethyl groups are shown. Thermal ellipsoids (atomic displacement parameters) are depicted at the 50% probability level.

Stereoselective rotaxane assembly is highly substrate‐dependent and reaction‐specific.^[^ ^32^, ^35^ ^]^ We investigated whether the spatial assembly could be preserved with changes in the reaction components. The reaction starting from *para*‐benzyloxy‐substituted phenyl sulfonyl chloride yielded chiral rotaxane **23** in 89:11 e.r. when sulfonyl chloride was added slowly, compared to 84:16 e.r. when it was added in one portion (Scheme 5a). The effectiveness of stereoinduction in the absence of the 3,5‐dimethyl groups of **15** also suggested that the approaching of aryl sulfonyl chloride is not determined by the steric property of the aryl group. On the other hand, replacing the trifluoromethyl groups of benzylamine with methoxycarbonyl groups resulted in 78:22 e.r. for rotaxane **24**.

The nucleophilic catalysis by the imidazolyl group and the spatial assembly are applicable to interlocking reactions beyond sulfonylation of amines. Switching the electrophile to diphenyl chlorophosphate yielded phosphoramidate rotaxane **25** with 72:28 e.r. Changes in the structure of the electrophile and the effectiveness of nucleophilic catalysis may contribute to reduced stereocontrol.

In addition, we probed the effect of shifting the ester linkage from the meta position to the ortho position of the benzo‐24‐crown‐8 scaffold. The change in the anchoring point influenced the positioning of the crown ether as indicated by a further shielded terminal methyl group upon loading of the crown ether (^1^H NMR δ = −1.7 ppm). Nonetheless, the transmission of stereochemical information remained effective (**26**, 75:25 e.r.), further demonstrating the adaptability of the cavitand.

Furthermore, we discovered that enantiopurity of cavitand **7** could be upgraded through the formation of a cavitand dimer (see Supporting Information for detailed synthetic procedure). Starting from enantioenriched **7** (98:2 e.r.), rotaxane **17** was accessed with 93:7 e.r. following the loading–assembly–unloading sequence.

We assign the stereochemistry of the major enantiomer of **17** through chemical correlation to the ester analog **27**, which was determined through X‐ray crystallography (Scheme 5b). Additionally, the relative stereochemistry of **22** (Scheme 4b) is supported by NOESY‐NMR analysis (see Figure S1). The stereochemistry of both **17** and **22** is consistent with the proposed mechanism of stereocontrol (Scheme 3b, iii). Therefore, the spatial assembly strategy provides predictable stereochemical outcomes as a result of the rationally designed chiral cavitands.

### Passive‐Template MPC Rotaxane Assembly

**Scheme 6:**
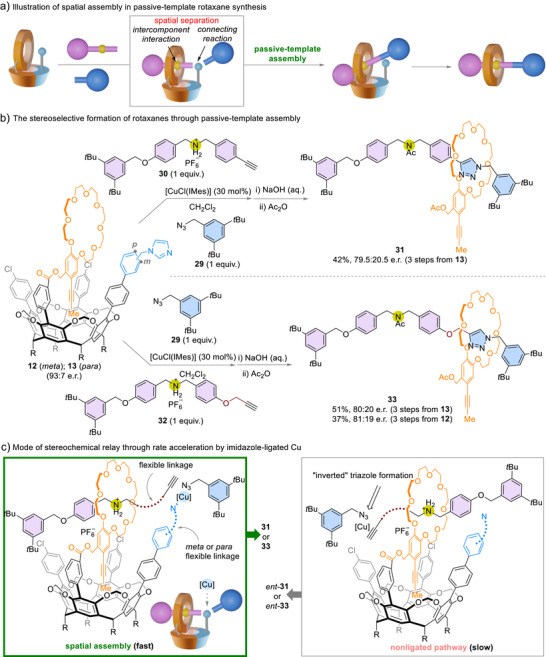
Passive‐template MPC rotaxane assembly. IMes: 1,3‐bis(2,4,6‐trimethylphenyl)imidazol‐2‐ylidene.

Unlike the macrocycle‐promoted reaction in active‐template assembly, the component positioning (i.e., the macrocycle–axle interactions) and component connecting (i.e., the reaction that conjoins the axle molecule) are decoupled in passive‐template assembly.^[^ ^45^ ^]^ The new cavitands are capable of transmitting the stereochemical information by encoding the disposition of the subcomponents rather than by confining the macrocycle–axle interactions within the range of influence of an existing chiral center. Therefore, the spatial assembly strategy developed for active‐template assembly could be amendable to passive‐template rotaxane synthesis (Scheme 6a).

We envisioned that the on‐rim imidazole group of cavitands could serve as a ligand for copper ions to direct the alkyne–azide cycloaddition.^[^ ^46^, ^47^ ^]^ This way, the triazole‐connected axle molecule is oriented selectively in the rotaxane products. In addition, diastereoselective pseudo‐rotaxane formation directed by the chiral cavitand could influence the stereochemical outcome. Indeed, starting from loaded cavitand **13** (93:7 e.r.), the Cu‐mediated alkyne–azide cycloaddition reaction between azide **29** and dialkylammonium‐tethered alkyne **30** afforded rotaxane **31** with 79.5:20.5 e.r. upon hydrolysis and acylation (Scheme 6b). The cavitand remains functional despite the temporal and spatial decoupling of the dialkylammonium–crown ether hydrogen bonding from the point of Cu‐mediated cycloaddition in the passive‐template assembly (Scheme 6c).

Moreover, the transmission of stereochemical information remained effective for alkyne **32**, in which the linker between the dialkylammonium ion and the alkyne is conformationally more flexible (Scheme 6b, **33**, 80:20 e.r.). Similar to the active‐templated sulfonamide formation, the reaction using meta‐(imidazolyl)methyl cavitand **12** (93:7 e.r.) gave comparable results (**33**, 81:19 e.r.). The tolerance towards structural flexibility shows that the spatial distribution of the Cu‐mediated reaction translates the stereochemical information encoded in the cavitand into the assembled products. This process does not rely on rigid preorganization of substrates through conformational confinement. The fact that cavitands initially optimized for active‐template rotaxane assembly confer stereoselectivity in a passive‐template mode further demonstrates the adaptability of the spatial assembly strategy.

## Conclusion

In summary, we have developed a spatial assembly strategy to access enantioenriched MPC rotaxanes. The rationally designed cavitands orchestrate the spatial arrangement of macrocycle and axle subcomponents, enabling the stereoselective construction of MPC rotaxanes with enantiomeric ratios up to 93:7. Notably, this spatial assembly strategy does not depend on confining the new stereogenic elements within the limited influence of preinstalled stereocenters; therefore, it is applicable to both active‐template and passive‐template rotaxane assembly despite their different spatial demands.

Our study demonstrates that it is feasible to transmit stereochemical information encoded in the spatial arrangement of multiple components, thereby conferring stereocontrol in the construction of mechanically interlocked molecules. Validation of this spatial assembly approach—without relying on inbuilt stereocenters—adds a new dimension to asymmetric synthesis. Given the modular design of hetero‐functionalized cavitands, we anticipate that spatial molecular assembly can be generalized by modulating catalytic functions and substrate‐interacting environments, thereby stimulating the engineering of supramolecular catalysts and artificial enzymes capable of precise and predictable spatial control.

## Conflict of Interests

The authors declare no conflict of interest.

## Supporting information



Supporting Information

Supporting Information

## Data Availability

The data that support the findings of this study are available in the Supporting Information of this article.
