# Supplementary material for: Spatial Assembly of Mechanically Planar Chiral Rotaxanes on Rationally Designed Cavitands
Source: Angew Chem Int Ed Engl. 2025 Oct 26;64(52):e202515927. doi: 10.1002/anie.202515927 (PMC12723448; doi:10.1002/anie.202515927)
Supplement: Supplementary file 1 — Supporting Information [file ANIE-64-e202515927-s001.pdf]

Spatial Assembly of Mechanically Planar Chiral Rotaxanes on Rationally Designed Cavitands

Liwen Xia[1], Wenyang Hong[1], Chong Tian[*,2], Ye Zhu[*,1]

[1]Department of Chemistry, Faculty of Science, National University of Singapore, 3 Science Drive 3, Singapore 117543

[2]Department of Pharmacy and Pharmaceutical Sciences, Faculty of Science, National University of Singapore, 18 Science Drive 4, Singapore 117559

Supporting Information

Table of contents

List of acronyms and abbreviations 3

General information 4

Divergent synthesis of functionalized cavitands 6

Preparation of crown ethers 18

Preparation of cavitands loaded with crown ether 28

Synthesis of rotaxane in toluene 43

Figure S1. Analysis of NMR spectra of **22.** 46

Preparation of cavitand 7 (93: 7 er) 52

General Procedure for rotaxane synthesis using slow addition 63

Enantiopurity upgrade for cavitand 7 (98:2 er) and rotaxane synthesis 75

Preparation of alkyne molecules for azide-alkyne cycloaddition 83

Rotaxane synthesis through azide-alkyne cycloaddition 86

References 93

NMR spectra 94

HPLC traces 127

X-ray crystallography data and CCDC deposition number 139

The chemical correlation between 17 and 27 142

List of acronyms and abbreviations

**dba**	dibenzylideneacetone
**dan**	naphthalene-1,8-diaminato
**dppb**	1,4-bis(diphenylphosphino)butane
**DBU**	1,8-diazabicyclo[5.4.0]undec-7-ene
**DCM**	dichloromethane
**DMAP**	4-(*N,N*-dimethylamino)pyridine
**DMF**	*N,N*-dimethylformamide
**DMSO**	dimethyl sulfoxide
**DIPEA**	*N,N*-diisopropylethylamine
**EDCl**	1-ethyl-3-(3-dimethylaminopropyl)carbodiimide
**NBS**	*N*-bromosuccinimide
**pin**	pinacolato
**TBSCl**	*tert*-butyldimethylsilyl chloride
**TBAF**	tetrabutylammonium fluoride
**2-MeTHF**	2-methyltetrahydrofuran

General information

Materials: Commercially available reagents and solvents were used as received. Commercial dry solvents (Aldrich Sure/Seal^{TM}) were sparged with nitrogen before used in catalytic reactions. Solvents used for column chromatography were analytical grade.

Methods: Unless otherwise noted, all experiments were set up under an atmosphere of nitrogen in a glovebox or using standard Schlenk techniques. Reactions were monitored by thin layer chromatography (TLC) or nuclear magnetic resonance (NMR) analysis. Flash column chromatography was performed using Tsingdao silica gel (60, particle size 300-400 mesh). Preparative thin layer chromatography (PTLC) was performed using Sanpont silica gel plate (20x20 cm, 0.5 mm). Yields refer to isolated yields after flash column chromatography purification.

Characterization: Products were characterized by means of nuclear magnetic resonance (NMR), mass spectrometry (MS), high performance liquid chromatography (HPLC), and optical rotation. ^{1}H, ^{13}C, and ^{19}F were recorded on Bruker 400 MHz and 500 MHz spectrometer with tetramethylsilane as internal standard. Chemical shifts were reported relative to tetramethylsilane (0 ppm) for ^{1}H NMR and relative to $CDCl_3$ (77.0 ppm) for ^{13}C NMR. ^{19}F spectra were calibrated from external standard ($CFCl_3$: 0 ppm). NMR data are reported as: chemical shift (parts per million, ppm), multiplicity (s = singlet, brs = broad singlet, d = doublet, t = triplet, q = quartet, m = multiplet), coupling constant (Hz), and integration. HPLC analysis was performed on a Shimadzu *i*-series HPLC system equipped with photodiode array (PDA) detector and Chiralcel and Chiralpak columns (0.46 cm∅× 25 cm). The wavelength that is an apex in the

spectrum was selected for analysis of the enantiomeric ratio (er) of each compound. Optical rotation ($[\alpha]_D^T$, deg•cm^3•g^{-1}•dm^{-1}) was measured on a Jasco DIP-1000 Digital Polarimeter at λ=589 nm in the given solvent at the indicated concentration (*c*, g/100 mL) and temperature (T, °C). HRMS-APCI was performed on Agilent 6546 LC-QTOF.

Divergent synthesis of functionalized cavitands

The synthesis of **2**

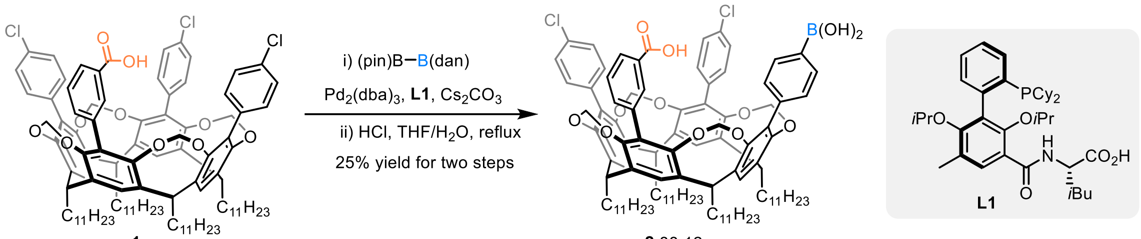

1 **2** 88:12 er

Under N_2 atmosphere in the glovebox, a 10 mL round bottom flask equipped with a magnetic stirring bar were added $Pd_2(dba)_3$ (8.0 mg, 0.0088 mmol), **L1** (14.0 mg, 0.0176 mmol), and 1.0 mL 2-MeTHF. The mixture was sealed with a cap (phenolic open top cap with red PTFE/white silicone septum), and stirred at room temperature for 20 min. The resulting metal-ligand complex solution was added to a sealed tube containing compound **1**[2] (260 mg, 0.16 mmol), (pin)B-B(dan) (56 mg, 0.19 mmol), Cs_2CO_3 (521 mg, 1.6 mmol, 10 equiv.), 7.0 mL 2-MeTHF and degassed H_2O (400 µL). The vial was then sealed, and the resulting solution was stirred at 60 °C for 14 h. The reaction mixture was then cooled to room temperature, and 1 M HCl aqueous solution was added to adjust to pH 1. The resulting mixture was then extracted with DCM three times. The combined organic phases were washed with water and brine, dried over Na_2SO_4, and concentrated *in vacuo* to afford the crude product. Then 1 mL 5 M HCl and 4 mL THF were added into the crude product. The mixture was stirred at reflux temperature (~80 °C) for four hours. The reaction mixture was then diluted with water and extracted with ethyl acetate three times. The combined organic layers were dried with Na_2SO_4 and concentrated *in vacuo* to afford the crude product. Chromatography purification on silica gel using hexane/ethyl acetate (1:1-1:2) as the eluents gave the purified product **2** (65 mg, 25% yield for two steps) as a yellow oil.

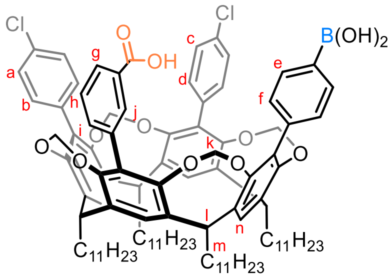

R_f = 0.3 (hexane/ethyl acetate =1:2).

$[\alpha]_D^{25}$ = – 6.1 (*c* 1.3 $CHCl_3$) for er =88:12

^{1}H NMR (400 MHz, $CDCl_3$) δ 8.17 – 8.06 (m, 2H, H_g and H_j), 7.76 – 7.49 (m, 3H, H_h and H_e), 7.45 – 7.27 (m, 6H, H_n and H_f), 7.30 – 7.22 (m, 2H), 7.15 – 7.07 (m, 2H, H_c), 7.07 – 6.93 (m, 5H, H_d), 5.36 – 5.06 (m, 4H, H_k), 4.91 – 4.79 (m, 4H, H_l), 4.55 – 4.33 (m, 1H, H_k), 4.29 – 4.13 (m, 3H, H_k), 2.48 – 2.26 (m, 8H, H_m, 4×CH_2), 1.58 – 1.40 (m, 16H, H_m, 8×CH_2), 1.40 – 1.19 (m, 56H, H_m, 28×CH_2), 0.89 (t, *J* = 6.5 Hz, 12H, H_m, 4×CH_3).

Note: 20 H (6.93 – 8.17 ppm) for aryl groups in compound **2**; H_a, H_b, and H_i resonate at 7.30–7.22 ppm and 7.07–6.93 ppm, but their signals could not be clearly distinguished.

^{13}C NMR (126 MHz, $CDCl_3$) δ 152.7, 152.6, 152.5, 138.6, 138.5, 138.44, 138.35, 138.3, 135.1, 135.0, 133.4, 133.22, 133.19, 132.5, 132.41, 132.35, 131.3, 131.2, 129.63, 129.57, 129.23, 129.16, 129.0, 128.65, 128.55, 128.48, 128.4, 128.3, 128.2, 128.12, 128.07, 120.41, 120.37, 120.2, 120.1, 100.5, 100.3, 100.2, 62.2, 44.9, 37.1, 32.0, 30.5, 29.9, 29.8, 29.4, 29.0, 28.0, 22.7, 14.1.

Note*:* The observed overlap of ^{13}C NMR signals in the 120.1–152.7 ppm region is likely a result of the symmetry presents in the cavitand scaffold. 42 non-equivalent aromatic carbons in theory, 35 signals recorded; The observed overlap of ^{13}C NMR signals in the 14.1–44.9 ppm region is likely a result of the symmetry present in the cavitand scaffold. 44 non-equivalent carbons for the $C_{11}H_{23}$ groups in theory, 11 signals recorded.

HRMS (m/z, APCI): Calcd. for Chemical Formula: $[C_{101}H_{128}B^{35}Cl_2O_{12}]^+[M+H]^+$: 1613.8798, Found: 1613.8785.

The synthesis of **S1** for the determination of the enantiopurity of **2**.

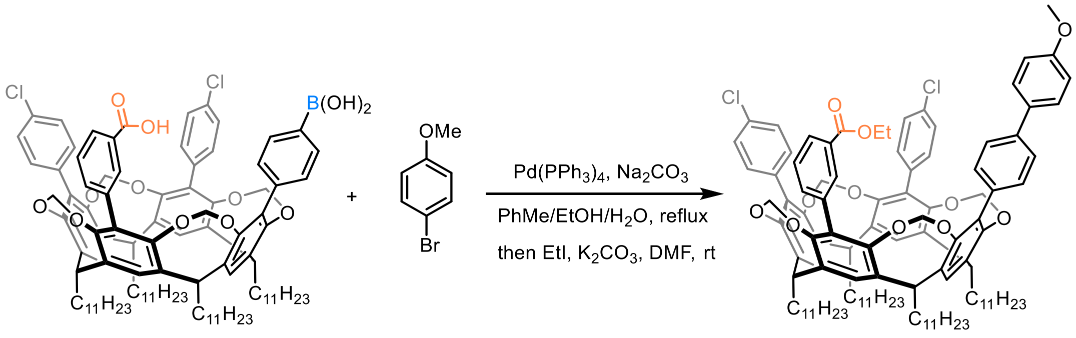

2 **S1**

Under N_2 atmosphere, to a solution of compound **2** (20 mg, 0.012 mmol) in PhMe/EtOH/H_2O (0.4 mL/0.1 mL/0.1 mL) were added 4-bromoanisole (7 mg, 0.036 mmol, 3 equiv.), $Pd(PPh_3)_4$ (1.4 mg, 10 mol%) and Na_2CO_3 (6 mg, 0.06 mmol, 5 equiv.). The reaction mixture was stirred under reflux condition overnight. The reaction mixture was cooled down to room temperature and diluted with 10 mL ethyl acetate. The organic phase was washed with saturated NaCl, dried with Na_2SO_4 and concentrated *in vacuo* to afford the crude product which can be directly used in the next step. The crude product was dissolved into DMF (5 mL). K_2CO_3 (8 mg, 0.06 mmol, 5 equiv.) and EtI (9 mg, 0.06 mmol, 5 equiv.) were added into the solution. The reaction was stirred at room temperature for 2 h. The reaction mixture was diluted with 10 mL ethyl acetate and washed with water (60 mL, 20 mL*3). The organic phase was washed with saturated NaCl, dried with Na_2SO_4 and concentrated *in vacuo* to afford the crude product. Chromatography purification on silica gel using hexane/ethyl acetate (20:1) as the eluents gave the purified product **S1** (16 mg, 75% yield) as a yellow oil.

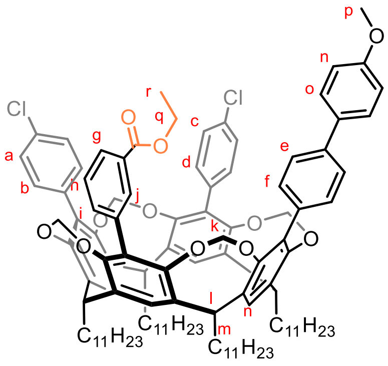

R_f = 0.6 (hexane/ethyl acetate =10:1).

^{1}H NMR (400 MHz, $CDCl_3$) δ 7.95 (dt, *J* = 7.6, 1.4 Hz, 1H, H_g), 7.56 (s, 1H, H_j), 7.50 (dd, *J* = 8.6 Hz, 4H, H_e and H_o), 7.41 (d, *J* = 7.6 Hz, 1H, H_i), 7.38 – 7.30 (m, 6H, H_n), 7.30 – 7.27 (m, 3H), 7.06 (d, *J* = 8.2 Hz, 2H, H_b), 7.01 (d, *J* = 8.4 Hz, 2H, H_a), 6.98 (d, *J* = 8.4 Hz, 2H, H_n), 6.94 (d, *J* = 8.8 Hz, 2H, H_f), 5.30 – 5.16 (m, 4H, H_k), 4.90 – 4.80 (m, 4H, H_l), 4.35 (q, *J* = 7.2 Hz, 2H, H_q), 4.27 – 4.17 (m, 4H, H_k), 3.75 (s, 3H, H_p), 2.39 – 2.28 (m, 8H, H_m, 4×CH_2), 1.50 – 1.42 (m, 16H, H_m, 8×CH_2), 1.36 – 1.27 (m, 59H, H_m and H_r, 28×CH_2+CH_3), 0.89 (t, *J* = 6.7 Hz, 12H, H_m, 4×CH_3).

Note: 24 H (6.94 – 7.95 ppm) for aryl groups in compound **S1**; H_c, H_d, and H_h resonate at 7.38–7.27 ppm, but their signals could not be clearly distinguished.

^{13}C NMR (126 MHz, $CDCl_3$) δ 166.5, 159.2, 152.8, 152.7, 152.6_3, 152.5_9, 152.5_5, 152.5_0, 139.4, 138.5, 138.4_5, 138.3_9, 138.3_4, 138.3_1, 138.2, 135.1, 134.3, 133.2, 133.1, 132.5, 132.4, 132.1, 131.3, 131.2, 130.3, 129.9, 129.2, 128.5, 128.4, 128.2_4, 128.1_6, 128.0, 126.1, 120.2, 120.1, 119.8, 114.2, 100.6_0, 100.5_6, 100.4, 61.2, 55.2, 37.1, 32.0, 31.5, 30.4, 29.9, 29.8, 29.4, 28.0, 22.7, 14.3, 14.1.

Note*:* The observed overlap of ^{13}C NMR signals in the 114.2–159.2 ppm region is likely a result of the symmetry presents in the cavitand scaffold. 46 non-equivalent aromatic carbons in theory, 36 signals recorded; The observed overlap of ^{13}C NMR signals in the 14.1–37.1 ppm region is likely a result of the symmetry present in the cavitand scaffold. 44 non-equivalent carbons for the $C_{11}H_{23}$ groups in theory, 10 signals recorded.

HRMS (m/z, APCI): Calcd. for Chemical Formula: $[C_{108}H_{133}{}^{35}Cl_2O_{11}]^+[M+H]^+$: 1675.9147, Found: 1675.9153.

HPLC analysis: Daicel Chiralpak IF, hexane/*iso*-propanol = 99: 1, T = 40 °C, 1.0 mL/min, λ = 254 nm, retention time: 20.49 min (major) and 24.32 min (minor).

The synthesis of functionalized cavitands via Suzuki coupling of 2

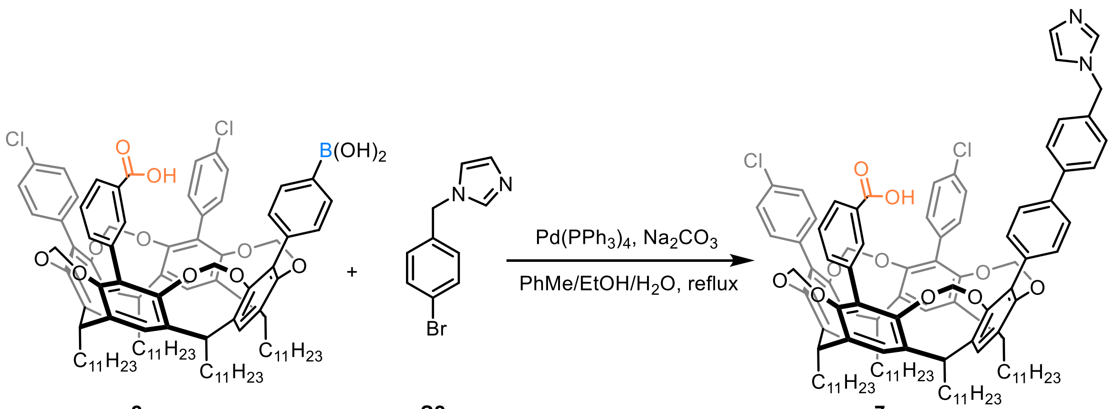

2 **S2** **7**

Under N_2 atmosphere, to a solution of compound **2** (20 mg, 0.012 mmol) in PhMe/EtOH/H_2O (0.4 mL/0.1 mL/0.1 mL) were added **S2** (9 mg, 0.036 mmol, 3 equiv.), $Pd(PPh_3)_4$ (1.4 mg, 10 mol%) and Na_2CO_3 (6 mg, 0.06 mmol, 5 equiv.). The reaction mixture was stirred under reflux condition overnight. The reaction mixture was cooled down to room temperature and diluted with 10 mL ethyl acetate. The organic phase was washed with saturated NaCl, dried with Na_2SO_4 and concentrated *in vacuo* to afford the crude product. Chromatography purification on silica gel using chloroform/methanol (20:1) as the eluents gave the purified product **7** (14 mg, 65% yield) as a colorless oil.

Note: The presence of both acidic (COOH) and basic (*N*-heterocycle) groups in cavitands **3** to **7** complicates their isolation and purification using silica gel chromatography. The products isolated from the Suzuki coupling reaction of **2**, although contain small quantities of impurities from the Pd-catalyzed reactions, can be directly used in the subsequent esterification reactions with crown ether **8** as described in the main text. The resulting loaded cavitands **9** to **13** can be readily purified using silica gel chromatography due to change in polarity once the crown ether is incorporated. Nevertheless, we have obtained small quantities of **3** to **6** for ^{1}H-NMR analysis through multiple preparative TLC. For **4** and **5**, we had to convert the initially isolated carboxylic acid products to corresponding ethyl esters for further purification. Compound **7** was prepared via direct Suzuki coupling of **1** and characterized subsequently.

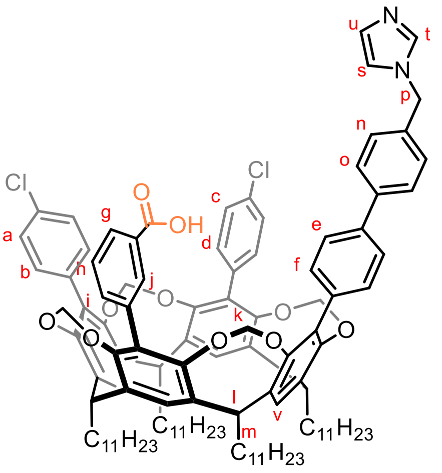

R$_f$ = 0.3 (chloroform/methanol= 20:1).

^{1}H NMR (500 MHz, $CDCl_3$) δ 8.10 – 7.88 (br, 1H, H_t), 7.76 – 7.32 (m, 16H, $H_{n,o,e,f,v}$), 7.22 – 7.09 (m, 5H, $H_{a,b}$), 7.08 – 6.92 (br, 3H), 6.76 (d, J = 8.6 Hz, 1H, H_j), 5.30 – 5.0 (m, 6H, H_k and H_p), 4.90 – 4.76 (m, 4H, H_l), 4.47 – 4.17 (m, 4H, H_k), 2.43 – 2.26 (m, 8H, H_m, 4×CH_2), 1.58 – 1.44 (m, 16H, H_m, 8×CH_2), 1.39 – 1.20 (m, 56H, H_m, 28×CH_2), 0.94 – 0.89 (m, 12H, H_m, 4×CH_3).

Note: 27 H (6.94 – 8.10 ppm) for aryl groups in compound **7**; H_n, $H_{c,d}$, H_o and H_v resonate at 7.44 –7.27 and 7.08 – 6.92 ppm, but their signals could not be clearly distinguished.

^{13}C NMR (126 MHz, $CDCl_3$) δ 155.1, 152.7, 152.6, 152.5, 138.3, 133.1, 132.5, 132.5, 131.3, 130.4, 129.3, 128.9, 128.8, 128.6, 128.3, 128.1, 127.74, 127.70, 126.6, 120.2, 120.1, 120.0, 119.9, 116.8, 100.4, 100.32, 100.27, 100.2, 60.5, 37.1, 32.0, 30.4, 29.9, 29.8, 29.4, 28.0, 22.7, 14.1.

Note*:* The observed overlap of ^{13}C NMR signals in the 116.8–152.7 ppm region is likely a result of the symmetry presents in the cavitand scaffold. 49 non-equivalent aromatic carbons in theory, 23 signals recorded; The observed overlap of ^{13}C NMR signals in the 14.1–37.1 ppm region is likely a result of the symmetry present in the cavitand scaffold. 44 non-equivalent carbons for the $C_{11}H_{23}$ groups in theory, 9 signals recorded.

HRMS (m/z, APCI): Calcd. for Chemical Formula: $[C_{111}H_{135}{}^{35}Cl_2N_2O_{10}]^+[M+H]^+$: 1725.9488, Found: 1725.9477

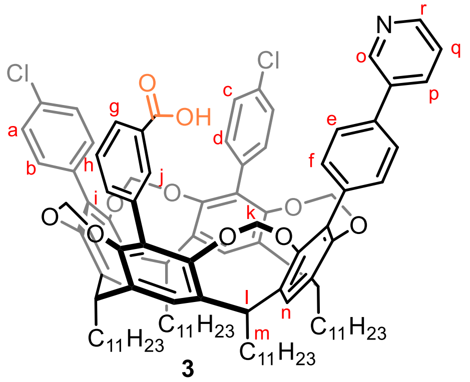

3

R$_f$ = 0.6 (hexane/ethyl acetate = 1: 1)

^{1}H NMR (400 MHz, Chloroform-*d*) δ 8.93 (s, 1H, H_o), 8.52 (s, 1H, H_r), 8.07 – 8.00 (m, 1H, H_p), 7.94 (d, *J* = 7.2 Hz, 1H, H_g), 7.57 (s, 1H, H_j), 7.53 (d, *J* = 8.1 Hz, 2H, H_e), 7.48 (d, *J* = 3.9 Hz, 2H), 7.40 – 7.34 (m, 5H), 7.32 – 7.28 (m, 3H), 7.17 (d, *J* = 7.9 Hz, 2H, H_f), 7.05 (d, *J* = 8.3 Hz, 2H, H_a), 7.00 (d, *J* = 8.3 Hz, 2H, H_b), 5.29 – 5.18 (m, 4H, H_k), 4.92 – 4.83 (m, 4H, H_l), 4.32 – 4.20 (m, 4H, H_k), 2.42 – 2.34 (m, 8H, H_m, 4×CH_2), 1.56 – 1.46 (m, 16H, H_m, 8×CH_2), 1.36 – 1.29 (m, 56H, H_m, 28×CH_2), 0.93 – 0.90 (m, 12H, H_m, 4×CH_3).

Note: 24 H (7.00 – 8.93 ppm) for aryl groups in compound **3**; H_c, H_d, H_h and H_q resonate at 7.48–7.28 ppm, but their signals could not be clearly distinguished.

^{13}C NMR (126 MHz, $CDCl_3$) δ 152.7, 152.6_1, 152.5_5, 152.5_1, 138.5, 138.4_2, 138.3_9, 138.3_7, 138.3, 134.4, 133.2, 132.0, 131.3_2, 131.2_9, 130.7, 129.9, 129.8, 128.9, 128.8, 128.5, 128.1, 126.7, 120.1_9, $120. 1_8$, $120. 1_5$, 120.1, $100. 5_1$, $100. 4_9$, $100. 4_5$ 37.1, 32.0, 29.9, $29. 8_1$, $29. 7_7$, 29.4, 28.0, 22.7, 14.1.

Note*:* The observed overlap of ^{13}C NMR signals in the 120.1–152.7 ppm region is likely a result of the symmetry presents in the cavitand scaffold. 47 non-equivalent aromatic carbons in theory, 28 signals recorded; The observed overlap of ^{13}C NMR signals in the 14.1–37.1 ppm region is likely a result of the symmetry present in the cavitand scaffold. 44 non-equivalent carbons for the $C_{11}H_{23}$ groups in theory, 11 signals recorded.

HRMS (m/z, APCI): Calcd. for Chemical Formula: $C_{106}H_{130}{}^{35}Cl_2NO_{10}$ $[M+H]^+$: 1646.9066, Found: 1646.9080.

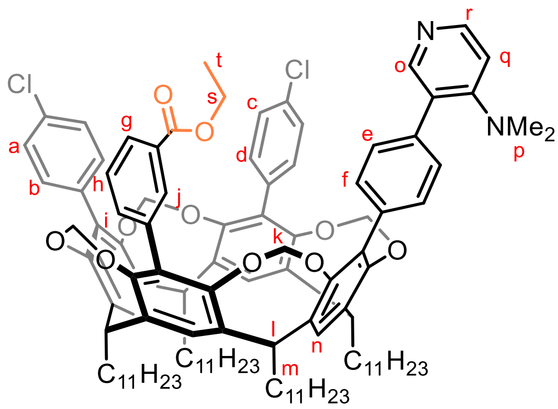

4(ester)

R$_f$ = 0.3 (100% ethyl acetate)

^{1}H NMR (400 MHz, Chloroform-*d*) δ 8.26 (d, *J* = 5.9 Hz, 1H, H_o), 8.19 (s, 1H), 7.96 (d, *J* = 7.6 Hz, 1H, H_r), 7.54 (s, 1H, H_j), 7.43 (d, *J* = 7.6 Hz, 1H, H_q), 7.38 (d, *J* = 8.2 Hz, 3H, H_e), 7.35 – 7.27 (m, 8H, H_n), 7.08 (d, *J* = 8.2 Hz, 2H, H_f), 7.02 (d, *J* = 8.4 Hz, 2H, H_a), 6.97 (d, *J* = 8.4 Hz, 2H, H_b), 6.74 (d, *J* = 6.0 Hz, 1H), 5.29 – 5.15 (m, 4H, H_k), 4.84 (q, *J* = 7.9 Hz, 4H, H_l), 4.36 (q, *J* = 7.1 Hz, 2H, H_s), 4.30 – 4.14 (m, 4H, H_k), 2.65 (s, 6H, H_p), 2.35 (s, 8H, H_m, 4×CH_2), 1.51 – 1.41 (m, 16H, H_m, 8×CH_2), 1.35 – 1.26 (m, 59H, H_m and H_t, 28×CH_2+CH_3), 0.89 (t, *J* = 6.7 Hz, 12H, H_m, 4×CH_3).

Note: 23 H (6.74 – 8.26 ppm) for aryl groups in compound **4**; H_c, H_d, H_i and H_h resonate at 7.38–7.27 ppm, but their signals could not be clearly distinguished.

^{13}C NMR (126 MHz, $CDCl_3$) δ 171.1, 166.4, 152.7, 152.6$_5$, 152.5$_9$, 152.5, 152.4, 141.5, 140.8, 139.8, 139.0, 138.6$_1$, 138.5$_6$, 138.5, 138.4$_4$, 138.3$_9$, 133.3$_2$, 133.2$_8$, 132.9, 132.4, 132.4, 131.2$_5$, 131.1$_9$, 130.2, 130.0, 129.3, 129.2, 129.0, 128.8, 128.5$_4$, 128.4$_9$, 128.4$_6$, 128.2$_3$, 128.2$_1$, 126.7, 126.3, 126.0, 125.6, 120.5, 120.2$_2$, 120.1$_9$, 119.9, 100.5, 100.4, 65.3, 61.1, 60.5, 37.1, 32.0, 30.5, 30.4, 29.9, 29.8$_3$, 29.7$_9$, 29.5, 28.0, 22.8, 14.1.

Note*:* The observed overlap of ^{13}C NMR signals in the 120.2–166.4 ppm region is likely a result of the symmetry presents in the cavitand scaffold. 47 non-equivalent aromatic carbons in theory, 41 signals recorded; The observed overlap of ^{13}C NMR signals in the 14.1–37.1 ppm region is likely a result of the symmetry present in the cavitand scaffold. 44 non-equivalent carbons for the $C_{11}H_{23}$ groups in theory,

11 signals recorded.

HRMS (m/z, APCI): Calcd. for Chemical Formula: $C_{106}H_{139}{}^{35}Cl_2N_2O_{10}$ $[M+H]^+$: 1717.9801, Found: 1717.9806.

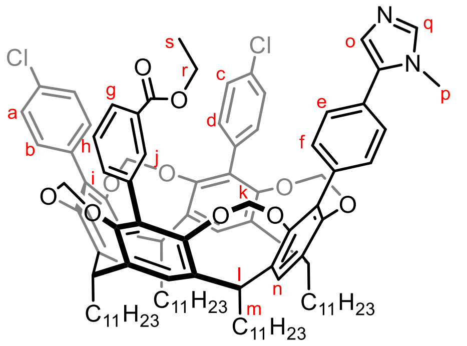

5 (ester)

R_f = 0.5 (chloroform/methanol= 20:1)

^{1}H NMR (400 MHz, Chloroform-*d*) δ 7.95 (dt, *J* = 7.6, 1.4 Hz, 1H, H_g), 7.62 (s, 1H, H_q), 7.56 (s, 1H, H_j), 7.44 – 7.36 (m, 2H), 7.35 – 7.27 (m, 10H), 7.14 – 7.09 (m, 3H), 7.03 (d, *J* = 8.4 Hz, 2H, H_a), 6.97 (d, *J* = 8.4 Hz, 2H, H_b), 5.27 – 5.16 (m, 4H, H_k), 4.88 – 4.81 (m, 4H, H_l), 4.35 (q, *J* = 7.1 Hz, 2H, H_r), 4.28 – 4.19 (m, 4H, H_k), 3.69 (s, 3H, H_p), 2.41 – 2.30 (m, 8H, H_m, 4×CH_2), 1.53 – 1.41 (m, 16H, H_m, 8×CH_2), 1.35 – 1.24 (m, 59H, H_m and H_s, 28×CH_2+CH_3), 0.89 (t, *J* = 6.7 Hz, 12H, H_m, 4×CH_3).

Note: 22 H (6.97 – 7.95 ppm) for aryl groups in compound **5**; H_{c-f}, H_i and H_h resonate at 7.44–7.09 ppm, but their signals could not be clearly distinguished.

^{13}C NMR (126 MHz, $CDCl_3$) δ 166.6, 152.7, 152.6, 152.5, 139.1, 138.39, 138. 35, 138.33, 138.29, 135.0, 134.4, 133.2, 132.5, 132.4, 131.3, 131.2, 130.4, 129.9, 128.8, 128.6, 128.5, 128.5, 128.3, 128.2, 127.8, 120.23, 120.18, 120.15, 120.11, 100.49, 100.46, 100.4, 76.8, 61.3, 37.1, 32.9, 32.0, 30.4, 29.9, 29.80, 29.76, 29.4, 28.0, 22.7, 14.3, 14.1.

Note*:* The observed overlap of ^{13}C NMR signals in the 120.11–152.7 ppm region is likely a result of the symmetry presents in the cavitand scaffold. 45 non-equivalent aromatic carbons in theory, 29 signals recorded; The observed overlap of ^{13}C NMR signals in the 14.1–37.1 ppm region is likely a result of the symmetry present in the cavitand scaffold. 44 non-equivalent carbons for the $C_{11}H_{23}$ groups in theory, 12 signals recorded.

HRMS (m/z, APCI): Calcd. for Chemical Formula: $C_{107}H_{135}{}^{35}Cl_2N_2O_{10}$ $[M+H]^+$: 1677.9488, Found: 1677.9492.

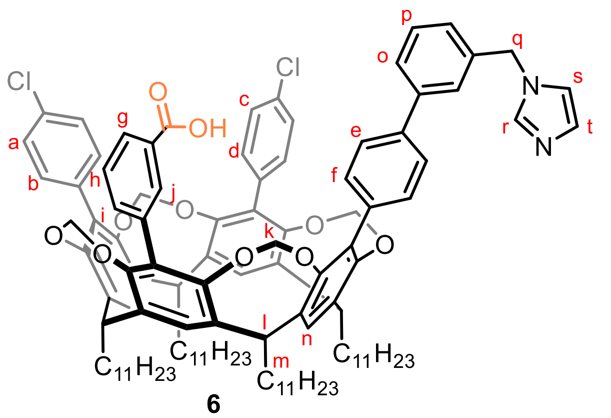

6

$\mathbf{R_f}$ = 0.4 (chloroform/methanol= 20:1)

^{1}H NMR (400 MHz, Chloroform-*d*) δ 8.12 – 8.01 (m, 2H, H_r and H_g), 7.46 (dd, *J* = 16.5, 8.2 Hz, 4H, H_e and H_f), 7.41 – 7.34 (m, 3H), 7.31 – 7.25 (m, 5H, H_n), 7.23 – 7.20 (m, 3H), 7.14 – 7.09 (m, 4H, H_t), 7.04 (d, *J* = 8.1 Hz, 2H, H_b), 6.93 (dd, *J* = 14.1, 8.4 Hz, 3H, H_a), 6.94 – 6.91 (m, 1H, H_s), 5.25 – 5.16 (m, 4H, H_k), 5.06 (s, 2H, H_q), 4.90 – 4.79 (m, 4H, H_l), 4.31 – 4.14 (m, 4H, H_k), 2.35 – 2.21 (m, 8H, H_m, 4×CH_2), 1.51 –1.41 (m, 16H, H_m, 8×CH_2), 1.38 – 1.25 (m, 56H, H_m, 28×CH_2), 0.84 – 0.79 (m, 12H, H_m, 4×CH_3).

Note: 27 H (6.91 – 8.12 ppm) for aryl groups in compound **6**; $H_{c\text{-}f}$, $H_{o,p}$, H_i and H_h resonate at 7.41 –7.34 and 7.23 – 7.09 ppm, but their signals could not be clearly distinguished.

^{13}C NMR (126 MHz, $CDCl_3$) δ 171.2, 152.7_3, 152.6_9, 152.6_3, 152.5_8, 152.5_2, 152.4_7, 141.7, 139.0, 138.5, 138.4, 135.8, 133.2, 132.4, 131.2, 130.3, 129.6, 129.4, 128.6, 128.2, 127.4, 126.7, 120.1, 120.1, 119.9, 119.5, 100.5, 100.4_4, 100.4_0, 60.5, 51.4, 37.1, 30.4, 29.9, 29.8_1, 29.7_7, 29.4, 28.0, 22.7, 14.2, 14.1.

Note*:* The observed overlap of ^{13}C NMR signals in the 119.5 –152.7_3 ppm region is likely a result of the symmetry presents in the cavitand scaffold. 51 non-equivalent aromatic carbons in theory, 25 signals recorded; The observed overlap of ^{13}C NMR signals in the 14.1–37.1 ppm region is likely a result of the symmetry present in the cavitand scaffold. 44 non-equivalent carbons for the $C_{11}H_{23}$ groups in theory, 10 signals recorded.

HRMS (m/z, APCI): Calcd. for Chemical Formula: $C_{111}H_{135}{}^{35}Cl_2N_2O_{10}$ $[M+H]^+$: 1725.9488, Found: 1725.9488.

Preparation of crown ethers

The synthesis of **S4**

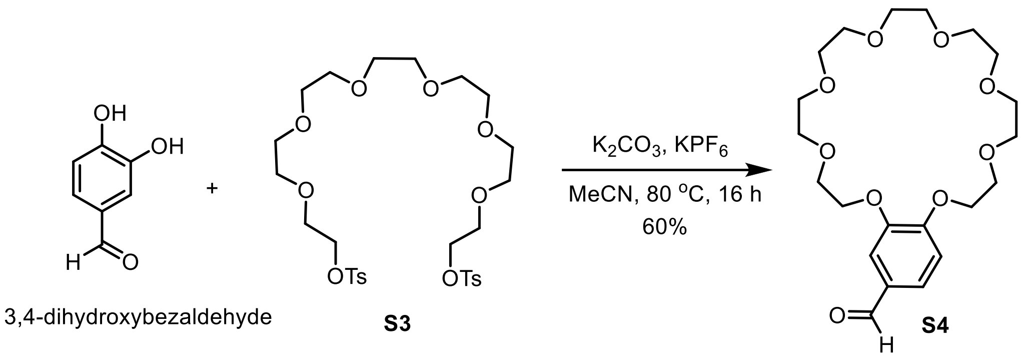

To a solution of **S3**[1] (462 mg, 0.72 mmol) in 10 mL dry MeCN were added 3,4-dihydroxybenzaldehyde (100 mg, 0.72 mmol), K_2CO_3 (298 mg, 2.16 mmol, 3 equiv.) and KPF_6 (395 mg, 2.16 mmol, 3 equiv.). The reaction mixture was stirred at 80 °C for 16 h. After filtration, the solution was concentrated *in vacuo*. The residue was dissolved in 20 mL ethyl acetate and washed with 10 mL water and 10 mL saturated NaCl. The combined organic layers were dried with Na_2SO_4 and concentrated *in vacuo* to afford the crude product. Chromatography purification on silica gel using ethyl acetate/methanol (20:1) as the eluents gave the purified product **S4** (185 mg, 60%) as a colorless oil.

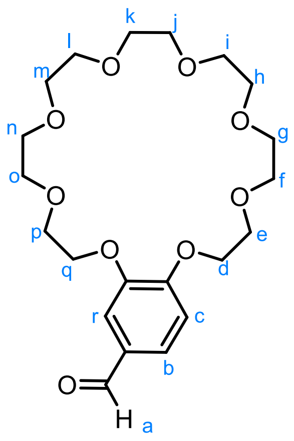

R$_f$ = 0.25 (ethyl acetate/methanol = 20: 1).

^{1}H NMR (500 MHz, $CDCl_3$) δ 9.86 (s, 1H, H_a), 7.52 (dd, J = 8.2, 1.8 Hz, 1H, H_b), 7.48 (d, *J* = 1.8 Hz, 1H, H_r), 7.08 (d, *J* = 8.2 Hz, 1H, H_c), 4.37 – 4.33 (m, 2H, H_q), 4.32 – 4.28 (m, 2H, H_d), 3.95 – 3.90 (m, 2H, H_p), 3.88 – 3.83 (m, 2H, H_e), 3.73 – 3.63 (m, 20H, $H_{f\text{-}o}$).

^{13}C NMR (126 MHz, $CDCl_3$) δ 190.9, 130.3, 126.9, 112.3, 111.4, 71.2, 71.1, 70.8, 70.7, 70.6, 69.6, 69.5, 69.13, 69.07.

Note: The observed overlap of ^{13}C NMR signals stacking in the 69.07–71.2 ppm region is the glycol groups in crown ether. 14 non-equivalent carbons in theory, 9 signals recorded.

HRMS (m/z, APCI): Calcd. for Chemical Formula: $[C_{21}H_{33}O_9]^+[M+H]^+$: 428.2046, Found: 428.2038.

The synthesis of **S5**

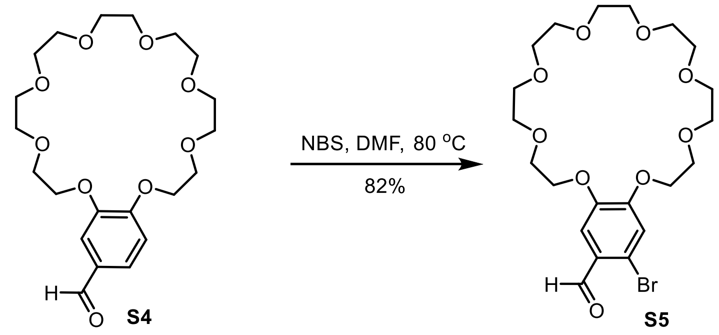

To a solution of **S4** (141 mg, 0.33 mmol) in 5 mL dry DMF was added NBS (89 mg, 0.49 mmol, 1.5 equiv.). The reaction mixture was stirred at 80 °C for 7 h. The solution was diluted with 20 mL ethyl acetate and washed with water (20 mL×2). The combined organic layers were dried with Na_2SO_4 and concentrated *in vacuo* to afford the crude product. Chromatography purification on silica gel using ethyl acetate/methanol (20:1) as the eluents gave the purified product **S5** (137 mg, 82%) as a light-yellow oil.

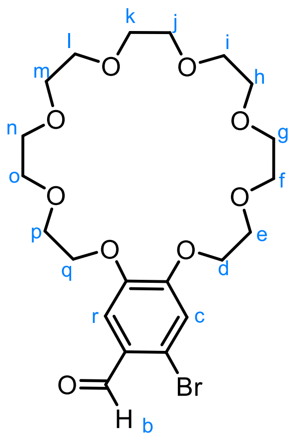

R_f = 0.2 (ethyl acetate/methanol = 20: 1).

^{1}**H NMR** (400 MHz, $CDCl_3$) δ 10.16 (s, 1H, H_b), 7.41 (s, 1H, H_r), 7.06 (s, 1H, H_c), 4.24 – 4.21 (m, 2H, H_q), 4.21 – 4.17 (m, 2H, H_d), 3.96 – 3.93 (m, 2H, H_p), 3.92 – 3.89 (m, 2H, H_e), 3.80 – 3.75 (m, 4H, $H_{f\text{-}o}$), 3.72 – 3.69 (m, 4H, $H_{f\text{-}o}$), 3.66 (d, *J* = 8.4 Hz, 12H, $H_{f\text{-}o}$).

^{13}**C NMR** (126 MHz, $CDCl_3$) δ 190.8, 126.7, 120.4, 117.1, 112.6, 71.3, 71.2, 70.9, 70.8, 70.7, 69.6, 69.5, 69.3.

Note: The observed overlap of ^{13}C NMR signals stacking in the 69.3–71.3 ppm region is the glycol groups in crown ether. 14 non-equivalent carbons in theory, 8 signals recorded.

HRMS (m/z, APCI): Calcd. for Chemical Formula: $[C_{21}H_{32}O_9{}^{79}Br]^+[M+H]^+$:506.1151, Found: 506.1147.

The synthesis of **S6**

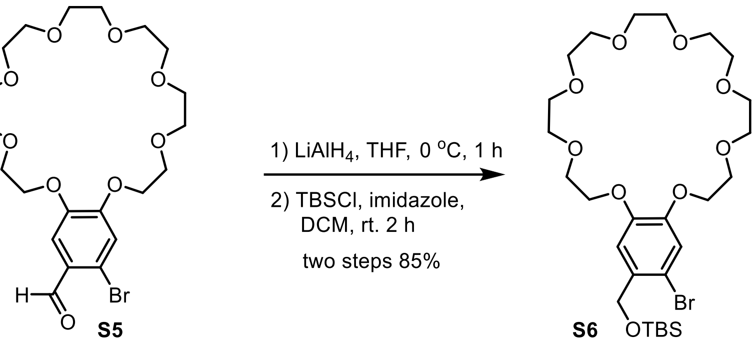

Under N_2 atmosphere, to a solution of **S5** (137 mg, 0.27 mmol) in dry THF (5 mL) was added $LiAlH_4$ (15 mg, 0.4 mmol, 1.5 equiv.) at 0 °C. The reaction mixture was stirred at this temperature for 1 h. When TLC indicated the consume of starting material, the reaction mixture was poured into an iced saturated Na_2SO_4 and 20 mL ethyl acetate were added subsequently. The combined organic phase was washed with saturate NaCl and dried with Na_2SO_4. The solution was then concentrated *in vacuo* to afford the crude product. The crude product was used directly into next step without purification. The crude product was dissolved into 10 mL dry DCM. Imidazole (27 mg, 0.4 mmol, 1.5 equiv.) and TBSCl (48 mg, 0.32 mmol, 1.2 equiv.) were added into the above solution and stirred at room temperature for 2 h. The reaction mixture was diluted with 10 mL DCM and washed with saturated NaH_2PO_4 and saturated NaCl subsequently. Chromatography purification on silica gel using ethyl acetate/methanol (20:1) as the eluents gave the purified product **S6** (143 mg, 85%) as a colorless oil.

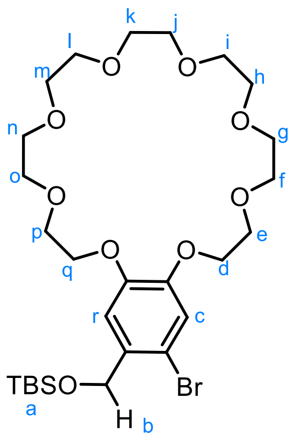

R_f = 0.2 (ethyl acetate/methanol = 10: 1).

^{1}H NMR (500 MHz, $CDCl_3$) δ 7.10 (s, 1H, H_c), 7.01 (s, 1H, H_r), 4.63 (s, 2H, H_b), 4.16 – 4.11 (m, 4H, H_q and H_d), 3.88 (q, *J* = 4.6 Hz, 4H, H_p and H_e), 3.78 – 3.74 (m, 4H, $H_{f\text{-}o}$), 3.71 – 3.68 (m, 4H, $H_{f\text{-}o}$), 3.67 – 3.64 (m, 12H, $H_{f\text{-}o}$), 0.95 (s, 9H, H_a, *t*Bu), 0.11 (s, 6H, H_a, 2×CH_3).

^{13}C NMR (126 MHz, $CDCl_3$) δ 148.4, 148.0, 133.4, 118.3, 113.4, 111.2, 70.8, 70.6, 70.5, 69.6, 69.5, 69.1, 64.2, 25.9, 18.4, -5.3.

Note: The observed overlap of ^{13}C NMR signals stacking in the 69.1–70.8 ppm region is the glycol groups in crown ether. 14 non-equivalent carbons in theory, 6 signals recorded.

HRMS (m/z, APCI): Calcd. for Chemical Formula: $[C_{27}H_{48}O_9{}^{79}BrSi]^+[M+H]^+$:623.2173, Found: 623.2165.

The synthesis of **S7**

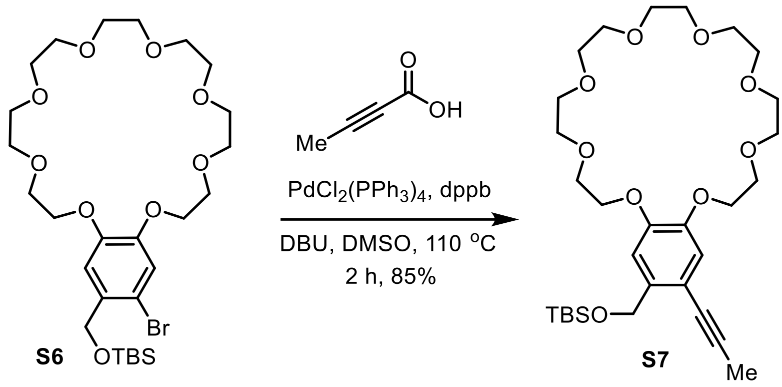

Under N_2 atmosphere, to a solution of **S6** (590 mg, 0.95 mmol) in 15 mL dry DMSO were added 2-butynoic acid (96 mg, 1.14 mmol, 1.2 equiv.), $PdCl_2(PPh_3)_4$ (33 mg, 0.047 mmol, 5 mol%), dppb (41 mg, 0.095 mmol, 10 mol%) and DBU (433 mg, 2.85 mmol, 3 equiv.). The solution was heat at 110 ^oC and stirred at this temperature for 2 h. When TLC indicated the consume of starting material, the reaction mixture was cooled to room temperature and diluted with 20 mL ethyl acetate. The combined organic phase was washed with saturated NaCl, dried with Na_2SO_4 and concentrated *in vacuo* to afford the crude product. Chromatography purification on silica gel using chloroform/methanol (20:1) as the eluents gave the purified product **S7** (469 mg, 85%) as a light-yellow oil.

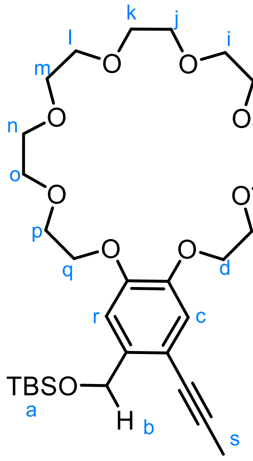

$\mathbf{R_f}$ = 0.3 (chloroform/methanol = 10: 1).

^{1}H NMR (500 MHz, $CDCl_3$) δ 7.03 (s, 1H, H_c), 6.87 (s, 1H, H_r), 4.77 (s, 2H, H_b), 4.18 – 4.15 (m, 2H, H_q), 4.14 – 4.10 (m, 2H, H_d), 3.91 – 3.87 (m, 4H, H_p and H_e), 3.79 – 3.76 (m, 4H, $H_{f\text{-}o}$), 3.74 – 3.70 (m, 4H, $H_{f\text{-}o}$), 3.68 – 3.65 (m, 8H, $H_{f\text{-}o}$), 3.66 – 3.64 (m, 4H, $H_{f\text{-}o}$), 2.06 (s, 3H, H_s), 0.94 (s, 9H, H_a, *t*Bu), 0.10 (s, 6H, H_a, 2×CH_3).

^{13}C NMR (126 MHz, $CDCl_3$) δ 148.9, 146.9, 137.0, 117.6, 113.2, 112.7, 111.8, 89.0, 71.0, 70.9, 70.7, 70.58, 70.55, 69.7, 69.6, 69.4, 68.9, 63.0, 26.0, 18.4, 4.5, -5.2.

Note: The observed overlap of ^{13}C NMR signals stacking in the 68.9–71.0 ppm region is the glycol groups in crown ether. 14 non-equivalent carbons in theory, 9 signals recorded.

HRMS (m/z, APCI): Calcd. for Chemical Formula: $[C_{30}H_{51}O_9Si]^+[M+H]^+$:582.3224, Found: 582.3215.

The synthesis of **8**

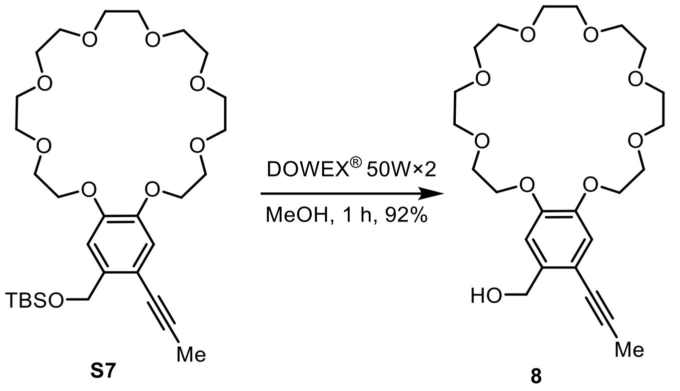

To a solution of **S7** (50 mg, 0.086 mmol) in 1 mL MeOH was added DOWEX®50W×2 (100 mg, 200-300 mesh). The solution was stirred at room temperature for 1 h. After filtration, the reaction solvent was evaporated under reduced pressure. The residue was diluted with 10 mL ethyl acetate and washed with saturated NaCl. The combined organic layers were dried with Na_2SO_4 and concentrated *in vacuo* to afford the crude product. Chromatography purification on silica gel using chloroform/methanol (20:1) as the eluents gave the purified product **8** (37 mg, 92%) as a colorless oil.

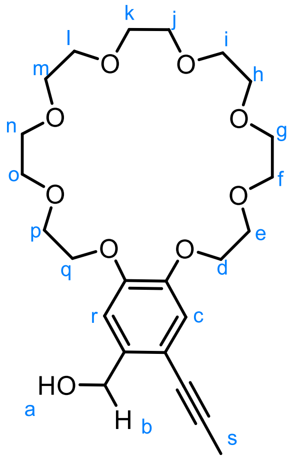

R_f = 0.24 (chloroform/methanol = 10: 1).

^{1}H NMR (500 MHz, CD_2Cl_2) δ 7.00 (s, 1H, H_c), 6.95 (s, 1H, H_r), 4.71 (s, 2H, H_b), 4.21 – 4.18 (m, 2H, H_q), 4.16 – 4.13 (m, 2H, H_d), 3.90 – 3.86 (m, 4H, H_p and H_e), 3.74 (dt, *J* = 5.3, 3.3 Hz, 4H, $H_{f\text{-}o}$), 3.68 (dt, *J* = 4.2, 2.0 Hz, 4H, $H_{f\text{-}o}$), 3.61– 3.60 (m, 9H, $H_{f\text{-}o}$), 3.59– 3.58 (m, 3H, $H_{f\text{-}o}$), 2.09 (s, 3H, H_s).

^{13}C NMR (126 MHz, CD_2Cl_2) δ 149.5, 148.3, 137.3, 124.3, 118.4, 115.3, 114.1, 113.2, 89.6, 77.5, 71.4, 71.19, 71.16, 71.1, 70.2, 69.9, 69.7, 64.0, 30.3, 4.6.

Note: The observed overlap of ^{13}C NMR signals stacking in the 69.7–71.4 ppm region is the glycol groups in crown ether. 14 non-equivalent carbons in theory, 7 signals recorded.

HRMS (m/z, APCI): Calcd. for Chemical Formula: $[C_{25}H_{37}O_8]^+$$[M+H]^+$:469.2432, Found: 469.2426.

Preparation of cavitands loaded with crown ether

The synthesis of **9**

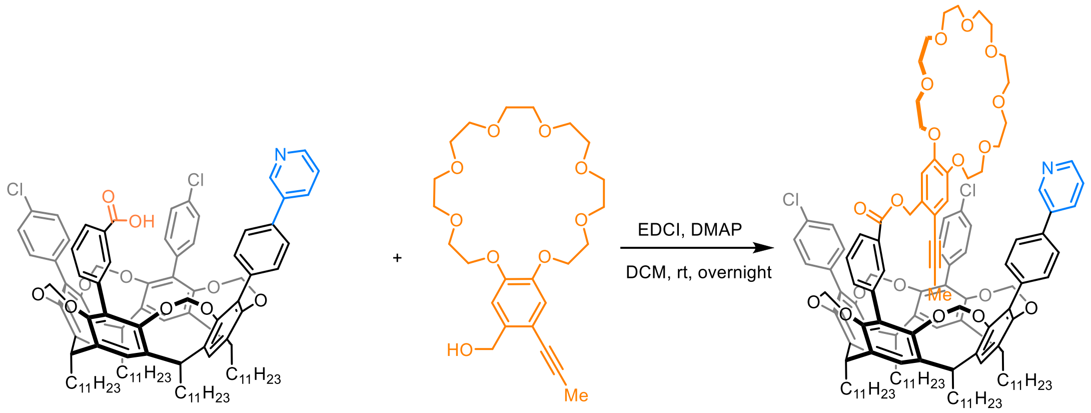

3 **8** **9**

To a solution of **3** (40 mg, 0.024 mmol) in 1 mL dry DCM were added DMAP (9 mg, 0.072 mmol, 3 equiv.) and EDCI (14 mg, 0.072 mmol, 3 equiv.). The solution was stirred at room temperature for 5 min and then alcohol **8** (13 mg in 0.5 mL dry DCM, 0.029 mmol, 1.2 equiv.) was added. The reaction mixture was stirred at room temperature overnight. The solution was diluted with 20 mL DCM and washed with 10 mL saturated NaH_2PO_4 and 10 mL saturated NaCl. The combined organic layers were dried with Na_2SO_4 and concentrated *in vacuo* to afford the crude product. Chromatography purification on silica gel using chloroform/methanol (20:1) as the eluents gave the purified product **9** (42 mg, 82 % yield) as a light-yellow oil.

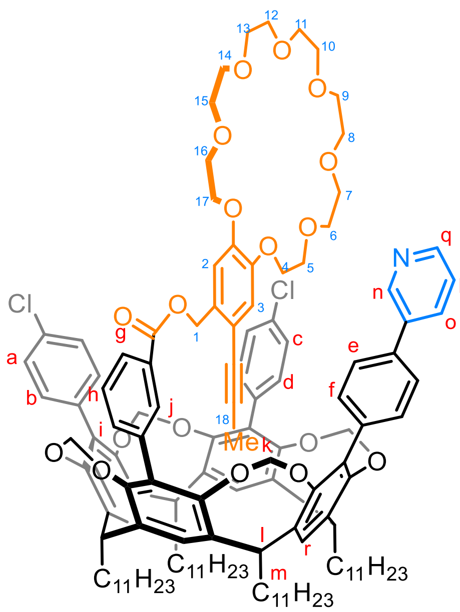

R_f = 0.5 (100% ethyl acetate).

^{1}H NMR (500 MHz, $CDCl_3$) δ 8.83 (s, 1H, H_n), 8.62 (s, 1H, H_q), 7.97 – 7.83 (m, 2H, H_g and H_i), 7.51 (s, 1H, H_j), 7.48 – 7.45 (m, 2H), 7.44 – 7.36 (m, 4H, H_r), 7.33 (d, *J* = 2.7 Hz, 2H), 7.31 (d, *J* = 3.8 Hz, 2H), 7.16 (d, *J* = 8.0 Hz, 2H, H_a), 7.10 (d, *J* = 7.1 Hz, 2H), 7.07 – 7.00 (m, 5H, H_f), 6.93 (d, *J* = 7.7 Hz, 2H, H_b), 6.51 (s, 1H, H_3), 5.43 – 5.34 (m, 2H, H_1), 5.14 (dd, *J* = 14.9, 7.0 Hz, 2H, H_k), 4.99 – 4.93 (m, 2H, H_k), 4.93-4.84 (m, 4H, H_l), 4.25 (d, *J* = 7.0 Hz, 2H, H_k), 4.20 (d, *J* = 7.0 Hz, 1H, H_k), 4.17 – 4.12 (m, 1H, H_k), 4.08 (d, *J* = 7.0 Hz, 1H, H_k), 3.97 (t, *J* = 4.1 Hz, 2H, H_{17}), 3.83 – 3.79 (m, 2H, H_4), 3.75 – 3.72 (m, 2H, $H_{5\text{-}16}$), 3.72 – 3.68 (m, 5H, $H_{5\text{-}16}$), 3.68 – 3.62 (m, 11H, $H_{5\text{-}16}$), 3.61 – 3.59 (m, 3H, $H_{5\text{-}16}$), 2.39 – 2.32 (m, 8H, H_m, 4×CH_2), 1.52 – 1.44 (m, 16H, H_m, 8×CH_2), 1.29 (s, 56H, H_m, 28×CH_2), 0.91 – 0.87 (m, 12H, H_m, 4×CH_3), -1.05 (s, 3H, H_{18}).

Note: 26 H (6.51 – 8.83 ppm) for aryl groups in compound **9**; $H_{c,d}$, H_e, H_i, H_r and H_h resonate at 7.48 – 7.45 and 7.33 – 7.10 ppm, but their signals could not be clearly distinguished.

^{13}C NMR (126 MHz, $CDCl_3$) δ 165.3, 152.8, 152.7$_3$, 152.7$_2$, 152.6, 152.4, 152.3, 149.0, 148.3, 147.7, 139.0, 138.7, 138.6$_7$, 138.6$_2$, 134.6, 134.3, 133.8, 133.3, 133.1, 132.8$_2$, 132.7$_8$, 131.4, 130.9, 130.8, 130.1, 129.8, 129.7, 129.6, 129.1, 128.5, 128.4, 128.3, 126.9, 120.2$_3$, 120.2$_0$, 120.1, 119.9, 99.9, 99.8$_3$, 99.8$_1$, 99.7, 88.9, 79.3, 71.0, 70.9, 70.8, 70.7$_5$, 70.7$_3$, 70.6$_8$, 70.6$_2$, 70.5$_6$, 69.2, 37.1, 36.9, 32.0, 30.2$_1$, 30.1$_8$,

30.0, 29.9, 29.8, 29.4, 28.0, 22.7, 14.1.

Note*:* The observed overlap of ^{13}C NMR signals in the 119.9–152.8 ppm region is likely a result of the symmetry presents in the cavitand scaffold. 53 non-equivalent aromatic carbons in theory, 36 signals recorded; The observed overlap of ^{13}C NMR signals in the 14.1–37.1 ppm region is likely a result of the symmetry present in the cavitand scaffold. 44 non-equivalent carbons for the $C_{11}H_{23}$ groups in theory, 12 signals recorded.

HRMS (m/z, APCI): Calcd. for Chemical Formula: $[C_{130}H_{164}{}^{35}Cl_2N_2O_{18}]^+[M+H]^+$: 2097.1247., Found: 2097.1239.

The synthesis of **10**

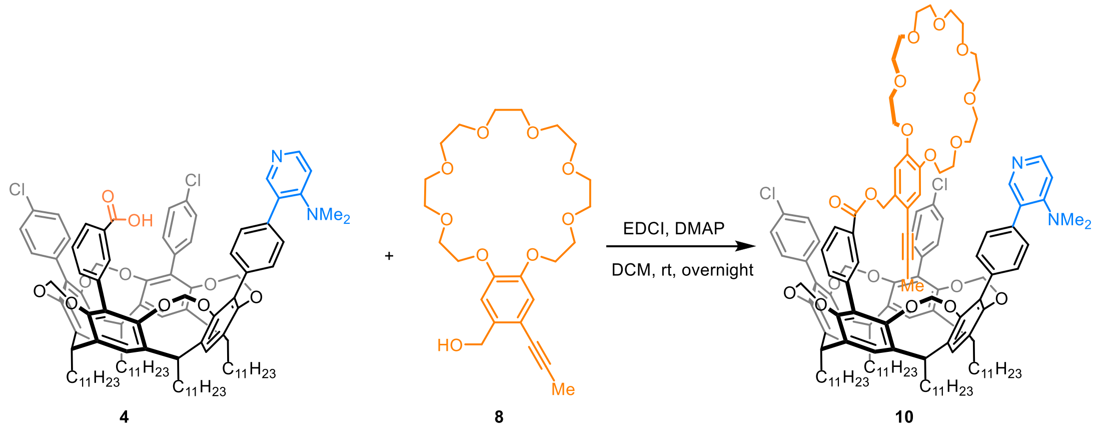

To a solution of **4** (30 mg, 0.018 mmol) in 1 mL dry DCM were added DMAP (7 mg, 0.054 mmol, 3 equiv.) and EDCI (10 mg, 0.054 mmol, 3 equiv.). The solution was stirred at room temperature for 5 min and then alcohol **8** (10 mg in 0.5 mL dry DCM, 0.021 mmol, 1.2 equiv.) was added. The reaction mixture was stirred at room temperature overnight. The solution was diluted with 20 mL DCM and washed with 10 mL saturated NaH_2PO_4 and 10 mL saturated NaCl. The combined organic layers were dried with Na_2SO_4 and concentrated *in vacuo* to afford the crude product. Chromatography purification on silica gel using chloroform/methanol (20:1) as the eluents gave the purified product **10** (25 mg, 65% yield) as a colorless oil.

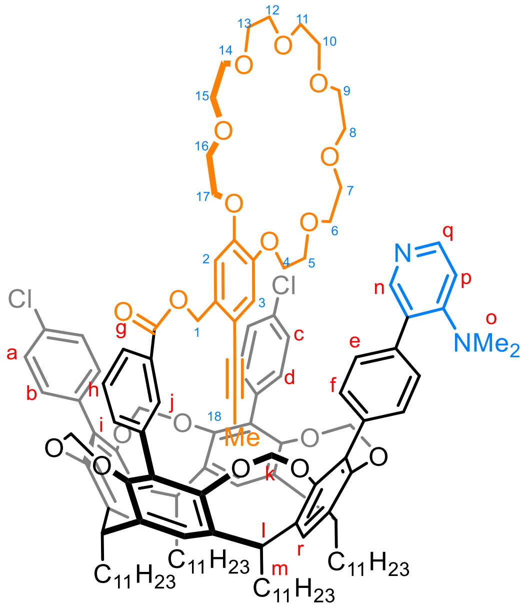

R$_f$ = 0.3 (chloroform/methanol = 10: 1).

^{1}H NMR (400 MHz, CDCl$_3$) δ 8.25 (d, *J* = 6.0 Hz, 1H, H$_q$), 8.15 (s, 1H, H$_n$), 7.94 – 7.91 (m, 1H, H$_h$), 7.51 – 7.45 (m, 3H, H$_j$, H$_g$ and H$_i$), 7.35 – 7.30 (m, 7H, H$_r$), 7.12 – 7.09 (m, 2H, H$_a$), 7.08 – 7.03 (m, 6H), 6.91 (d, *J* = 7.9 Hz, 2H, H$_b$), 6.79 (d, *J* = 6.0 Hz, 1H, H$_p$), 6.51 (s, 1H, H$_3$), 5.41 (d, *J* = 12.0 Hz, 1H, H$_1$), 5.27 – 5.23 (m, 1H, H$_1$), 5.18 – 5.12 (m, 2H, H$_k$), 4.93 – 4.89 (m, 2H, H$_k$), 4.88 – 4.82 (m, 4H, H$_l$), 4.27 – 4.20 (dd, *J* = 9.5, 5.5 Hz, 4H, H$_k$), 4.16 – 4.12 (m, 2H, H$_k$), 3.96 – 3.92 (m, 2H, H$_{17}$), 3.91 – 3.86 (m, 2H, H$_4$), 3.80 (d, *J* = 5.3 Hz, 2H, H$_{5\text{-}16}$), 3.69 (s, 6H, H$_{5\text{-}16}$), 3.66 (s, 14H, H$_{5\text{-}16}$), 2.64 (s, 6H, H$_o$), 2.39 – 2.33 (m, 8H, H$_m$, 4×CH$_2$), 1.53-1.41 (m, 16H, H$_m$, 8×CH$_2$), 1.40-1.21 (m, 56H, H$_m$, 28×CH$_2$), 0.90 (d, *J* = 6.5 Hz, 12H, H$_m$, 4×CH$_3$), -1.03 (s, 3H, H$_{18}$).

Note: 25 H (6.51 – 8.25 ppm) for aryl groups in compound **10**; H$_{c\text{-}f}$, H$_p$ and H$_h$ resonate at 7.35 –7.30 and 7.08 – 7.03 ppm, but their signals could not be clearly distinguished.

^{13}C NMR (126 MHz, CDCl$_3$) δ 165.3, 156.4, 152.8, 152.7$_3$, 152.7$_0$, 152.6$_5$, 152.5$_9$, 152.3, 152.2, 149.1, 148.6, 139.0, 138.9, 138.8, 138.6, 138.5, 138.4, 138.3, 134.7, 133.8, 133.5, 133.2, 132.8, 132.7, 131.4, 131.1, 130.9, 130.7, 130.3, 129.8, 129.7, 129.6, 129.0, 128.5, 128.4, 128.2, 124.9, 120.2, 120.0, 119.9, 119.5, 118.3, 116.9, 116.4, 115.1, 110.4, 100.6, 100.0, 99.9, 99.6, 88.8, 79.3, 71.2, 71.0, 70.9, 70.8$_5$,

70.76, 69.7, 69.6, 69.5, 69.4, 42.1, 37.1, 30.4, 30.3, 30.2, 30.0, 29.9, 29.8, 29.4, 28.0, 22.7, 14.1.

Note*:* The observed overlap of ^{13}C NMR signals in the 110.4–156.4 ppm region is likely a result of the symmetry presents in the cavitand scaffold. 53 non-equivalent aromatic carbons in theory, 45 signals recorded; The observed overlap of ^{13}C NMR signals in the 14.1–37.1 ppm region is likely a result of the symmetry present in the cavitand scaffold. 44 non-equivalent carbons for the $C_{11}H_{23}$ groups in theory, 10 signals recorded.

HRMS (m/z, APCI): Calcd. for Chemical Formula: $[C_{132}H_{169}{}^{35}Cl_2N_2O_{18}]^+[M+H]^+$: 2140.1669, Found: 2140.1677.

The synthesis of **11**

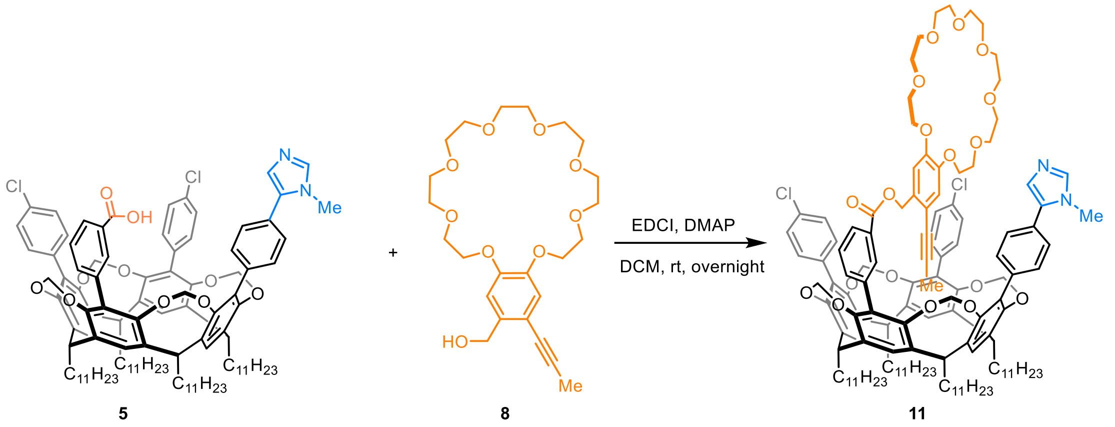

To a solution of **5** (18 mg, 0.011 mmol) in 1 mL dry DCM were added DMAP (4 mg, 0.033 mmol, 3 equiv.) and EDCI (6 mg, 0.033 mmol, 3 equiv.). The solution was stirred at room temperature for 5 min and then alcohol **8** (6 mg in 0.5 mL dry DCM, 0.013 mmol, 1.2 equiv.) was added. The reaction mixture was stirred at room temperature overnight. The solution was diluted with 20 mL DCM and washed with 10 mL saturated NaH_2PO_4 and 10 mL saturated NaCl. The combined organic layers were dried with Na_2SO_4 and concentrated *in vacuo* to afford the crude product. Chromatography purification on silica gel using chloroform/methanol (20:1) as the eluents gave the purified product **11** (12 mg, 52 % yield) as a light-yellow oil.

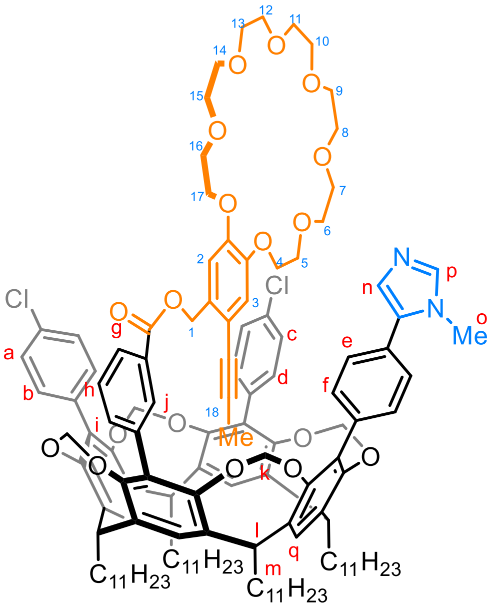

$\mathbf{R_f}$ = 0.4 (chloroform/methanol = 10:1).

^{1}H NMR (500 MHz, $CDCl_3$) δ 8.16 – 8.10 (br, 1H, H_p), 7.95 – 7.90 (m, 1H, H_h), 7.49 – 7.44 (m, 3H, H_j, H_g and H_i), 7.35 – 7.29 (m, 5H, H_q), 7.24 – 7.16 (br, 3H), 7.15 – 7.12 (br, 2H, H_a), 7.08 – 7.00 (m, 6H, H_c and H_d), 6.94 (d, *J* = 7.8 Hz, 2H, H_b), 6.47 (s, 1H, H_3), 5.13 (dd, *J* = 16.7, 6.8 Hz, 2H, H_2), 4.95 (d, *J* = 6.8 Hz, 1H, H_k), 4.90 – 4.83 (m, 5H, H_l), 4.28 (d, *J* = 6.9 Hz, 1H, H_k), 4.26 – 4.21 (m, 2H, H_k), 4.20 – 4.14 (m, 2H, H_k), 4.05 – 4.00 (m, 1H, H_k), 3.98 – 3.87 (m, 3H, H_{17} and H_4), 3.80 – 3.74 (m, 4H, $H_{5\text{-}16}$), 3.73 – 3.65 (m, 14H, $H_{5\text{-}16}$), 3.64 – 3.59 (m, 9H, $H_{5\text{-}16}$), 2.36 (s, 8H, H_m, 4×CH_2), 1.53 – 1.43 (m, 16H, H_m, 8×CH_2), 1.29 (s, 56H, H_m, 28×CH_2), 0.89 (t, *J* = 6.1 Hz, 12H, H_m, 4×CH_3), -1.10 (s, 3H, H_{18}).

Note: 24 H (6.47 – 8.16 ppm) for aryl groups in compound **11**; $H_{c,d}$, H_a and H_n resonate at 7.24 –7.16 and 7.08 – 7.03 ppm, but their signals could not be clearly distinguished.

^{13}C NMR (126 MHz, $CDCl_3$) δ 165.2, 152.7_2, 152.6_8, 152.6_6, 152.3, 139.1_2, 139.0_8, 139.0, 138.9, 138.7, 138.6_3, 138.6_0, 138.5, 134.6, 133.8, 133.4, 133.1, 132.8, 132.7, 131.2, 130.9, 130.8, 130.7, 130.0, 129.8, 129.6, 129.1, 128.5, 128.4, 128.2, 120.3_2, 120.2_9, 120.2, 119.9, 99.9, 99.8, 99.7, 99.6, 70.8_4, 70.7_5, 70.7, 70.6, 70.3, 69.6, 69.4, 65.1, 37.1, 37.0, 36.9, 32.0, 30.2, 30.0, 29.9, 29.8_0, 29.7_6, 29.4, 28.0, 22.7, 14.1, 1.0.

Note*:* The observed overlap of ^{13}C NMR signals in the 119.9–152.72 ppm region is likely a result of the symmetry presents in the cavitand scaffold. 51 non-equivalent aromatic carbons in theory, 33 signals recorded; The observed overlap of ^{13}C NMR signals in the 14.1–37.1 ppm region is likely a result of the symmetry present in the cavitand scaffold. 44 non-equivalent carbons for the $C_{11}H_{23}$ groups in theory, 13 signals recorded.

HRMS (m/z, APCI): Calcd. for Chemical Formula: $[C_{129}H_{165}{}^{35}Cl_2N_2O_{18}]^+[M+H]^+$: 2100.1429, Found: 2100.1424.

The synthesis of **12**

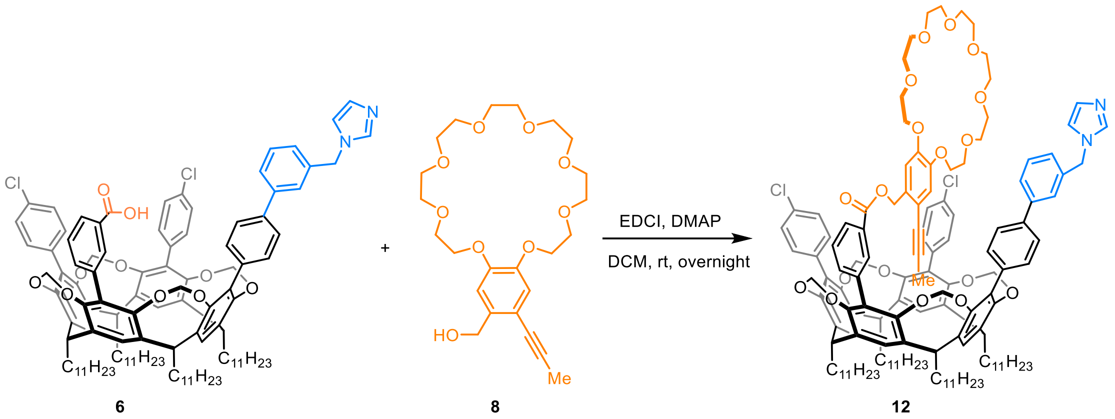

To a solution of **6** (22 mg, 0.013 mmol) in 1 mL dry DCM were added DMAP (5 mg, 0.039 mmol, 3 equiv.) and EDCI (10 mg, 0.039 mmol, 3 equiv.). The solution was stirred at room temperature for 5 min and then alcohol **8** (9 mg in 0.5 mL dry DCM, 0.02 mmol, 1.5 equiv.) was added. The reaction mixture was stirred at room temperature overnight. The solution was diluted with 20 mL DCM and washed with 10 mL saturated NaH_2PO_4 and 10 mL saturated NaCl. The combined organic layers were dried with Na_2SO_4 and concentrated *in vacuo* to afford the crude product. Chromatography purification on silica gel using chloroform/methanol (20:1) as the eluents gave the purified product **12** (17 mg, 60% yield) as a colorless oil.

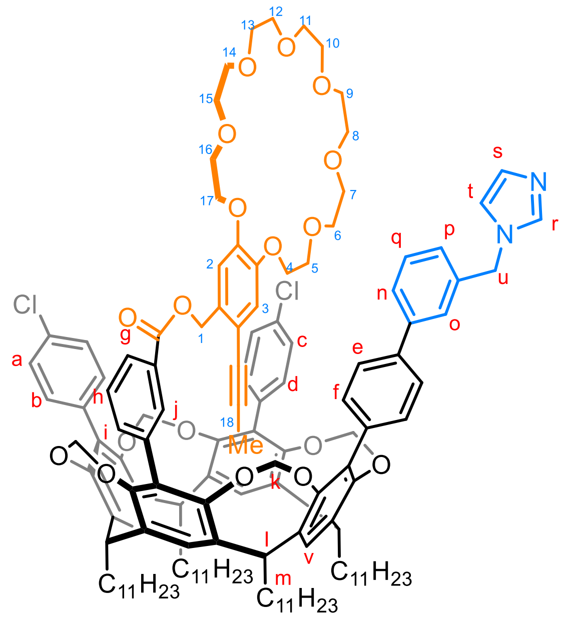

R$_f$ = 0.3 (chloroform/methanol = 10: 1).

^{1}H NMR (500 MHz, CDCl$_3$) δ 8.29 – 8.00 (br, 1H, H$_r$), 7.94 – 7.88 (m, 1H, H$_h$), 7.54 – 7.50 (m, 2H, H$_g$ and H$_i$), 7.49 – 7.35 (m, 6H, H$_{n\text{-}p}$), 7.33 (d, *J* = 2.2 Hz, 3H, H$_f$), 7.29 – 7.33 (m, 3H, H$_v$), 7.22 – 7.15 (br, 2H, H$_b$), 7.10 – 7.00 (m, 8H), 6.97 – 6.91 (m, 2H, H$_a$), 6.58-6.47 (br, 1H, H$_3$), 5.35 – 5.15 (m, 4H, H$_1$ and H$_h$), 5.15-5.07 (m, 2H, H$_k$), 4.93 – 4.80 (m, 6H, H$_k$ and H$_l$), 4.30 – 4.21 (br, 3H, H$_k$), 4.21 – 4.07 (m, 3H, H$_k$), 3.95 – 3.84 (m, 2H, H$_{5\text{-}16}$), 3.78 – 3.56 (m, 25H, H$_{5\text{-}16}$), 2.36 (s, 8H, H$_m$, 4×CH$_2$), 1.48 (s, 16H, H$_m$, 8×CH$_2$), 1.29 (s, 56H, H$_m$, 28×CH$_2$), 0.91 – 0.87 (m, 12H, H$_m$, 4×CH$_3$), -1.11 (s, 3H, H$_{18}$).

Note: 29 H (6.47 – 8.29 ppm) for aryl groups in compound **12**; H$_{c,d}$, H$_b$ and H$_v$ resonate at 7.49 –7.35 and 7.10 – 7.00 ppm, but their signals could not be clearly distinguished.

^{13}C NMR (126 MHz, CDCl$_3$) δ 165.2, 152.8, 152.7, 152.6$_0$, 152.5$_5$, 152.4, 152.3, 139.1, 139.0, 138.7, 138.6, 138.5, 138.4, 138.3, 134.7, 133.8, 133.2, 133.1, 132.9, 132.8, 132.5, 131.3, 131.2, 130.8$_2$, 130.7$_7$, 130.7, 130.4, 129.7$_3$, 129.6$_9$, 128.5, 128.3, 128.2, 126.8, 126.7, 120.2, 118.5, 115.2, 100.5, 99.7, 79.4, 72.3, 71.2, 71.1, 70.9, 70.7, 69.8$_5$, 69.7$_5$, 69.7, 69.6, 66.5, 64.8, 37.1, 32.0, 30.4, 30.2, 29.9, 29.8, 29.4, 28.0, 22.7, 14.1.

Note*:* The observed overlap of ^{13}C NMR signals in the 115.2–152.8 ppm region is likely a result of the

symmetry presents in the cavitand scaffold. 57 non-equivalent aromatic carbons in theory, 36 signals recorded; The observed overlap of ^{13}C NMR signals in the 14.1–37.1 ppm region is likely a result of the symmetry present in the cavitand scaffold. 44 non-equivalent carbons for the $C_{11}H_{23}$ groups in theory, 10 signals recorded.

HRMS (m/z, APCI): Calcd. for Chemical Formula: $[C_{135}H_{169}{}^{35}Cl_2N_2O_{18}]^+[M+H]^+$: 2176.1742, Found: 2176.1746.

The synthesis of **13**

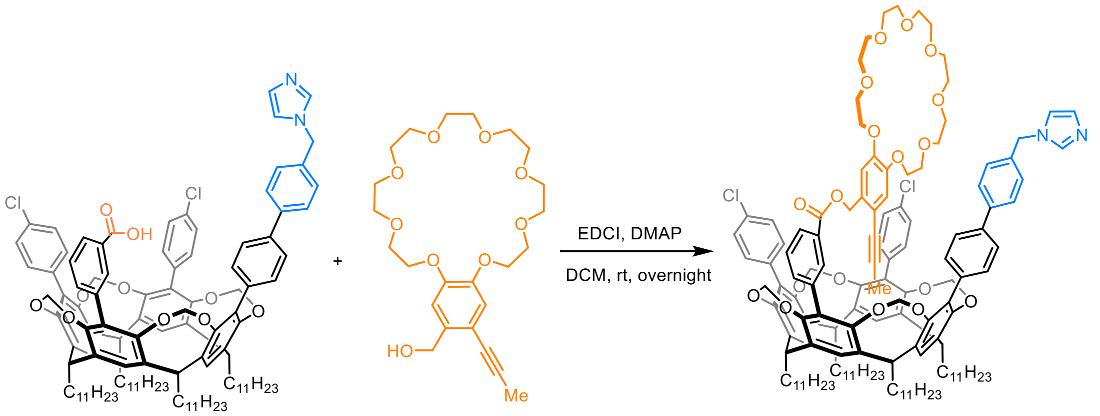

To a solution of **7** (31 mg, 0.018 mmol) in 1 mL dry DCM were added DMAP (7 mg, 0.054 mmol, 3 equiv.) and EDCI (10 mg, 0.054 mmol, 3 equiv.). The solution was stirred at room temperature for 5 min and then alcohol **8** (10 mg in 0.5 mL dry DCM, 0.021 mmol, 1.2 equiv.) was added. The reaction mixture was stirred at room temperature overnight. The solution was diluted with 20 mL DCM and washed with 10 mL saturated NaH_2PO_4 and 10 mL saturated NaCl. The combined organic layers were dried with Na_2SO_4 and concentrated *in vacuo* to afford the crude product. Chromatography purification on silica gel using chloroform/methanol (20:1) as the eluents gave the purified product **13** (29 mg, 74% yield) as a colorless oil.

$\mathbf{R_f}$ = 0.3 (chloroform/methanol = 10: 1).

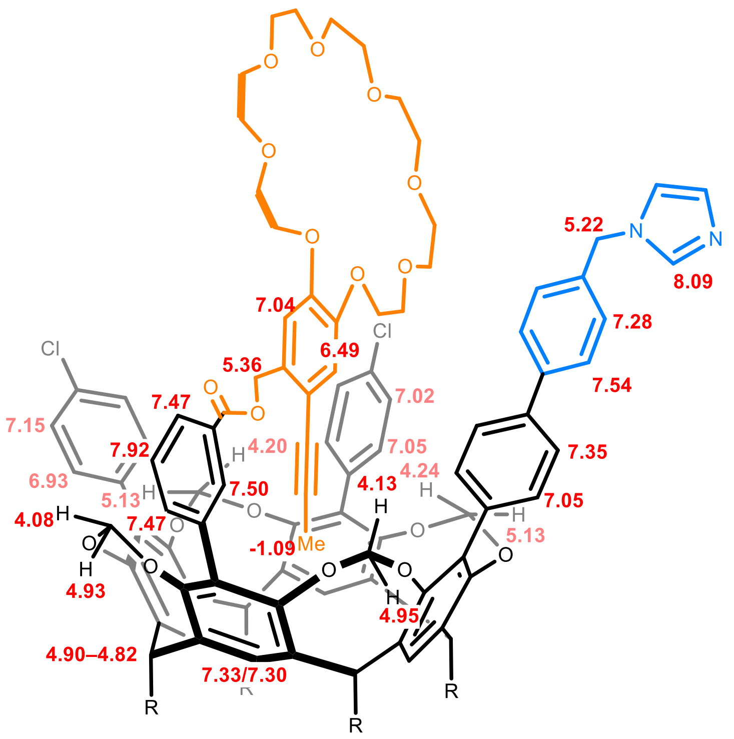

13

^{1}H NMR chemical shifts are shown in red color (δ ppm).

^{1}H NMR (500 MHz, $CDCl_3$) δ 8.09 (s, 1H), 7.92 (tt, *J* = 5.6, 4.1 Hz, 1H), 7.54 (d, *J* = 8.3 Hz, 2H), 7.50 (q, *J* = 1.3 Hz, 1H), 7.49 – 7.45 (m, 2H), 7.37 – 7.34 (m, 2H), 7.34 – 7.30 (m, 4H), 7.28 (d, *J* = 8.1 Hz, 2H), 7.20 (s, 1H), 7.15 (d, *J* = 8.2 Hz, 2H), 7.08 – 6.99 (m, 8H), 6.93 (d, *J* = 7.9 Hz, 2H), 6.49 (s, 1H), 5.42 – 5.30 (m, 2H), 5.22 (s, 2H), 5.13 (t, *J* = 7.2 Hz, 2H), 4.95 (d, *J* = 4.9 Hz, 1H), 4.93 (d, *J* = 4.7 Hz, 1H), 4.90 – 4.82 (m, 4H), 4.27 – 4.22 (m, 3H), 4.20 (d, *J* = 7.1 Hz, 1H), 4.13 (d, *J* = 7.4 Hz, 1H), 4.08 (d, *J* = 7.2 Hz, 1H), 3.95 (dt, *J* = 4.3, 2.7 Hz, 2H), 3.81 (td, *J* = 4.3, 2.1 Hz, 2H), 3.74 (t, *J* = 4.7 Hz, 2H), 3.72 – 3.67 (m, 4H), 3.67 – 3.61 (m, 12H), 3.60 – 3.56 (m, 4H), 2.43 – 2.28 (m, 8H), 1.55 – 1.41 (m, 16H), 1.40 – 1.26 (m, 56H), 0.92 – 0.86 (m, 12H), -1.09 (s, 3H).

^{13}C NMR (126 MHz, $CDCl_3$) δ 165.39, 152.92, 152.87, 152.84, 152.81, 152.76, 152.75, 152.48, 152.37, 149.03, 139.14, 139.08, 138.99, 138.80, 138.75, 138.69, 138.66, 138.62, 134.77, 133.86, 133.40, 133.22, 132.93, 132.87, 131.47, 130.90, 130.44, 129.94, 129.81, 129.73, 128.61, 128.52, 128.51, 128.48, 127.96, 127.02, 120.34, 120.25, 120.23, 120.11, 120.04, 119.35, 116.63, 115.92, 115.72,99.99, 99.96,

99.77, 89.09, 79.47, 77.36, 71.29, 70.96, 70.87, 70.80, 69.81, 69.73, 69.40, 69.08, 65.20, 51.61, 37.17, 37.04, 32.10, 30.33, 30.15, 30.04, 30.02, 29.93, 29.89, 29.88, 29.57, 28.12, 22.85, 14.28.

HRMS (m/z, APCI): Calcd. for Chemical Formula: $[C_{135}H_{169}{}^{35}Cl_2N_2O_{18}]^+$$[M+H]^+$: 2176.1742, Found: 2176.1746.

Synthesis of rotaxane in toluene

Rotaxane formation through sulfonylation of amine

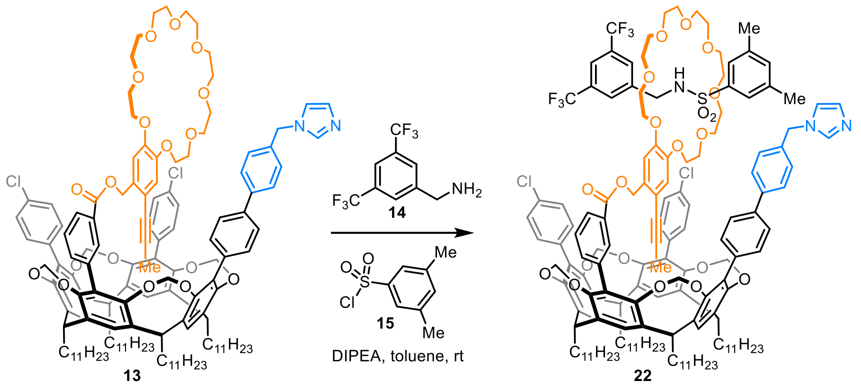

Under N_2 atmosphere in the glovebox, a 10 mL round bottom flask equipped with a magnetic stirring bar were added cavitand **13** (0.004 mmol), 3,5-bis(trifluoromethyl)benzylamine **14** (0.012 mmol, 3 equiv., 2.8 mg) and DIPEA (0.016 mmol, 4 equiv., 2 mg) in 0.3 mL toluene. 3,5-dimethylbenzenesulfonyl chloride **15** (0.012 mmol, 3 equiv., 2.4 mg) in 0.2 mL toluene was added into the reaction mixture at room temperature. The reaction mixture was stirred at room temperature for 24 hours. The solution was diluted with 20 mL ethyl acetate and washed with 10 mL saturated NaH_2PO_4 and saturated NaCl. The combined organic layers were dried with Na_2SO_4 and concentrated *in vacuo* to afford the crude product. Chromatography purification on silica gel using hexane/ethyl acetate = 1:2 to 1:3 as the eluents gave the products **22**.

R_f = 0.4 (hexane/ethyl acetate = 1:3).

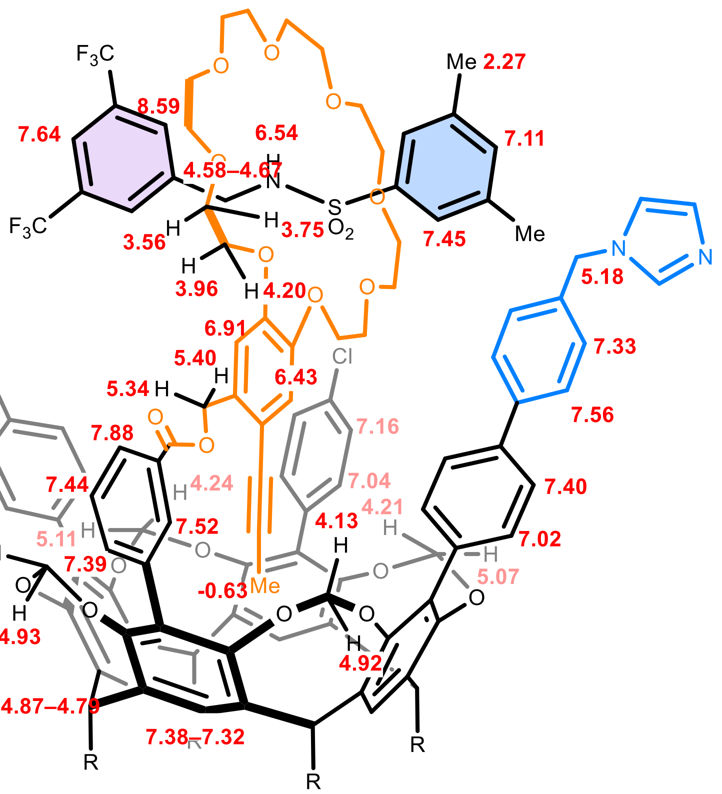

22

^{1}H NMR chemical shifts are shown in red color (δ ppm).

^{1}H NMR (500 MHz, CD_2Cl_2) δ 8.59 (s, 2H), 8.03 (s, 1H), 7.88 (d, *J* = 7.6 Hz, 1H), 7.64 (s, 1H), 7.56 (d, *J* = 7.9 Hz, 2H), 7.52 (s, 1H), 7.45 (s, 2H), 7.44 (s, 1H), 7.40 (d, *J* = 8.4 Hz, 3H), 7.38 – 7.31 (m, 7H), 7.18 (d, *J* = 9.2 Hz, 2H), 7.16 (s, *J* = 9.5 Hz, 2H), 7.11 (s, 1H), 7.05 (s, 1H), 7.02 (d, *J* = 7.9 Hz, 4H), 6.92 (d, *J* = 8.0 Hz, 2H), 6.91 (s, 1H), 6.54 (t, *J* = 5.7 Hz, 1H), 6.43 (s, 1H), 5.40 (d, *J* = 12.0 Hz, 1H), 5.34 (d, *J* = 11.9 Hz, 1H), 5.18 (d, *J* = 8.6 Hz, 2H), 5.11 (d, *J* = 7.0 Hz, 1H), 5.07 (d, *J* = 7.1 Hz, 1H), 4.93 (d, *J* = 7.5 Hz, 1H), 4.92 (d, *J* = 7.4 Hz, 1H), 4.87 – 4.78 (m, 4H), 4.52 (qd, *J* = 13.8, 5.3 Hz, 2H), 4.24 (d, *J* = 7.0 Hz, 1H), 4.21 (d, *J* = 7.2 Hz, 1H), 4.20 (m, 1H), 4.13 (d, *J* = 7.4 Hz, 1H), 4.09 (d, *J* = 7.5 Hz, 1H), 3.96 (dd, *J* = 10.8, 5.7 Hz, 1H), 3.75 (dd, *J* = 10.7, 5.5 Hz, 1H), 3.69 (t, *J* = 8.4 Hz, 1H), 3.56 (dd, *J* = 11.1, 6.6 Hz, 1H), 3.52 – 3.46 (m, 1H), 3.39 (dt, *J* = 11.3, 6.4 Hz, 2H), 3.36 – 3.23 (m, 12H), 3.16 (dt, *J* = 11.2, 6.1 Hz, 4H), 3.07 (q, *J* = 10.7 Hz, 2H), 2.96 (d, *J* = 8.9 Hz, 1H), 2.81 – 2.74 (m, 1H), 2.42 – 2.32 (m, 8H), 2.27 (s, 6H), 1.53 – 1.41 (m, 16H), 1.40 – 1.26 (m, 56H), 0.88 (t, *J* = 6.6 Hz, 12H), -0.63 (s, 3H).

^{13}C NMR (126 MHz, CD_2Cl_2) δ 165.09, 152.74, 152.73, 152.68, 152.64, 152.41, 152.32, 148.67, 148.05, 141.48, 140.80, 140.64, 139.08, 138.95, 138.89, 138.84, 138.69, 138.60, 138.56, 138.52, 138.47, 134.71, 133.80, 133.73, 133.60, 133.46, 133.43, 133.39, 133.30, 133.15, 133.07, 132.99, 132.86, 132.83, 131.56, 131.37, 130.90, 130.44, 130.23, 130.21, 130.17, 130.13, 129.96, 129.90, 129.46, 129.34, 128.78, 128.63, 128.42, 128.35, 127.91, 127.58, 127.48, 127.39, 126.80, 125.26, 124.42, 123.05, 121.11, 120.50, 120.45, 120.22, 119.23, 116.29, 115.06, 114.43, 100.00, 99.91, 99.87, 79.27, 70.88, 70.81, 70.76, 70.69, 70.62, 70.49, 70.46, 70.42, 70.28, 69.87, 69.69, 69.56, 69.44, 68.66, 67.64, 65.27, 51.55, 45.94, 37.21, 37.11, 31.97, 30.23, 30.14, 30.09, 29.94, 29.80, 29.77, 29.75, 29.68, 29.43, 28.01, 22.71, 20.97, 13.89.

Figure S1. Analysis of NMR spectra of **22**.

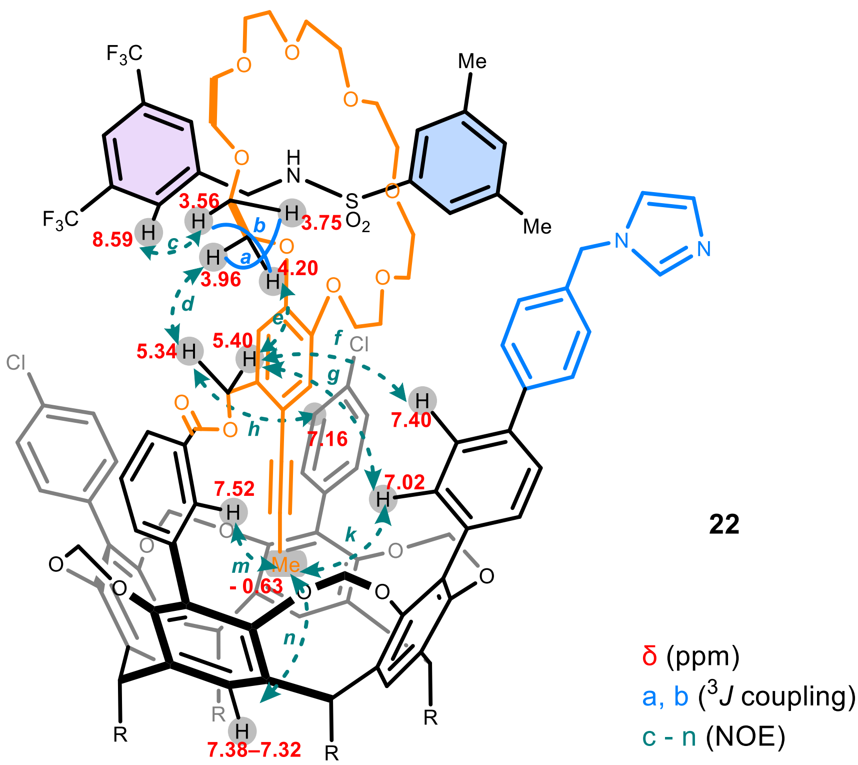

22

δ (ppm)
a, b (^{3}J coupling)
c - n (NOE)

HSQC

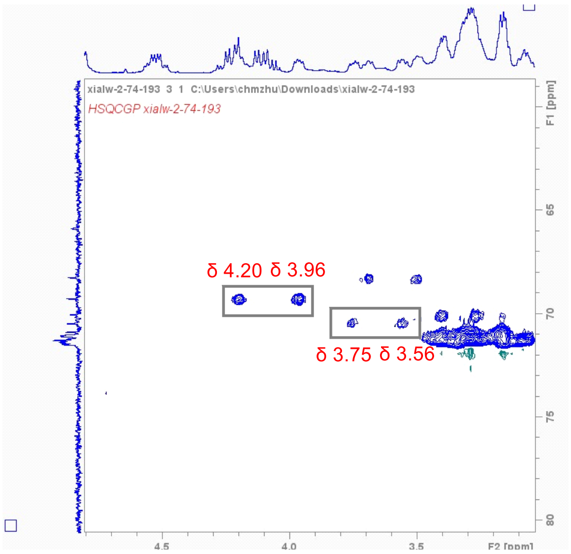

COSY

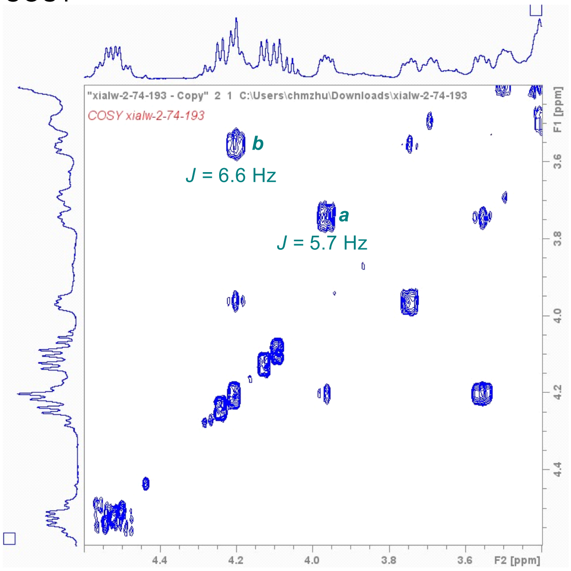

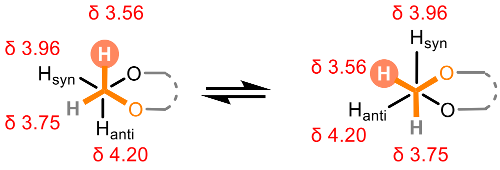

Est. vicinal ^{1}H–^{1}H coupling: J_{anti} = (11.4 + 2.3)/2 = 6.8 Hz (**a** and **b**)
J_{syn} = 2.3 Hz (not observed)

Vicinal ^{1}H–^{1}H coupling in 1,2-dimethoxyethane and polyoxyethylene: J_{trans} = 11.4 Hz and J_{gauche} = 2.3 Hz (*Polym J.* 1985, *17*, 641)

Note:

Figure S1 (left) shows the partial HSQC (^{1}H–^{13}C) spectrum of compound **22** (500 MHz, CD_2Cl_2, 25 °C). Within the crown ether moiety, the **CH_2**O protons adjacent to the phenyl group at δH 3.96 and 4.20 ppm exhibit a strong correlation with δC 68.8 ppm, while the **CH_2**O protons distant from the phenyl group at δH 3.75 and 3.56 ppm correlate with δC 70.0 ppm.

Figure S1 (right) shows the partial COSY (^{1}H–^{1}H) spectrum of compound **22** (500 MHz, CD_2Cl_2, 25 °C). Within the –O–CH_2–CH_2–O– fragment, the coupling constant between vicinal protons δH 3.96 and δH 3.75: J=5.7 Hz (a), and the coupling constant between vicinal protons δH 3.56 and δH 4.20: J=6.6 Hz (b).

Figure S1 (bottom) The vicinal ^{1}H–^{1}H coupling constants depend strongly on the dihedral angle according to the Karplus relationship: a large J_{trans} ≈ 11.4 Hz is expected for the *anti* (180°) orientation, and a small J_{gauche} ≈ 2.3 Hz for the gauche (60°) orientation (reference: *Polym J*. **1985**, 17, 641). Because of rapid conformational averaging of the –O–CH_2–CH_2–O– fragment in solution, the observed coupling of H_{anti} corresponds to the mean value, $J \approx (11.4 + 2.3)/2 = 6.8$ Hz. Therefore, vicinal protons δH 3.96 and δH 3.75 (J=5.7 Hz, a) are designated as *anti*, and vicinal protons δH 3.56 and δH 4.20 (J=6.6 Hz, b)are designated as *anti*. In contrast, the small coupling expected for H_{syn} (≈ 2.3 Hz) is not resolved in the COSY (^{1}H–^{1}H) spectrum.

Figure S1 (continued). Analysis of NMR spectra of **22**.

NOE

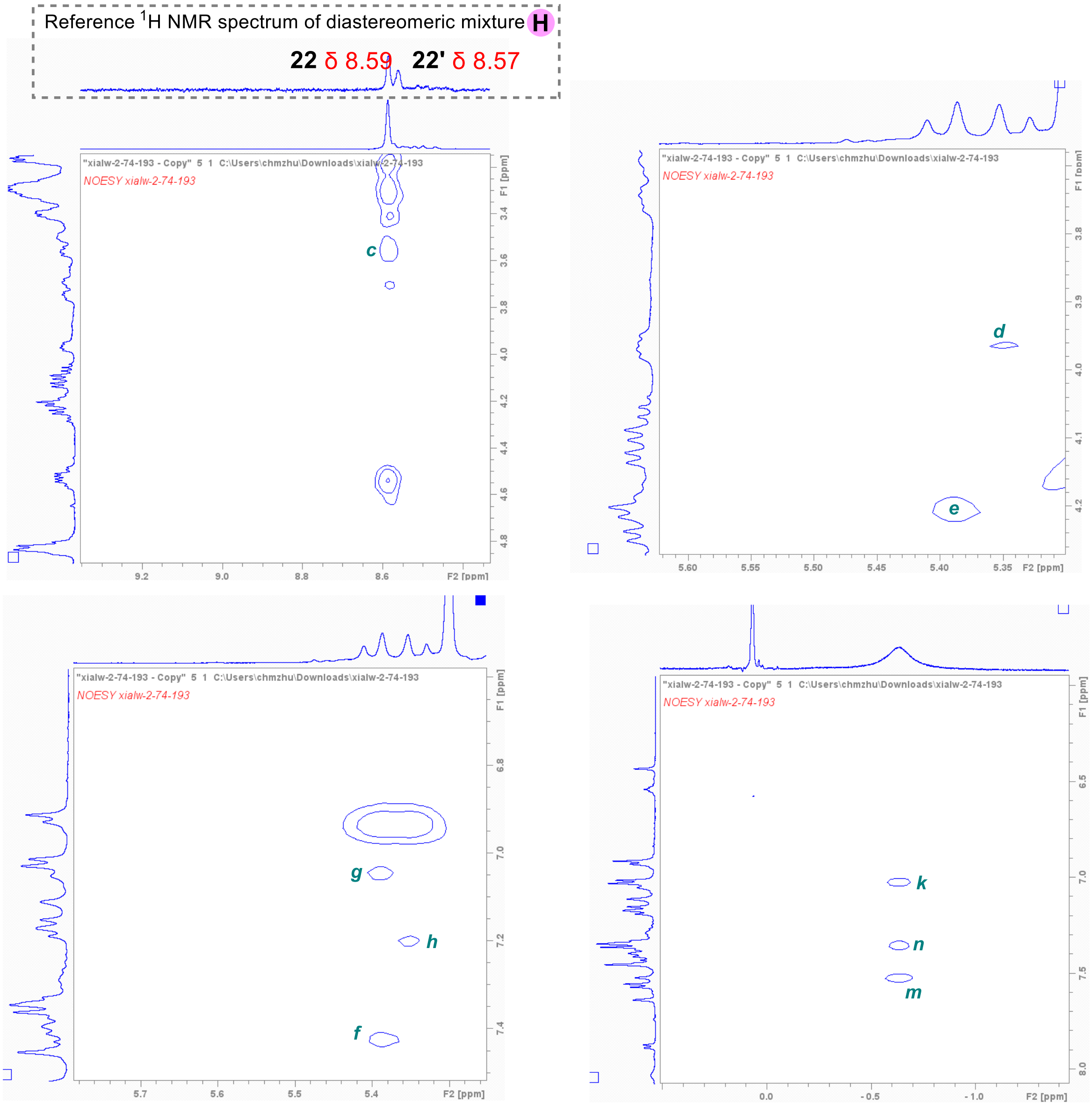

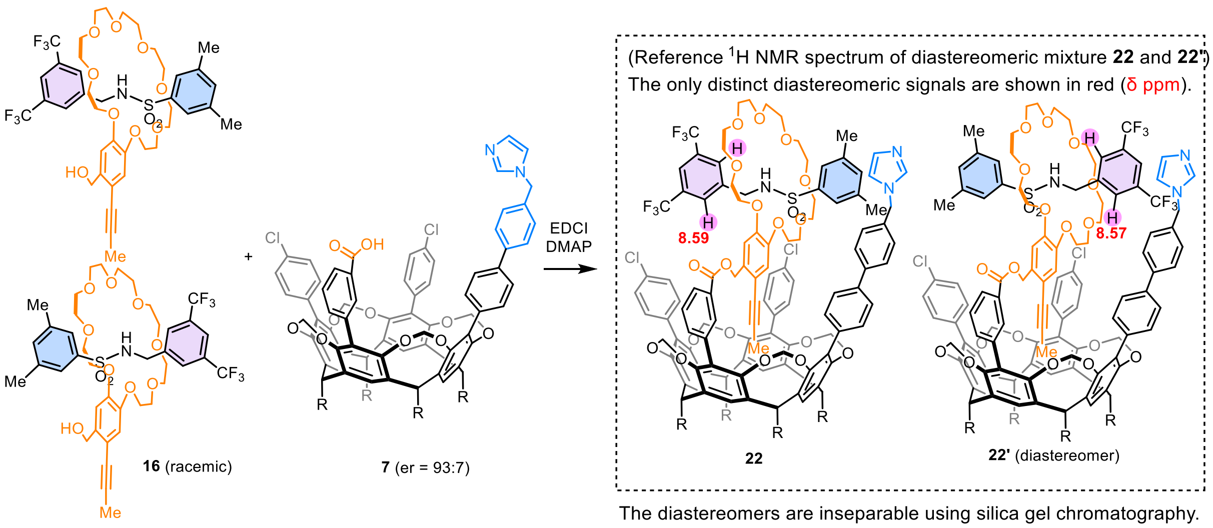

The diastereomers are inseparable using silica gel chromatography.

Note:

Figure S1 (continued) shows the NOESY (^1H–^1H) spectrum of compound **22** (500 MHz, CD_2Cl_2, 25 °C). The spectrum displays the spatial correlations between protons in close proximity, and the correlations are consistent with the predicted relative stereochemistry of the major diastereomer of compound **22** .

(Top left) Protons (δH 8.59 ppm) on 3,5-bis(trifluoromethyl)benzyl group have correlations with proton (CH_2, δH 3.56 ppm) on crown ether (**c**). No correlation was observed between protons (δH 8.59 ppm) on 3,5-bis(trifluoromethyl)benzyl group and proton (CH_2, δH 3.75 ppm) on crown ether. Therefore, the 3,5-bis(trifluoromethyl)benzyl group should be on the same side as proton (CH_2, δH 3.56 ppm) on crown ether, which is on the same side as vicinal proton (δH 3.96) but opposite to vicinal proton (δH 4.20) on crown ether.

(Top right) proton (δH 5.34) on benzyl ester CH_2 has correlations (**d**) with proton (δH 3.96), and proton (δH 5.40) on benzyl ester CH_2 has correlations (**e**) with proton (δH 4.20). Therefore, proton (δH 5.34) on benzyl ester CH_2 and proton (δH 3.96) are on the same side with respect of the crown ether, while proton (δH 5.40) on benzyl ester CH_2 and proton (δH 4.20) are on the same side.

(Middle left) proton (δH 5.34) on benzyl ester CH_2 has correlations (**h**) with proton (δH 7.16, the 4-chlorophenyl group), and proton (δH 5.40) on benzyl ester CH_2 has correlations (**f, g**) with protons (δH 7.40 and 7.02, the phenylene group linked to the [(imidazolyl)methyl]phenyl group). Therefore, the proton (δH 5.40) on benzyl ester CH_2 is on the same side as the [(imidazolyl)methyl]phenyl group with respect to the crown ether.

(Middle right) terminal methyl group (δH -0.63 ppm) have correlations (**k, m, n**) with protons (δH 7.02, 7.38 and 7.52 ppm) on the cavitand scaffold. Therefore, the crown ether moiety is projected

upwards from the cavitand scaffold.

(Bottom) To clarify the difference between diastereomers **22** and **22'** by ^{1}H NMR, we synthesized a mixture of **22** and **22'** by coupling chiral cavitand **7** (93:7 er) with racemic rotaxane **16** (50:50 er). The only distinct diastereomeric signals observed in the ^{1}H NMR spectrum of mixture of **22** and **22'** are the protons (*δ*H 8.59 and 8.57 ppm) of the 3,5-bis(trifluoromethyl)benzyl group.

Therefore, it is reliable to use the protons (*δ*H 8.59) on 3,5-bis(trifluoromethyl)benzyl group as the starting point for correlation of compound **22**, because the possible NOE single from minor diastereomer **22'** (*δ*H 8.57 ppm) should not overlap as shown in the top-left spectrum.

In conclusion, the 5-bis(trifluoromethyl)benzyl group and the [(imidazolyl)methyl]phenyl group are on the opposite sides with respect to the crown ether moiety. The results are consistent with the predicted stereochemical outcomes based on the mechanistic proposal that the imidazolyl group activates the sulfonyl chloride, therefore the 5-bis(trifluoromethyl)benzyl group approaches the crown ether from the opposite side.

Hydrolysis of **22** and TBS protection to yield **17**

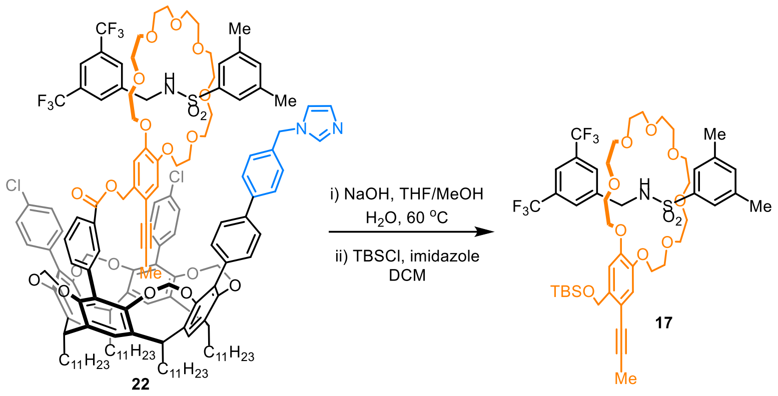

To a solution of **22** in THF/MeOH (2 mL THF and 0.5 mL MeOH) was added 5 M NaOH (0.5 mL, 1 mmol) in water. The reaction mixture was stirred at 60 °C for 2 h. Then the solution was concentrated *in vacuo* to remove solvent. The mixture was dissolved in 20 mL ethyl acetate and washed with 10 mL saturated NaCl. The organic layer was dried with Na_2SO_4 and concentrated *in vacuo* to afford the crude product. The crude product was used into next step without further purification. The crude product was dissolved in 2 mL dry DCM. TBSCl (15 mg, 0.1 mmol) and imidazole (7 mg, 0.1 mmol) were added into the reaction mixture subsequently. The reaction mixture was stirred at room temperature for 2 h. The reaction mixture was diluted with 10 mL DCM and washed with saturated NaH_2PO_4 and saturated NaCl. The combined organic layers were dried with Na_2SO_4 and concentrated *in vacuo* to afford the crude product. Chromatography purification on silica gel using hexane/ethyl acetate = 4:1 to 3:1 as the eluents gave the purified product **17** which was used for HPLC analysis.

Note: The racemic **17** for analytic method development on chiral stationary phase HPLC was prepared through reaction between TBS-protected **8**, **14** and **15** in toluene. Other racemic rotaxanes were prepared in similar manner.

Preparation of cavitand 7 (93: 7 er)

The synthesis of **19**

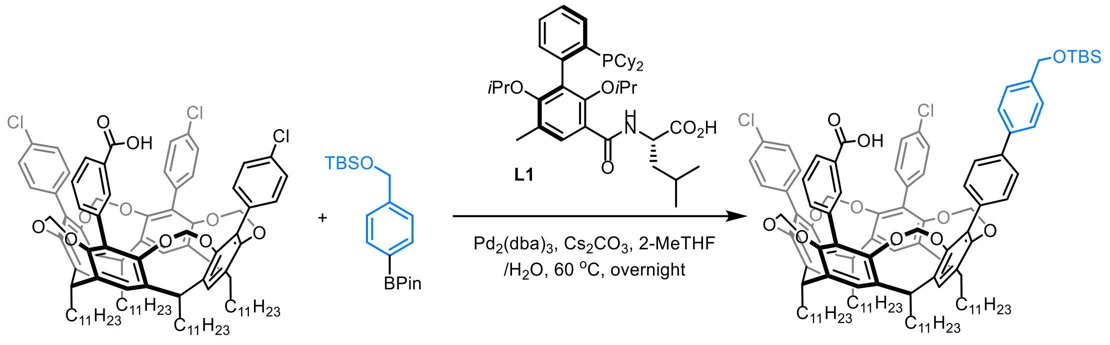

Under N_2 atmosphere in the glovebox, a 10 mL round bottom flask equipped with a magnetic stirring bar were added $Pd_2(dba)_3$ (6.0 mg, 0.0066 mmol), **L1** (11.0 mg, 0.0176 mmol), and 1.0 mL 2-MeTHF. The mixture was sealed with a cap (phenolic open top cap with red PTFE/white silicone septum), and stirred at room temperature for 20 min. The resulting metal-ligand complex solution was added to a sealed tube containing compound **1** (200 mg, 0.125 mmol), **18**[3] (52 mg, 0.15 mmol), 7.0 mL 2-MeTHF, Cs_2CO_3 (406 mg, 1.25 mmol), and degassed H_2O (400 µL). The vial was then sealed, and the resulting solution was stirred at 60 °C overnight. The reaction mixture was then cooled to room temperature, and 1 M HCl aqueous solution was added to adjust to pH= 1. The resulting mixture was then extracted with DCM three times. The combined organic phases were washed with water and brine, dried over Na_2SO_4, and concentrated *in vacuo* to afford the crude product **19**. The crude product was used directly in the next step.

The synthesis of **20**

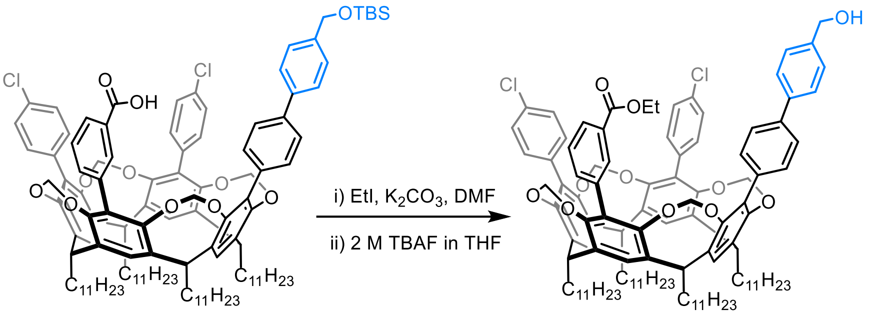

19 **20**

The crude product **19** was dissolved into 5 mL DMF. K_2CO_3 (172 mg, 1.25 mmol) and EtI (195 mg, 1.25 mmol) were added into the above reaction mixture. The mixture was stirred at room temperature for two hours. The reaction mixture was then diluted with water and extracted with ethyl acetate three times. The combined organic layers were dried with Na_2SO_4 and concentrated *in vacuo* to afford the crude product (246 mg). The crude product was then mixed with 2 mL 2M TBAF in THF and the solution was stirred at room temperature for 2 h. After TLC indicated the disappearing starting material, the reaction mixture was diluted with 10 mL ethyl acetate. The organic phase was washed with 10 mL H_2O and 10 mL saturated NaCl subsequently. The combined organic layers were dried with Na_2SO_4 and concentrated *in vacuo* to afford the crude product. Chromatography purification on silica gel using hexane/ethyl acetate (3:1-2:1) as the eluents gave the purified product **20** (68 mg, 32% yield for three steps) as a yellow oil.

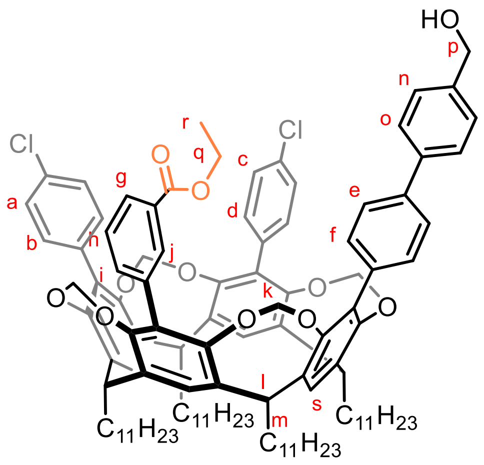

R_f = 0.2 (hexane/ethyl acetate =3:1).

^{1}H NMR (500 MHz, $CDCl_3$) δ 7.95 (dt, *J* = 7.7, 1.4 Hz, 1H, H_h), 7.60 – 7.56 (d, *J* = 8.2 Hz, 2H, H_e), 7.55 (s, 1H, H_j), 7.54 – 7.52 (d, *J* = 8.4 Hz, 2H, H_n), 7.44 – 7.40 (m, 3H, H_f), 7.39 – 7.36 (m, 1H), 7.35 – 7.27 (m, 8H, H_s), 7.10 (d, *J* = 8.2 Hz, 2H, H_f), 7.02 (d, *J* = 8.4 Hz, 2H, H_o), 6.97 (d, *J* = 8.4 Hz, 2H, H_b), 5.28 (d, *J* = 7.0 Hz, 1H, H_k), 5.23 (dd, *J* = 7.0, 3.9 Hz, 2H, H_k), 5.19 (d, *J* = 7.0 Hz, 1H, H_k), 4.89 – 4.81 (m, 4H, H_l), 4.74 (s, 2H, H_p), 4.36 (q, *J* = 7.1 Hz, 2H, H_q), 4.28 – 4.19 (m, 4H, H_k), 2.38 – 2.30 (m, 8H, H_m, 4×CH_2), 1.51 – 1.41 (m, 16H, H_m, 4×CH_2), 1.37 – 1.27 (m, 59H, H_m, 28×CH_2+CH_3), 0.89 (t, *J* = 6.8 Hz, 12H, H_m, 4×CH_3).

Note: 24 H (6.97 – 7.95 ppm) for aryl groups in compound **20**; $H_{c,d}$, H_a and H_h resonate at 7.39 –7.27 ppm, but their signals could not be clearly distinguished.

^{13}C NMR (126 MHz, $CDCl_3$) δ 166.6, 152.74, 152.70, 152.62, 152.58, 152.54, 152.50, 140.0, 139.5, 138.5, 138.4, 138.35, 138.30, 138.26, 135.1, 134.4, 133.2, 132.9, 132.5, 132.4, 131.3, 131.2, 130.3, 130.2, 129.9, 129.1, 128.9, 128.5, 128.3, 128.2, 127.5, 127.2, 126.5, 120.3, 120.2, 120.1, 119.9, 100.6, 100.56, 100.47, 65.1, 61.2, 37.1, 32.0, 30.4, 29.9, 29.80, 29.77, 29.4, 28.0, 22.7, 14.3, 14.1.

Note*:* The observed overlap of ^{13}C NMR signals in the 119.9–152.74 ppm region is likely a result of the symmetry presents in the cavitand scaffold. 46 non-equivalent aromatic carbons in theory, 36 signals recorded; The observed overlap of ^{13}C NMR signals in the 14.3–37.1 ppm region is likely a result of the symmetry present in the cavitand scaffold. 44 non-equivalent carbons for the $C_{11}H_{23}$ groups in theory, 11 signals recorded.

HRMS (m/z, APCI): Calcd. for Chemical Formula: $[C_{110}H_{137}{}^{35}Cl_2O_{11}]^+$$[M+H]^+$: 1703.9532, Found: 1703.9537.

The synthesis of **S8** for the determination of enantiopurity of **20**

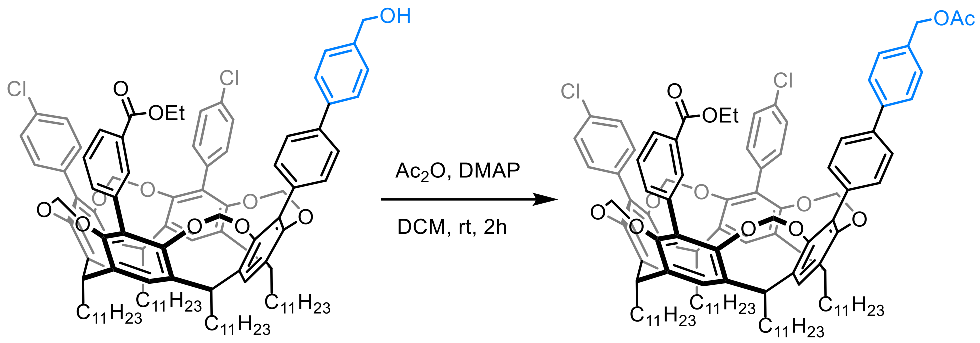

20 **S8**

To a solution of **20** (15 mg, 0.0088 mmol) in 2 mL DCM were added DMAP (11 mg, 0.088 mmol, 10 equiv.) and Ac_2O (9 mg, 0.088 mmol, 10 equiv.). The reaction stirred at room temperature for 2 h. The reaction mixture was then diluted with 10 mL DCM and washed with saturated aqueous solution of NaCl. The combined organic layers were dried with Na_2SO_4 and concentrated *in vacuo* to afford the crude product. Chromatography purification on PTLC using hexane/ethyl acetate (8:1) as the eluents gave the purified product **S8** (14.4 mg, 94%) as a colorless oil.

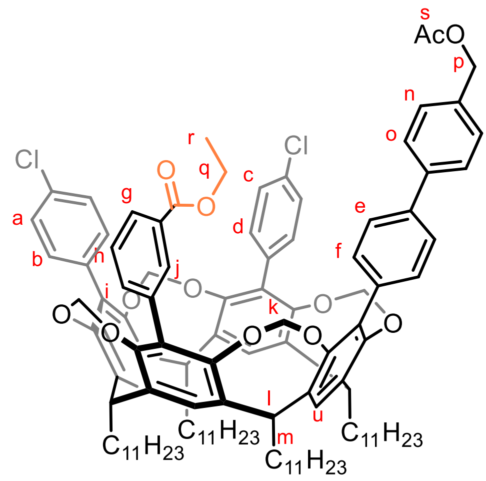

R_f = 0.4 (hexane/ethyl acetate =10:1)

^{1}H NMR (500 MHz, $CDCl_3$) δ 7.96 (d, *J* = 7.6 Hz, 1H, H_h), 7.58 (s, 1H, H_j), 7.51 (d, *J* = 7.6 Hz, 2H, H_e), 7.45 – 7.38 (m, 3H, H_f and H_g), 7.38 – 7.30 (m, 8H, H_u), 7.30 – 7.27 (m, 3H), 7.08 – 6.94 (m, 6H), 5.26 – 5.18 (m, 4H, H_k), 5.13 (s, 2H, H_p), 4.89 – 4.82 (m, 4H, H_l), 4.35 (q, *J* = 7.0 Hz, 2H, H_q), 4.28 – 4.22 (m, 3H, H_k), 4.20 (d, *J* = 7.2 Hz, 1H, H_k), 2.40 – 2.29 (m, 8H, H_m, 4×CH_2), 1.52 – 1.41 (m, 16H, H_m, 8×CH_2), 1.37 – 1.27 (m, 59H, H_m and H_r, 28×CH_2+CH_3), 0.89 (t, *J* = 6.8 Hz, 12H, H_m, 4×CH_3).

Note: 24 H (6.94 – 7.96 ppm) for aryl groups in compound **S8**; $H_{a\text{-}d}$ and H_h resonate at 7.30 –6.94 ppm, but their signals could not be clearly distinguished.

^{13}C NMR (126 MHz, $CDCl_3$) δ 171.0, 166.5, 152.74, 152.72, 152.6, 140.7, 139.2, 138.5, 138.4, 138.3, 135.0, 134.9, 134.3, 133.22, 133.19, 133.1, 132.5, 131.28, 131.23, 130.2, 130.0, 129.2, 128.7, 128.6, 128.3, 128.2, 127.2, 126.5, 120.2, 120.14, 120.11, 100.5, 66.0, 61.2, 37.1, 32.0, 30.4, 29.9, 29.80, 29.76, 29.4, 28.0, 24.9, 22.7, 14.3, 14.1.

Note*:* The observed overlap of ^{13}C NMR signals in the 120.11–152.74 ppm region is likely a result of the symmetry presents in the cavitand scaffold. 46 non-equivalent aromatic carbons in theory, 29 signals recorded; The observed overlap of ^{13}C NMR signals in the 14.1–37.1 ppm region is likely a result of the symmetry present in the cavitand scaffold. 44 non-equivalent carbons for the $C_{11}H_{23}$ groups in theory, 12 signals recorded.

HPLC analysis of this compound: Daicel Chiralpak IC, hexane/*iso*-propanol = 98:2, 1.0 mL/min, temperature = 40 ^{o}C, λ = 254 nm, retention time: 7.9 min (minor) and 10.3 min (major).

HRMS (m/z, APCI): Calcd. for Chemical Formula: $[C_{112}H_{139}{}^{35}Cl_2O_{12}]^+[M+H]^+$: 1745.9565, Found: 1745.9560.

The synthesis of **S9**

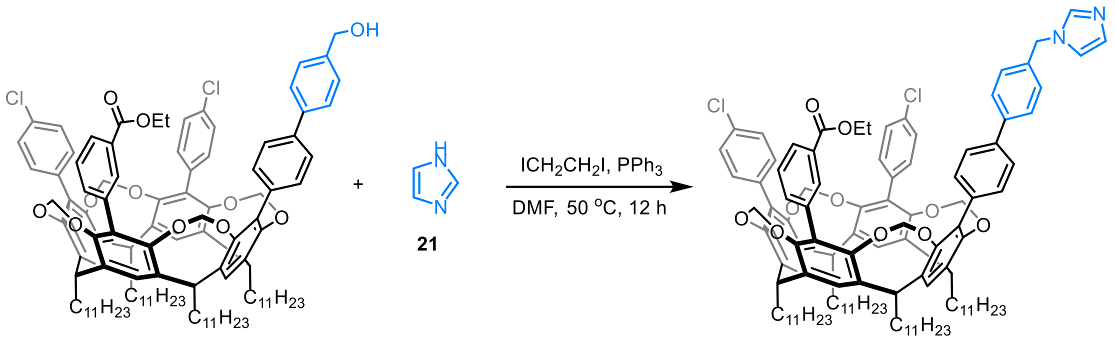

To a solution of alcohol **20** (87 mg, 0.051 mmol) in 5 mL dry DMF were added 1,2-diiodoethane (17 mg, 0.061 mmol, 1.2 equiv.) and PPh_3 (16 mg, 0.061 mmol, 1.2 equiv.). The solution was stirred at room temperature for 5 min. Imidazole **21** (10 mg, 0.153 mmol, 3 equiv.) was added into the reaction mixture and stirred at 50 °C for 12 h. The mixture was diluted with 10 mL ethyl acetate and washed with 20 mL water and 10 mL saturated NaCl. The combined organic layers were dried with Na_2SO_4 and concentrated *in vacuo* to afford the crude product. Chromatography purification on silica gel using 100% ethyl acetate as the eluents gave the purified product **S9** (73 mg, 82% yield) as a colorless oil.

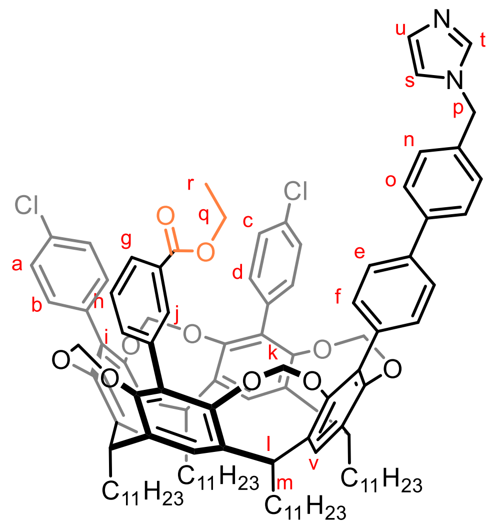

R_f = 0.4 (100% ethyl acetate).

^{1}H NMR (500 MHz, $CDCl_3$) δ 8.10 (br, 1H, H_t), 7.95 (d, *J* = 7.6 Hz, 1H, H_h), 7.60 (d, *J* = 8.0 Hz, 2H, H_n), 7.57 – 7.54 (s, 1H, H_j), 7.54 – 7.50 (d, *J* = 8.2 Hz, 2H, H_e) 7.44 – 7.27 (m, 13H, H_v and $H_{a\text{-}d}$), 7.11 (d, *J* = 8.0 Hz, 2H, H_o), 7.05 – 7.00 (d, *J* = 8.2 Hz, 2H, H_f), 7.00 – 6.94 (m, 3H), 5.30 – 5.13 (m, 6H, H_k and H_p), 4.88 – 4.81 (m, 4H, H_l), 4.36 (q, *J* = 7.1 Hz, 2H, H_q), 4.27 – 4.18 (m, 4H, H_k), 2.38 – 2.31 (m, 8H, H_m, 4×CH_2), 1.50 – 1.42 (m, 16H, H_m, 8×CH_2), 1.36 – 1.27 (m, 59H, H_m and H_r, 28×CH_2), 0.90 – 0.87 (m, 12H, H_m, 4×CH_3).

Note: 27 H (6.94 – 8.10 ppm) for aryl groups in compound **S9**; $H_{a\text{-}d}$, H_s and H_i resonate at 7.44 –7.27 and 7.00 – 6.94 ppm, but their signals could not be clearly distinguished.

^{13}C NMR (126 MHz, $CDCl_3$) δ 166.6, 152.7, 152.5, 138.7, 138.4, 135.1, 134.5, 133.2, 132.5, 131.3, 131.2, 130.4, 130.0, 128.5, 128.4_5, 128.4_3, 128.2, 126.6, 122.7, 122.1, 120.2, 100.6, 100.5_4, 100.4_8, 64.8, 61.3, 37.1, 32.0, 30.4, 29.9, 29.8_0, 29.7_6, 29.4, 28.0, 22.7, 14.3, 14.1.

Note*:* The observed overlap of ^{13}C NMR signals in the 120.2–152.7 ppm region is likely a result of the symmetry presents in the cavitand scaffold. 49 non-equivalent aromatic carbons in theory, 20 signals recorded; The observed overlap of ^{13}C NMR signals in the 14.1–37.1 ppm region is likely a result of the symmetry present in the cavitand scaffold. 44 non-equivalent carbons for the $C_{11}H_{23}$ groups in theory, 11 signals recorded.

HRMS (m/z, APCI): Calcd. for Chemical Formula: $[C_{113}H_{139}{}^{35}Cl_2N_2O_{10}]^+$$[M+H]^+$: 1753.9729, Found: 1753.9746.

The synthesis of **7** (93:7 e.r.)

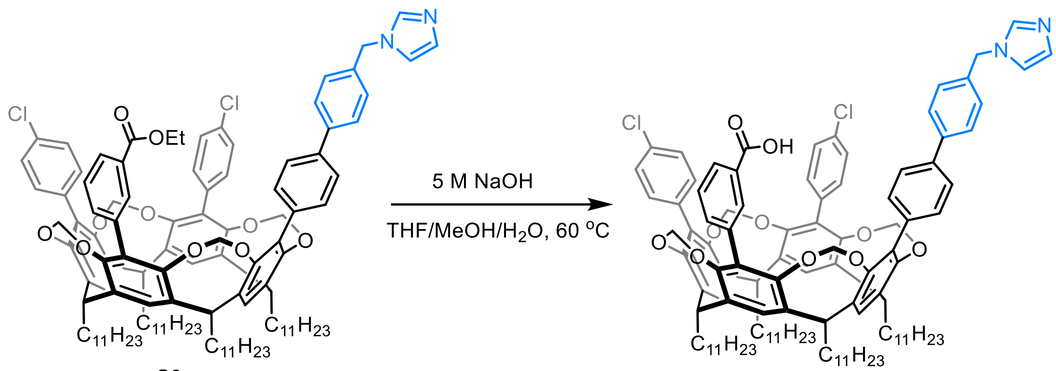

To a solution of **S9** (37 mg, 0.021 mmol) in THF/MeOH (1 mL THF/0.5 mL MeOH) was added 0.5 mL 5 M NaOH (2.5 mmol) in H_2O. The solution was heat at 60 °C and allowed to stir at the temperature for 2h. The solution was concentrated under vacuum and then dissolved in 10 mL ethyl acetate. The mixture was washed with 10 mL saturated NaCl. The combined organic layers were dried with Na_2SO_4 and concentrated *in vacuo* to afford the crude product. Chromatography purification on silica gel using 100% ethyl acetate as the eluents gave the purified product **7** (31 mg, 85% yield) as a colorless oil. The product is consistent with **7** prepared via desymmetrizing borylation of **2** followed by Suzuki coupling (page S11).

General Procedure for rotaxane synthesis using slow addition

The synthesis of compound **22**

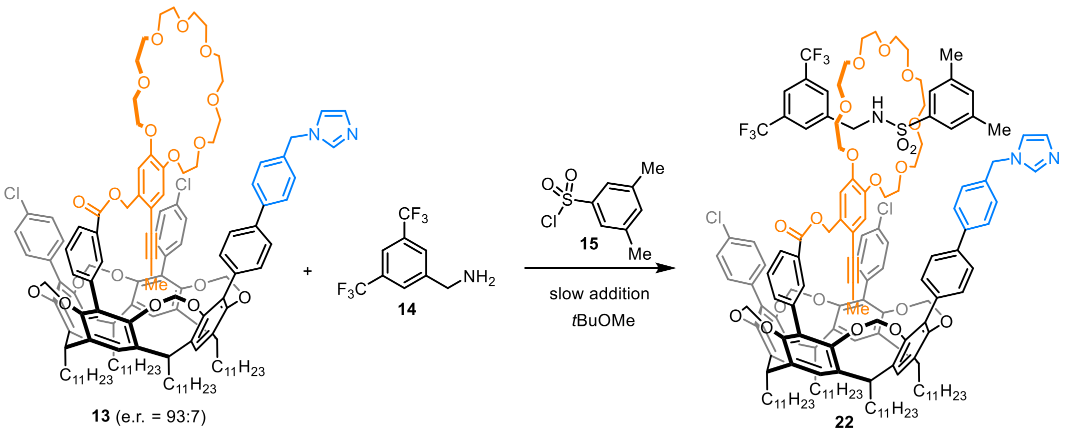

Under N_2 atmosphere in the glovebox, a 2 mL flask equipped with a magnetic stirring bar were added **13** (5 mg, 0.0023 mmol), 3,5-bis(trifluoromethyl)benzylamine **14** (2.3 mg, 0.0092 mmol, 4 equiv.) in 0.2 mL *t*BuOMe. 3,5-dimethylbenzenesulfonyl chloride **15** (0.94 mg, 0.0046 mmol, 2 equiv.) in 0.2 mL *t*BuOMe was added into the reaction mixture by syringe pump within 4 h at room temperature (Syringe BD Plastic 1 mL 4.699 mm, infuse only, 1.6 uL/min). The reaction mixture was then stirred at room temperature for 12 hours. After the reaction was completed, the mixture was diluted with 10 mL ethyl acetate and washed with 20 mL 1 M NaH_2PO_4 and 10 mL saturated NaCl. The combined organic layers were dried with Na_2SO_4 and concentrated *in vacuo* to afford the crude product. Chromatography purification on silica gel using hexane/ethyl acetate (1:3) as the eluents gave the purified product **22**.

The synthesis of rotaxane **17**

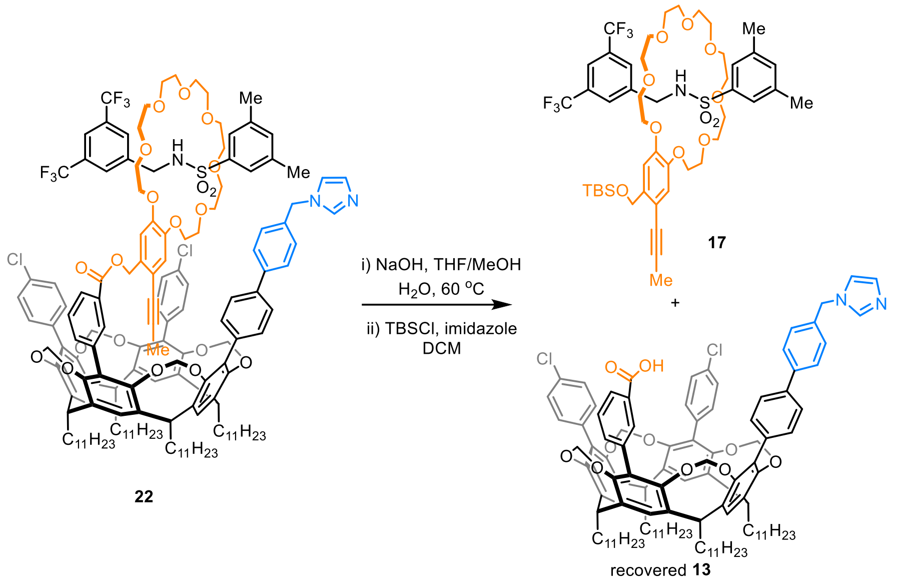

After basic hydrolysis and TBS protection (same as the procedure described previously for the reactions in toluene), the reaction offered the purified product **17** (1.6 mg, 72% for three steps) as colorless oil. **13** was recovered in 82% yield (4.1 mg **13** was recovered from the reaction mixture.)

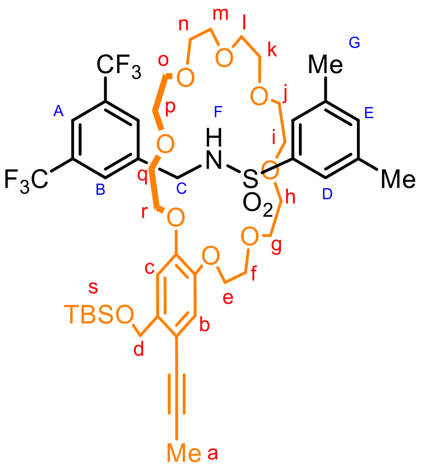

R$_f$ = 0.3 (hexane/ethyl acetate = 4: 1).

[α]$_D^{25}$ = – 37.1 (*c* 0.07 $CHCl_3$) for e.r. = 90:10

^{1}H NMR (500 MHz, $CDCl_3$) δ 8.58 (s, 2H, H_B), 7.56 (s, 1H, H_A), 7.44 (s, 2H, H_D), 6.97 (s, 1H, H_E), 6.81 (s, 1H, H_c), 6.58 (s, 1H, H_b), 6.52 (t, *J* = 5.7 Hz, 1H, H_F), 5.30 (s, 1H, H_d), 4.75 (d, *J* = 4.4 Hz, 1H, H_d), 4.63 (d, *J* = 5.7 Hz, 2H, H_C), 4.01 – 3.96 (m, 1H, H_r), 3.94 – 3.87 (m, 2H, H_r and H_e), 3.87 – 3.82 (m, 1H, H_e), 3.73 –

3.57 (m, 7H, H_{f-q}), 3.56 – 3.46 (m, 7H, H_{f-q}), 3.39 – 3.26 (m, 10H, H_{f-q}), 2.97 – 2.91 (m, 2H, H_{f-q}), 2.25 (s, 6H, H_G), 2.07 (s, 3H, H_a), 0.96 (s, 9H, H_s, *t*Bu), 0.11 (d, J = 7.1 Hz, 6H, H_s, 2×CH_3).

^{19}F NMR (377 MHz, $CDCl_3$) δ – 62.0.

^{13}C NMR (126 MHz, $CDCl_3$) δ 147.9, 146.1, 141.2, 140.3, 138.2, 135.8, 133.2, 131.4, 129.4, 129.1, 125.1, 122.9, 119.4, 114.7, 112.1, 109.6, 88.5, 71.0, 70.9, 70.8, 70.7_1, 70.6_7, 70.5, 69.8_5, 69.8_2, 68.2, 68.0, 62.9, 45.9, 29.7, 26.0, 21.2, 18.4, 4.5, 1.0, – 5.3.

Note: The observed overlap of ^{13}C NMR signals stacking in the 68.0–71.0 ppm region is the glycol groups in crown ether. 14 non-equivalent carbons in theory, 10 signals recorded.

HPLC analysis of this compound: Daicel Chiralpak IF, hexane/*iso*-propanol = 95: 5, 1.0 mL/min, temperature = 40 °C, λ = 254 nm, retention time: 20.4 min (major) and 21.7 min (minor).

HRMS (m/z, APCI): Calcd. for Chemical Formula: $[C_{47}H_{66}F_6NO_{11}SSi]^+[M+H]^+$: 994.4025, Found: 994.4024.

The assignment of compound **17** by 2D NMR

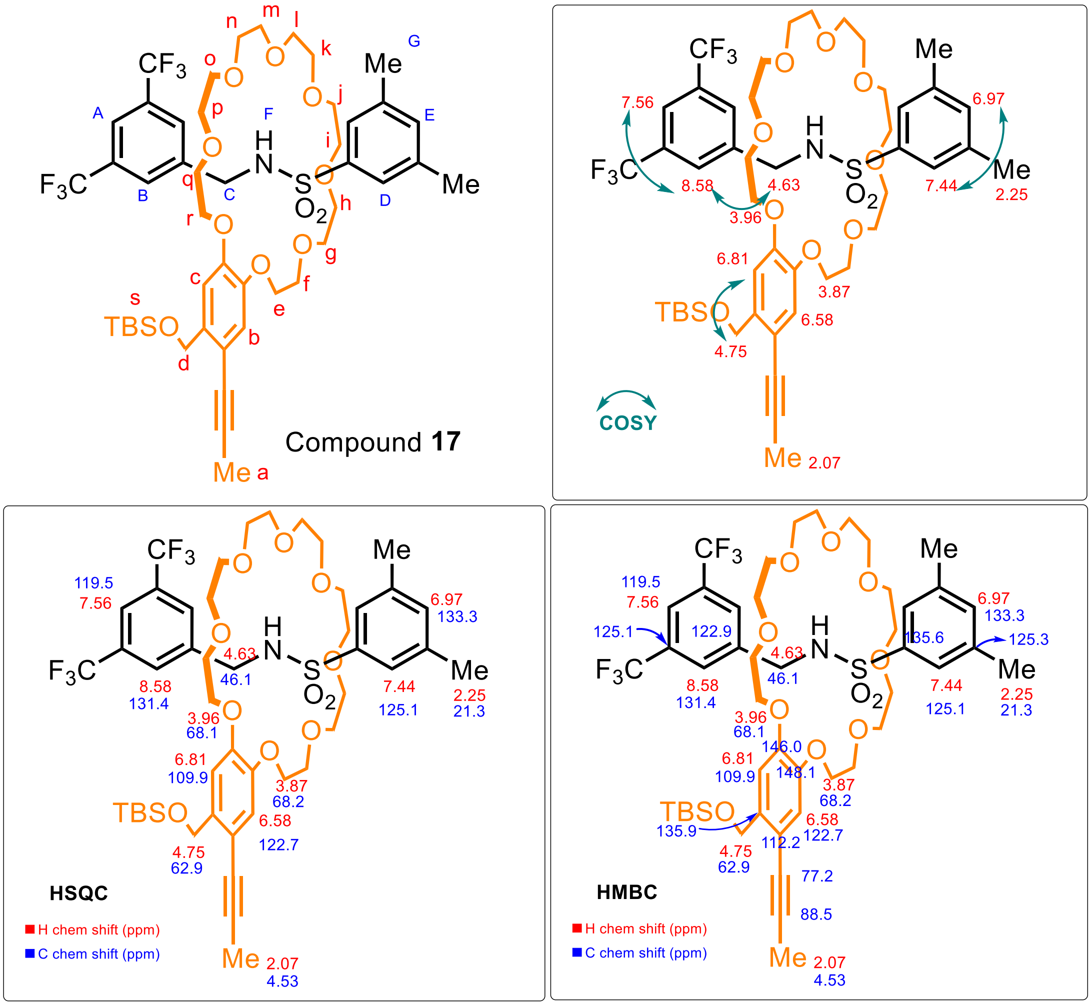

The synthesis of **23**

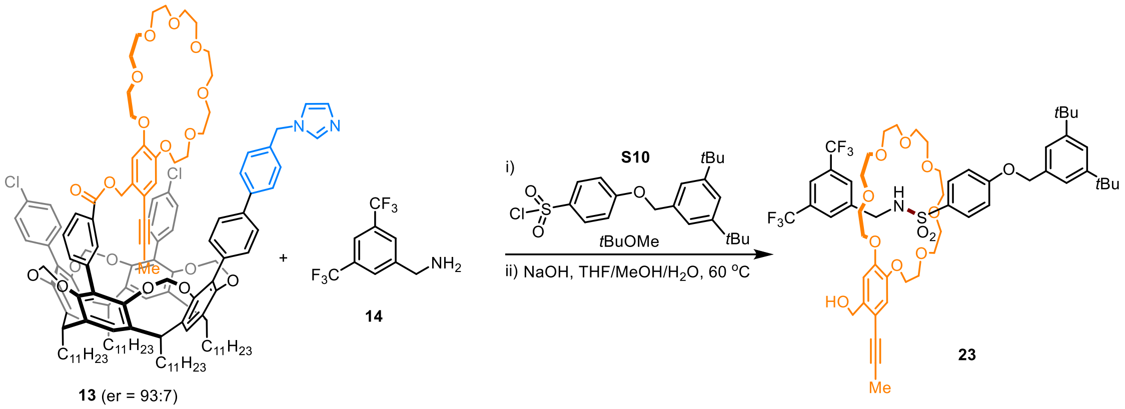

According to the general procedure for slow addition, a reaction was conducted using **13** (5 mg, 0.0023 mmol, er = 93:7), sulfonyl chloride **S10**[4] (1.8 mg, 0.0046 mmol, 2 equiv.), and 3,5-bis(trifluoromethyl)benzylamine **14** (2.3 mg, 0.0092 mmol, 4 equiv.) in 0.4 mL of *t*BuOMe. The reaction afforded product **23** (1.4 mg, 57% yield) as a colorless oil.

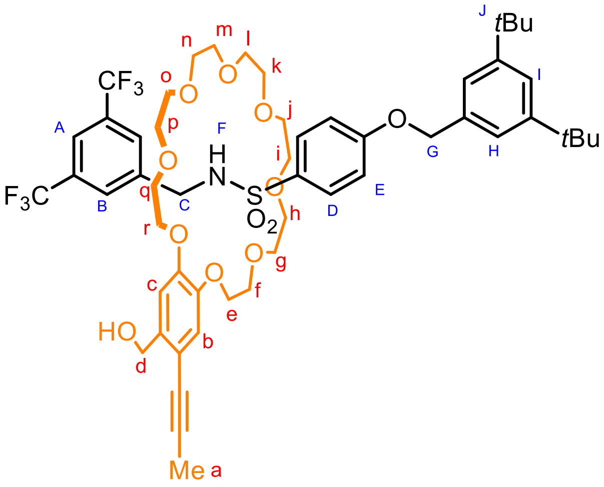

R_f = 0.4 (hexane/ethyl acetate = 2: 1).

$[\alpha]_D^{25}$ = 60 (*c* 0.05 $CHCl_3$) for e.r. = 89: 11

^{1}H NMR (500 MHz, $CDCl_3$) δ 8.59 (s, 2H, H_B), 7.67 (d, *J* = 8.9 Hz, 2H, H_D), 7.59 (s, 1H, H_A), 7.42 (t, *J* = 1.8 Hz, 1H, H_I), 7.29 (d, *J* = 1.8 Hz, 2H, H_H), 6.84 (d, *J* = 8.9 Hz, 2H, H_E), 6.73 (d, *J* = 11.0 Hz, 2H, H_c and H_b), 6.52 (t, *J* = 5.7 Hz, 1H, H_F), 5.05 (s, 2H, H_d), 4.74 (d, *J* = 12.8 Hz, 1H, H_C), 4.67 – 4.62 (m, 3H, H_C and H_G), 4.01 (td, *J* = 11.1, 5.9 Hz, 2H, H_r), 3.95 – 3.85 (m, 2H, H_e), 3.73 – 3.63 (m, 4H, $H_{f\text{-}q}$), 3.61 – 3.55 (m, 2H, $H_{f\text{-}}$

$_q$), 3.54 – 3.49 (m, 4H, H_{f-q}), 3.43 – 3.36 (m, 2H, H_{f-q}), 3.43 – 3.37 (m, 2H, H_{f-q}), 3.34 – 3.28 (m, 5H, H_{f-q}), 3.28 – 3.22 (m, 3H, H_{f-q}), 2.87 – 2.82 (m, 2H, H_{f-q}), 2.02 (s, 3H, H_a), 1.35 (s, 18H, H_J).

^{19}F NMR (377 MHz, $CDCl_3$) δ -62.2.

^{13}C NMR (126 MHz, $CDCl_3$) δ 161.2, 151.3, 148.1, 147.0, 141.3, 131.2, 129.4, 122.5, 122.0, 119.4, 115.2, 114.6, 113.7, 111.2, 88.7, 71.2, 71.01, 70.96, 70.84, 70.79, 70.71, 70.69, 70.54, 70.47, 70.4, 69.7, 68.25, 68.21, 63.8, 45.9, 34.9, 31.5, 29.7, 4.5. 1.0

Note: The observed overlap of ^{13}C NMR signals stacking in the 68.0–71.0 ppm region is the glycol groups in crown ether. 14 non-equivalent carbons in theory, 13 signals recorded.

HPLC analysis of this compound: Daicel Chiralpak IF, hexane/*iso*-propanol = 80: 20, 1.0 mL/min, temperature = 40 °C, λ = 254 nm, retention time: 13.9 min (major) and 19.6 min (minor).

HRMS (m/z, APCI): Calcd. for Chemical Formula: $[C_{54}H_{70}F_6NO_{12}S]^+[M+H]^+$: 1070.4517, Found: 1070.4508.

The synthesis of **24**

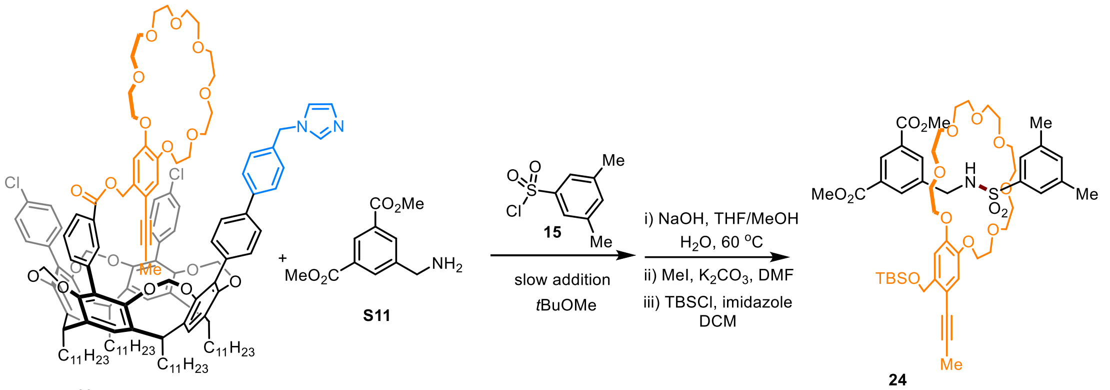

13 (er = 93:7)

According to the general procedure for slow addition, a reaction was conducted using **13** (5 mg, 0.0023 mmol), 3,5-dimethylbenzenesulfonyl chloride **15** (0.94 mg, 0.0046 mmol, 2 equiv.) and **S11**[2] (2.1 mg, 0.0094 mmol, 4 equiv.) in 0.4 mL *t*BuOMe. The reaction afforded the product **24** (1.1 mg, 48% yield) as colorless oil.

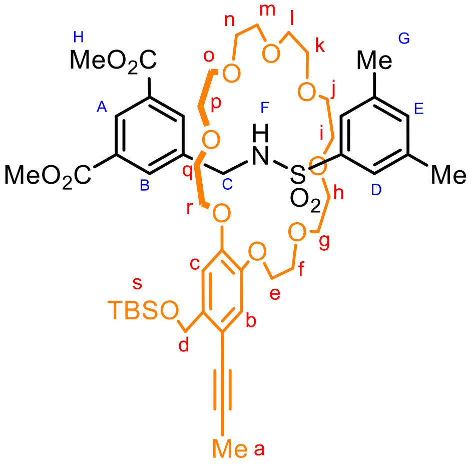

R$_f$ = 0.3 (hexane/ethyl acetate = 1: 1).

[α]$_D^{25}$ = 12 (*c* 0.05 $CHCl_3$) for e.r. = 78:22

^{1}H NMR (400 MHz, $CDCl_3$) δ 8.71 (d, *J* = 1.5 Hz, 2H, H_B), 8.33 (t, *J* = 1.5 Hz, 1H, H_C), 7.49 (s, 2H, H_D), 6.99 (s, 1H, H_E), 6.74 (s, 1H, H_c), 6.61 (t, *J* = 5.8 Hz, 1H, H_F), 6.58 (s, 1H, H_b), 4.73 (d, *J* = 6.1 Hz, 2H, H_d), 4.70 (d, *J* = 6.0 Hz, 2H, H_C), 4.02 – 3.94 (m, 2H, H_r), 3.91 – 3.87 (m, 1H, H_e), 3.87 – 3.85 (m, 1H, H_q), 3.84 (s, 6H, H_H), 3.67 – 3.50 (m, 12H, $H_{f\text{-}q}$), 3.46 – 3.34 (m, 10H, $H_{f\text{-}q}$), 3.21 – 3.13 (m, 2H, $H_{f\text{-}q}$), 2.27 (s, 6H, H_G),

2.06 (s, 3H, H_a), 0.95 (s, 9H, H_s, *t*Bu), 0.10 (d, *J* = 2.4 Hz, 6H, H_s, 2×CH_3).

^{13}C NMR (126 MHz, $CDCl_3$) δ 166.9, 148.0, 146.3, 140.6, 139.8, 138.1, 136.1, 135.6, 133.1, 128.9, 128.4, 125.2, 114.8, 111.9, 109.6, 88.4, 70.9, 70.8$_3$, 70.8$_0$, 70.7, 70.6$_4$, 70.6$_1$, 69.7$_5$, 69.7$_1$, 68.1, 68.0, 63.0, 51.9, 45.9, 26.0, 21.3, 4.5, -5.3.

Note: The observed overlap of ^{13}C NMR signals stacking in the 45.9–70.9 ppm region is the glycol groups in crown ether. 14 non-equivalent carbons in theory, 10 signals recorded.

HPLC analysis of this compound: Daicel Chiralpak IF, hexane/*iso*-propanol = 80: 20, 1.0 mL/min, temperature = 40 °C, λ = 220 nm, retention time: 13.7 min (major) and 14.7 min (minor).

HRMS (m/z, APCI): Calcd. for Chemical Formula: $[C_{49}H_{72}NO_{15}SSi]^+[M+H]^+$: 974.4386, Found: 974.4384.

The synthesis of **25**

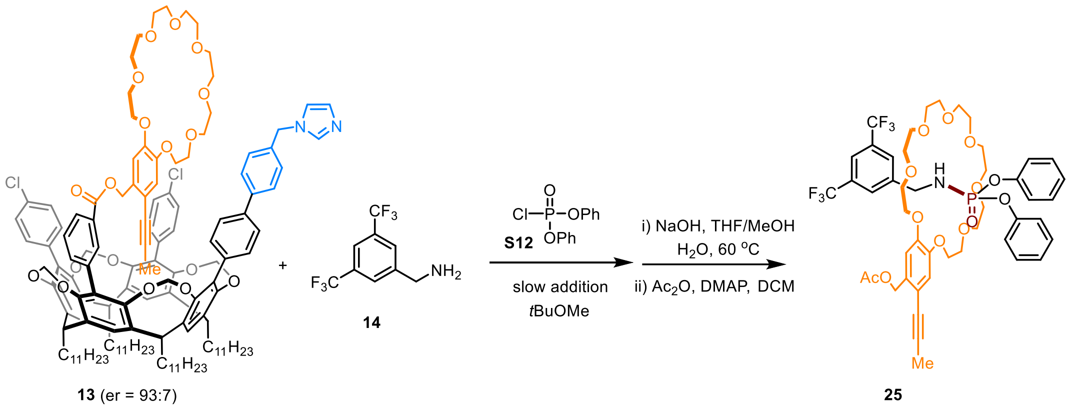

According to the general procedure for slow addition, a reaction was conducted using **13** (4.9 mg, 0.0022 mmol, er = 93:7), 3,5-bis(trifluoromethyl)benzylamine **14** (2.1 mg, 0.0088 mmol, 4 equiv.) and diphenyl chlorophosphite **S12** (1.2 mg, 0.0044 mmol, 2 equiv.) in 0.4 mL *t*BuOMe. The reaction afforded the product **25** (1.7 mg, 77% yield) as colorless oil.

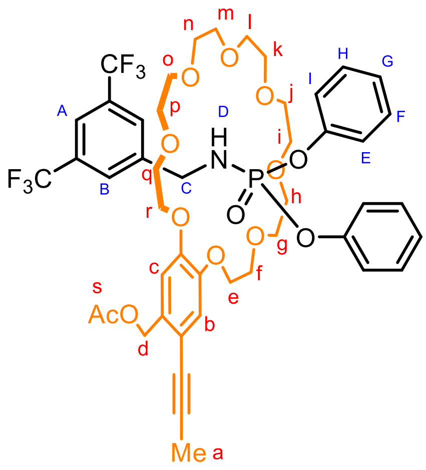

R$_f$ = 0.3 (hexane/ethyl acetate = 1:1).

[α]$_D^{25}$ = 25 (*c* 0.05 CHCl$_3$) for e.r. = 72: 28

^{1}H NMR (400 MHz, CDCl$_3$) δ 8.62 (s, 2H, H$_B$), 7.57 (s, 1H, H$_A$), 7.24 – 7.17 (m, 4H, H$_{E-I}$), 7.17 – 7.12 (m, 4H, H$_{E-I}$), 7.09 – 7.03 (m, 2H, H$_{E-I}$), 6.70 (d, *J* = 1.3 Hz, 1H, H$_D$), 5.32 (dt, *J* = 12.0, 5.9 Hz, 1H, H$_d$), 4.91 – 4.85 (m, 2H, H$_C$), 4.11 – 4.04 (m, 2H, H$_r$), 4.00 – 3.92 (m, 2H, H$_e$), 3.74 – 3.68 (m, 2H, H$_{f-q}$), 3.62 – 3.47 (m, 13H, H$_{f-q}$), 3.35 – 3.23 (m, 8H, H$_{f-q}$), 3.01 – 2.94 (m, 2H, H$_{f-q}$), 2.10-2.03 (m, 6H, H$_a$ and H$_s$).

^{13}C NMR (126 MHz, $CDCl_3$) δ 151.4, 148.8, 148.0, 147.7, 147.6, 143.10, 143.06, 143.0, 142.9, 130.9, 129.2, 126.6, 124.2, 120.81, 120.77, 119.1, 115.9, 115.0, 113.7, 112.4, 88.5, 70.9, 70.82, 70.78, 70.6, 70.4, 69.70, 69.65, 69.6, 69.5, 68.7, 68.4, 68.3, 66.0, 64.8, 44.3, 29.7, 21.0, 20.9, 4.5.

Note: The observed overlap of ^{13}C NMR signals stacking in the 68.3–70.9 ppm region is the glycol groups in crown ether. 14 non-equivalent carbons in theory, 12 signals recorded.

HPLC analysis of this compound: Daicel Chiralpak IF, hexane/*iso*-propanol = 80: 20, 1.0 mL/min, temperature = 40 ^{o}C, λ = 254 nm, retention time: 22.4 min (major) and 25.4 min (minor).

HRMS (m/z, APCI): Calcd. for Chemical Formula: $[C_{47}H_{55}F_6NO_{13}P]^+[M+H]^+$: 986.3310, Found:986. 3320.

The synthesis of **26**

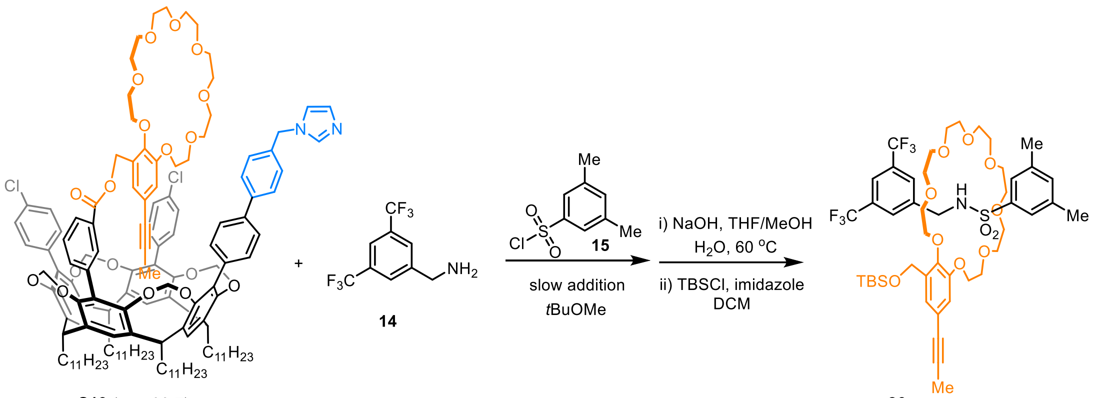

S13 (er = 93:7)   **26**

According to the general procedure for slow addition, a reaction was conducted using **S13** (7.3 mg, 0.0034 mmol, er = 93:7), 3,5-bis(trifluoromethyl)benzylamine **14** (3.3 mg, 0.0136 mmol, 4 equiv.) and 3,5-dimethylbenzenesulfonyl chloride **15** (1.4 mg, 0.0068 mmol, 2 equiv.) in 0.4 mL *t*BuOMe. The reaction afforded the product **26** (1.6 mg, 48% yield) as colorless oil.

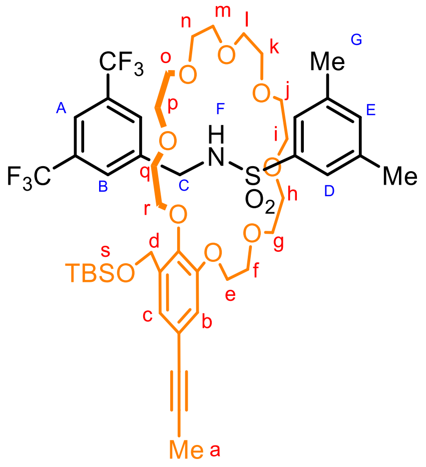

R_f = 0.3 (hexane/ethyl acetate = 3: 1).

$[\alpha]_D^{25}$ = 2.1 (*c* 0.12 $CHCl_3$) for e.r. = 75:25

^{1}H NMR (500 MHz, $CDCl_3$) δ 8.46 (s, 2H, H_B), 7.58 (s, 2H, H_D), 7.51 (s, 1H, H_A), 7.12 (s, 1H, H_E), 6.99 (s, 1H, H_c), 6.76 (t, *J* = 5.7 Hz, 1H, H_F), 6.68 (s, 1H, H_b), 4.75 (dd, *J* = 14.1, 6.0 Hz, 1H, H_d), 4.63 (dd, *J* = 14.3, 5.6 Hz, 1H, H_d), 4.57 (d, *J* = 13.6 Hz, 1H, H_C), 4.47 (d, *J* = 13.8 Hz, 1H, H_C), 4.20 – 4.13 (m, 2H, H_r), 4.07 – 3.99 (m, 2H, H_e), 3.79 – 3.74 (m, 1H, H_{f-q}), 3.75 – 3.62 (m, 4H, H_{f-q}), 3.56 – 3.46 (m, 6H, H_{f-q}), 3.45 – 3.30 (m,

6H, H_{f-q}), 3.28 – 3.15 (m, 4H, H_{f-q}), 3.11 – 3.07 (m, 1H, H_{f-q}), 3.02 – 2.96 (m, 2H, H_{f-q}), 2.35 (s, 6H, H_G), 2.03 (s, 3H, H_a), 0.91 (s, 9H, H_s, *t*Bu), 0.07 (s, 6H, H_s, 2×CH_3).

^{13}C NMR (126 MHz, $CDCl_3$) δ 151.3, 145.9, 141.5, 138.8, 135.4, 133.7, 131.4, 129.8, 129.5, 126.0, 123.4, 119.7, 119.1, 115.4, 84.5, 80.5, 72.2, 71.5, 71.3$_4$, 71.2$_8$, 71.2$_2$, 71.2$_0$, 71.1$_7$, 71.1, 71.0, 70.5, 69.1, 60.6, 46.4, 26.4, 26.1, 21.7, 4.8, 1.5, -4.9.

Note: The observed overlap of ^{13}C NMR signals stacking in the 69.1–72.2 ppm region is the glycol groups in crown ether. 14 non-equivalent carbons in theory, 11 signals recorded.

HPLC analysis of this compound: Daicel Chiralpak IF, hexane/*iso*-propanol = 90: 10, 1.0 mL/min, temperature = 40 ^{o}C, λ = 254 nm, retention time: 12.4 min (minor) and 13.4 min (major).

HRMS (m/z, APCI): Calcd. for Chemical Formula: $[C_{47}H_{66}F_6NO_{11}SSi]^+[M+H]^+$: 994.4025, Found: 994.4024.

Enantiopurity upgrade for cavitand 7 (98:2 er) and rotaxane synthesis

Our initial attempts to upgrade the enantiopurity of cavitand **7** through esterification of **19** using (*R*)-BINOL failed to yield diastereomeric derivatives that were separable by chromatography. On the other hand, we discovered that enantioenrichment could be achieved by rerouting the synthesis through a dimeric intermediate **S15**. We postulated that the resorcinarene moieties self-encapsulate the terminal acetyl groups (^{1}H NMR, $CH_3C(=O)$ δ = −1.0 ppm, Δδ = −3.0 ppm) within the chiral dimer, thus facilitating their separation from the meso-isomeric dimer through silica gel chromatography.

The synthesis of **S14**

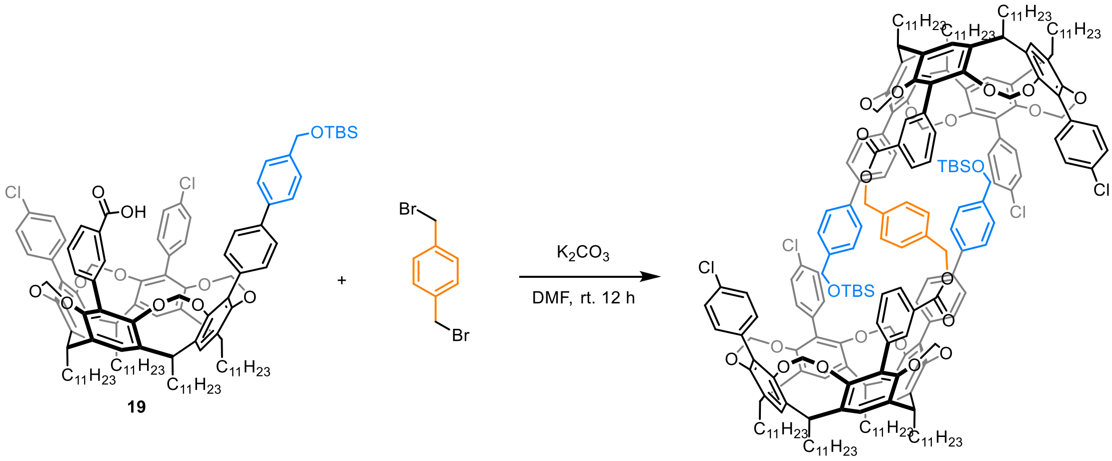

To a solution of **19** (42 mg, 0.023 mmol) and potassium carbonate (13 mg, 0.094 mmol, 4 equiv.) in 0.5 mL dry DMF was added 1,4-bis(bromomethyl)benzene (2.5 mg, 0.0094 mmol, 0.4 equiv.). The reaction stirred at room temperature for 12 h. The reaction mixture was diluted with 10 mL ethyl acetate and washed with saturated NaCl. The combined organic layers were dried with Na_2SO_4 and concentrated *in vacuo* to afford the crude product. Chromatography purification on silica gel using hexane/ethyl acetate (50:1) as the eluents gave the purified product **S14** (30 mg, 70%) as a colorless oil.

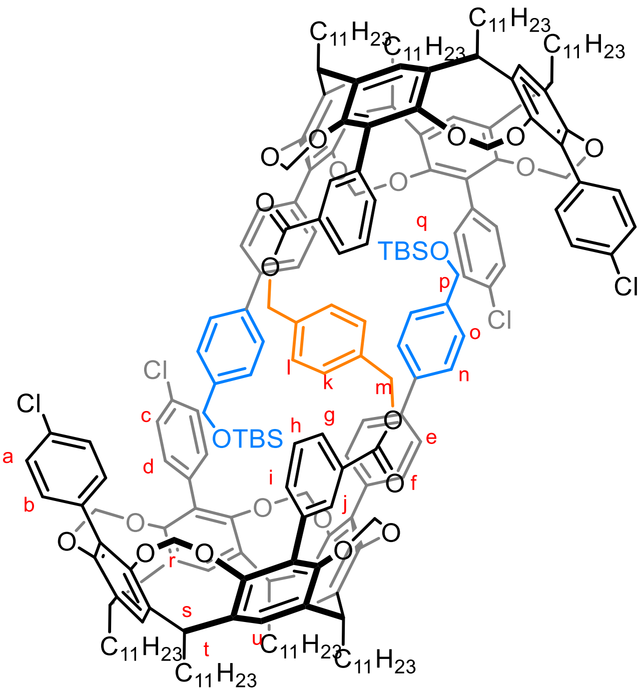

R$_f$ = 0.7 (hexane/ethyl acetate = 20:1)

^{1}H NMR (500 MHz, CDCl$_3$) δ 7.95 (d, *J* = 7.6 Hz, 2H, 2×H_h), 7.63 (s, 2H, 2×H_j), 7.55 (dd, *J* = 8.1, 4.0 Hz, 8H, 2×H_j , 2×H_g, 2×H_o and 2×H_e), 7.44 (s, 4H, 2×H_l and 2×H_k), 7.40 – 7.30 (m, 22H, H_u), 7.09 (d, *J* = 8.0 Hz, 4H, 2×H_n), 7.03 (d, *J* = 8.3 Hz, 4H, 2×H_f), 6.97 (d, *J* = 8.3 Hz, 4H, 2×H_b), 5.39 – 5.31 (m, 4H, 2×H_p), 5.27 (d, *J* = 6.9 Hz, 2H, 2×H_m), 5.25 – 5.20 (m, 4H, 2×H_m), 5.17 (d, *J* = 6.9 Hz, 2H, 2×H_m), 4.89 – 4.80 (m, 8H, 2×H_r), 4.77 (s, 4H, 2×H_s), 4.27 – 4.22 (m, 4H, 2×H_s), 4.20 (d, *J* = 6.9 Hz, 4H, 2×H_r), 2.39 – 2.31 (m, 16H, 2×H_t, 8×CH_2), 1.44 (s, 32H, 2×H_t, 16×CH_2), 1.29 (s, 112H, 2×H_t, 56×CH_2), 0.94 (s, 18H, 2×H_q, 2×*t*Bu), 0.89 (q, *J* = 6.8 Hz, 24H, 2×H_t, 8×CH_3), 0.11 (s, 12H, 2xH_q, 4×CH_3).

Note: 52 H (6.97 – 7.95 ppm) for aryl groups in compound **S14**; $H_{c,d}$, H_u and H_a resonate at 7.40 –7.30 ppm, but their signals could not be clearly distinguished.

^{13}C NMR (126 MHz, CDCl$_3$) δ 166.4, 152.8, 152.7, 152.6$_4$, 152.5$_9$, 152.5$_4$, 152.4$_9$, 140.7, 139.8, 139.2, 138.5, 138.4, 138.3$_3$, 138.2$_6$, 138.2, 136.0, 135.1, 134.7, 133.2, 132.7, 132.5$_1$, 132.4$_7$, 131.3, 131.2, 130.6, 130.2, 129.6, 129.2, 128.5$_4$, 128.5$_0$, 128.4$_7$, 128.4$_2$, 128.3$_8$, 128.3, 128.1, 126.9, 126.5, 120.3, 120.2, 120.1, 119.8, 100.6, 100.6$_2$, 100.5$_8$, 66.6, 64.8, 60.4, 37.1, 32.0, 30.4, 29.9, 29.8$_0$, 29.7$_7$, 29.4,

28.0, 26.0, 22.7, 14.1, -5.2.

Note*:* The observed overlap of ^{13}C NMR signals in the 119.8–152.8 ppm region is likely a result of the symmetry presents in the cavitand scaffold. 48 non-equivalent aromatic carbons in theory, 40 signals recorded; The observed overlap of ^{13}C NMR signals in the 14.1–37.1 ppm region is likely a result of the symmetry present in the cavitand scaffold. 44 non-equivalent carbons for the $C_{11}H_{23}$ groups in theory, 11 signals recorded.

MS (m/z, MALDI-TOF-MS): Calcd. for Chemical Formula: $[C_{236}H_{299}{}^{35}Cl_4O_{22}Si_2]^+$$[M+H]^+$: 3681.1, Found: 3681.1.

The synthesis of **S15**

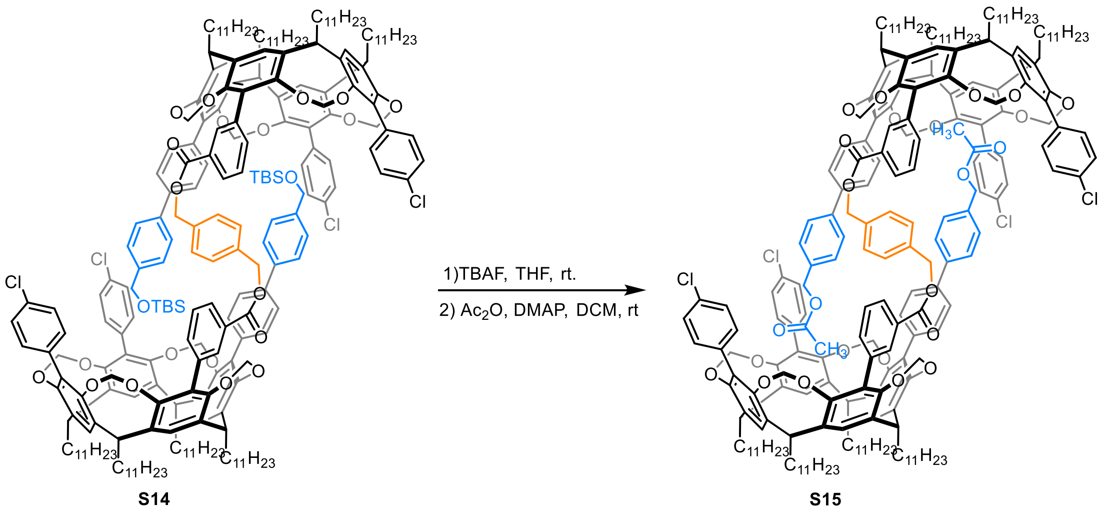

To a solution of **S14** (13 mg, 0.0035 mmol) in 1 mL dry THF was added TBAF (9 mg, 0.035 mmol, 10 equiv.). The reaction stirred at room temperature for 2 h. The reaction mixture was diluted with 10 mL ethyl acetate and washed with saturated NaCl. The combined organic layers were dried with Na_2SO_4 and concentrated *in vacuo* to afford the crude product. The crude product was dissolved into 1 mL dry DCM and added 4-dimethylaminopyridine (4 mg, 10 equiv.). Ac_2O (3.5 mg, 10 equiv.) was added into this reaction mixture and the solution was stirred at room temperature for 2 hours. The reaction mixture was diluted with 10 mL DCM and washed with saturated NaCl. The combined organic layers were dried with Na_2SO_4 and concentrated *in vacuo* to afford the crude product. Chromatography purification on PTLC using hexane/ethyl acetate (6:1) as the eluents gave the purified product **S15** (7.4 mg, 60%) as a colorless oil.

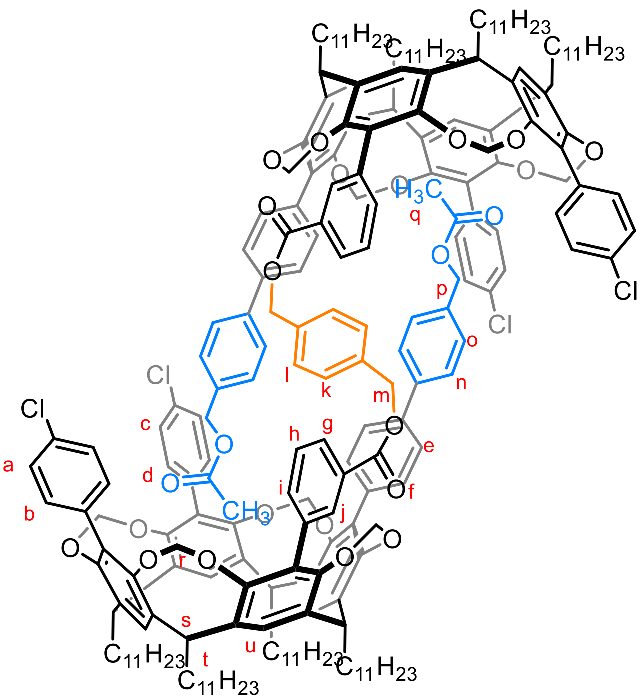

R_f = 0.6 (hexane/ethyl acetate = 6:1)

^{1}H NMR (500 MHz, $CDCl_3$) δ 7.98 (d, *J* = 7.4 Hz, 2H, 2×H_h), 7.69 (s, 2H, 2×H_j), 7.51 (s, 4H, 2×H_l and 2×H_k), 7.48 (d, *J* = 8.2 Hz, 4H, 2×H_g and 2×H_i), 7.45 (d, *J* = 8.1 Hz, 4H), 7.41-7.35 (m, 6H), 7.35 – 7.29 (m, 10H, H_u), 7.20 (d, *J* = 7.5 Hz, 4H), 7.15 (d, *J* = 8.3 Hz, 4H, 2×H_n), 7.05 (d, *J* = 8.4 Hz, 4H, 2×H_f), 6.94 (d, *J* = 8.0 Hz, 4H, 2×H_b), 6.89 (d, *J* = 7.0 Hz, 4H), 5.58 (d, *J* = 12.0 Hz, 2H, 2×H_p), 5.45 (d, *J* = 12.0 Hz, 2H, 2×H_p), 5.21 (d, *J* = 6.9 Hz, 2H, 2×H_m), 5.16-5.12 (m, 4H, 2×H_s), 5.07 (dd, *J* = 17.7, 9.7 Hz, 4H, 2×H_m), 4.91 – 4.82 (m, 10H, 2×H_r), 4.44 – 4.31 (m, 6H, 2×H_r), 4.09 (d, *J* = 7.0 Hz, 2H, 2×H_r), 2.41 – 2.30 (m, 16H, 2×H_t, 8×CH_2), 1.47 (dd, *J* = 14.0, 6.9 Hz, 32H, 2×H_t, 16×CH_2), 1.29 (s, 112H, 2×H_t, 56×CH_2), 0.89 (t, *J* = 5.7 Hz, 24H, 2×H_t, 8×CH_3), -1.03 (s, 6H, 2×H_q).

Note: 52 H (6.97 – 7.95 ppm) for aryl groups in compound **S15**; $H_{c,d}$, H_u, H_i and H_e resonate at 7.40 –7.30 ppm, but their signals could not be clearly distinguished.

^{13}C NMR (126 MHz, $CDCl_3$) δ 171.0, 166.3, 153.0, 152.8, 152.7_3, 152.6_9, 152.6_6, 152.6, 141.1, 138.8, 138.6, 138.4_5, 138.3_9, 138.3, 138.2, 136.8, 135.2, 134.9, 134.6, 133.3, 133.2, 132.6, 132.5, 131.3, 131.1, 130.3, 129.8_1, 129.7_7, 129.6, 129.4, 129.3, 129.2, 129.0, 128.3, 128.2, 128.1, 127.3, 126.7, 120.2, 120.0_0,

119.9_{8}, 119.9, 100.3, 100.1_{2}, 100.0_{6}, 99.8, 66.3, 66.2, 37.1, 32.0, 30.4, 30.3, 30.2, 29.9, 29.8_{0}, 29.7_{6}, 29.4, 28.0, 22.7, 14.1.

Note*:* The observed overlap of ^{13}C NMR signals in the 119.9–153.0 ppm region is likely a result of the symmetry presents in the cavitand scaffold. 48 non-equivalent aromatic carbons in theory, 40 signals recorded; The observed overlap of ^{13}C NMR signals in the 14.1–37.1 ppm region is likely a result of the symmetry present in the cavitand scaffold. 44 non-equivalent carbons for the $C_{11}H_{23}$ groups in theory, 12 signals recorded.

MS (m/z, MALDI-TOF-MS): Calcd. for Chemical Formula: $[C_{228}H_{275}{}^{35}Cl_4O_{24}]^+[M+H]^+$: 3536.9, Found: 3536.8.

The synthesis of **20**

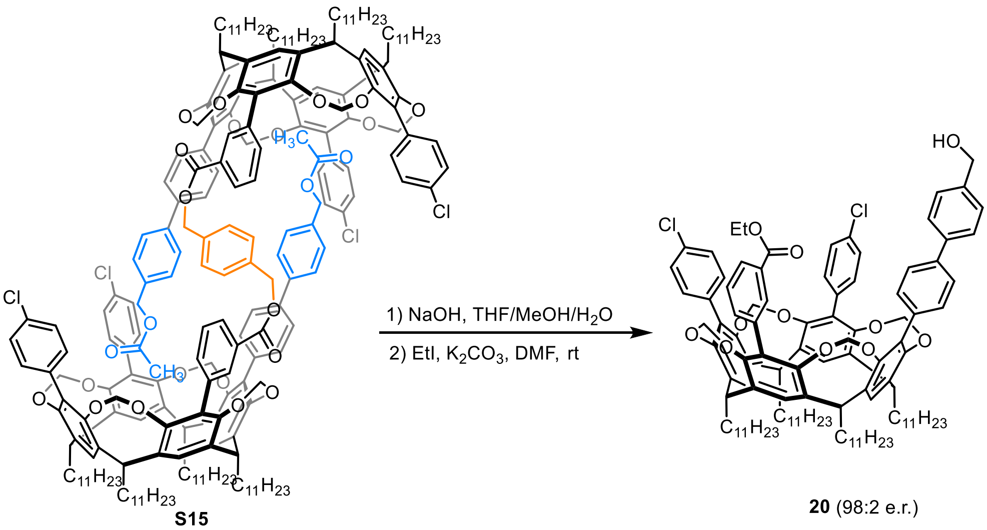

S15

20 (98:2 e.r.)

To a solution of **S15** (10 mg, 0.0028 mmol) in THF/MeOH (0.8 mL/0.2 mL) was added 0.2 mL 5N NaOH solution. The reaction stirred at 60 °C for 2 h. The reaction mixture was diluted with 10 mL ethyl acetate and washed with saturated NaCl. The combined organic layers were dried with Na_2SO_4 and concentrated *in vacuo* to afford the crude product. The crude product was dissolved into 1 mL DMF. K_2CO_3 (4 mg, 10 equiv.) and EtI (4 mg, 10 equiv.) were added into the solution. After 2 hours, the reaction mixture was diluted with 10 mL ethyl acetate and washed with saturated NaCl. The combined organic layers were dried with Na_2SO_4 and concentrated *in vacuo* to afford the crude product. Chromatography purification on PTLC using hexane/ethyl acetate (2:1) as the eluents gave the purified product **20** (8.2 mg, 86%) as a colorless oil. The enantiopurity of compound **20** (e.r. = 98:2) was confirmed by HPLC (see HPLC traces). Following the above procedure, compound **20** was used as the starting material for the synthesis of template **13**, which is presumed to retain the same enantiopurity.

The synthesis of chiral rotaxane **17**

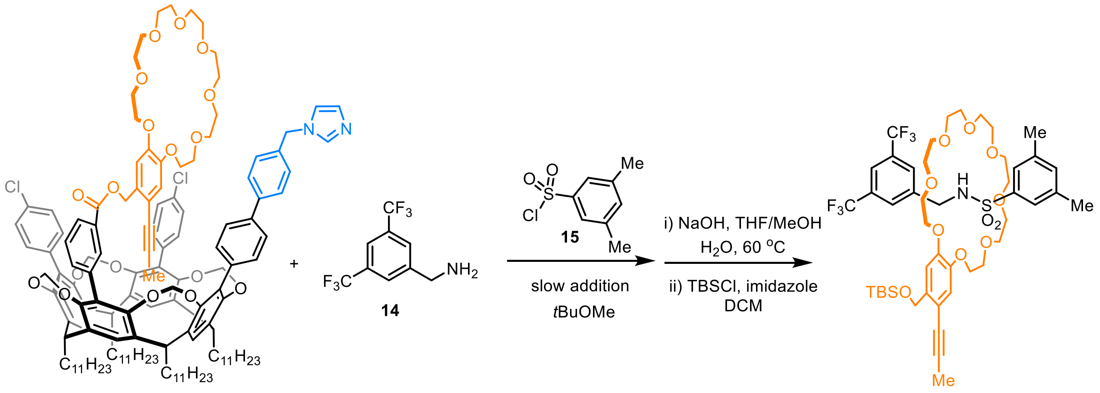

13 (e.r. = 98:2) **17**

According to the general procedure for slow addition, a reaction was conducted using **13** (5 mg, 0.0023 mmol, e.r. = 98:2), 3,5-bis(trifluoromethyl)benzylamine **14** (2.3 mg, 0.0092 mmol, 4 equiv.) and sulfonyl chloride **15** (0.9 mg, 0.0046 mmol, 2 equiv.) in 0.4 mL *t*BuOMe. The reaction afforded the product **17** (1.7 mg, 75% yield, e.r. = 93:7) as colorless oil.

$[\alpha]_D^{25}$ = – 24 (*c* 0.1 $CHCl_3$) for e.r. = 93:7

HPLC analysis of this compound **17**: Daicel Chiralpak IF, hexane/*iso*-propanol = 95:5, 1.0 mL/min, temperature = 40 °C, λ = 254 nm, retention time: 19.4 min (major) and 21.0 min (minor).

Preparation of alkyne molecules for azide-alkyne cycloaddition

The synthesis of **30**

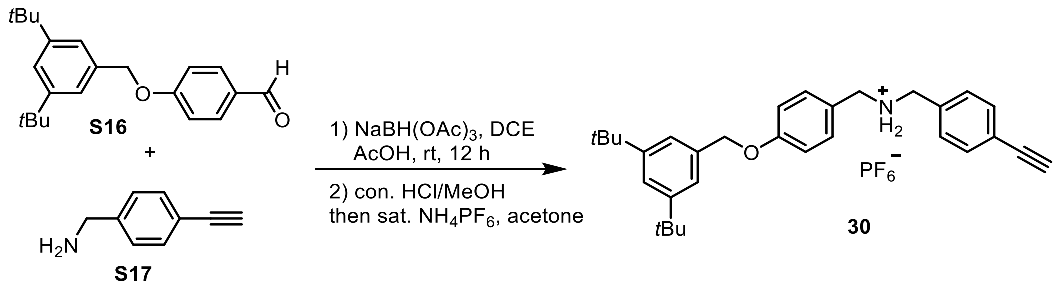

S16 synthesis from reported procedure[5]. To a solution of **S16** (32 mg, 0.1 mmol) and **S17** (13 mg, 0.1 mmol) in 1 mL DCE was added 0.6 mL AcOH. Sodium triacetoxyborohydride (42 mg, 0.2 mmol) was added into this mixture. The reaction stirred at room temperature for 12 h. The reaction mixture was diluted with 10 mL ethyl acetate and washed with saturated NaCl. The combined organic layers were dried with Na_2SO_4 and concentrated *in vacuo* to afford the crude product. Chromatography purification on silica gel using hexane/ethyl acetate (3:1) as the eluents gave the purified product as a colorless oil. The purified product was dissolved into 2 mL methanol and added conc. HCl to adjust pH to 2. The solvent was removed under reduced pressure. The residue was added 2 mL acetone and saturated NH_4PF_6 until the reaction mixture became a clean solution. The clean solution was removed under reduced pressure and washed the residue with water to give the desired product as light-brown solid **30** (36 mg, 62% for three steps).

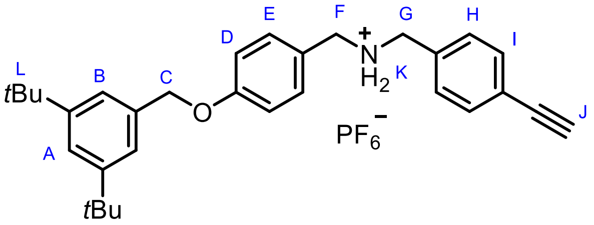

R$_f$ = 0.6 (100% ethyl acetate).

^{1}H NMR (400 MHz, DMSO-d6) δ 9.10 (s, 2H, H$_K$), 7.56 (d, *J* = 8.3 Hz, 2H, H$_I$), 7.49 (d, *J* = 8.3 Hz, 2H, H$_H$), 7.41 (d, *J* = 8.7 Hz, 2H, H$_E$), 7.36 (t, *J* = 1.8 Hz, 1H, H$_A$), 7.27 (d, *J* = 1.7 Hz, 2H, H$_B$), 7.10 (d, *J* = 8.7 Hz, 2H,

H_D), 5.09 (s, 2H, H_C), 4.28 (s, 1H, H_J), 4.14 (d, *J* = 19.5 Hz, 4H, H_F and H_G), 1.29 (s, 18H, H_L, 2×*t*Bu).

^{13}C NMR (126 MHz, DMSO-d6) δ 159.5, 150.9, 136.3, 133.1, 132.4, 132.1, 130.7, 124.1, 122.8, 122.5, 122.0, 115.4, 83.4, 82.3, 70.5, 50.2, 49.9, 35.0, 31.7.

HRMS (m/z, APCI): Calcd. for Chemical Formula: $[C_{31}H_{38}NO]^+[M-PF_6]^+$: 440.2948, Found: 440.2955.

The synthesis of **32**

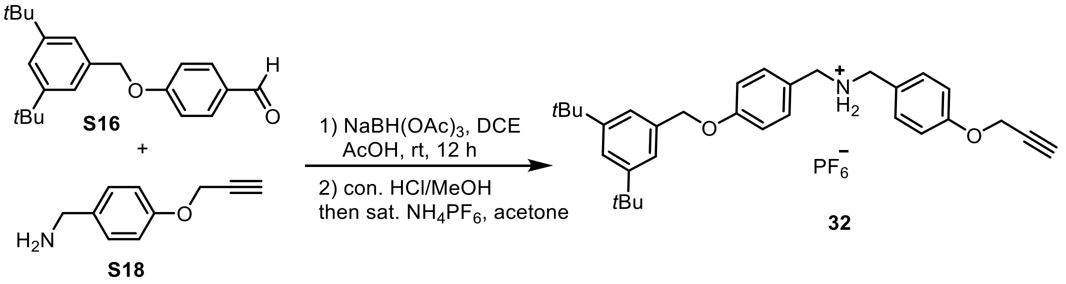

Following the general procedure, **S16** (32 mg, 0.1 mmol) and **S18** (17 mg, 0.1 mmol) were used to prepare product **32** as a light-brown solid (46 mg, 75% over three steps).

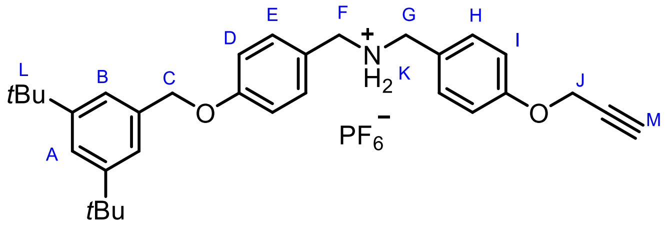

R_f = 0.7 (hexane/ethyl acetate = 1:2).

^{1}H NMR (500 MHz, $CDCl_3$) δ 7.36 (s, 1H, H_A), 7.24 – 7.21 (m, 6H, $H_{B, H, E}$), 6.92 (dd, *J* = 16.3, 8.4 Hz, 4H, H_E and H_I), 4.97 (s, 2H, H_C), 4.64 (d, *J* = 2.1 Hz, 2H, H_J), 3.70 (s, 4H, H_F and H_G), 2.47 (s, 1H, H_M), 1.29 (s, 18H, H_L, 2×*t*Bu).

^{13}C NMR (126 MHz, $CDCl_3$) δ 158.2, 156.7, 151.0, 136.0, 133.3, 132.4, 129.4, 122.2, 114.9, 114.8, 78.7, 75.5, 71.0, 55.9, 52.4, 52.3, 34.9, 31.5.

Note: The observed overlap of ^{13}C NMR signals stacking in the 114.8 – 158.2 ppm region is the phenyl groups. 12 non-equivalent aromatic carbons in theory, 10 signals recorded.

HRMS (m/z, APCI): Calcd. for Chemical Formula: $[C_{32}H_{40}NO_2]^+[M-PF_6]^+$: 470.3054, Found: 470.3043.

Rotaxane synthesis through azide-alkyne cycloaddition

The synthesis of **31**

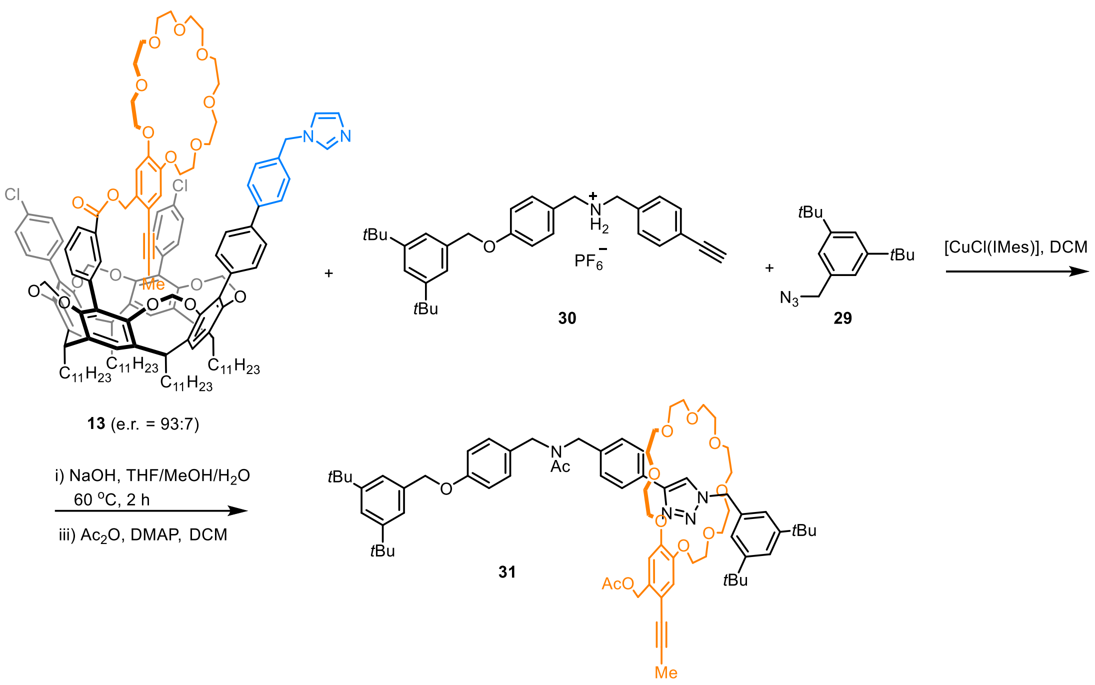

Under N_2 atmosphere in the glovebox, a 2 mL flask equipped with a magnetic stirring bar were added **13** (5 mg, 0.0023 mmol), alkyne **30** (1.3 mg, 0.0023 mmol, 1 equiv.) and azide **29** (0.5 mg, 0.0023 mmol) in 0.2 mL DCM. [CuCl(IMes)] (30 mol%, 0.3 mg) were added into the reaction mixture. The reaction mixture was then stirred at room temperature for 24 hours. The solution was diluted with 20 mL ethyl acetate and washed with 10 mL saturated NaH_2PO_4 and saturated NaCl. The organic phase was concentrated under reduced pressure to give a light-yellow oil as crude product. The crude product was hydrolysis under the condition of 5 M NaOH in THF/MeOH/H_2O (2 mL/0.5 mL/0.5 mL, T = 60 °C) for 2 h. Then the solution was concentrated under concentrated *in vacuo* to remove solvent. The mixture was dissolved in 20 mL ethyl acetate and washed with saturated NaCl. The organic layer was dried with Na_2SO_4 and concentrated *in vacuo* to afford the crude product. The crude product was used into next step without further purification. The crude product was dissolved in 2 mL dry DCM. Ac_2O (2.4 mg, 0.023 mmol, 10 equiv.) and DAMP (2.8 mg, 0.023 mmol, 10 equiv.) were added into the reaction mixture

subsequently. The reaction mixture was stirred at room temperature for 2 h. The reaction mixture was diluted with 10 mL DCM and washed with saturated NaH_2PO_4 and saturated NaOH. The combined organic layers were dried with Na_2SO_4 and concentrated *in vacuo* to afford the crude product. Chromatography purification on PTLC using hexane/acetone = 3:1 to 2:1 as the eluents gave the purified product **31** (1.2 mg, 42% yield).

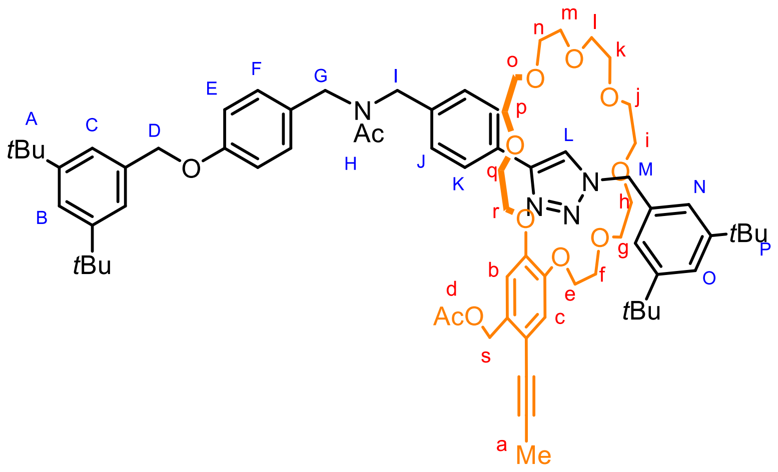

R_f = 0.3 (hexane/acetone = 2:1)

$[\alpha]_D^{25}$ = 18 (*c* 0.1 $CHCl_3$) for e.r. = 79.5:20.5

^{1}H NMR (500 MHz, DMSO-d6, 373K) δ 8.42 (s, 1H, H_L), 7.78 (d, *J* = 7.7 Hz, 2H, H_K), 7.50-7.46 (m, 2H, H_N), 7.44 – 7.37 (m, 3H, H_J and H_O), 7.31 (s, 1H, H_B), 7.26 – 7.20 (m, 4H, H_F and H_C), 7.17 – 7.06 (m, 1H, H_b), 6.99 – 6.94 (m, 2H, H_E), 6.91 – 6.85 (m, 1H, H_c), 5.67 (s, 1H, H_S), 5.60 (s, 2H, H_M), 5.52 – 5.44 (m, 2H, H_D), 5.09 – 5.06 (m, 1H, H_S), 4.50 – 4.42 (m, 4H, H_G and H_I), 4.13 – 4.05 (m, 2H, H_r), 4.04 – 3.95 (m, 2H, H_e), 3.64 (s, 4H, $H_{f\text{-}q}$), 3.48 – 3.28 (m, 18H, $H_{f\text{-}q}$), 3.25 – 3.18 (m, 2H, $H_{f\text{-}q}$), 2.11 (s, 3H, H_d), 2.04-2.00 (m, 6H, H_H and H_a), 1.30 (s, 18H, H_A), 1.28 (s, 18H, H_P).

^{13}C NMR (126 MHz, DMSO-d6) δ 170.7, 165.9, 151.9, 151.3, 150.8, 146.5, 137.5, 136.6, 136.5, 135.7, 129.6, 128.7, 128.5, 127.6, 126.0, 125.7, 125.3, 122.5, 122.5, 122.2, 121.9, 118.9$_5$, 118.8$_7$, 115.5, 115.2, 86.5, 79.5, 73.1, 70.6, 70.5, 70.4, 70.2, 69.9, 69.3, 69.2, 68.8, 55.4, 54.0, 50.7, 47.6, 35.0, 31.7, 31.6,

29.5, 29.2, 25.6, 22.6, 22.0, 14.4, 4.3.

Note: The observed overlap of ^{13}C NMR signals stacking in the 68.8 – 73.1 ppm region is the glycol groups in crown ether. 14 non-equivalent carbons in theory, 9 signals recorded.

HPLC analysis of this compound: Daicel Chiralpak IB, hexane/*iso*-propanol = 90: 10, 1.0 mL/min, temperature = 40 °C, λ = 220 nm, retention time: 46.5 min (minor) and 50.6 min (major).

HRMS (m/z, APCI): Calcd. for Chemical Formula: $[C_{74}H_{101}N_4O_{12}]^+[M+H]^+$: 1237.7411, Found: 1237.7428.

The synthesis of **33**

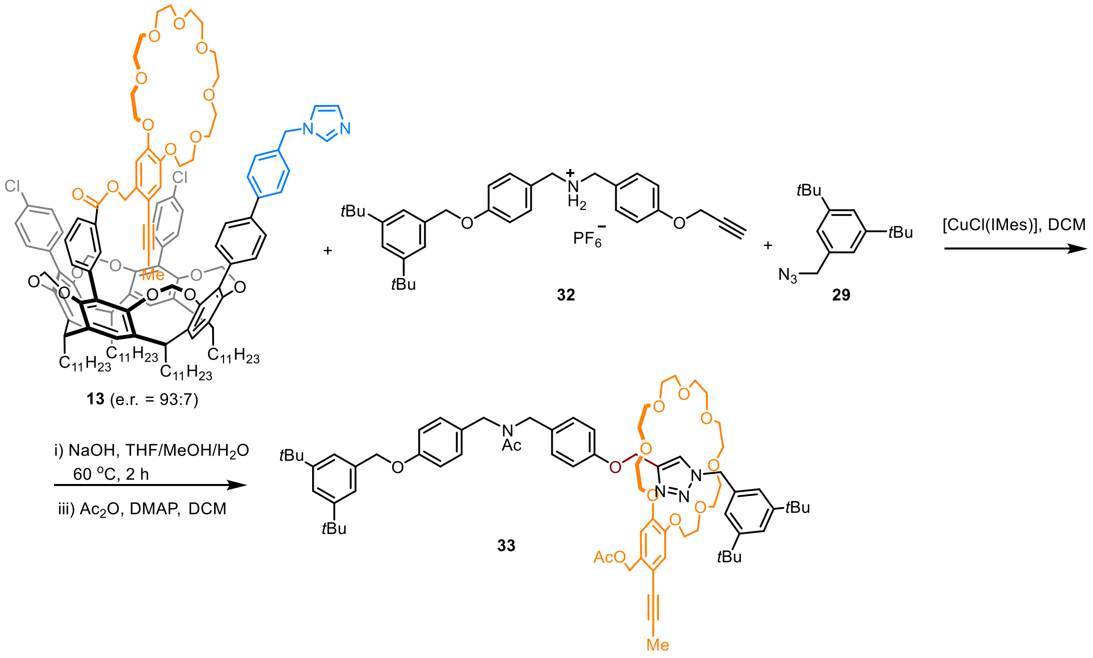

According to the general procedure for click reaction, a reaction was conducted using **13** (5 mg, 0.0023 mmol, e.r. = 93:7), alkyne **32** (1.4 mg, 0.0023 mmol), azide **29** (0.5 mg, 0.0023 mmol) and [CuCl(IMes)] (30 mol%, 0.3 mg) in 0.2 mL DCM. The reaction afforded the product **33** as colorless oil (1.5 mg, yield 51%).

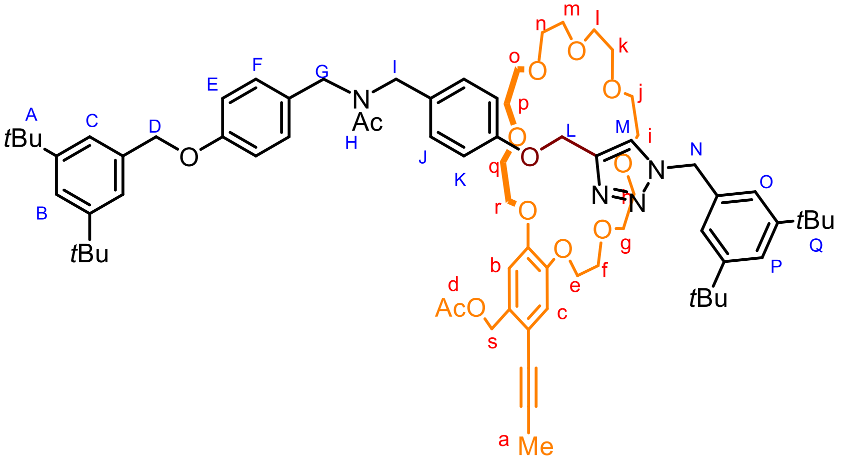

R_f = 0.4 (acetone/hexane = 1:2) (two inseparable isomers, the ratio of two isomers is 1: 0.6).

$[\alpha]_D^{25}$ = 32 (*c* 0.1 $CHCl_3$) for **33** er = 80:20

^{1}H NMR (500 MHz, DMSO-d6, 373K, major isomer = 1H) δ 8.22 (s, 1H, H_M), 7.48 (d, *J* = 5.6 Hz, 1H, H_F),

7.44 (d, *J* = 8.0 Hz, 1H, H_K), 7.38 (d, *J* = 7.1 Hz, 2H, H_J), 7.26 (s, 1H, H_P), 7.21 (s, 2H, H_O), 7.14 (s, 2H, H_C), 7.12 – 7.05 (m, 3H, H_c and H_B), 7.02 – 6.93 (m, 3H, H_F and H_b), 6.93 – 6.87 (m, 2H, H_E), 5.57 (s, 1H, H_M), 5.47 (d, *J* = 5.6 Hz, 3H, H_M and H_s), 5.16 (s, 1H, H_D), 5.07 (d, *J* = 6.3 Hz, 2H, H_I), 5.02 (s, 1H, H_D), 4.42 – 4.30 (m, 4H, H_G and H_r), 4.05 – 3.98 (m, 3H, H_{f-q}), 3.73 – 3.58 (m, 4H, H_{f-q}), 3.48 – 3.19 (m, 20H, H_{f-q}), 2.15 – 1.98 (m, 9H, H_H and H_d and H_a), 1.35 – 1.26 (m, 36H, H_A and H_Q).

^{1}H NMR (500 MHz, DMSO-d6, 373K, minor isomer = 0.6 H compare to major isomer) δ 8.11 (s, 0.6x1H, H_M), 7.38 (d, *J* = 7.1 Hz, 0.6x2H, H_F), 7.31 (s, 0.6x1H, H_J), 7.26 (s, 0.6x3H, H_P), 7.21 (s, 0.6x1H, H_O), 7.14 (s, 0.6x2H, H_C), 7.12–7.05 (m, 0.6x3H, H_B and H_c), 7.02 – 6.93 (m, 0.6x4H, H_F and H_b), 6.93 – 6.87 (m, 0.6x1H, H_E), 6.87 – 6.83 (m, 0.6x1H, H_E), 5.59 (d, *J* = 4.1 Hz, 0.6x3H, H_M and H_s), 5.07 (d, *J* = 6.3 Hz, 0.6x2H, H_D), 5.02 (s, 0.6x1H, H_D), 4.42 – 4.30 (m, 0.6x4H, H_G and H_r), 4.11 – 4.07 (m, 0.6x2H, H_{f-q}), 4.05 – 3.98 (m, 0.6x3H, H_{f-q}), 3.73 – 3.58 (m, 0.6x4H, H_{f-q}), 3.48 – 3.19 (m, 0.6x20H, H_{f-q}), 2.15 – 1.98 (m, 0.6x9H, H_H and H_d and H_a), 1.35 – 1.26 (m, 0.6x36H, H_A and H_Q).

^{13}C NMR (126 MHz, DMSO-d6, two isomers) δ 170.7, 170.6, 170.4$_1$, 170.3$_5$, 159.2, 159.1, 158.6, 158.5, 158.2, 158.1, 157.7, 157.6, 151.2, 151.1, 150.8, 149.9, 149.8, 149.0, 149.0, 148.1, 147.9, 147.6$_9$, 147.6$_7$, 144.9$_9$, 144.9$_6$, 143.5, 143.4, 137.8, 137.7, 136.6, 136.5, 135.7, 135.5, 129.6, 128.8, 128.6, 128.5, 128.2, 128.0, 127.5, 127.2, 127.0, 126.9, 125.8, 125.7, 125.0, 124.3, 124.2, 123.0, 122.5, 122.4, 122.2, 121.9, 116.8, 116.5, 116.2, 115.9, 115.6, 115.4, 115.3, 115.1, 115.0, 114.8, 114.0, 113.2, 89.5$_4$, 89.4$_9$, 70.5, 69.8, 69.6$_1$, 69.5$_8$, 69.5, 69.3, 69.2, 69.0, 65.8, 65.7, 64.5, 64.4, 61.5, 55.4, 53.8, 50.7, 50.4, 50.1, 47.2, 47.1, 35.6, 35.0, 34.9$_4$, 34.8$_7$, 31.8, 31.7, 31.6, 29.8, 29.5, 29.2, 29.0, 22.6, 22.1, 22.0, 21.1, 21.0, 14.4, 4.4.

Note: The observed overlap of ^{13}C NMR signals stacking in the 65.7 – 70.5 ppm region is the glycol groups in crown ether. 14 non-equivalent carbons in theory, 10 signals recorded.

HPLC analysis of this compound **33**: Daicel Chiralpak IF, hexane/*iso*-propanol = 70: 30, 1.0 mL/min,

temperature = 40 °C, λ = 254 nm, retention time: 13.2 min (minor) and 15.1 min (major).

HRMS (m/z, APCI): Calcd. for Chemical Formula:[$C_{75}H_{103}N_4O_{13}$]$^+$[M+H]$^+$: 1267.7516, Found: 1267.7489.

The synthesis of **33** with **12**

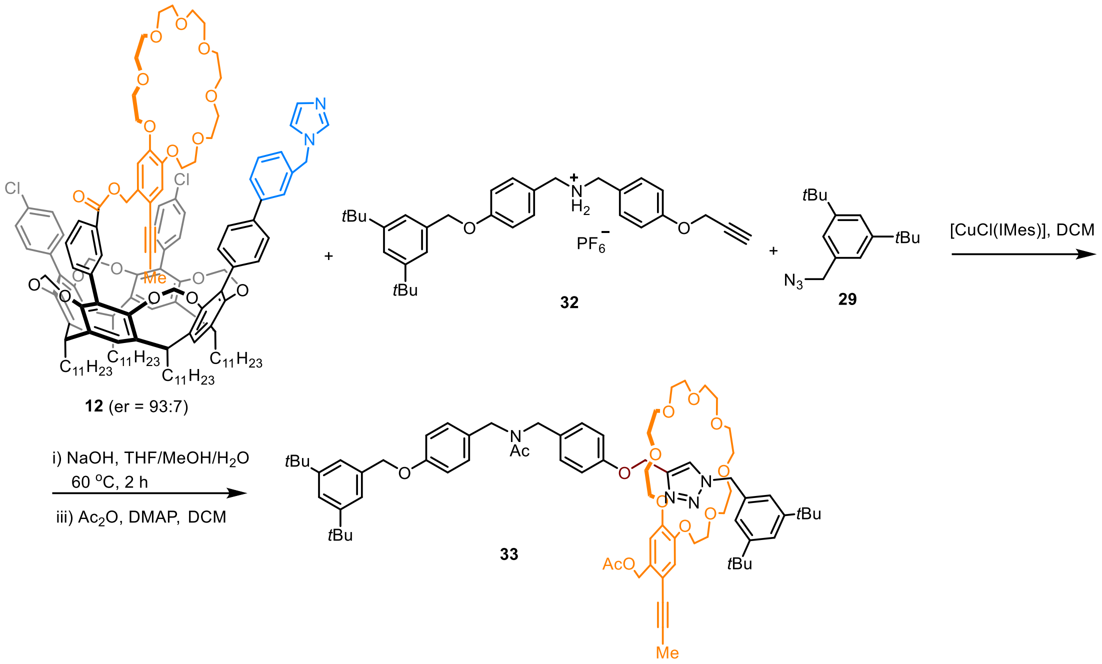

According to the general procedure for click reaction, a reaction was conducted using **12** (5 mg, 0.0023 mmol, e.r. = 93:7), alkyne **32** (1.4 mg, 0.0023 mmol), azide **29** (0.5 mg, 0.0023 mmol) and [CuCl(IMes)] (30 mol%, 0.3 mg) in 0.2 mL DCM. The reaction afforded the product **33** as colorless oil (1.0 mg, yield 37%) and HPLC analysis of **33** with e.r. = 81:19.

References

1. I. Neira, R. J. H. Hafkamp, S. G. Lemay, W. S. Y. Wong, R. Eelkema, *Org. Biomol. Chem.* **2020**, *18*, 5228–5233.
2. M. Li, C. K. S. Ho, I. K. W. On, V. Gandon, Y. Zhu, *Chem* **2024**, *10*, 3323–3341.
3. M. Wollenburg, D. Moock, F. Glorius, *Angew. Chem. Int. Ed.* **2019**, *58*, 6549–6553.
4. Z. Wang, H. Zhang, X. Liu, J. Wu, S. S. LaMarche, D. C. Johnson, S. K. Wang, Y. Wang, S. J. Elmore, T. K. Sawyer, *Arch. Pharm.* **2017**, *350*, e1600251.
5. Z. Luo, W. Zhang, H. Wang, Y. Liu, W. Yang, Y. Chen, *Angew. Chem. Int. Ed.* **2023**, *62*, e202306489.

NMR spectra

2, ^{1}H NMR ($CDCl_3$, 400 MHz)

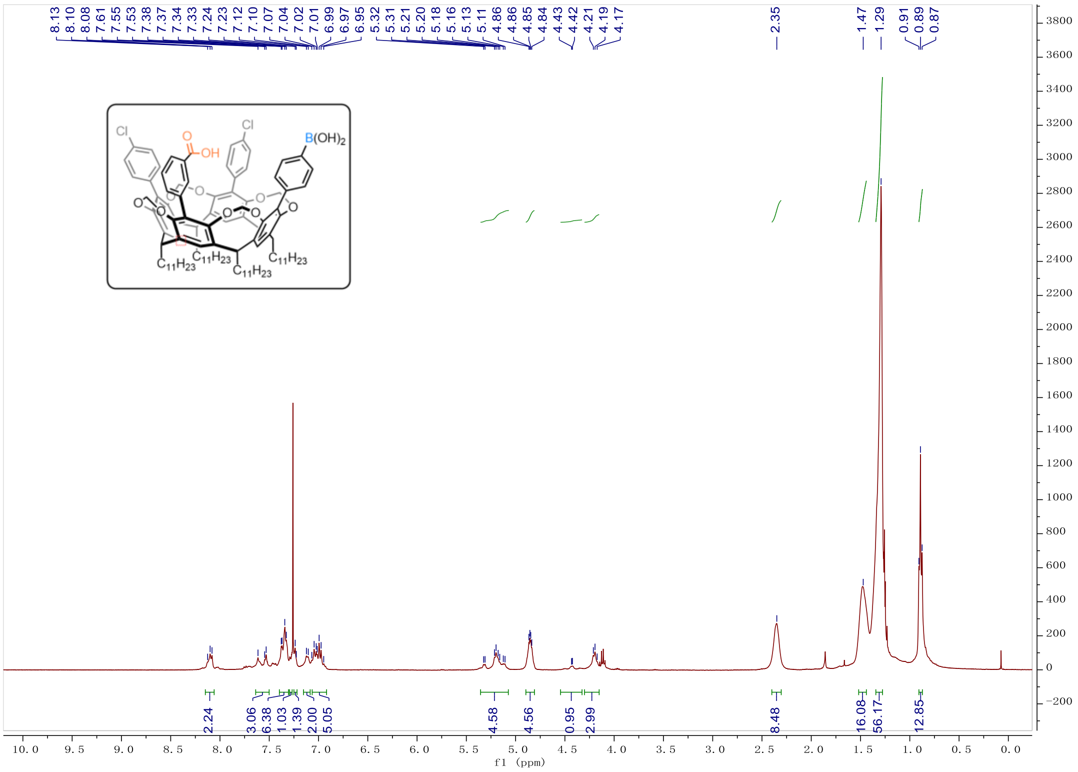

2, ^{13}C NMR ($CDCl_3$, 126 MHz)

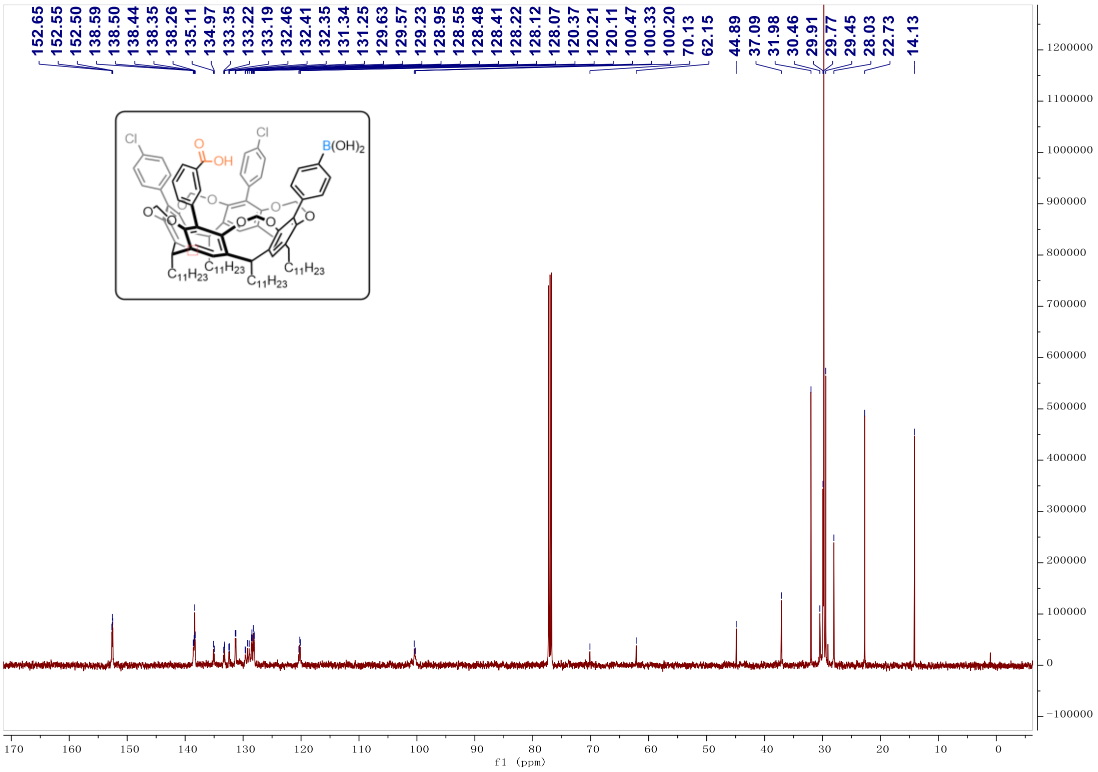

S1, ^{1}H NMR ($CDCl_3$, 400M)

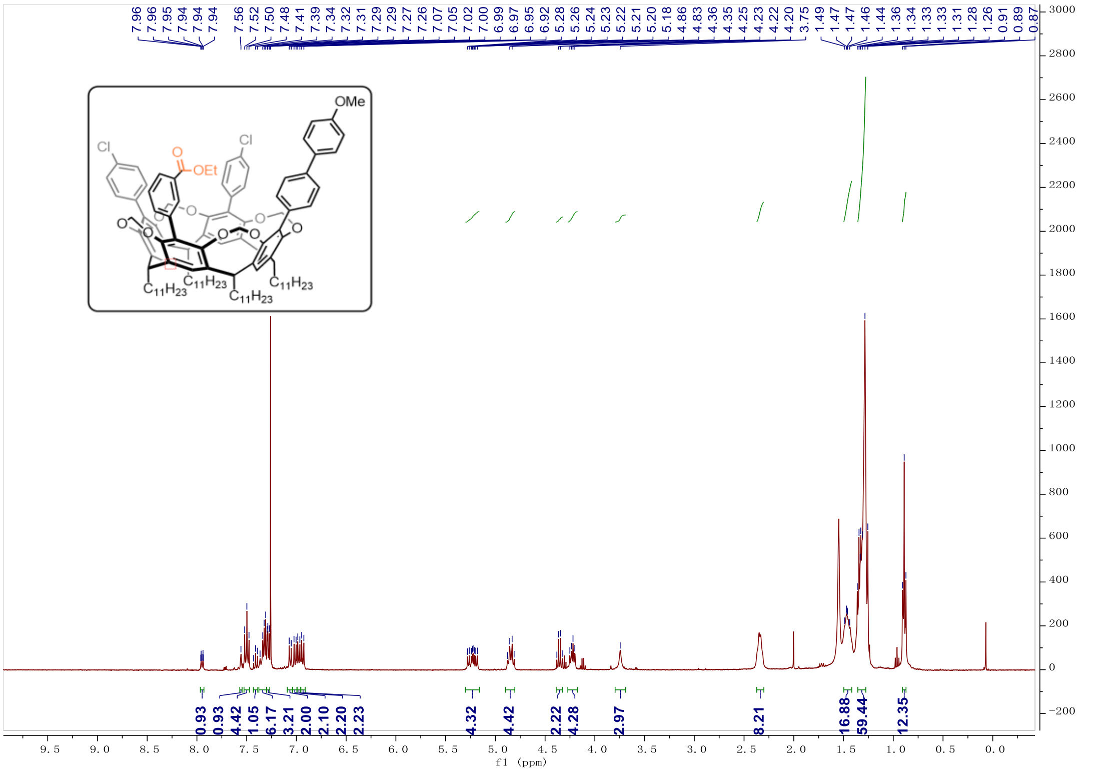

S1, ^{13}C NMR ($CDCl_3$, 126 MHz)

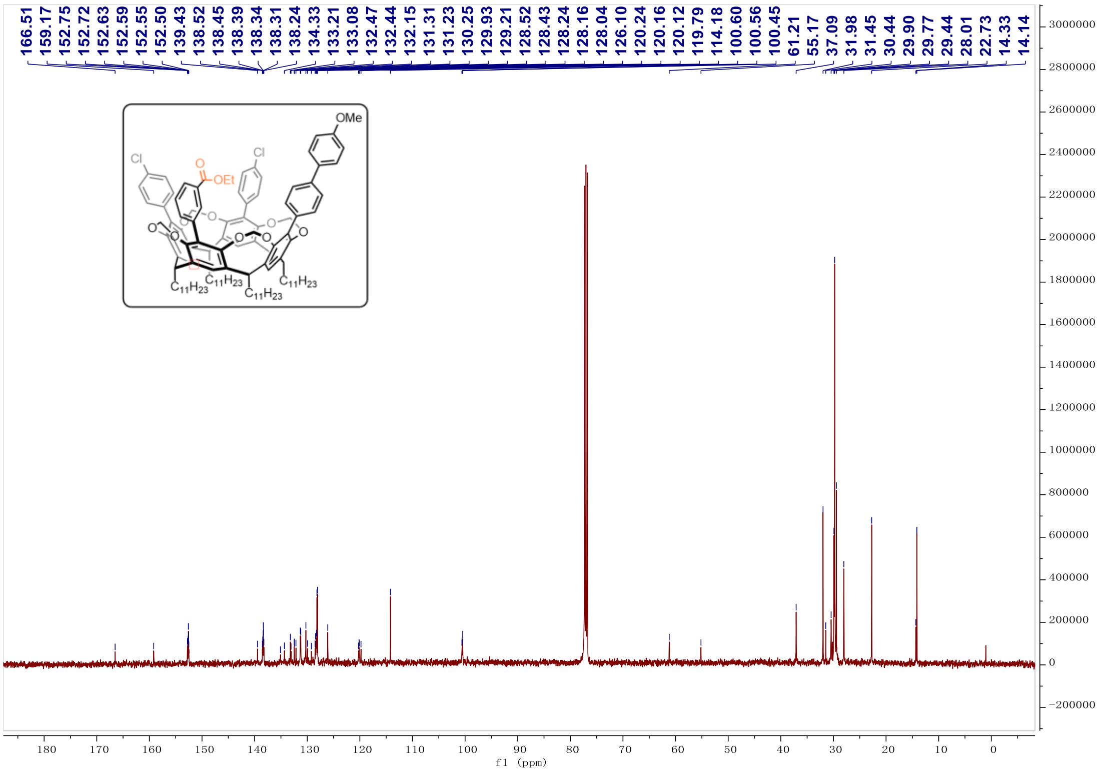

3, ^{1}H NMR ($CDCl_3$, 500 MHz)

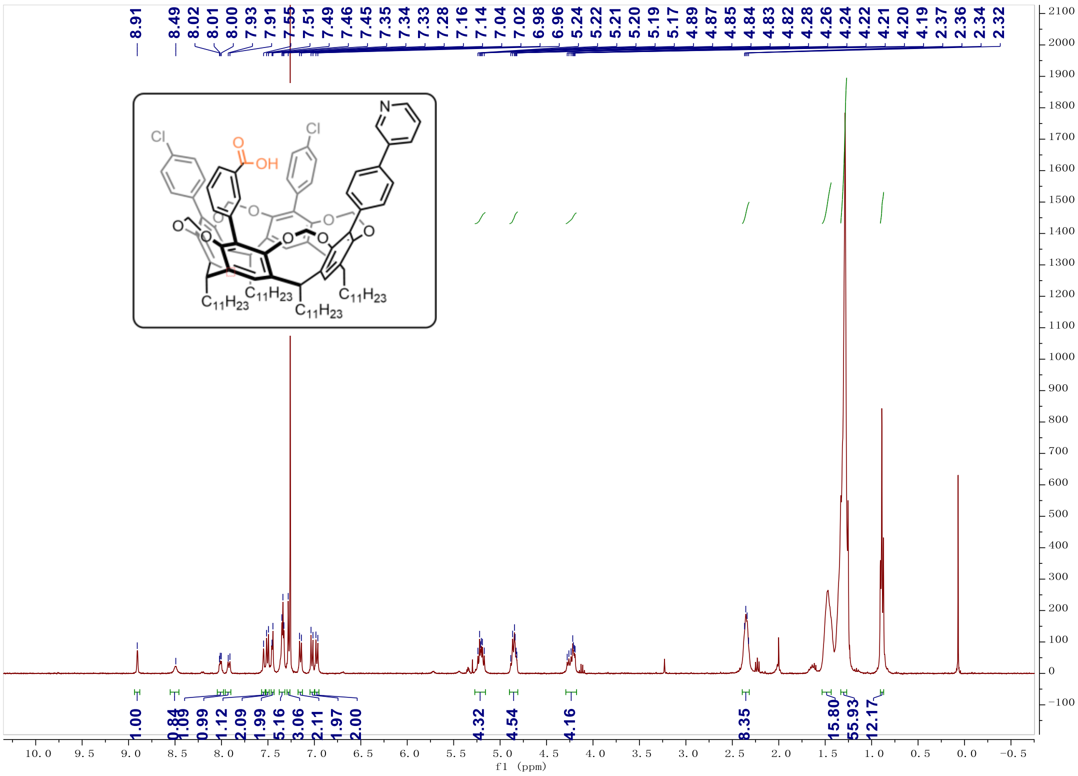

3, ^{13}C NMR ($CDCl_3$, 126 MHz)

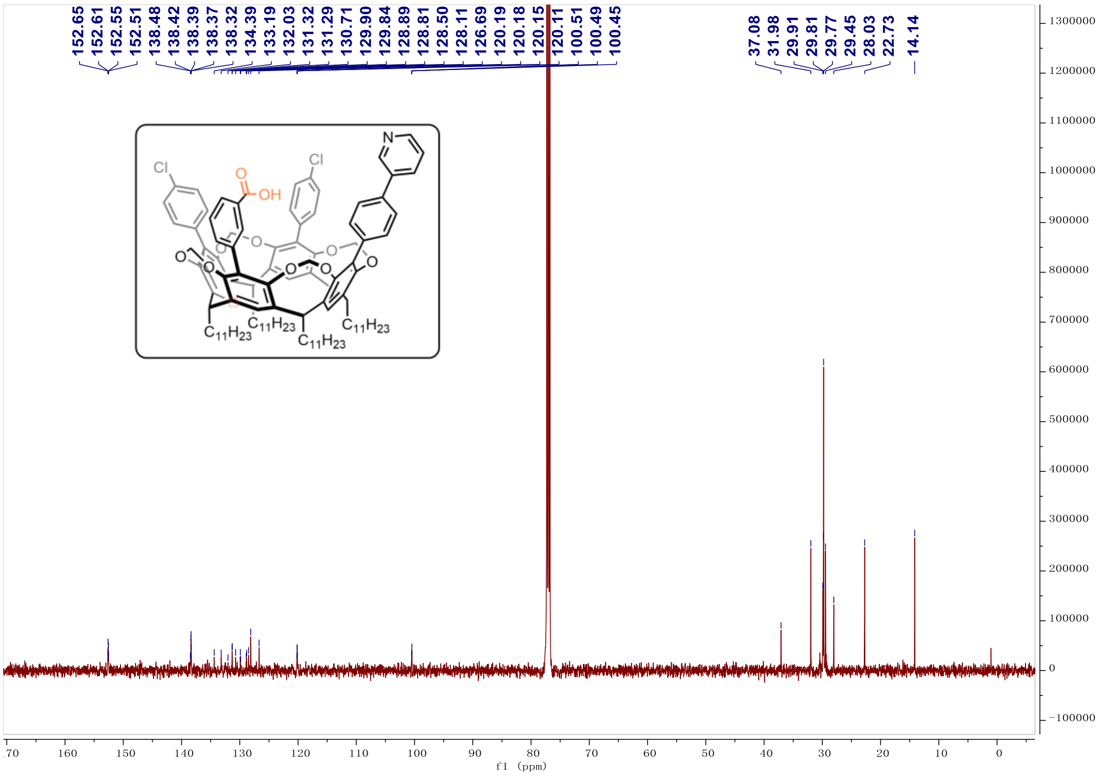

4 (ethyl ester), ^{1}H NMR ($CDCl_3$, 400 MHz)

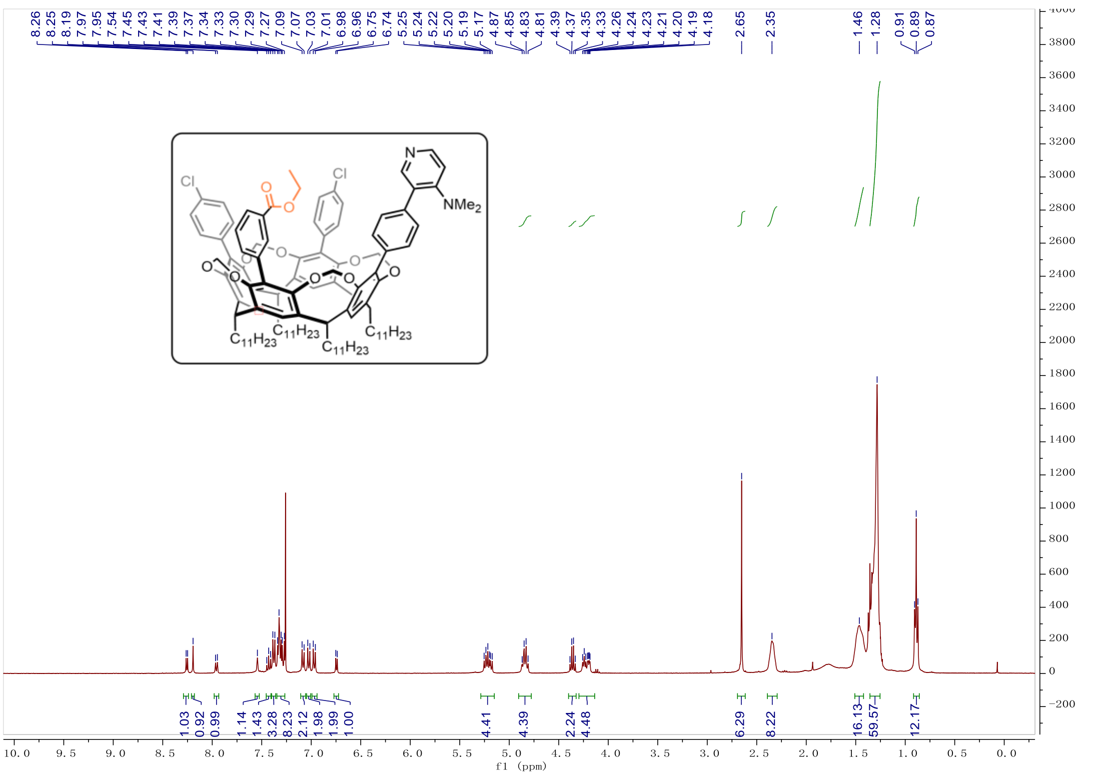

4 (ethyl ester), ^{13}C NMR ($CDCl_3$, 126 MHz)

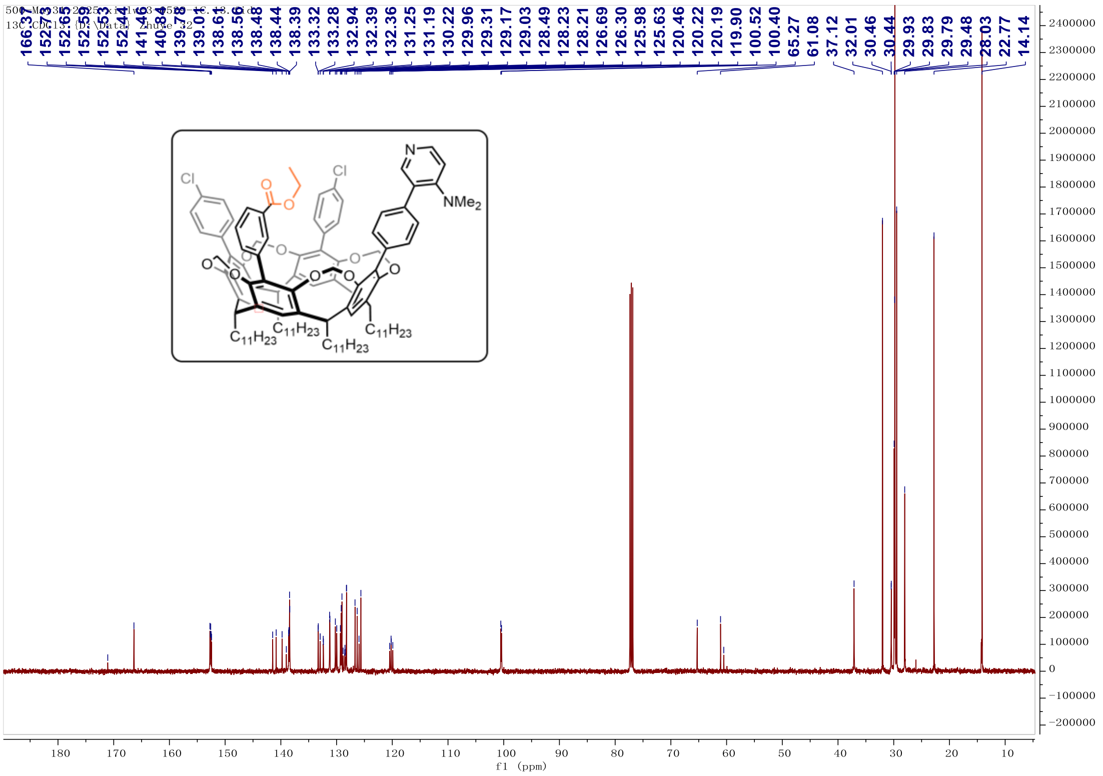

5, (ethyl ester) ^{1}H NMR ($CDCl_3$, 500 MHz)

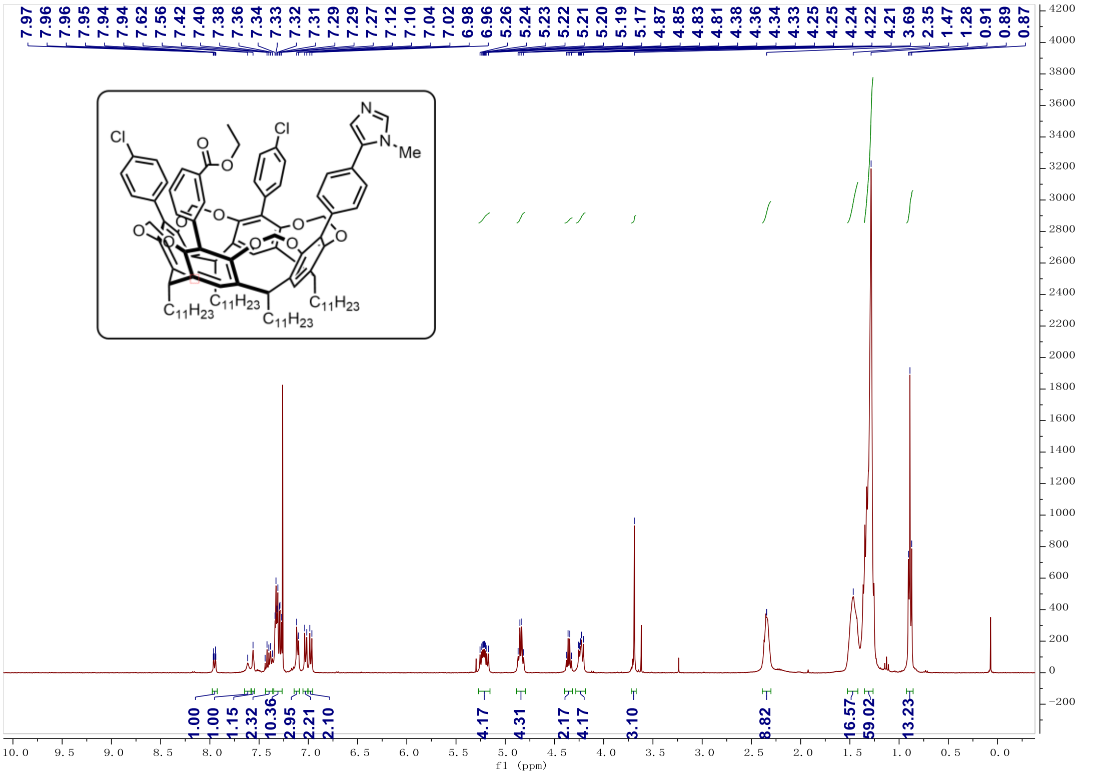

5, (ethyl ester), ^{13}C NMR ($CDCl_3$, 126 MHz)

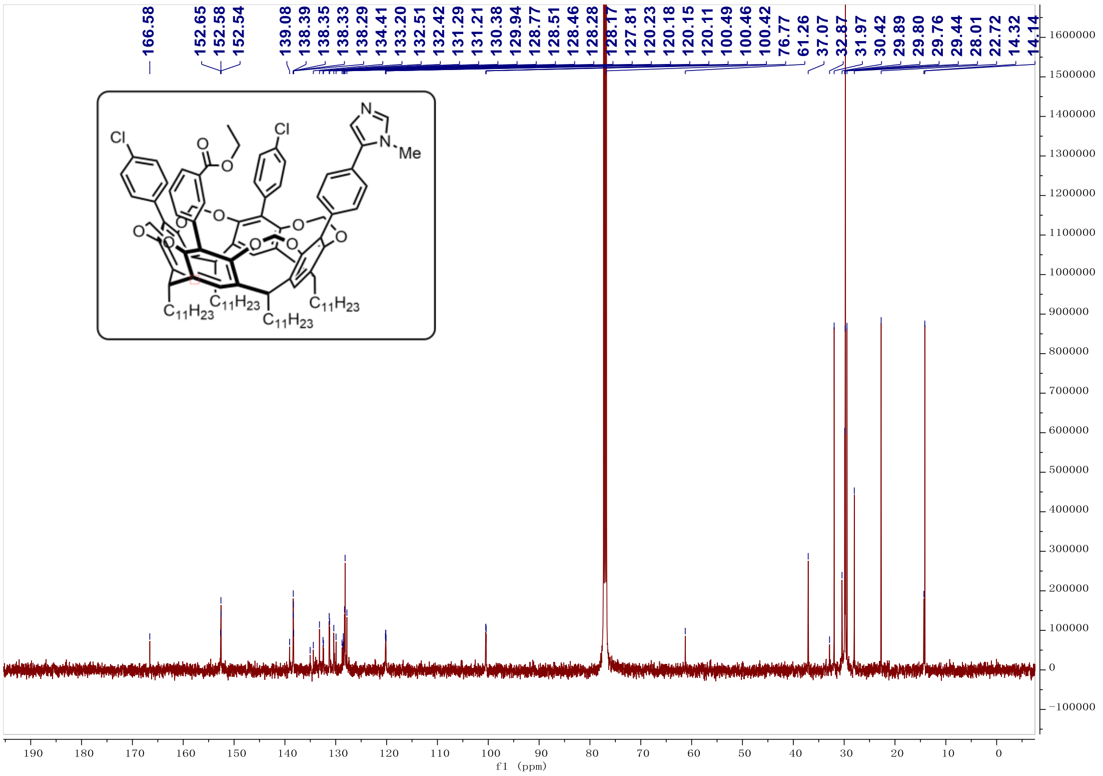

6, ^{1}H NMR (CDCl$_3$, 400 MHz)

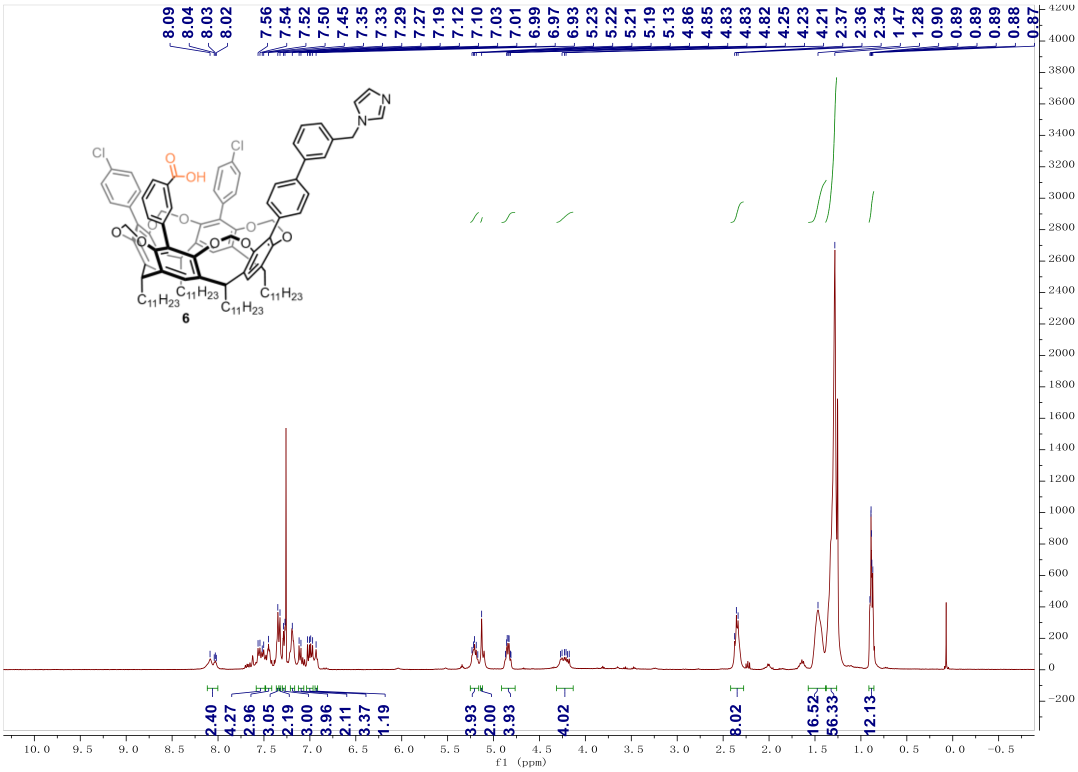

6, ^{13}C NMR (CDCl$_3$, 126 MHz)

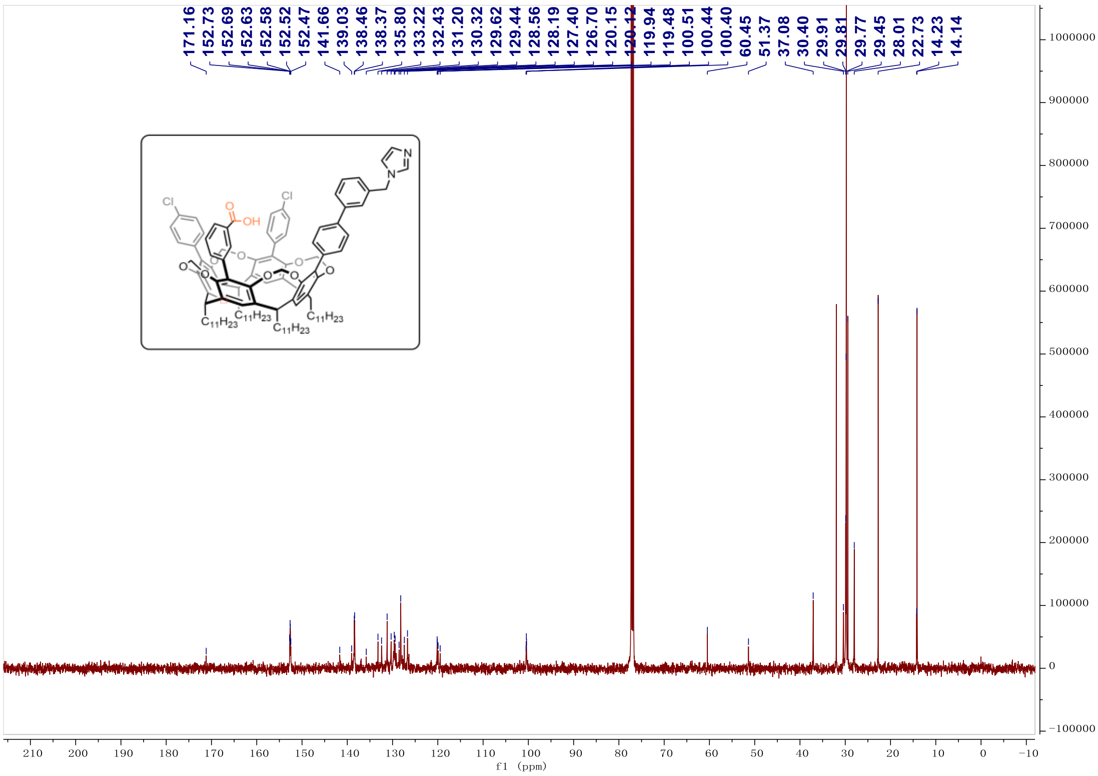

7, ^{1}H NMR ($CDCl_3$, 500 MHz)

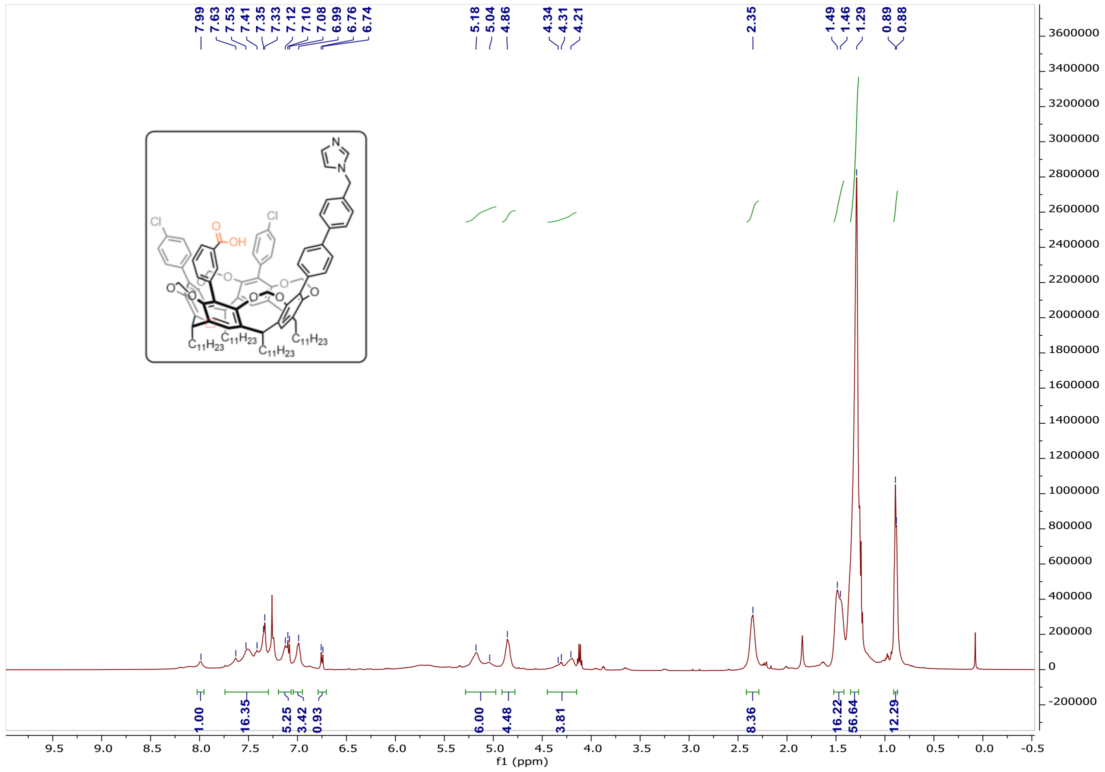

7, ^{13}C NMR ($CDCl_3$, 126 MHz)

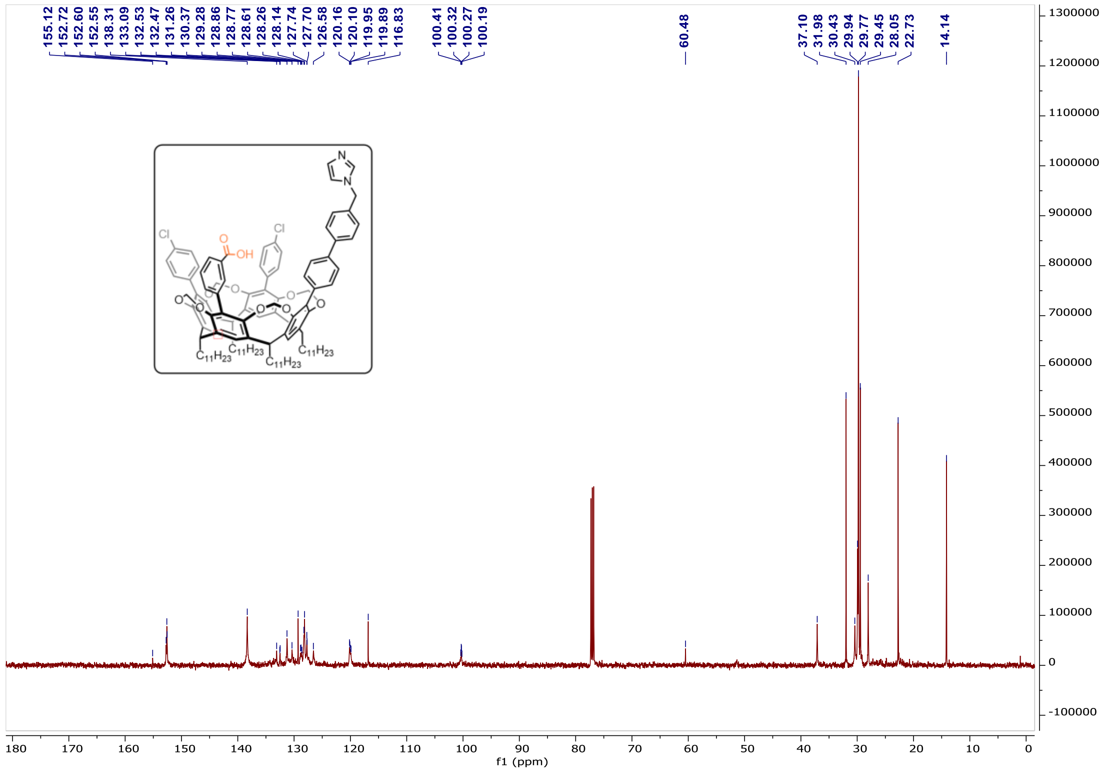

S4, ^{1}H NMR ($CDCl_3$, 500 MHz)

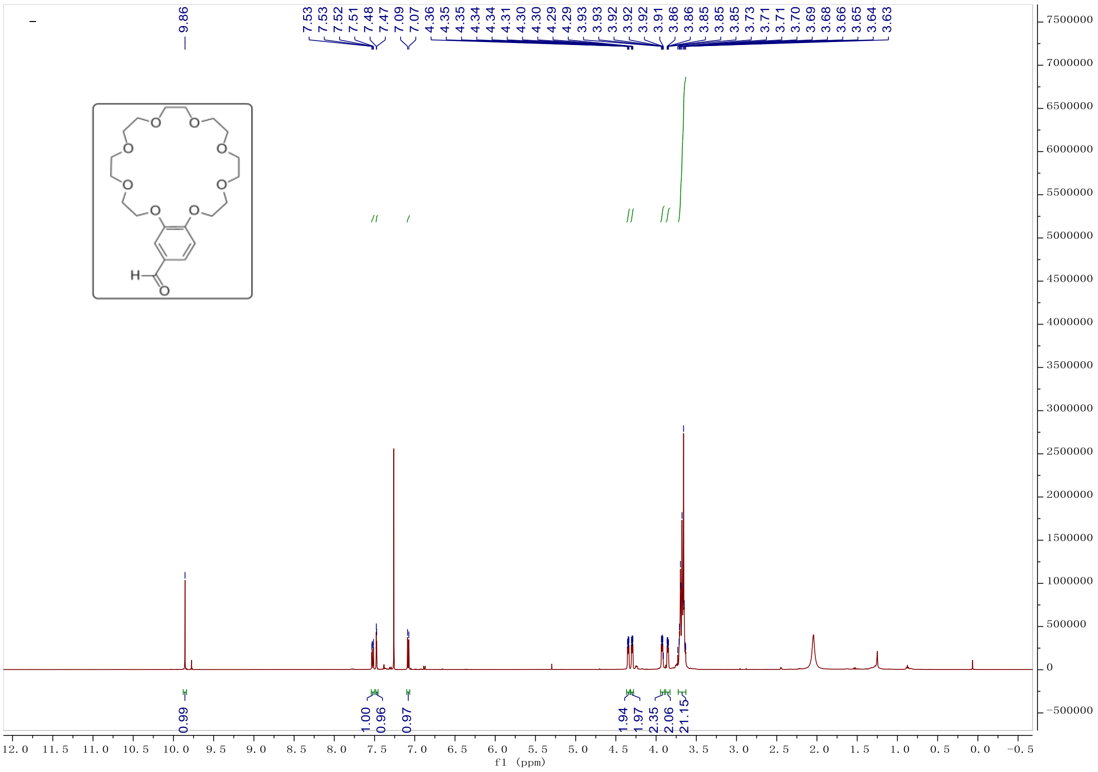

S4, ^{13}C NMR ($CDCl_3$, 126 MHz)

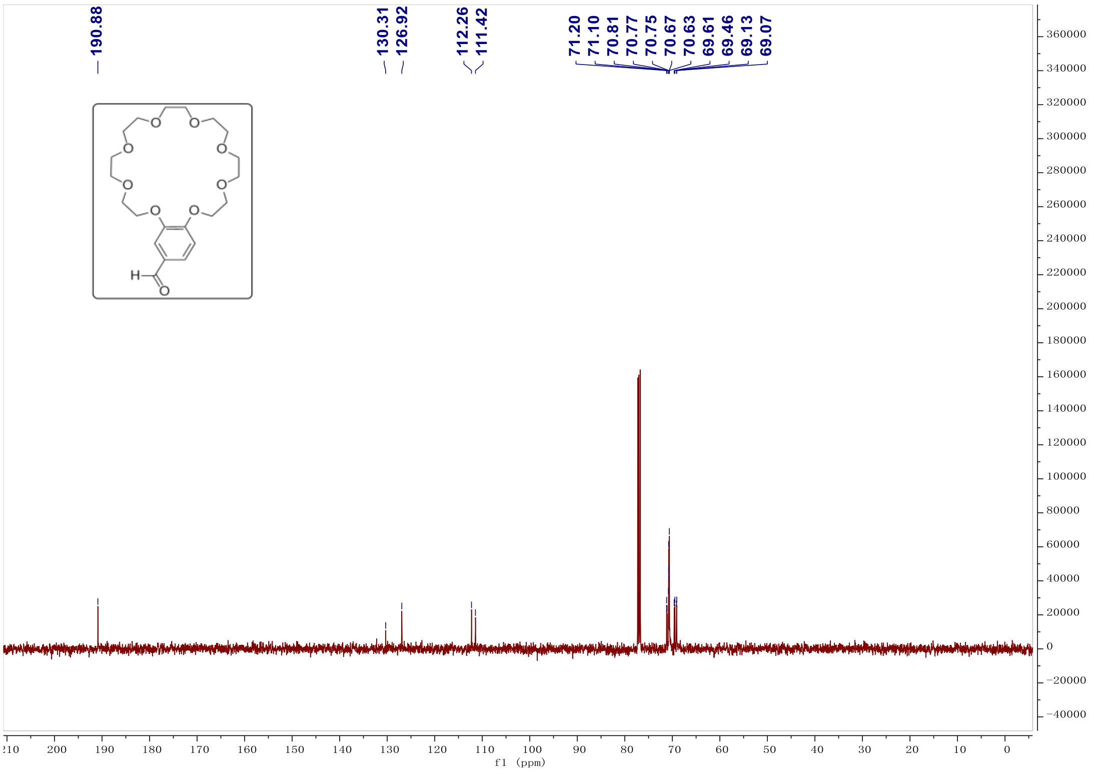

S5, ^{1}H NMR ($CDCl_3$, 500 MHz)

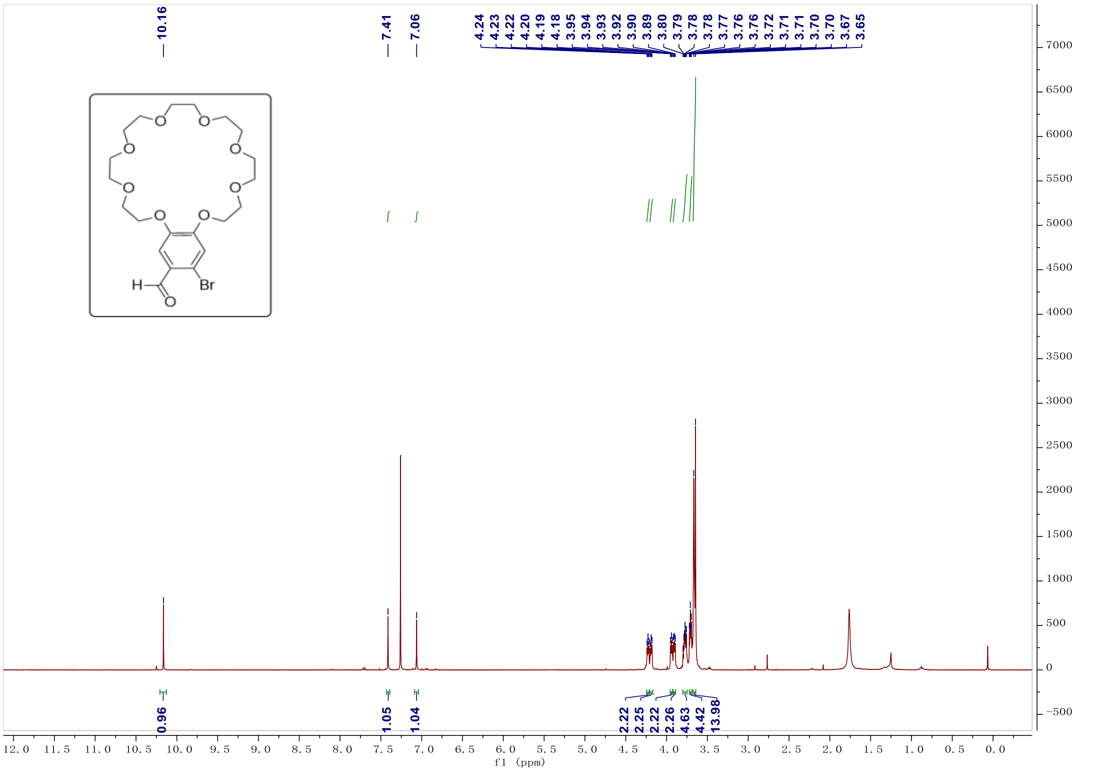

S5, ^{13}C NMR ($CDCl_3$, 126 MHz)

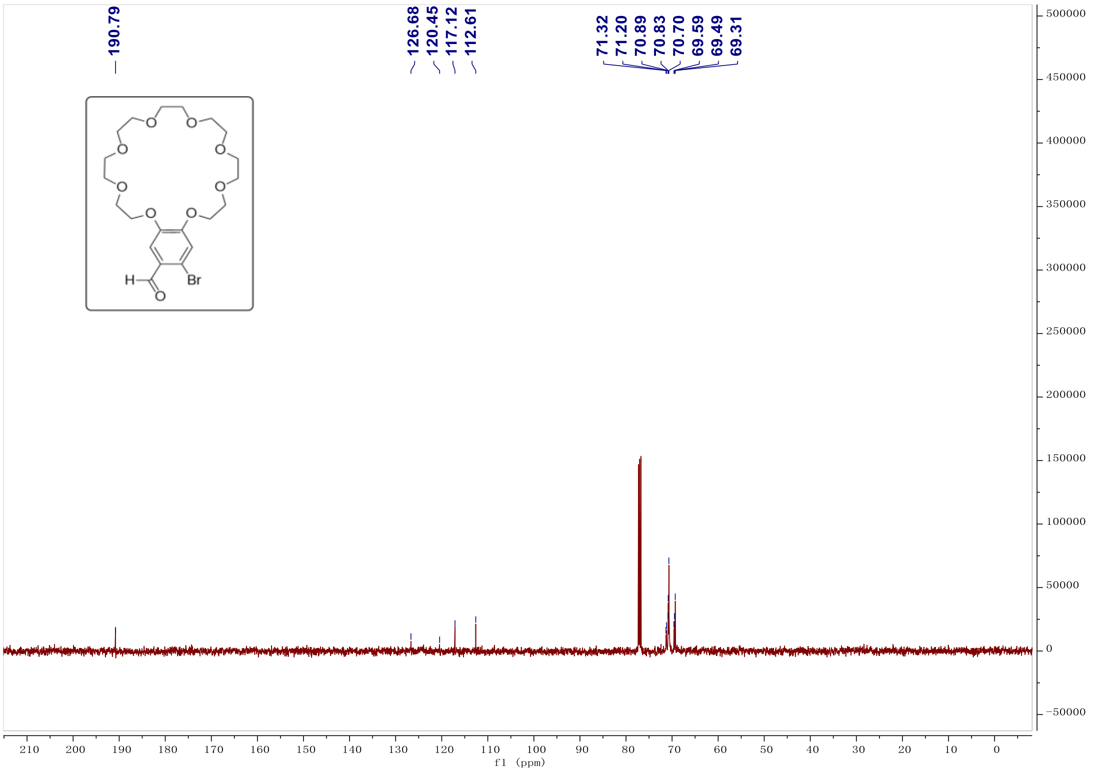

S6, ^{1}H NMR ($CDCl_3$, 500 MHz)

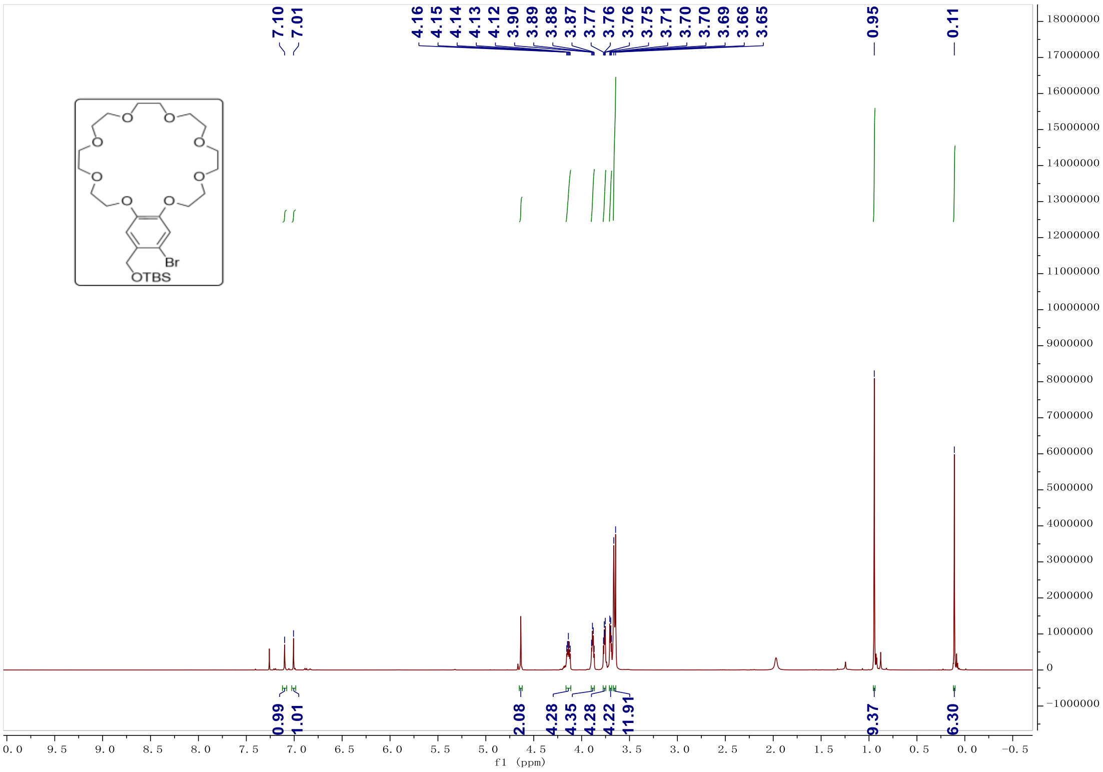

S6, ^{13}C NMR ($CDCl_3$, 126 MHz)

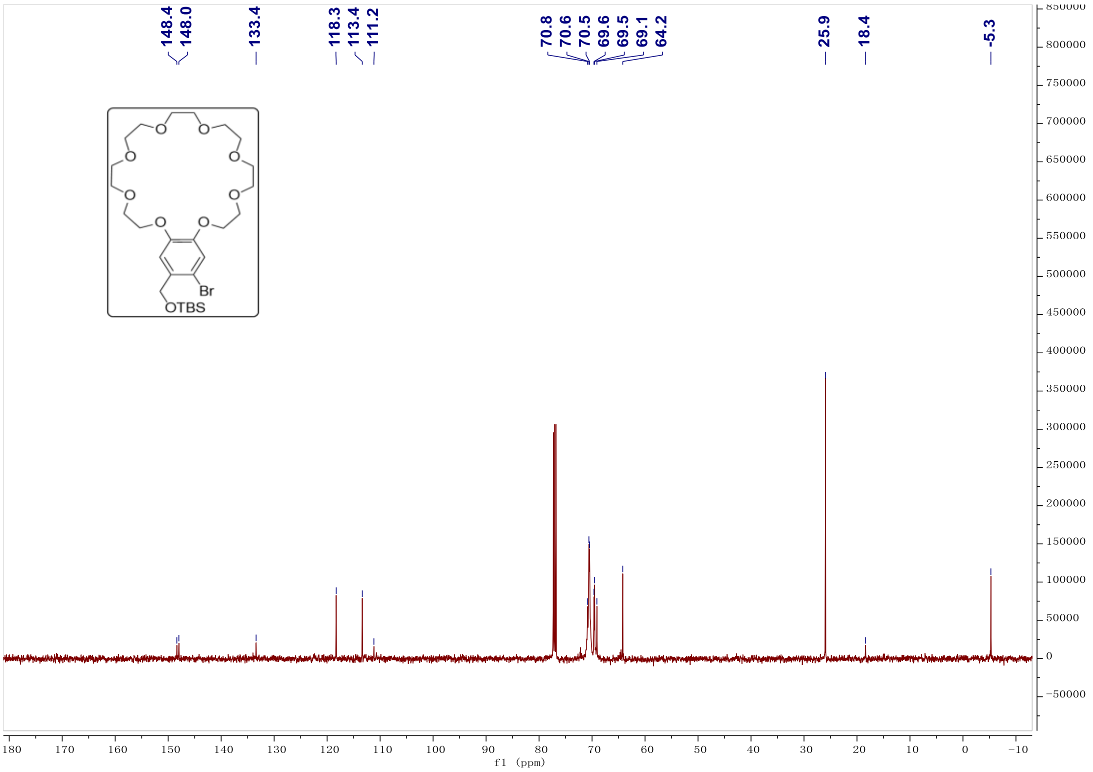

S7, ^{1}H NMR ($CDCl_3$, 500 MHz)

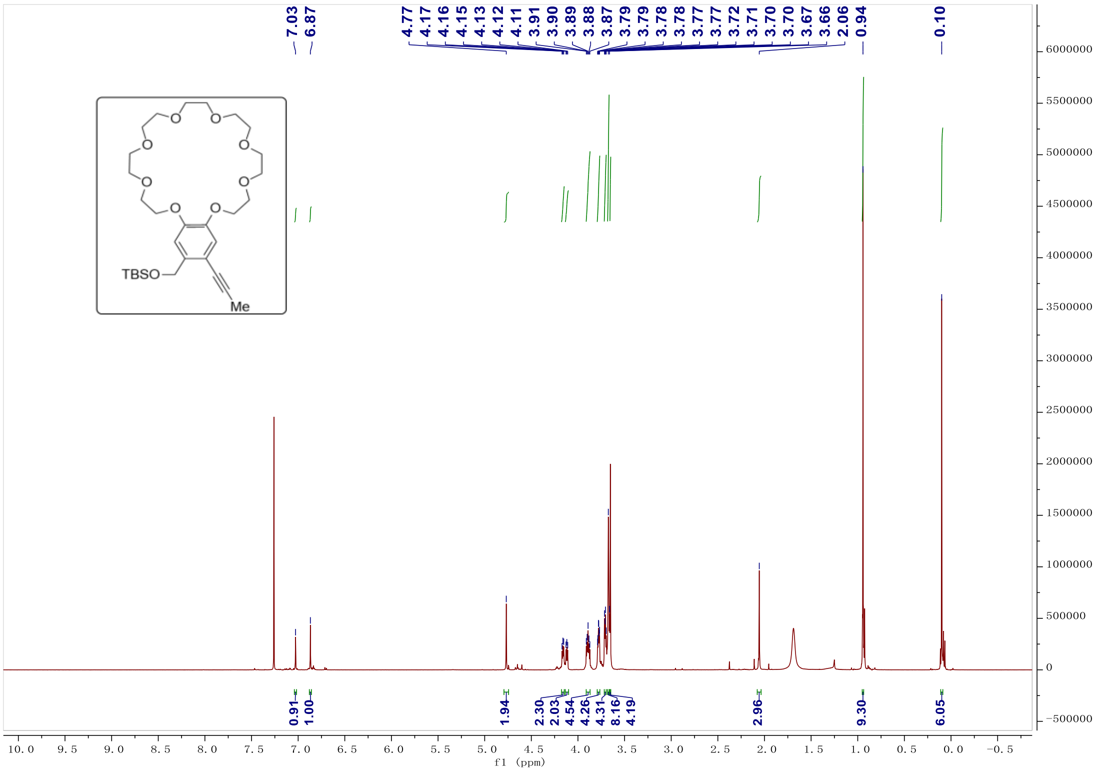

S7, ^{13}C NMR ($CDCl_3$, 126 MHz)

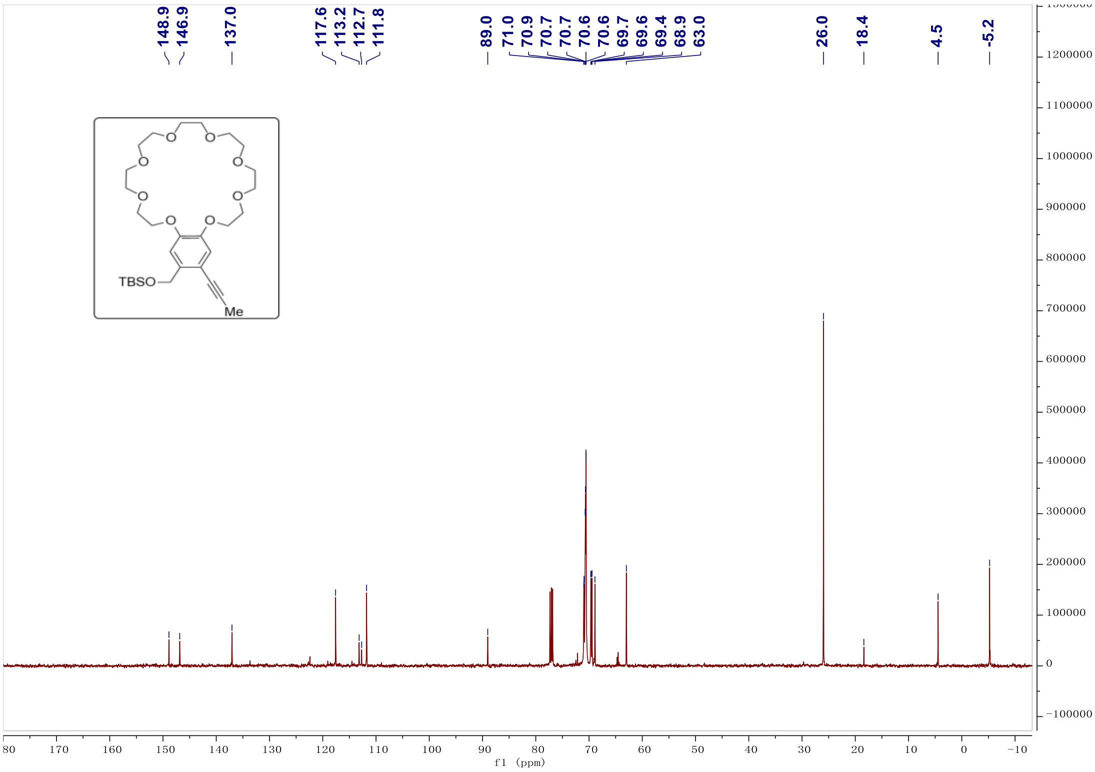

8, ^{1}H NMR (CD_2Cl_2, 500 MHz)

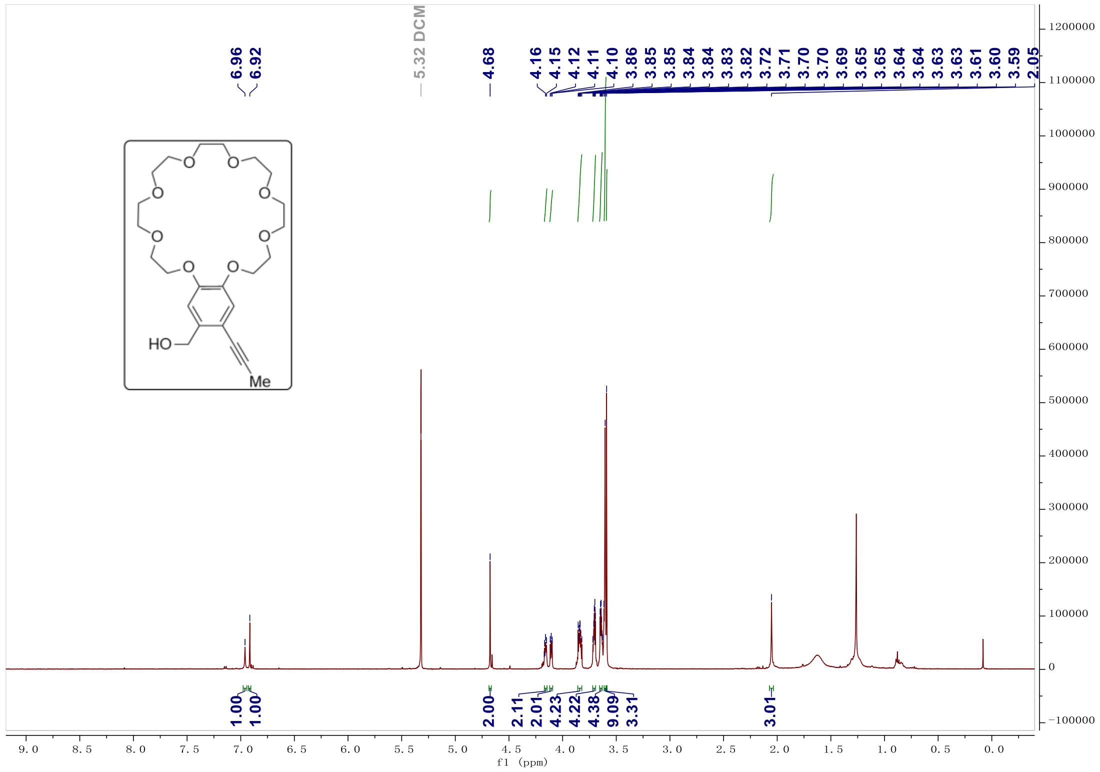

8, ^{13}C NMR (CD_2Cl_2, 126 MHz)

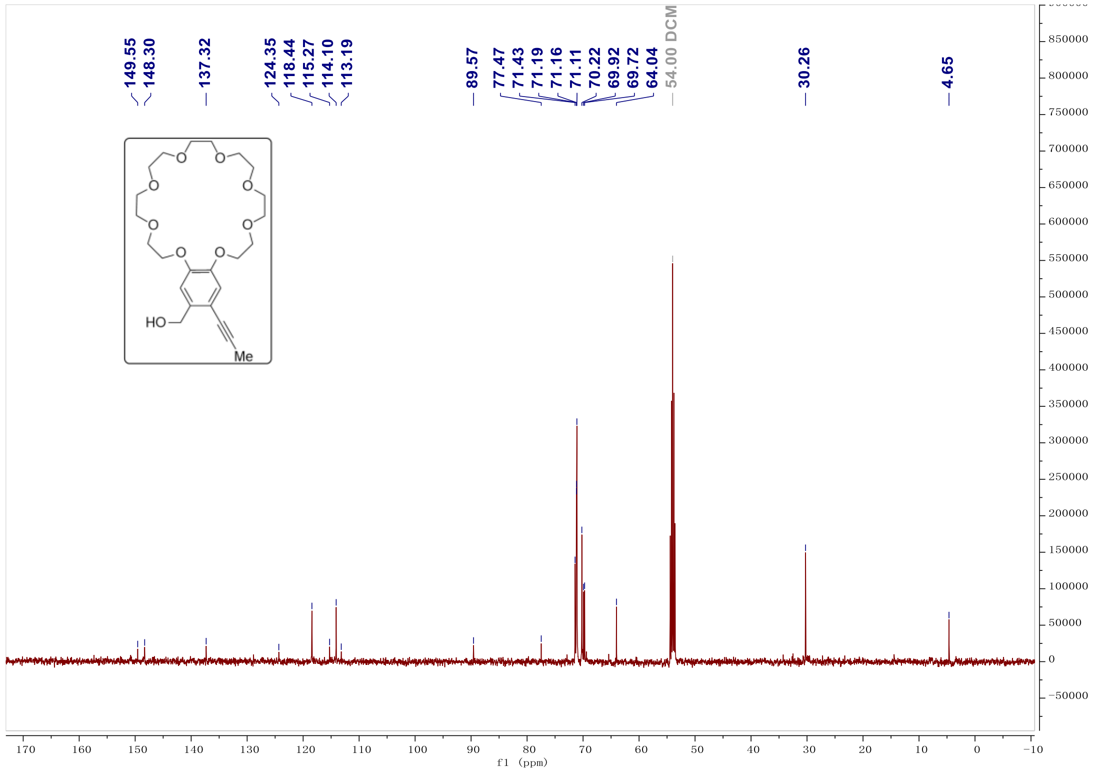

9, ^{1}H NMR ($CDCl_3$, 500 MHz)

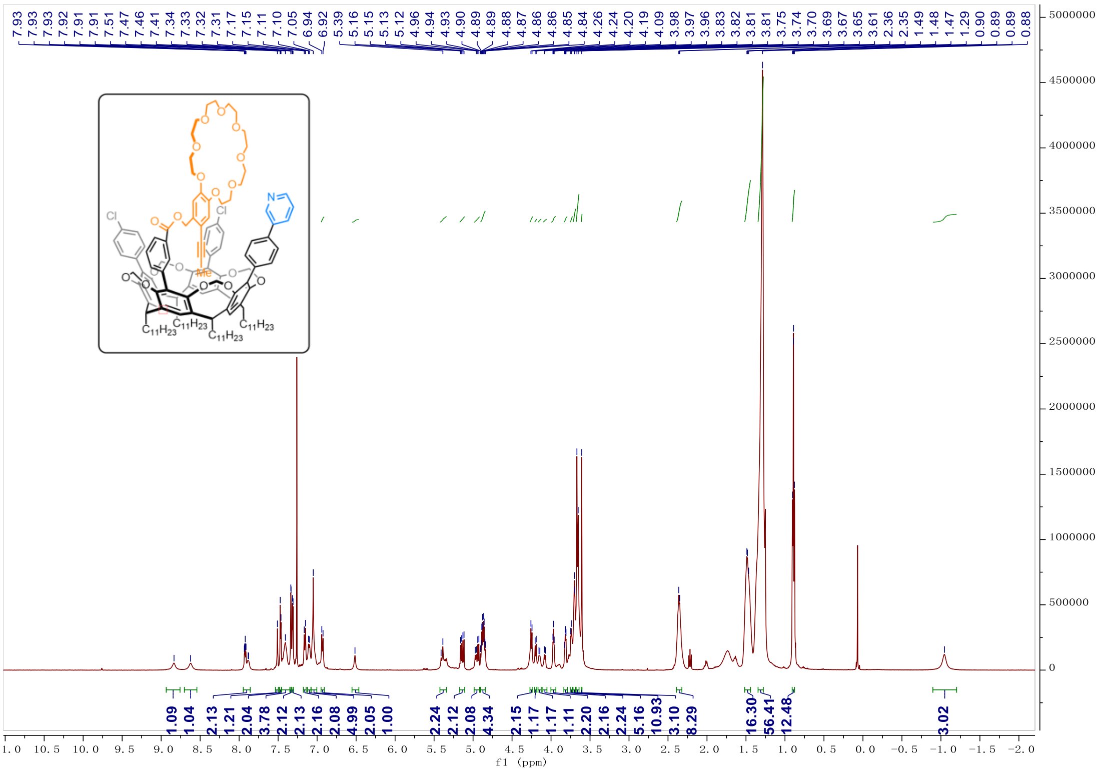

9, ^{13}C NMR ($CDCl_3$, 126 MHz)

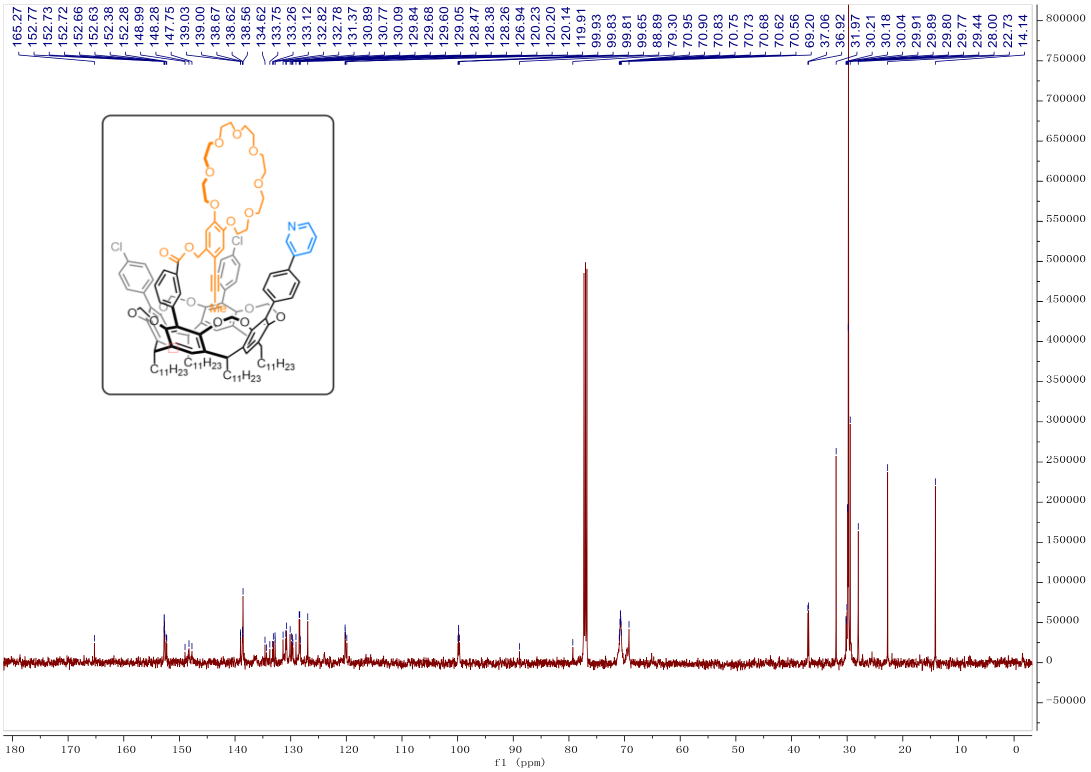

10, ^{1}H NMR ($CDCl_3$, 400 MHz)

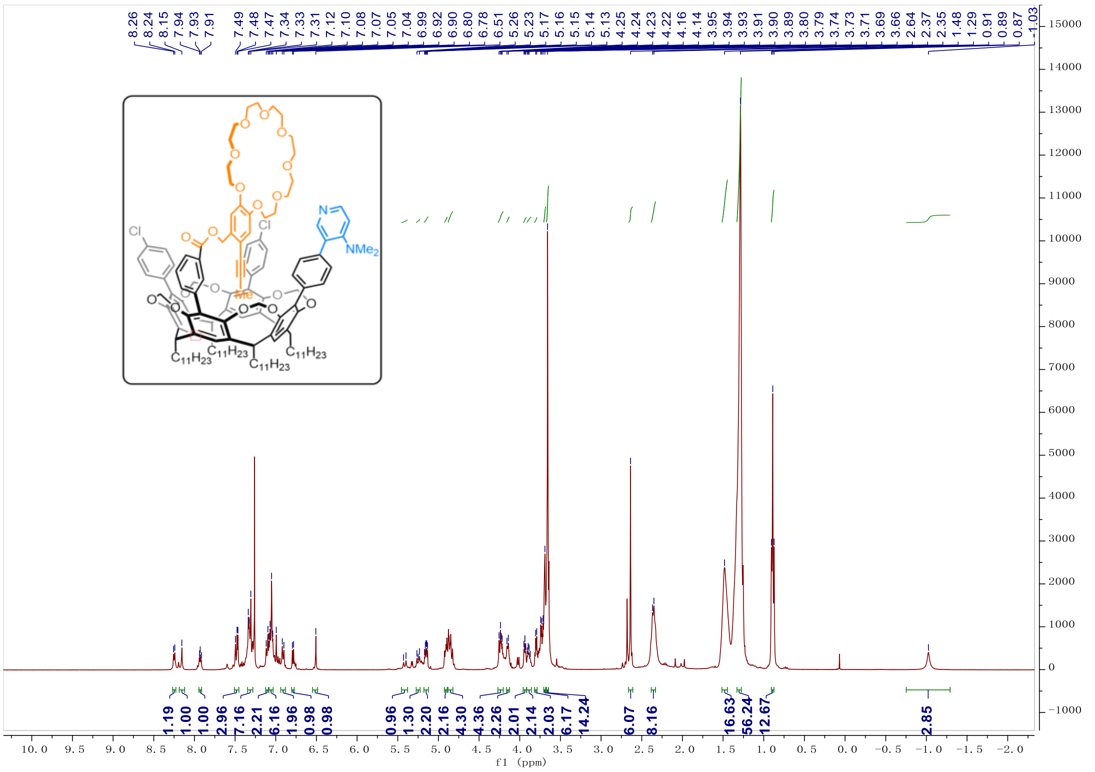

10, ^{13}C NMR ($CDCl_3$, 126 MHz)

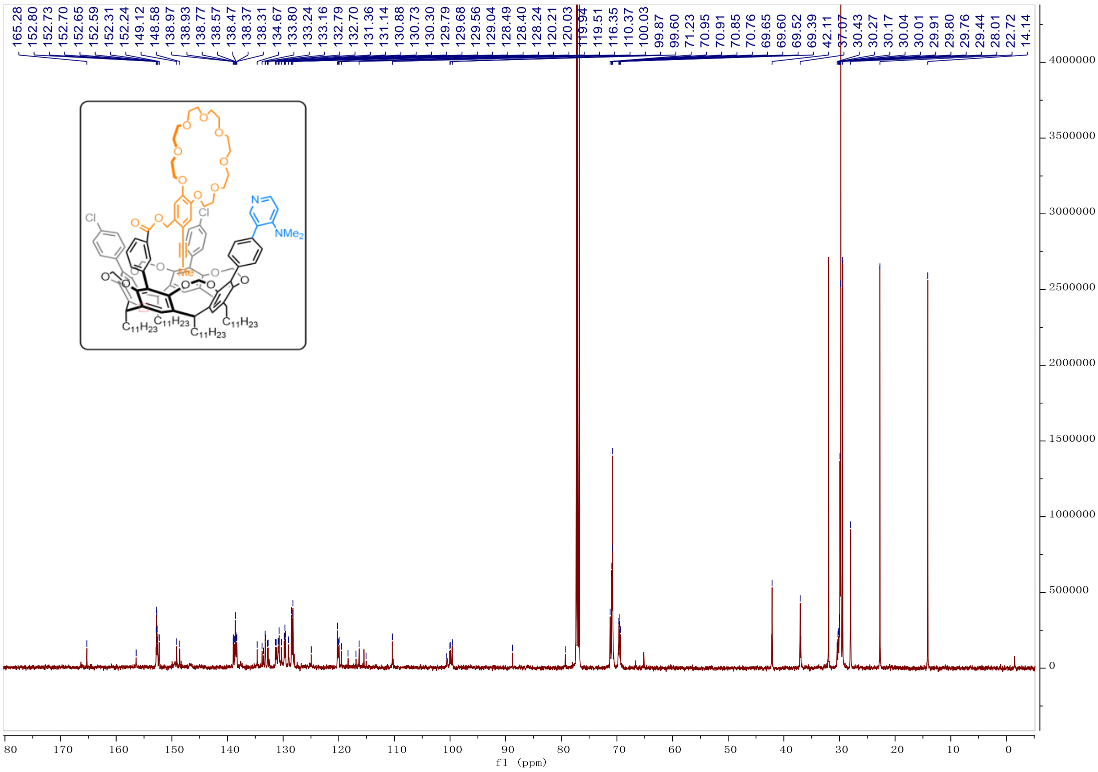

11, ^{1}H NMR ($CDCl_3$, 500 MHz)

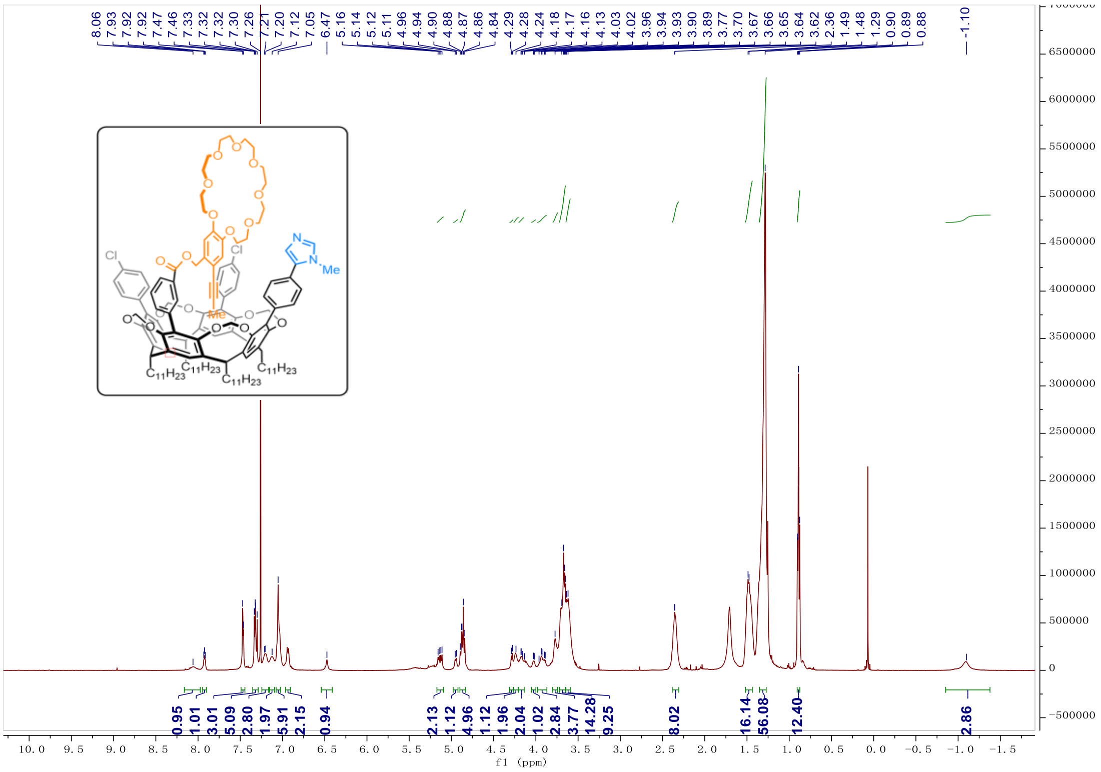

11, ^{13}C NMR ($CDCl_3$, 126 MHz)

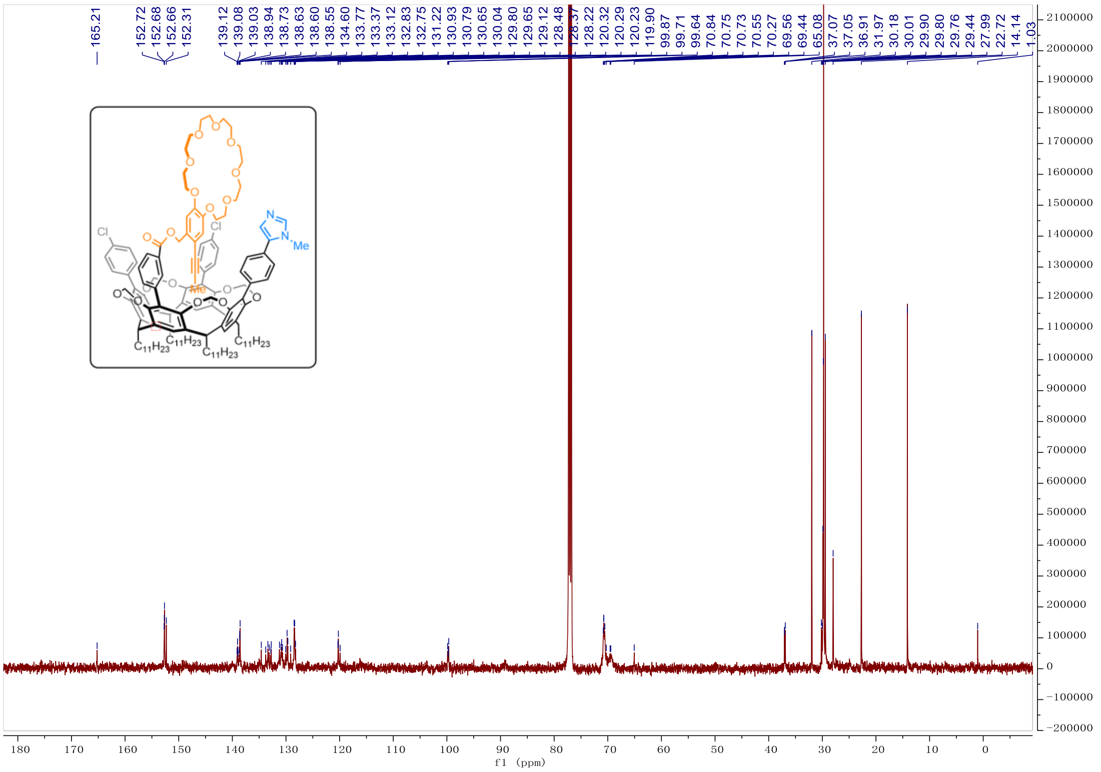

12, ^{1}H NMR ($CDCl_3$, 500 MHz)

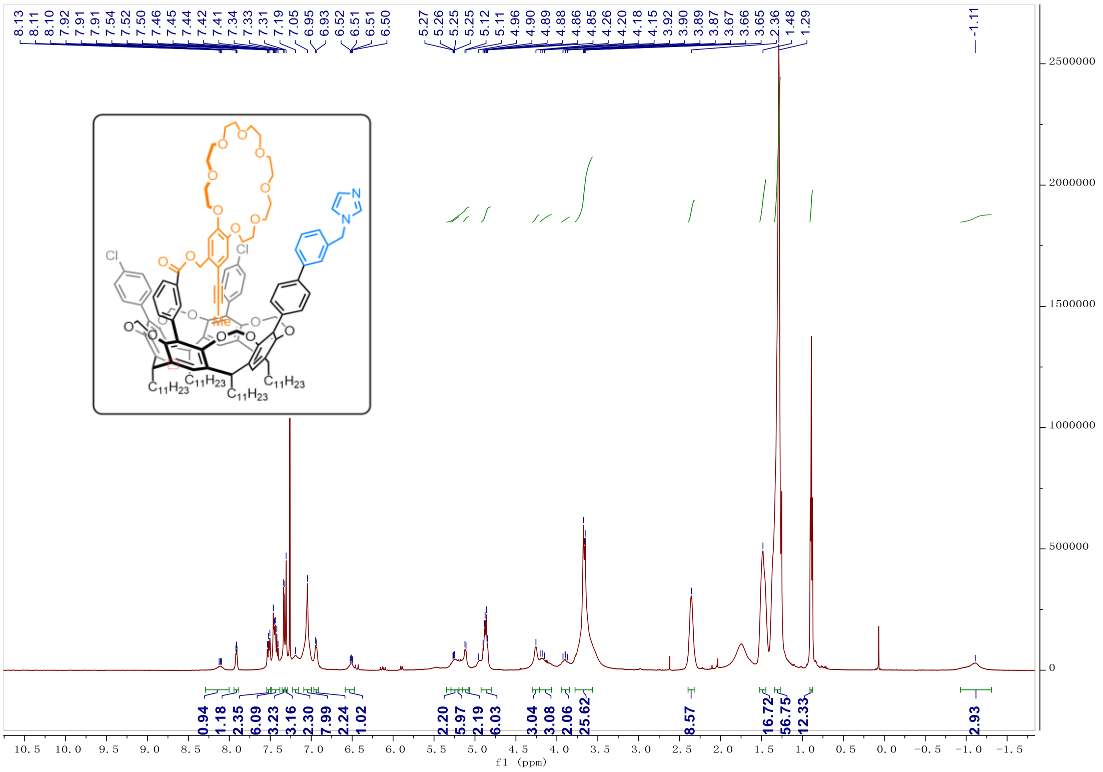

12, ^{13}C NMR ($CDCl_3$, 126 MHz)

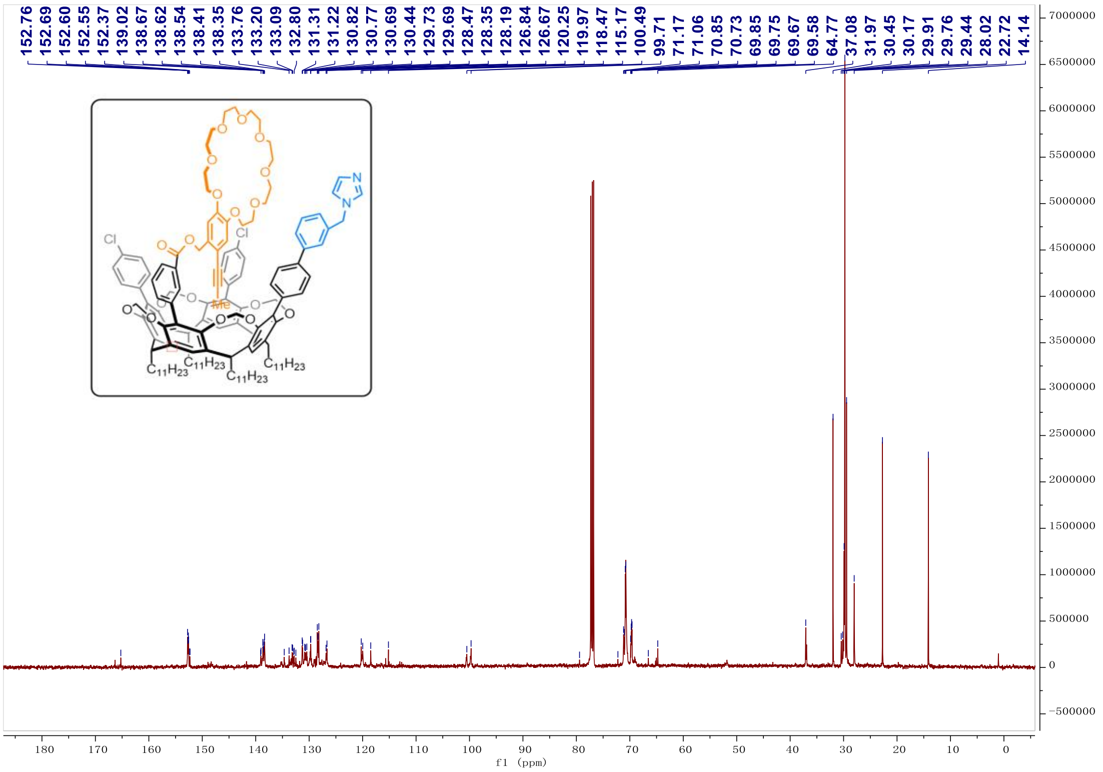

13, ^{1}H NMR ($CDCl_3$, 500 MHz)

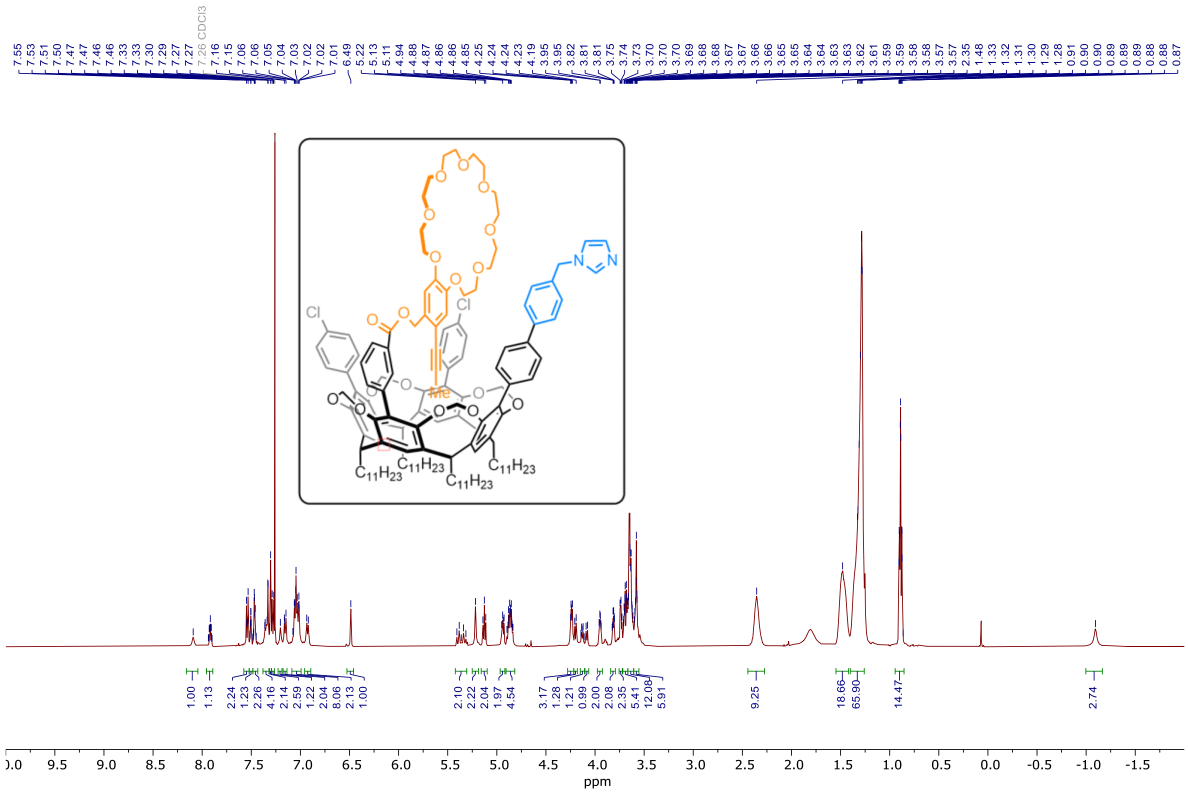

13, ^{13}C NMR ($CDCl_3$, 126 MHz)

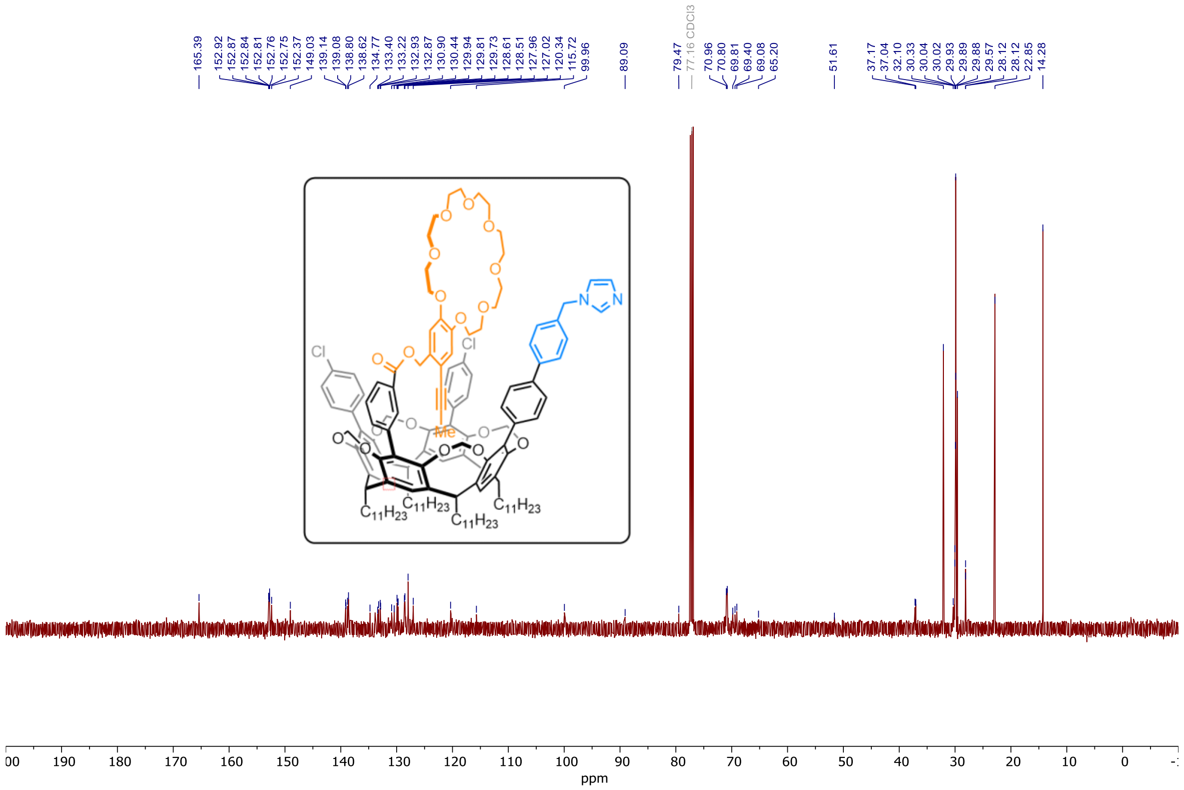

20, ^{1}H NMR ($CDCl_3$, 500 MHz)

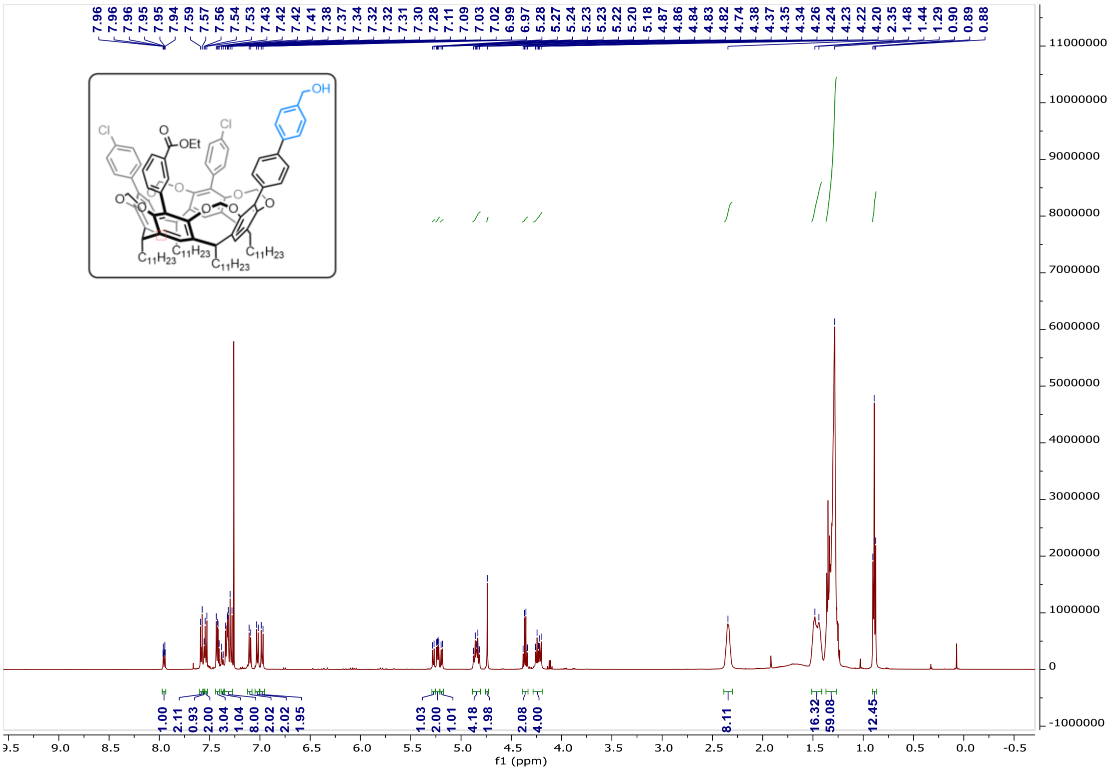

20, ^{13}C NMR ($CDCl_3$, 126 MHz)

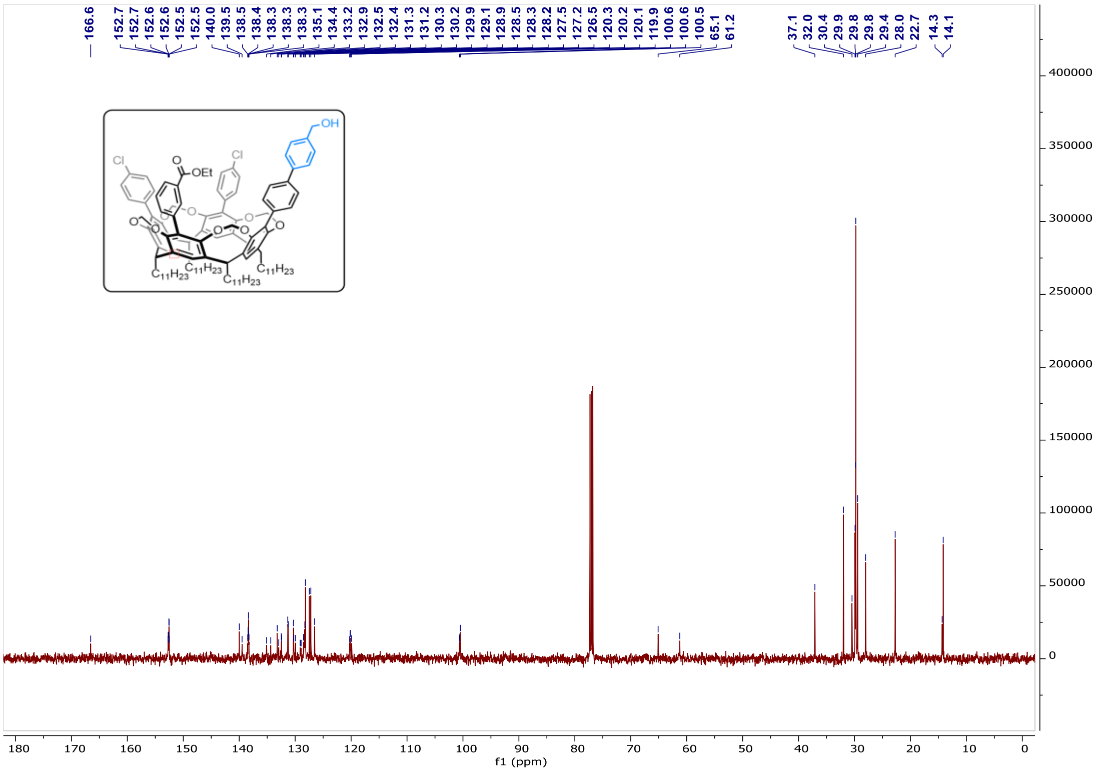

S8, ^{1}H NMR ($CDCl_3$, 500 MHz)

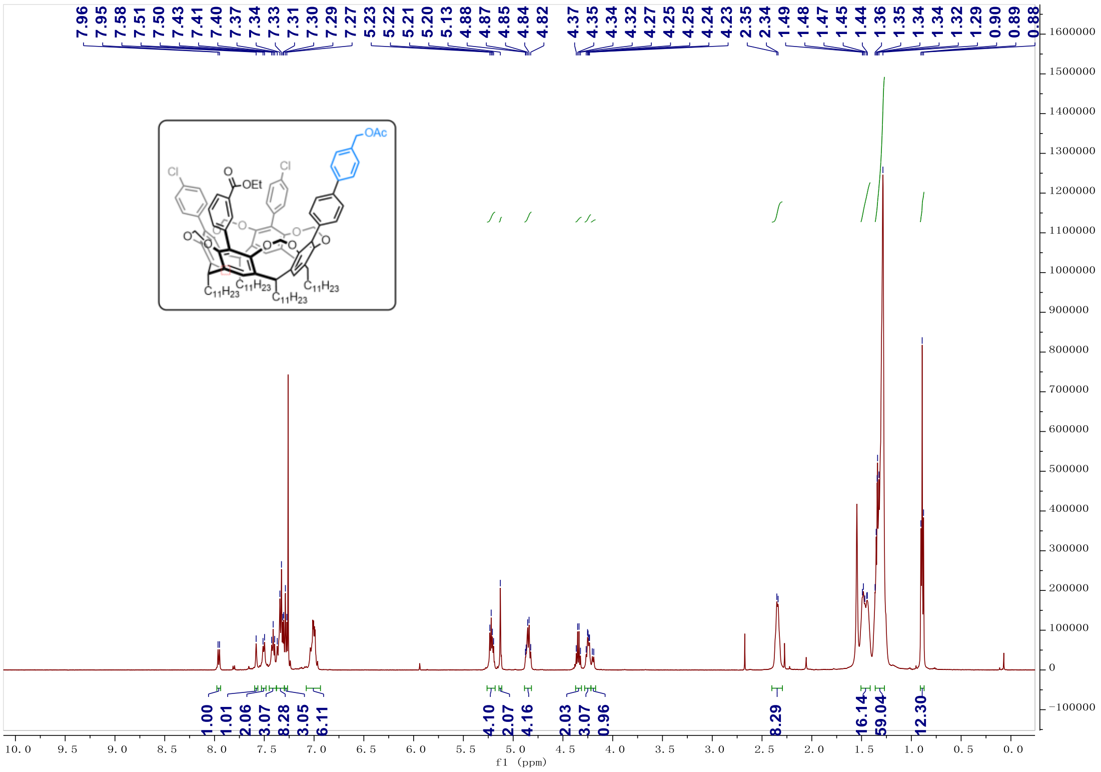

S8, ^{13}C NMR ($CDCl_3$, 126 MHz)

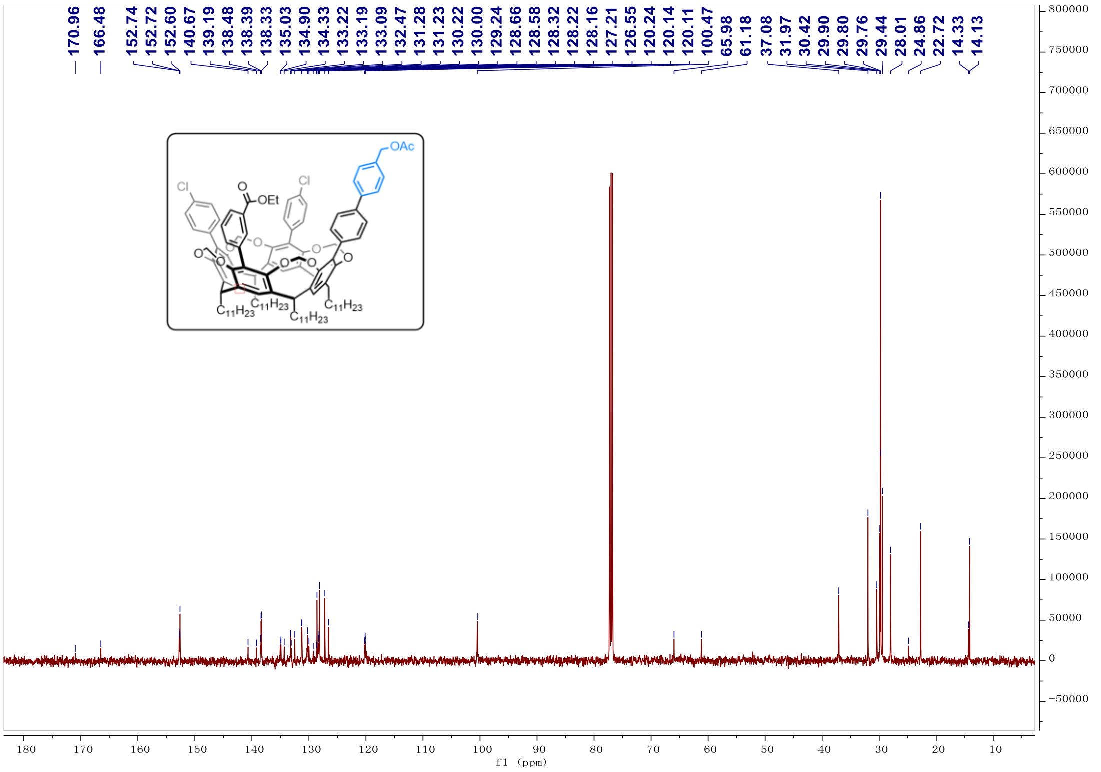

S9, ^{1}H NMR ($CDCl_3$, 500 MHz)

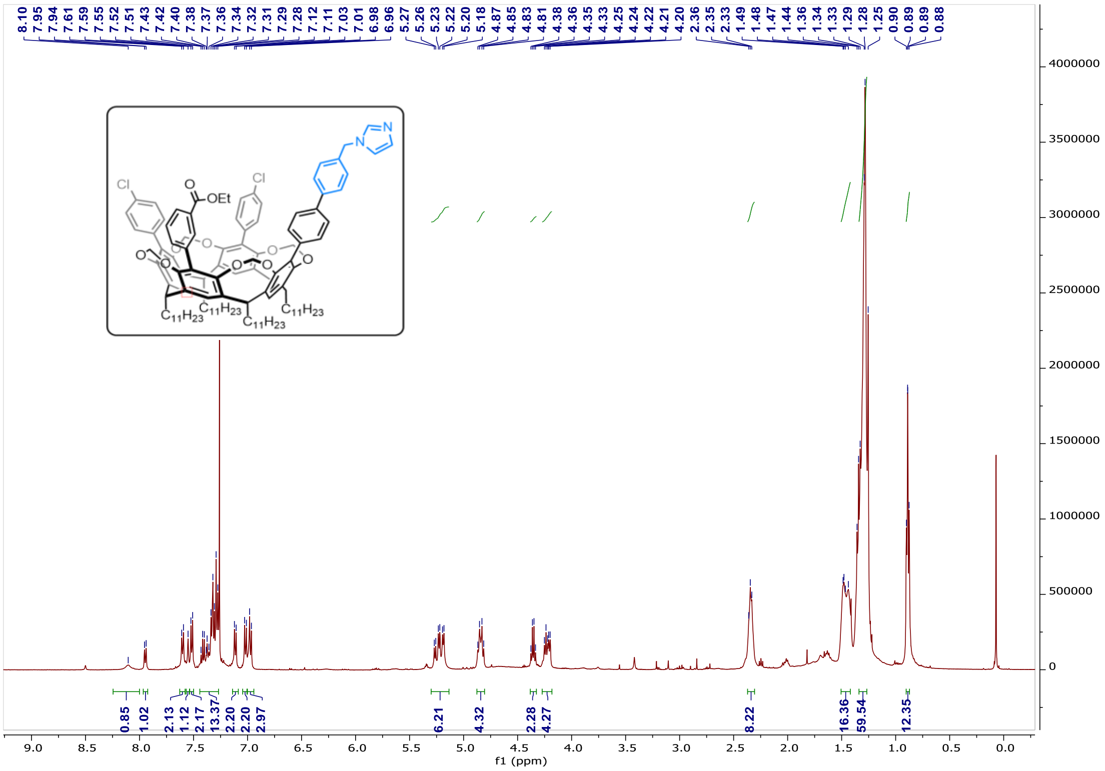

S9, ^{13}C NMR ($CDCl_3$, 126 MHz)

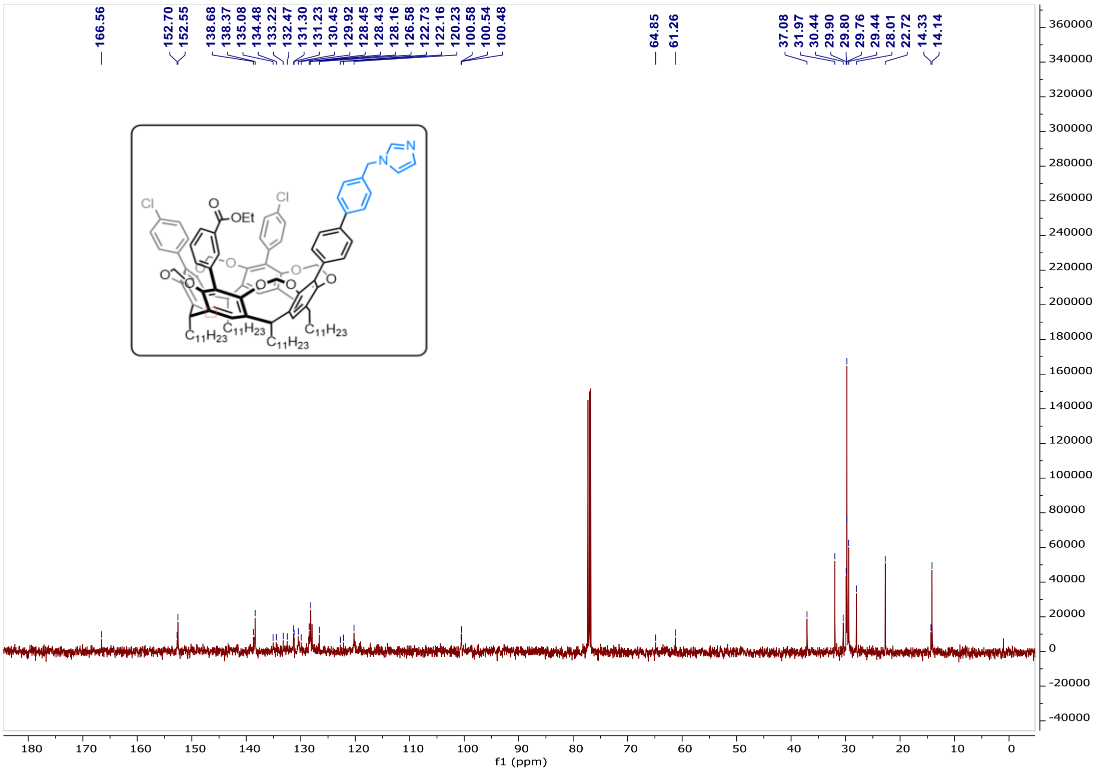

22, ^{1}H NMR (CD_2Cl_2, 500 MHz)

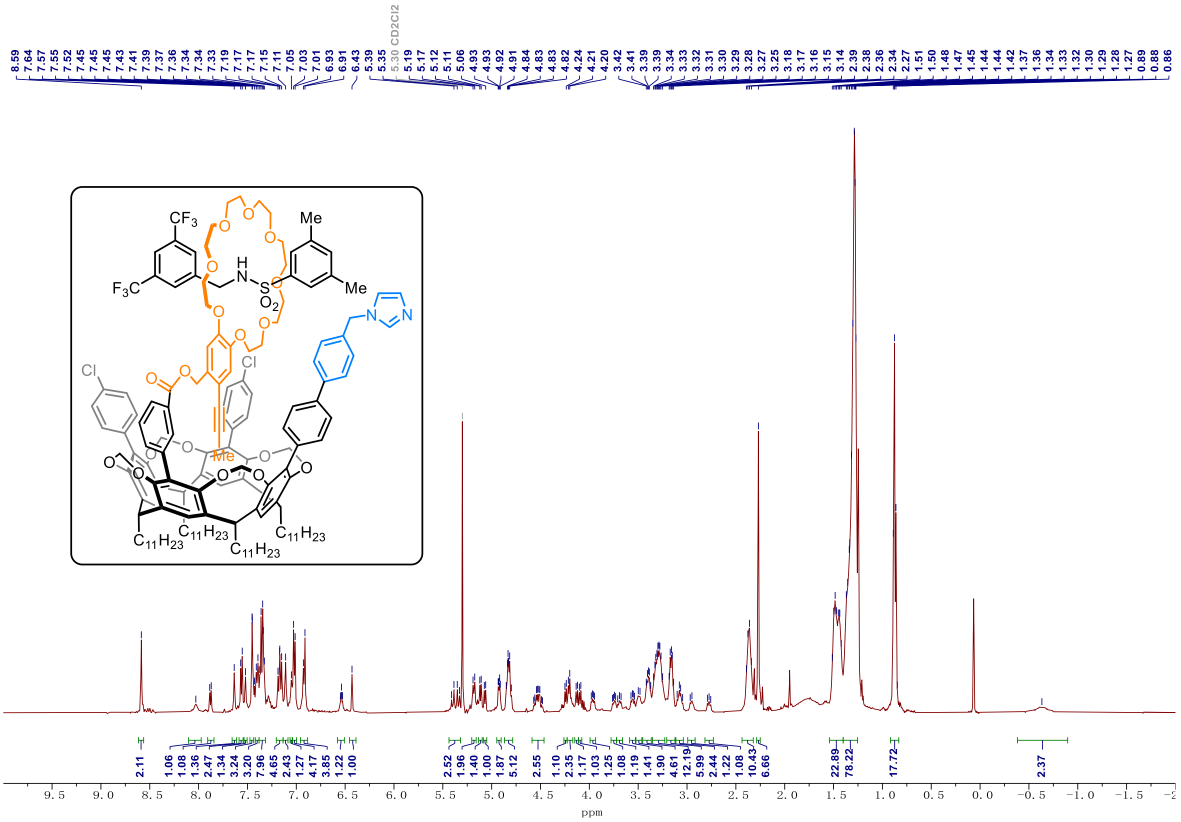

22, ^{13}C NMR (CD_2Cl_2, 126 MHz)

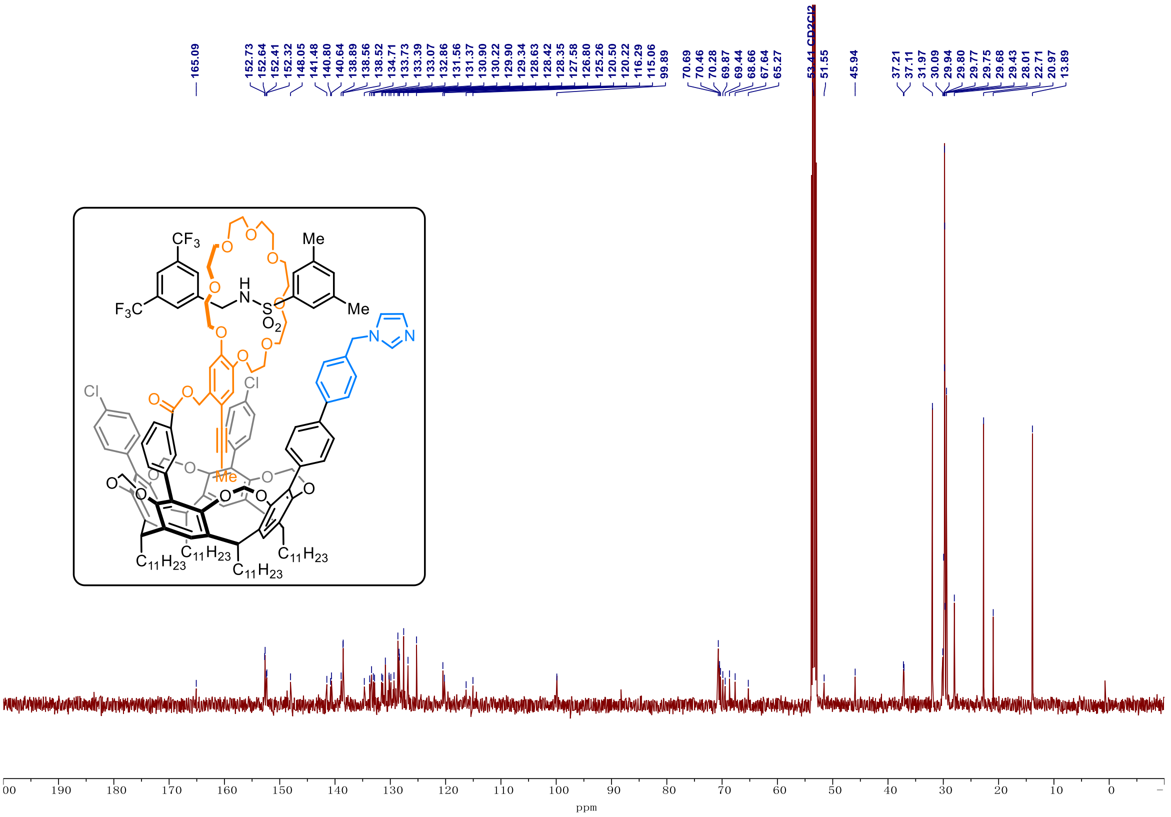

17, ^{1}H NMR ($CDCl_3$, 500 MHz)

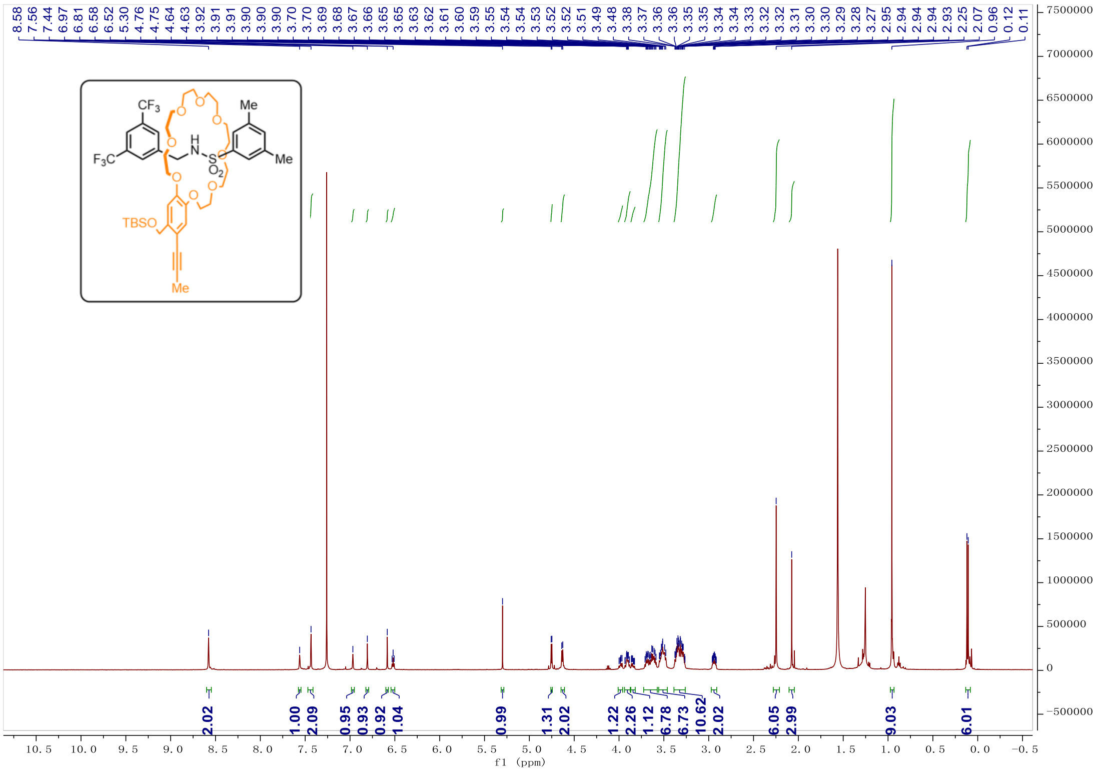

17, ^{13}C NMR ($CDCl_3$, 126 MHz)

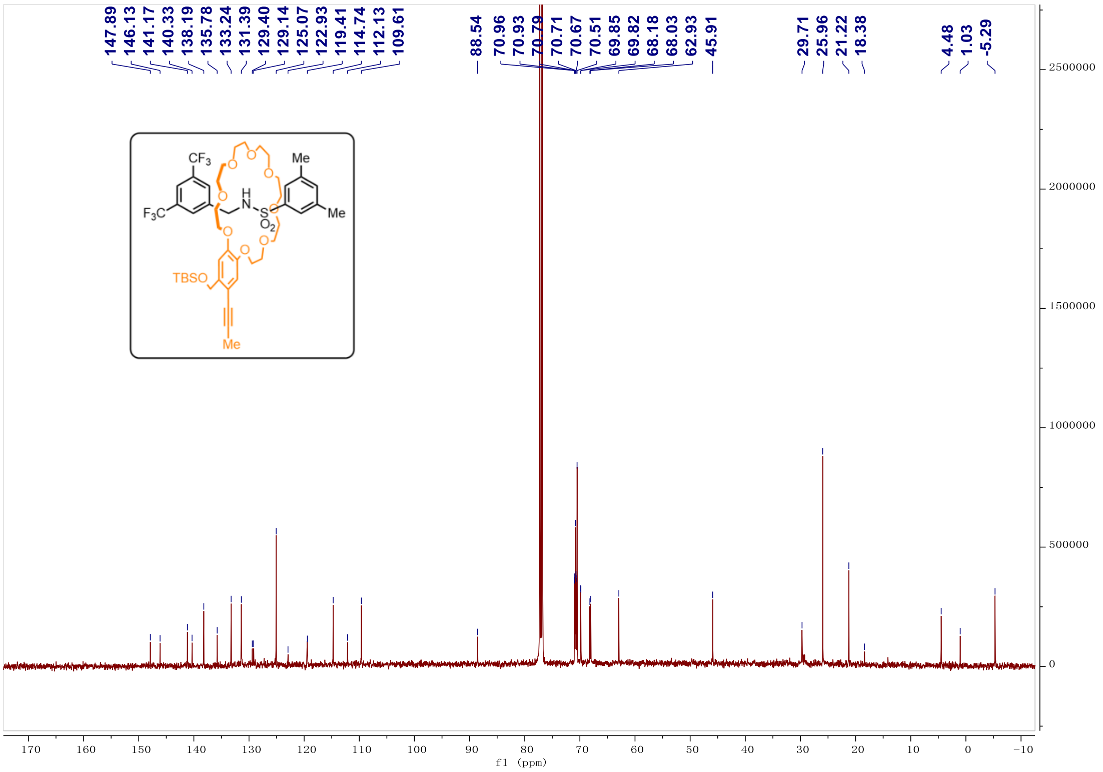

17, ^{19}F NMR ($CDCl_3$, 377 MHz)

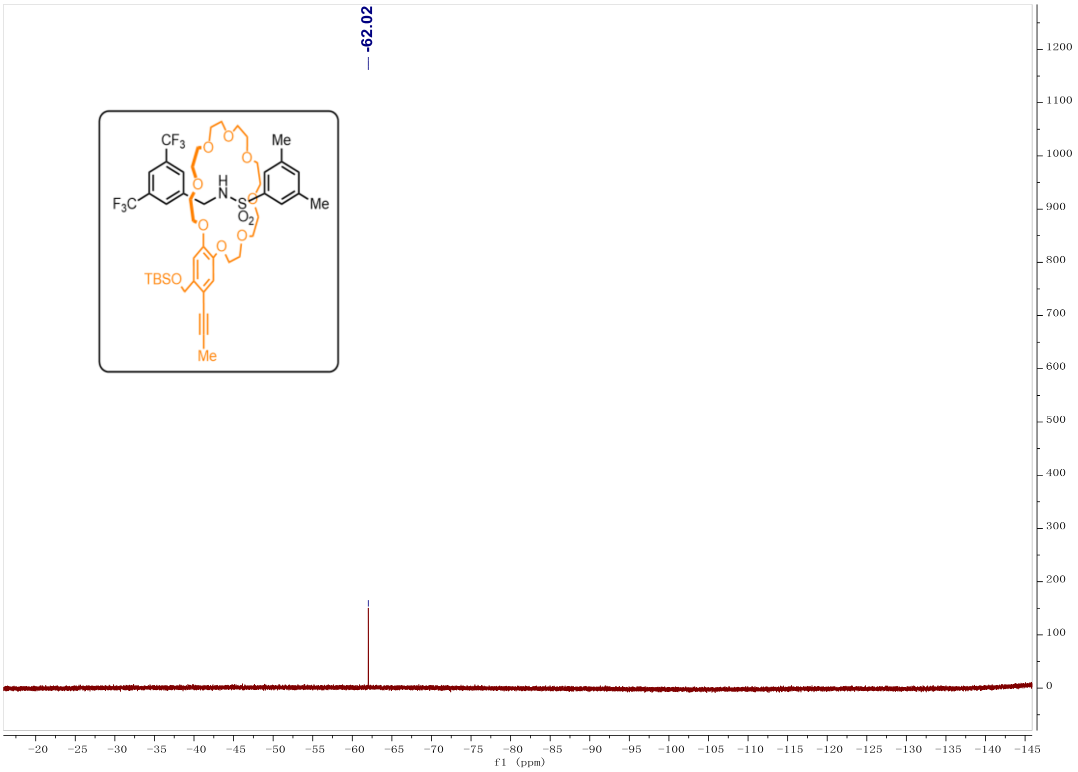

23, ^{1}H NMR ($CDCl_3$, 500 MHz)

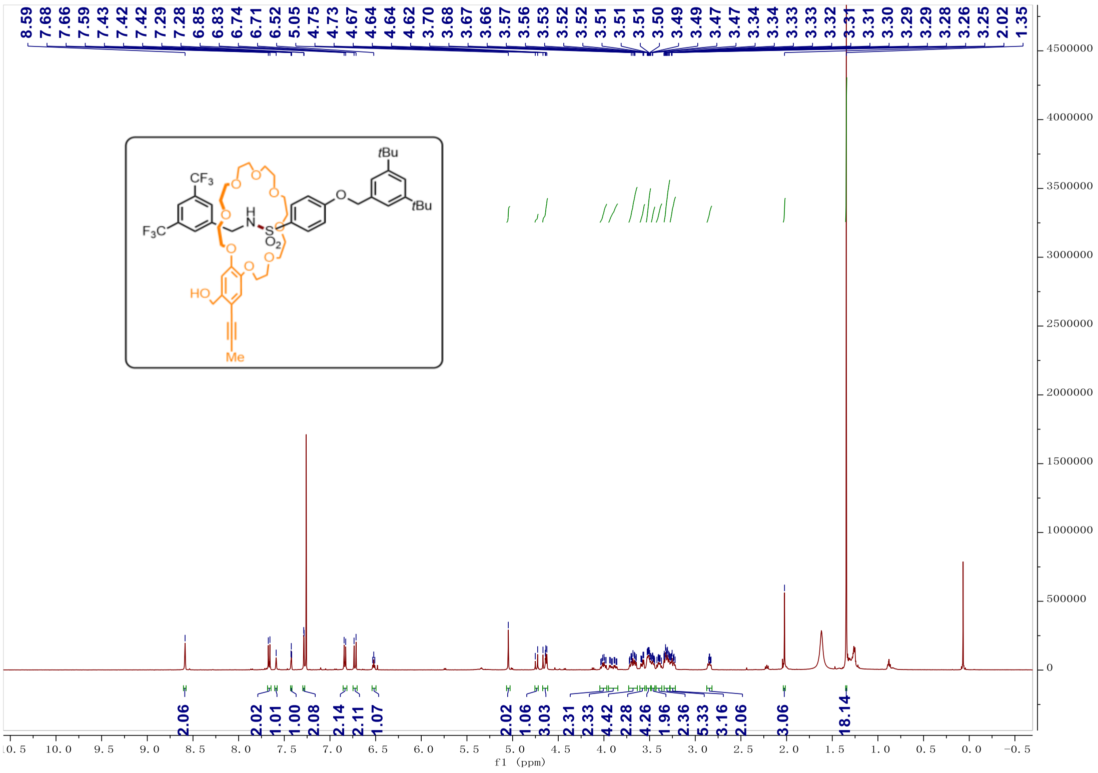

23, ^{13}C NMR ($CDCl_3$, 126 MHz)

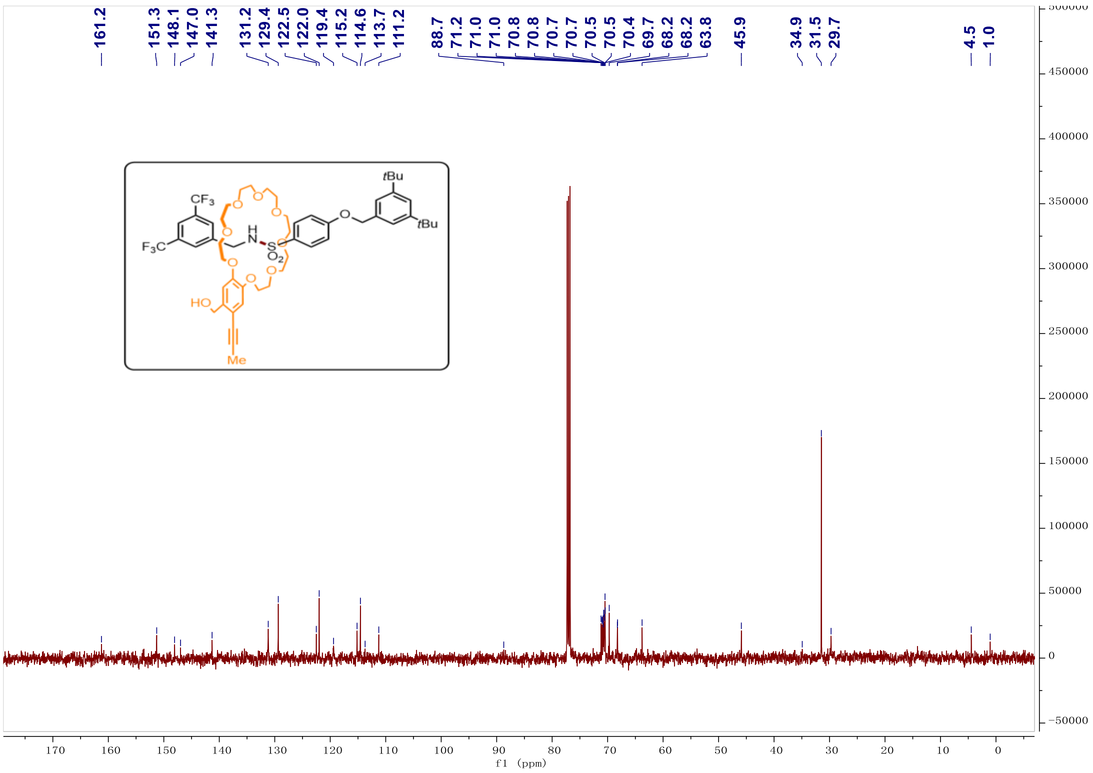

23, ^{19}F NMR ($CDCl_3$, 377 MHz)

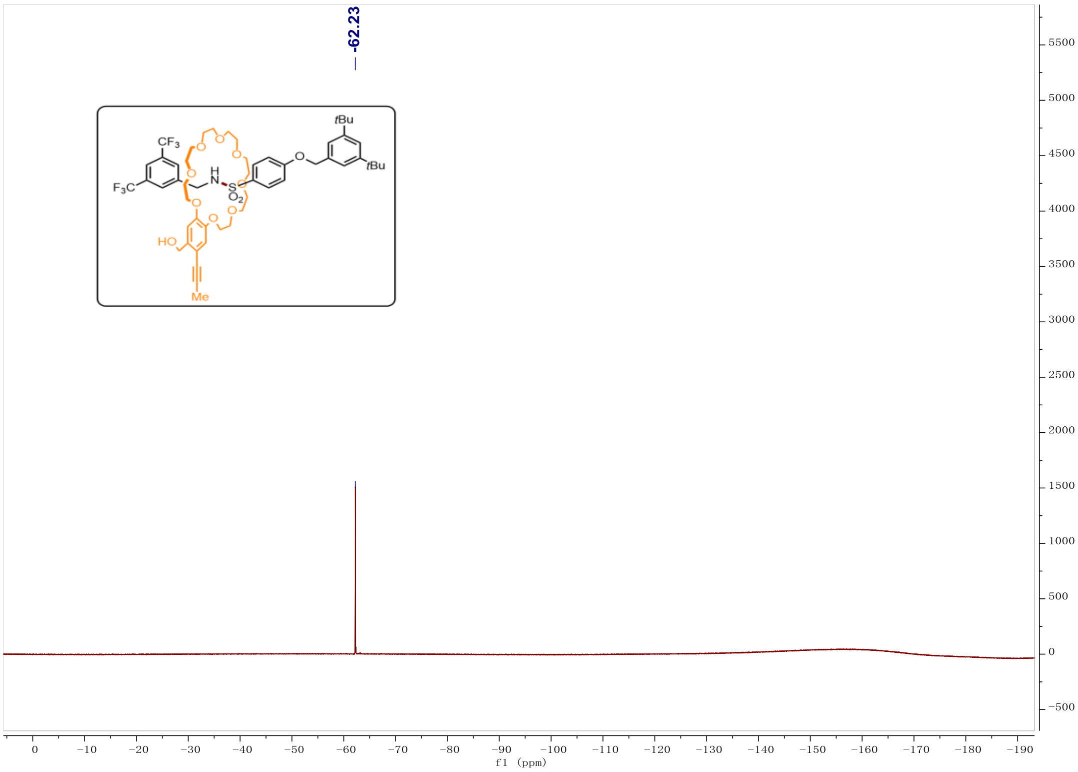

24, ^{1}H NMR ($CDCl_3$, 500 MHz)

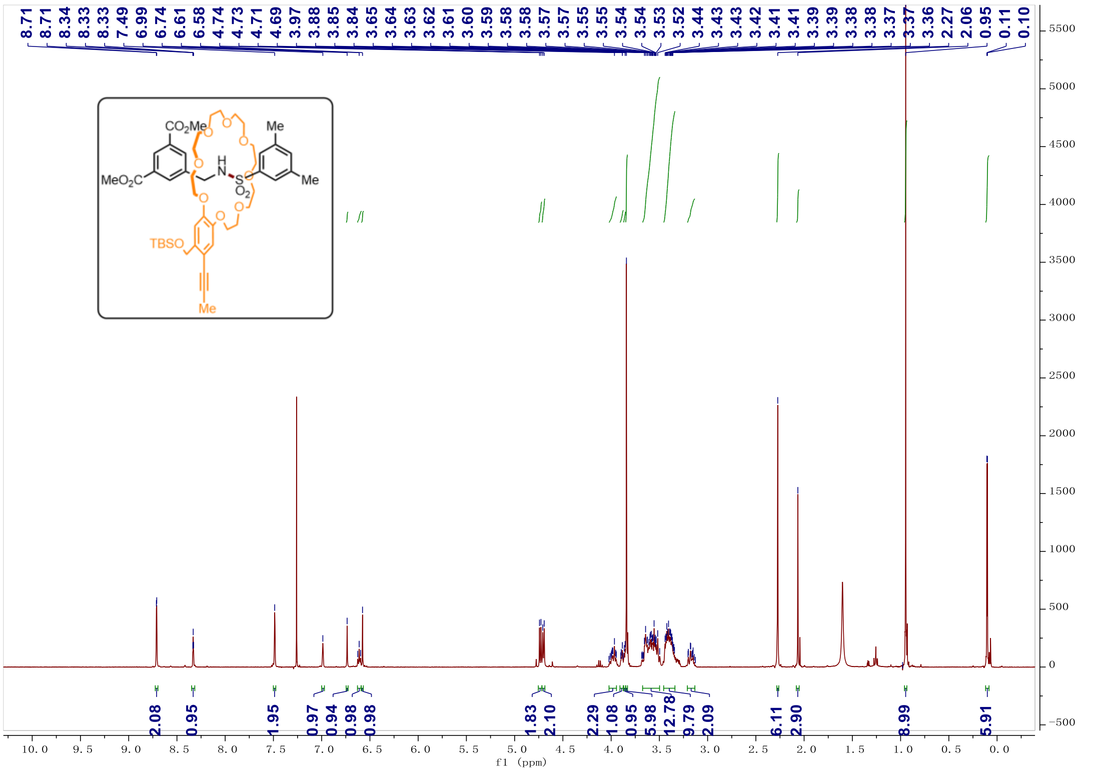

24, ^{13}C NMR ($CDCl_3$, 126 MHz)

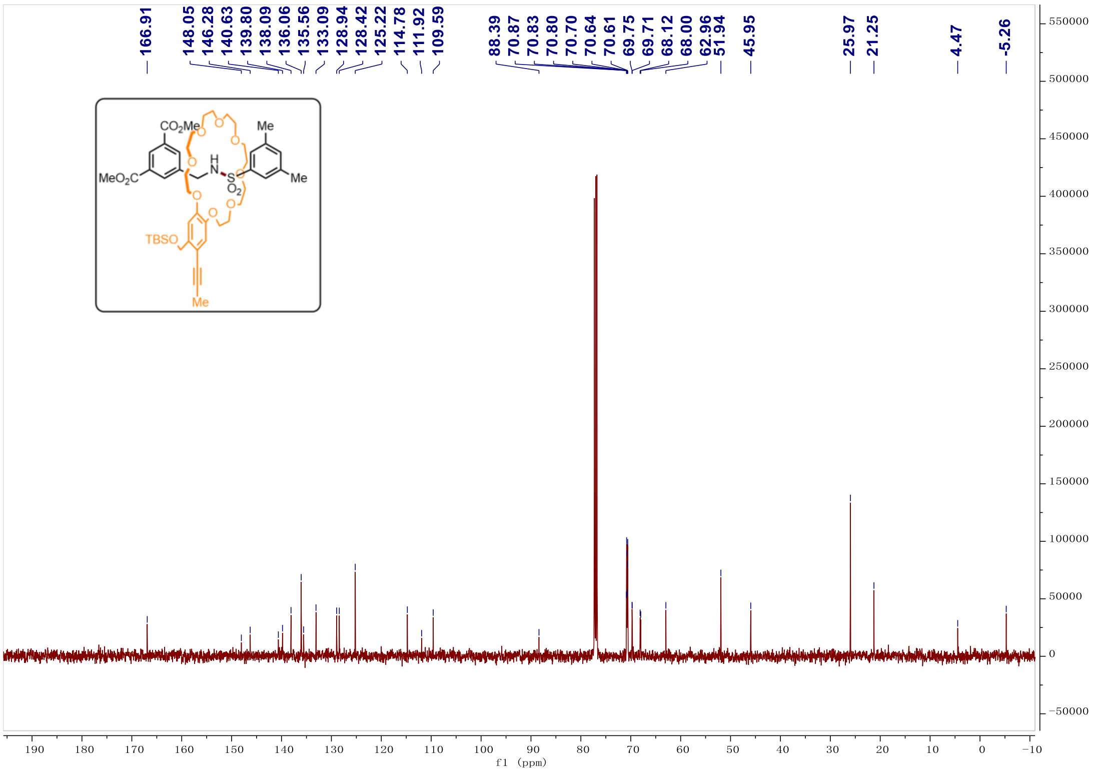

25, ^{1}H NMR ($CDCl_3$, 400 MHz)

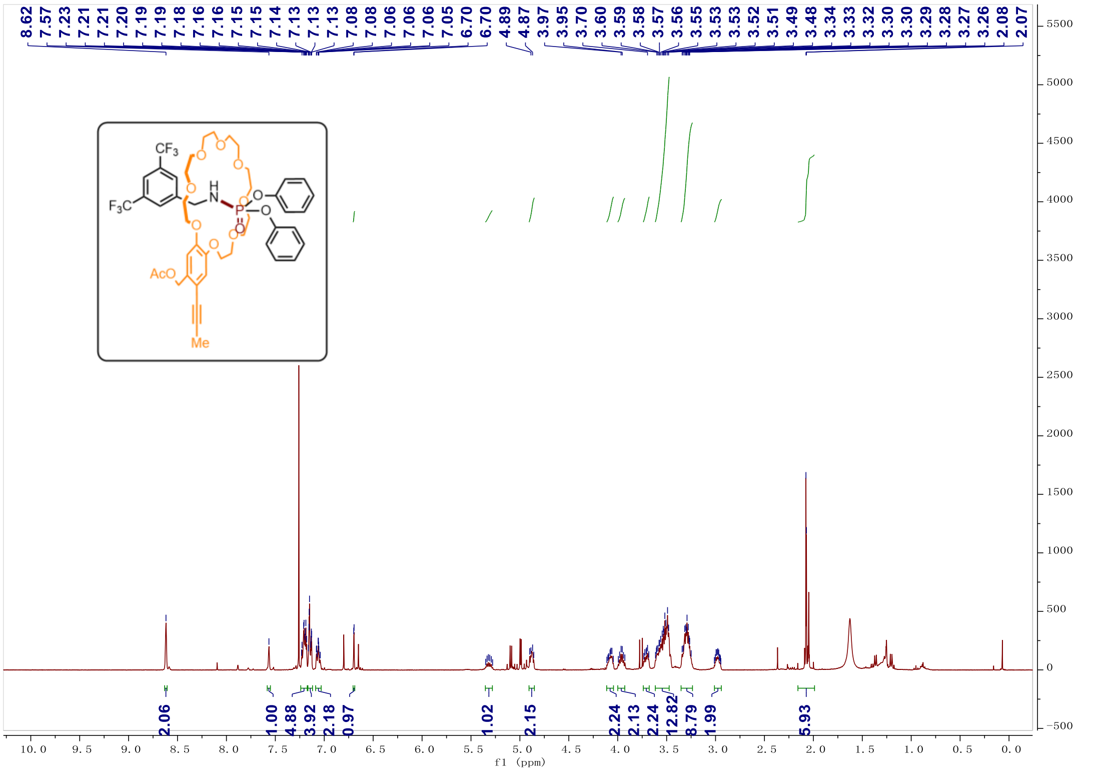

25, ^{13}C NMR ($CDCl_3$, 126 MHz)

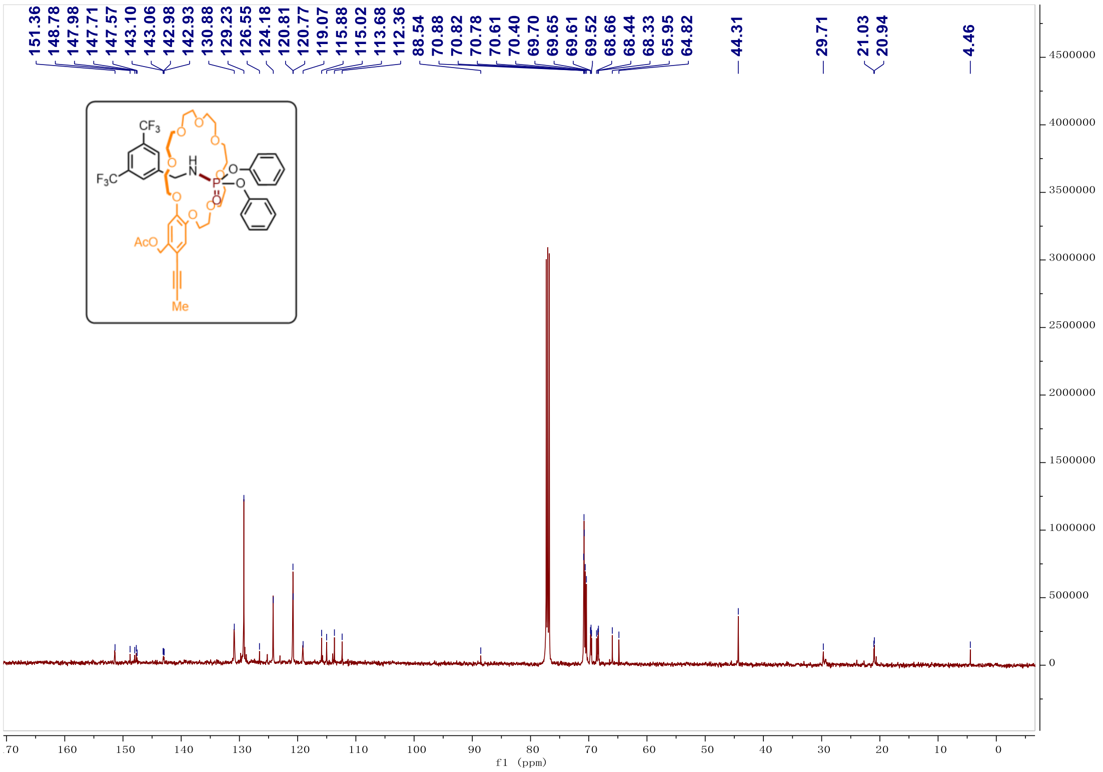

26, ^{1}H NMR ($CDCl_3$, 500 MHz)

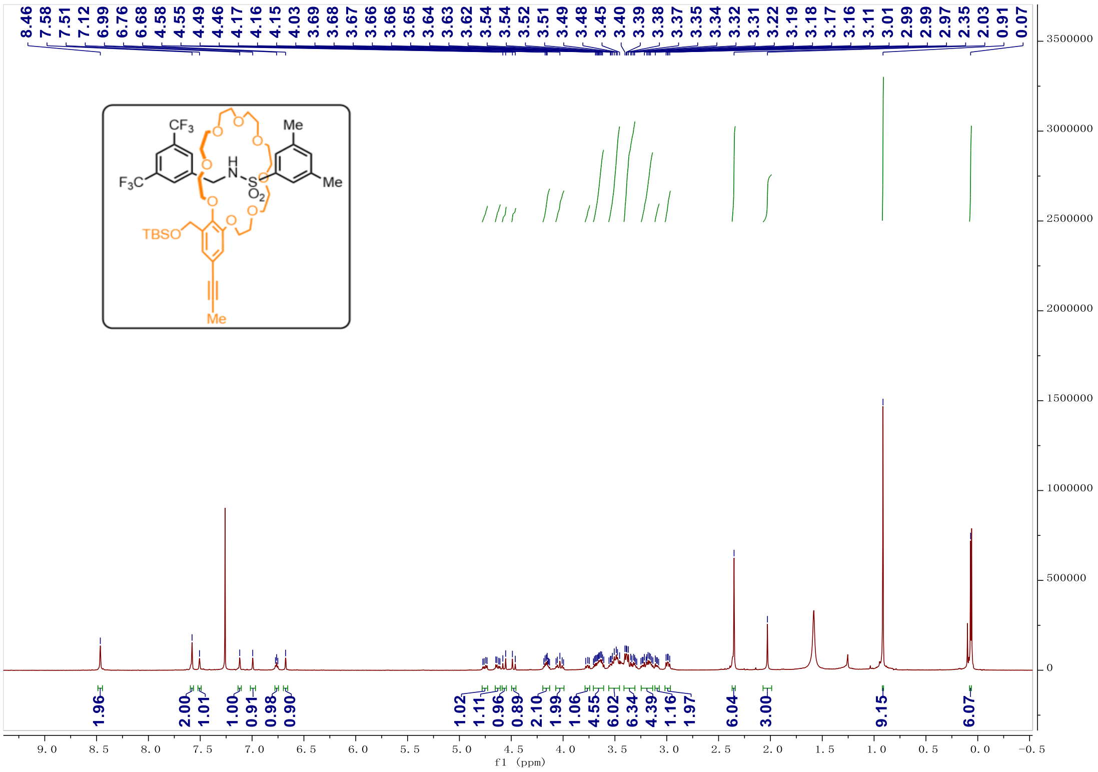

26, ^{13}C NMR ($CDCl_3$, 126 MHz)

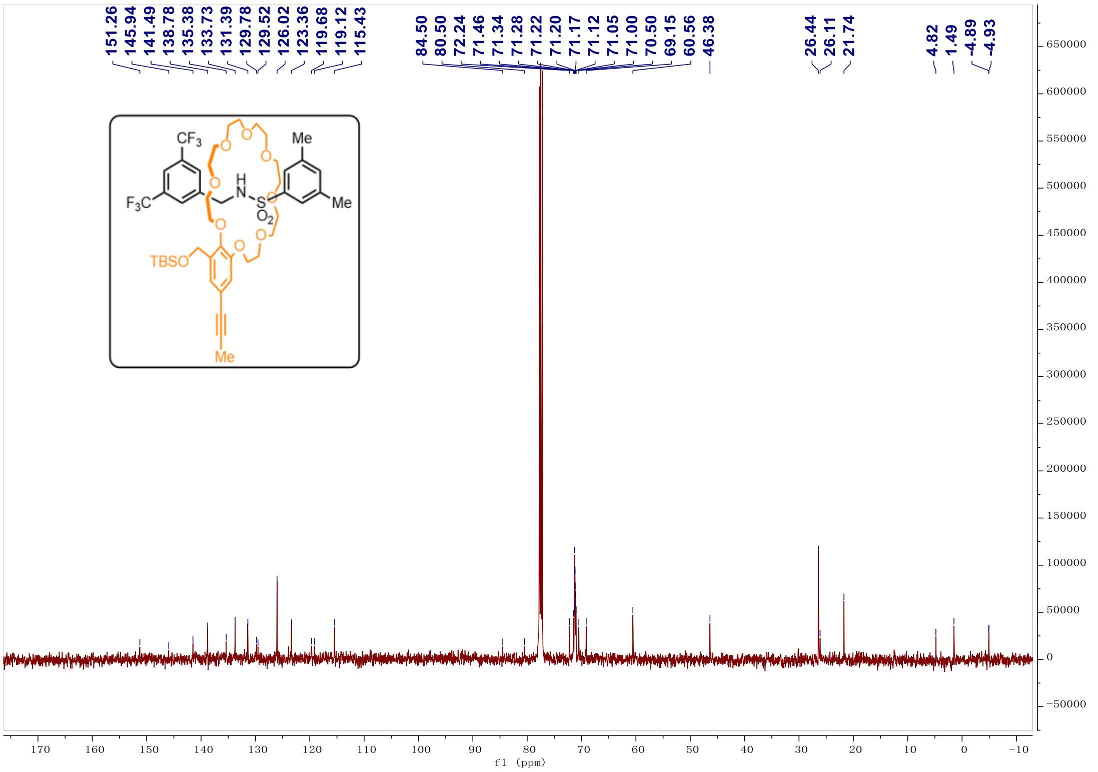

S14, ^{1}H NMR ($CDCl_3$, 500 MHz)

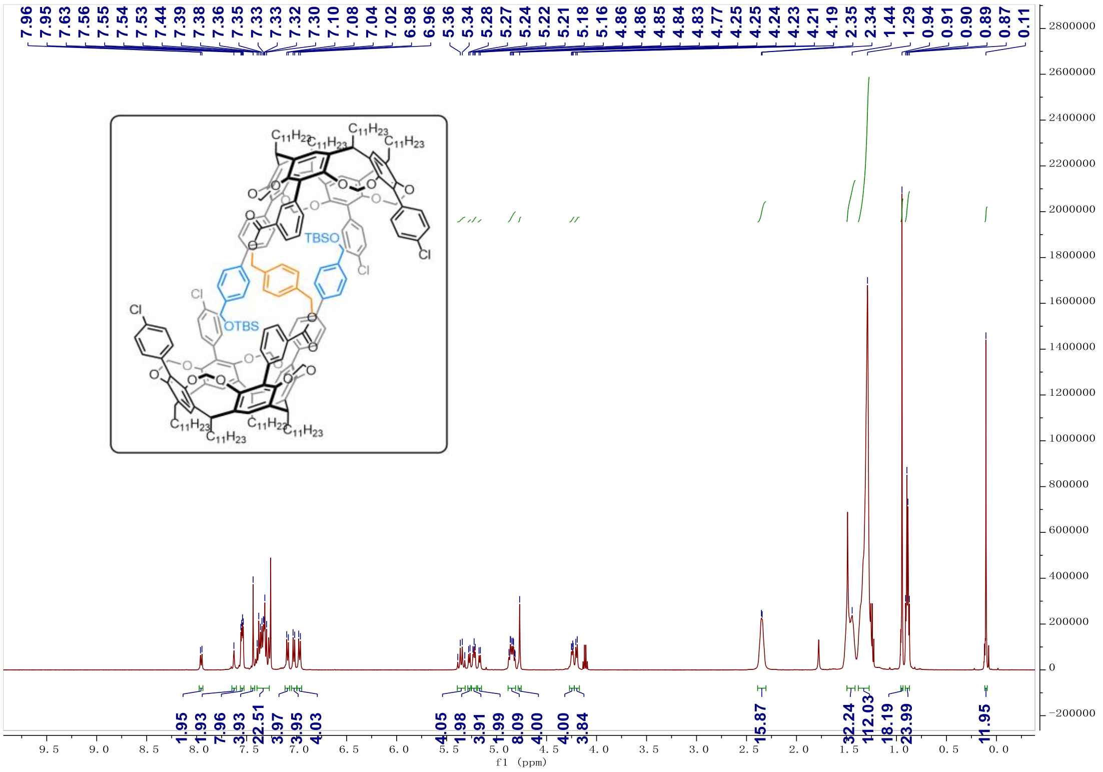

S14, ^{13}C NMR ($CDCl_3$, 126 MHz)

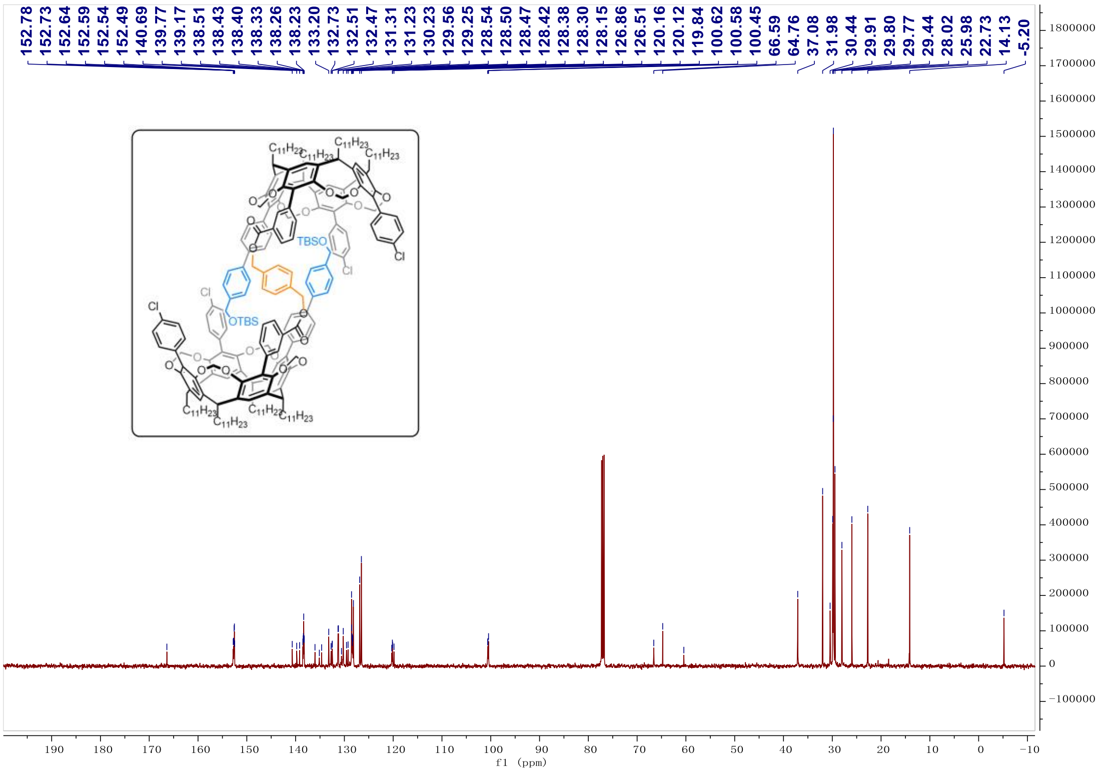

S15, ^{1}H NMR ($CDCl_3$, 500 MHz)

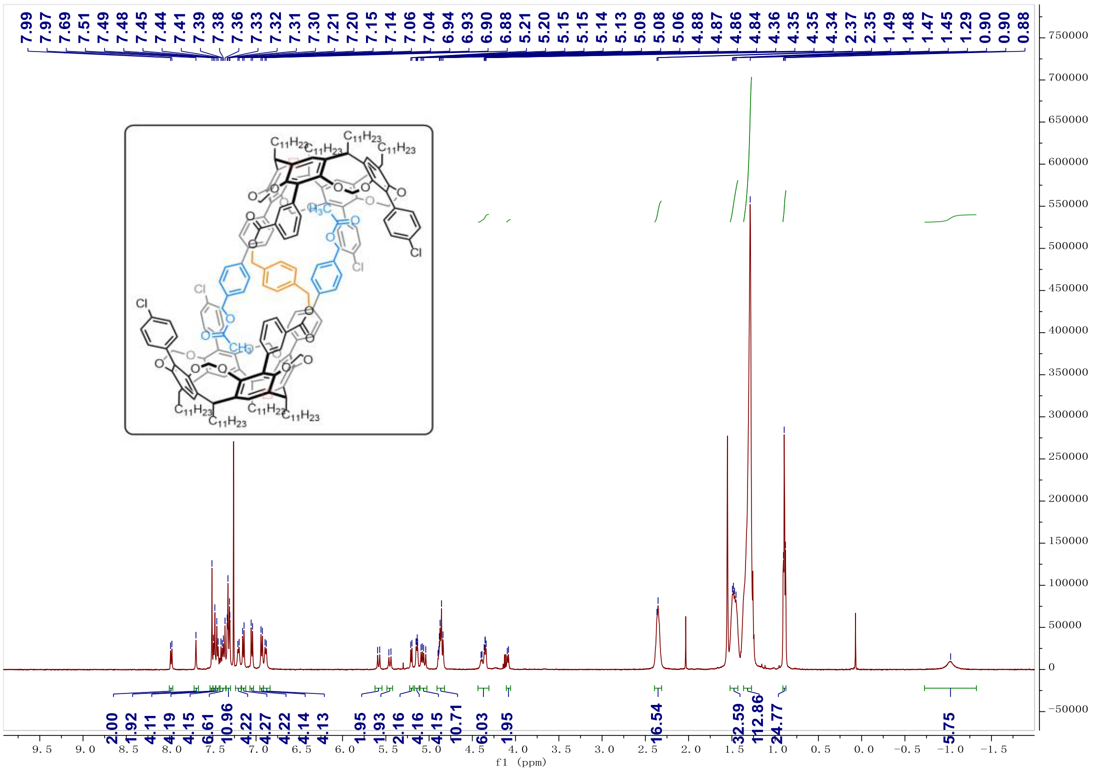

S15, ^{13}C NMR ($CDCl_3$, 126 MHz)

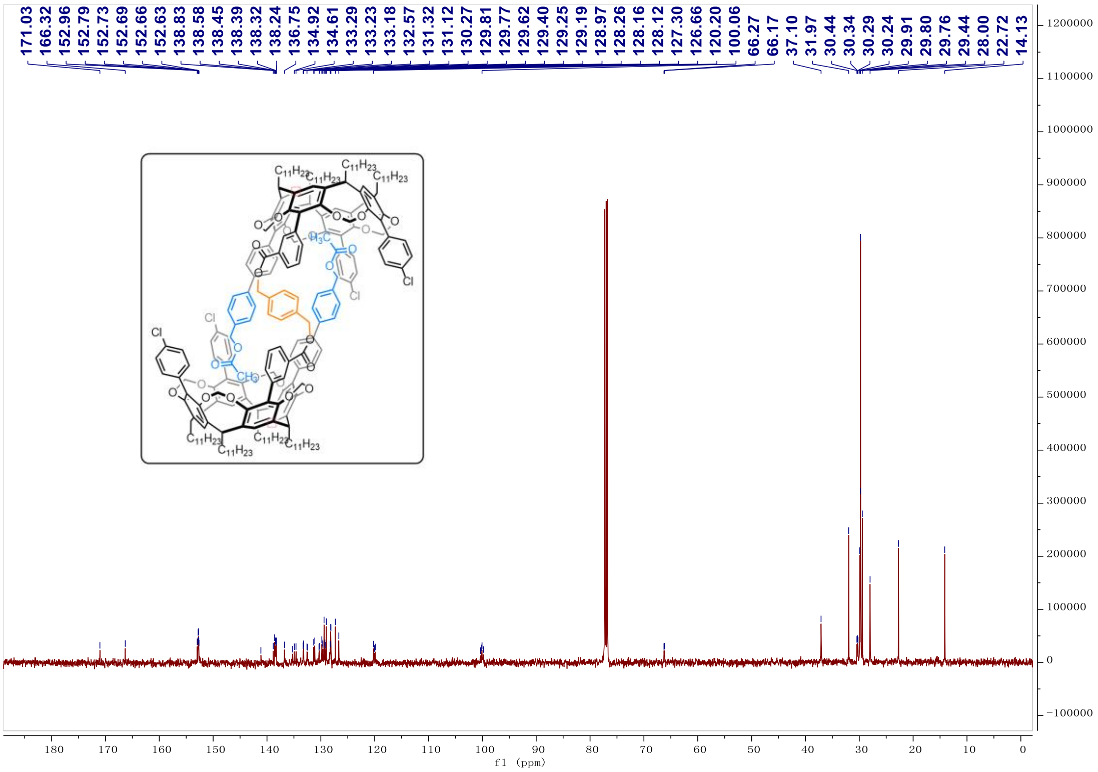

30, ^{1}H NMR (DMSO-d6, 400 MHz)

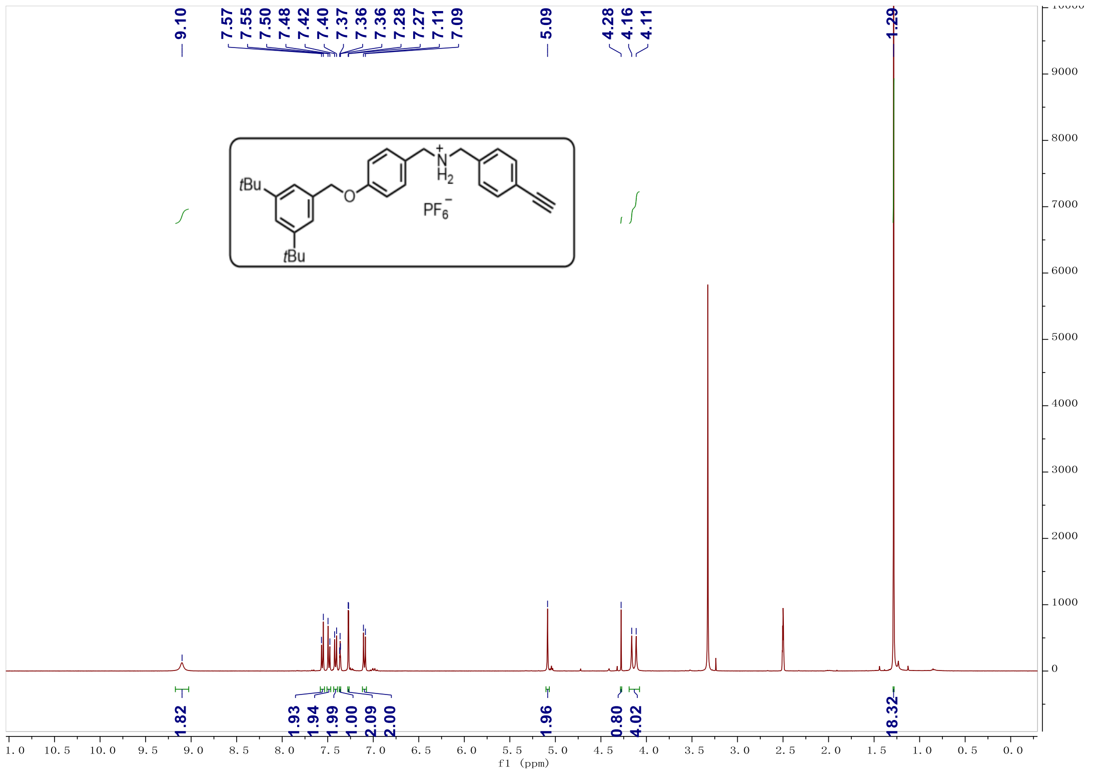

30, ^{13}C NMR (DMSO-d6, 126 MHz)

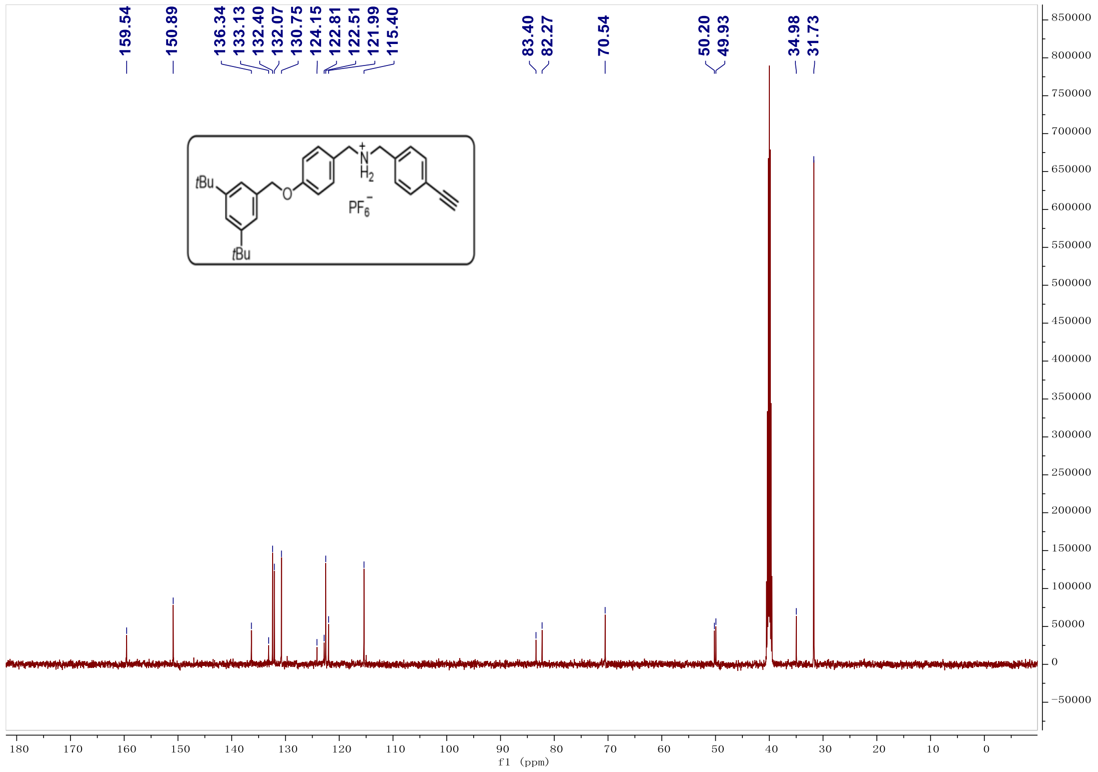

32, ^{1}H NMR ($CDCl_3$, 500 MHz)

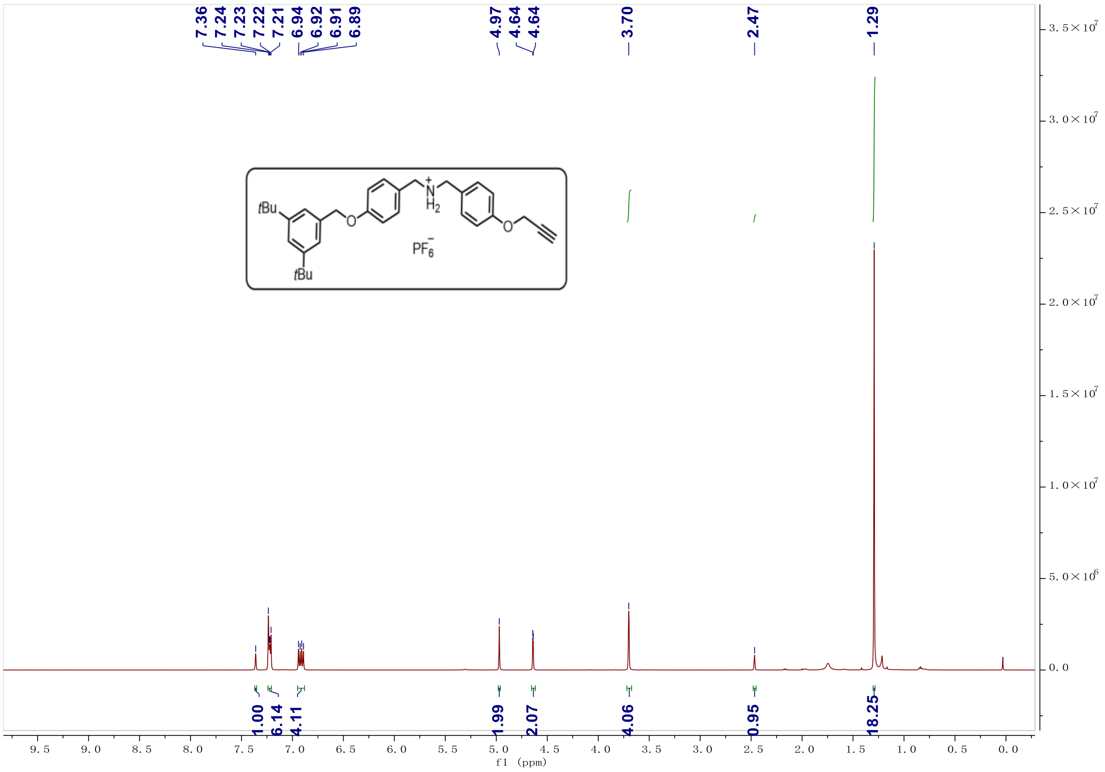

32, ^{13}C NMR ($CDCl_3$, 126 MHz)

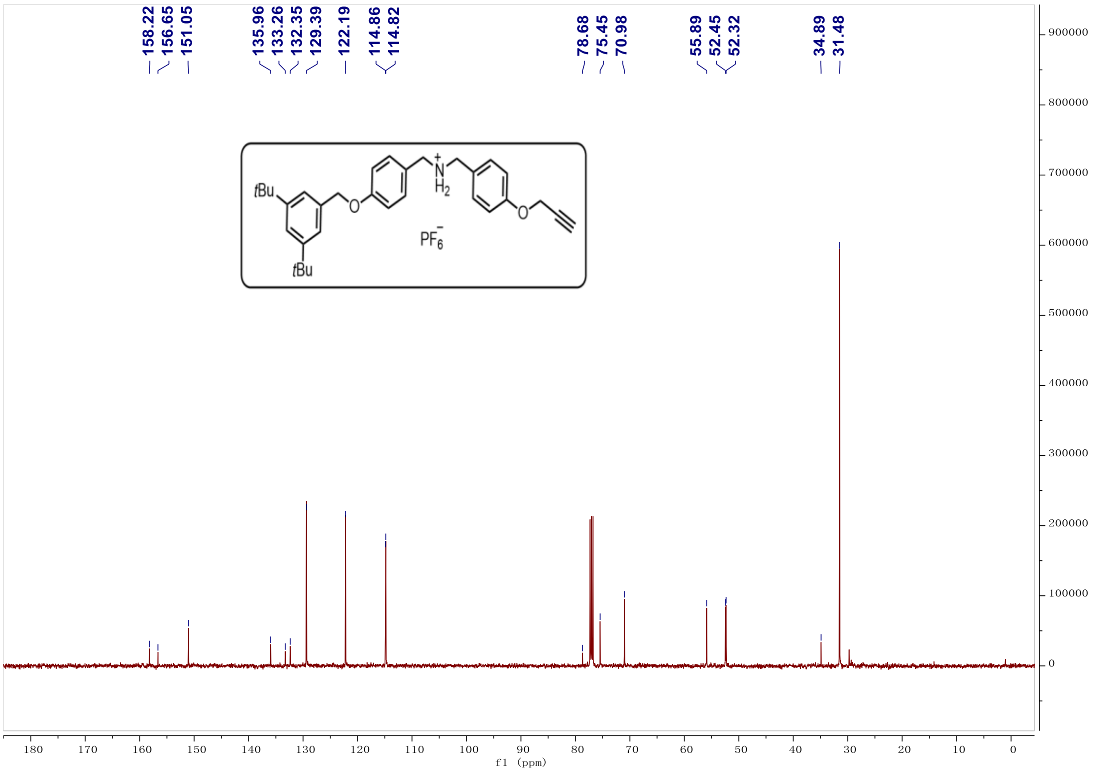

31, ^{1}H NMR (DMSO-d6, 500 MHz, 373K)

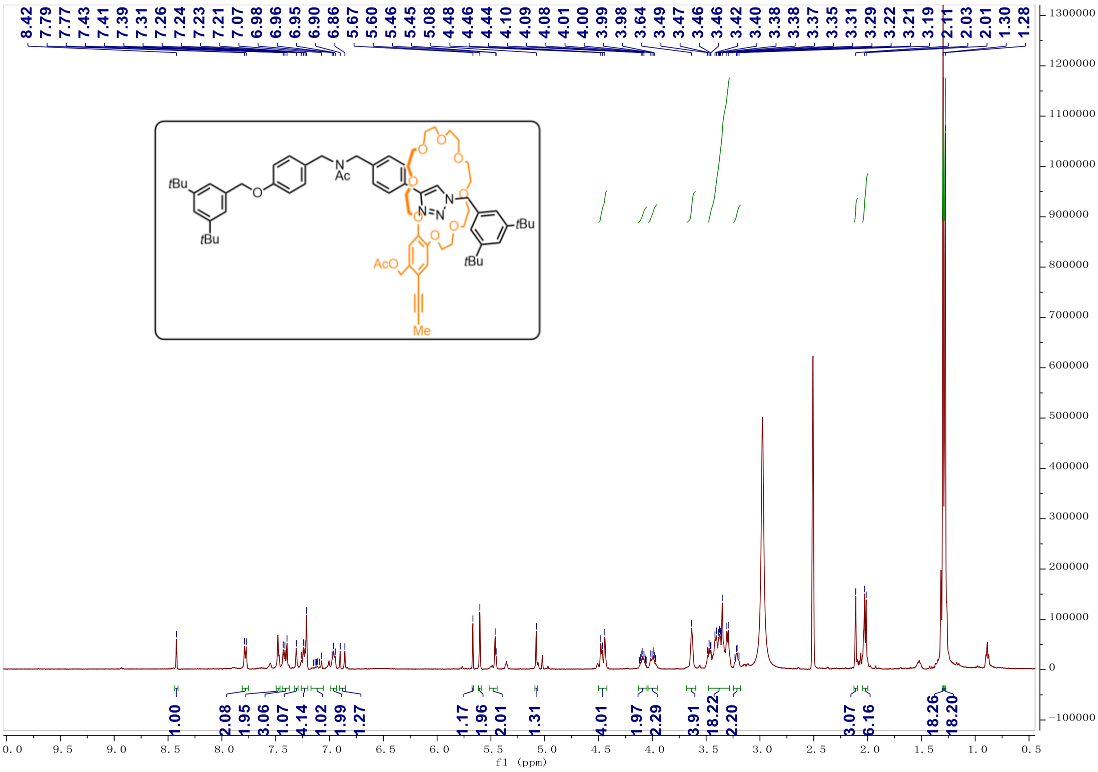

31, ^{13}C NMR (DMSO-d6, 126 MHz)

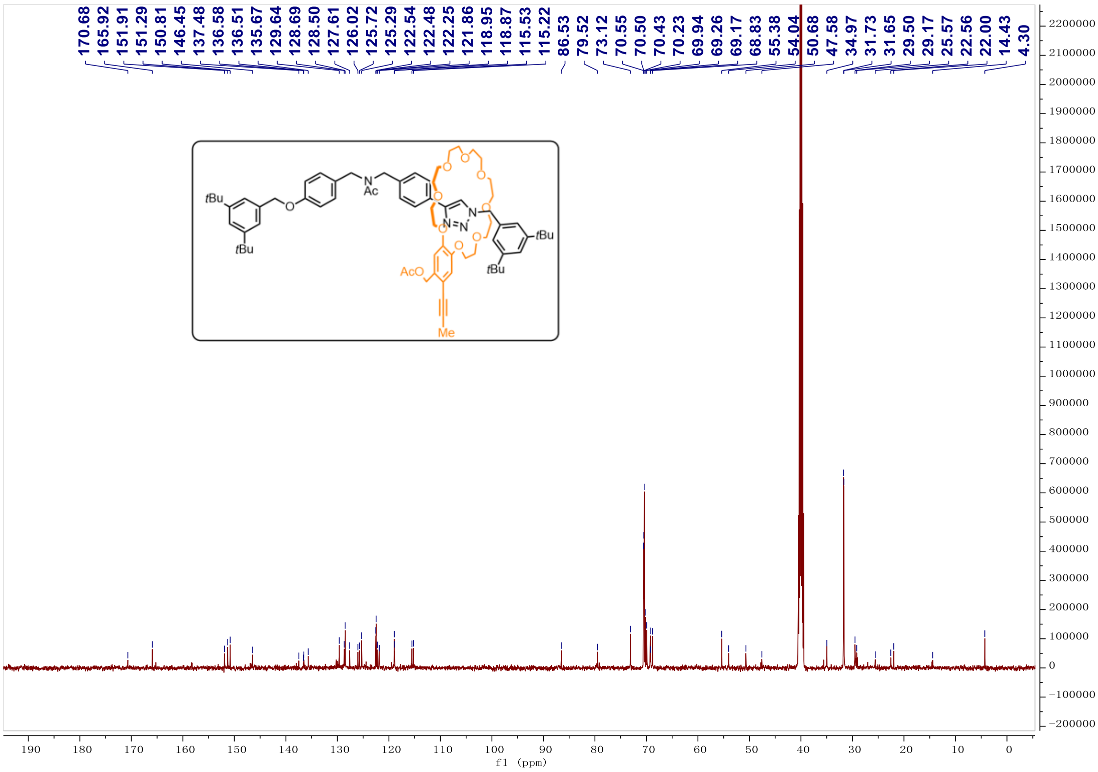

33, ^{1}H NMR (DMSO-d6, 500 MHz, 373K)

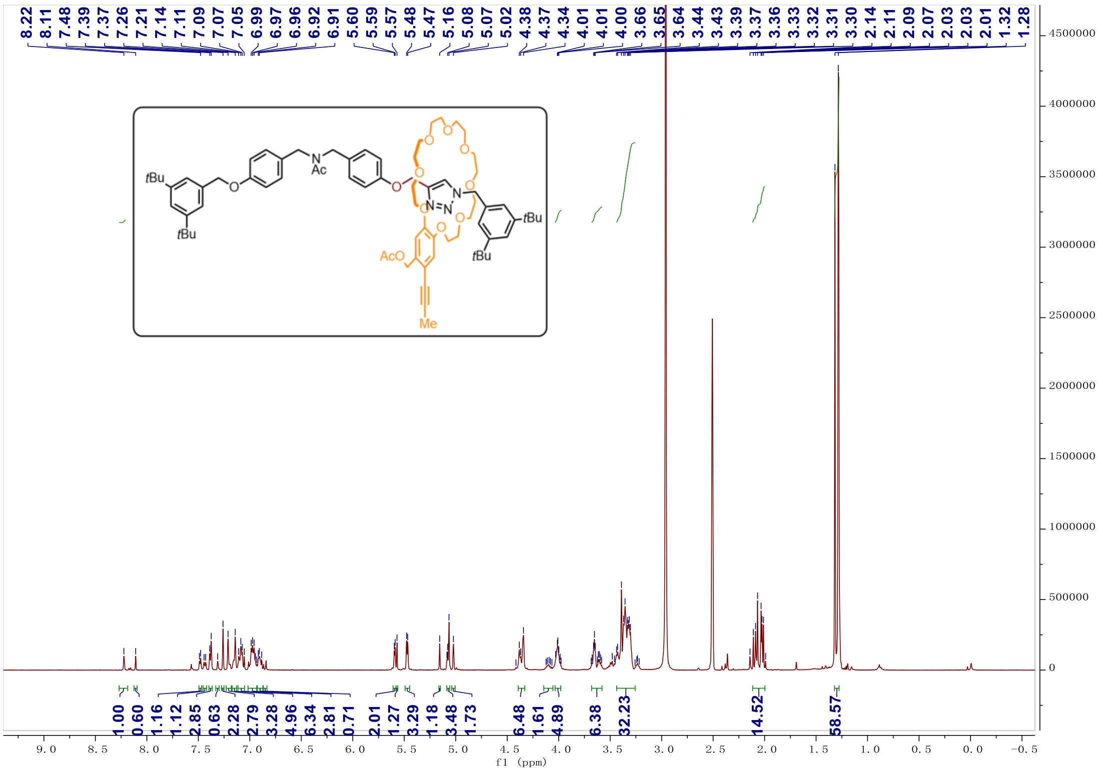

33, ^{13}C NMR (DMSO-d6, 126 MHz)

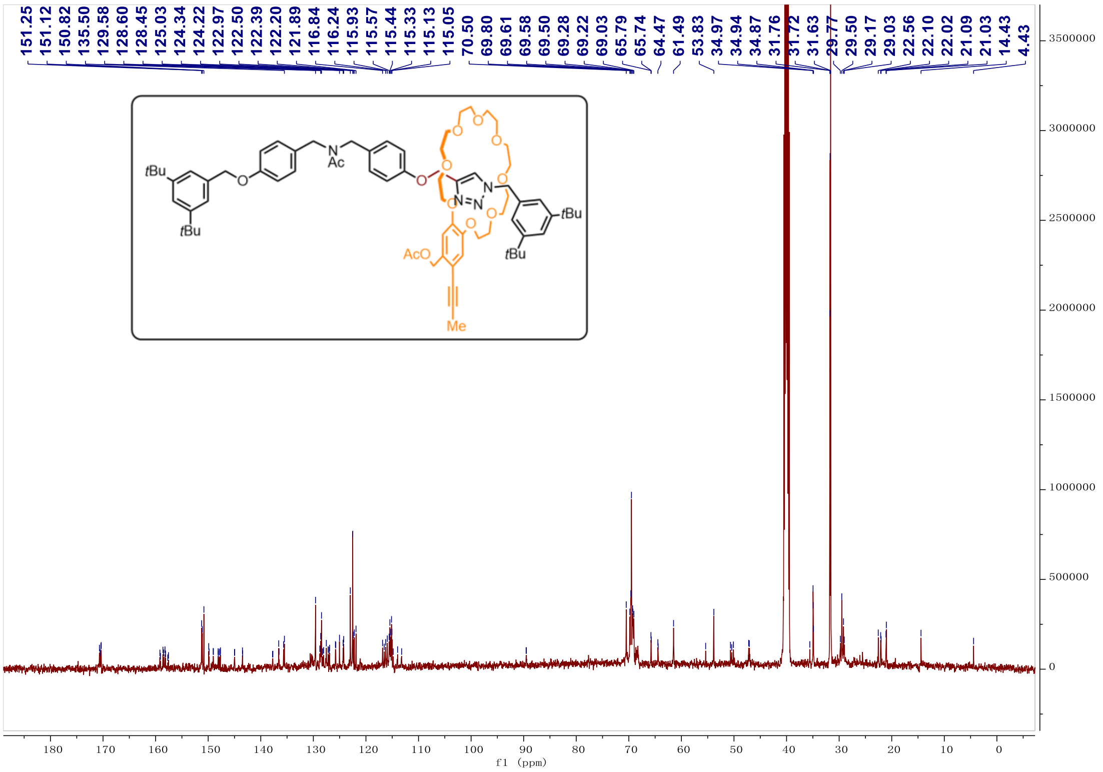

HPLC traces

HPLC analysis of racemic **S1**

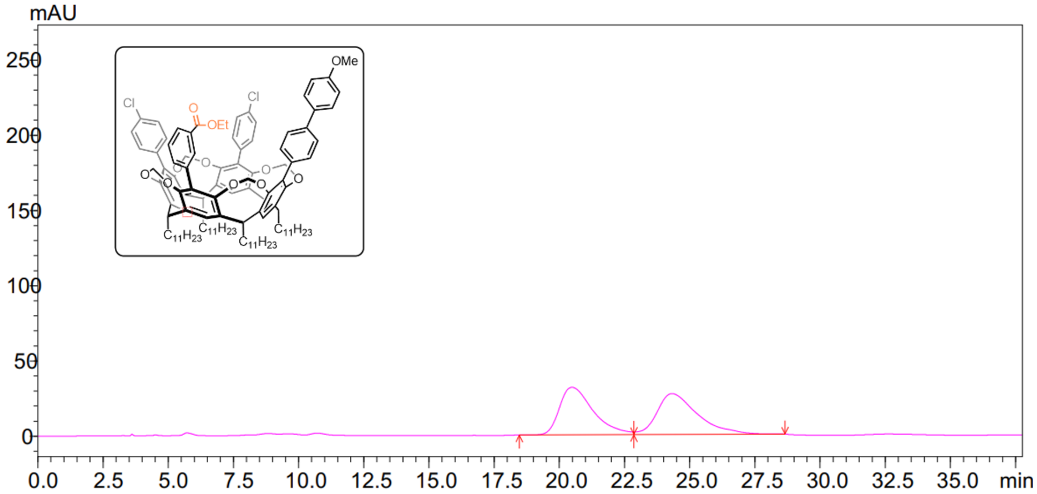

<Peak Table>

PDA Ch1 254nm

Peak#	Ret. Time	Area	Mark	Peak Start	Peak End	Area%
1	20.486	2839053	M	18.464	22.859	49.039
2	24.322	2950331	V M	22.859	28.651	50.961
Total		5789384				100.000

HPLC analysis of chiral **S1**

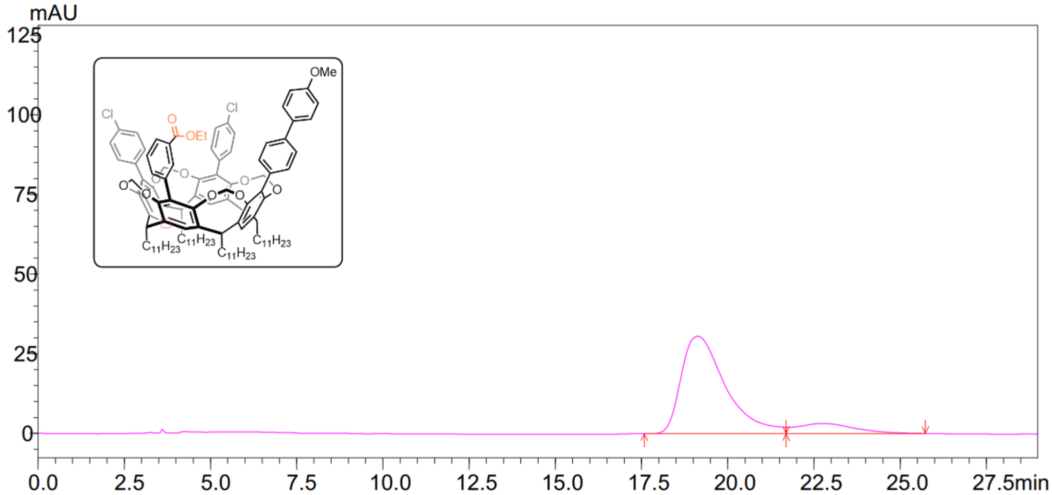

<Peak Table>

PDA Ch1 254nm

Peak#	Ret. Time	Area	Mark	Peak Start	Peak End	Area%
1	19.131	2736043	M	17.579	21.696	88.100
2	22.736	369578	V M	21.696	25.728	11.900
Total		3105621				100.000

HPLC analysis of racemic **S8**

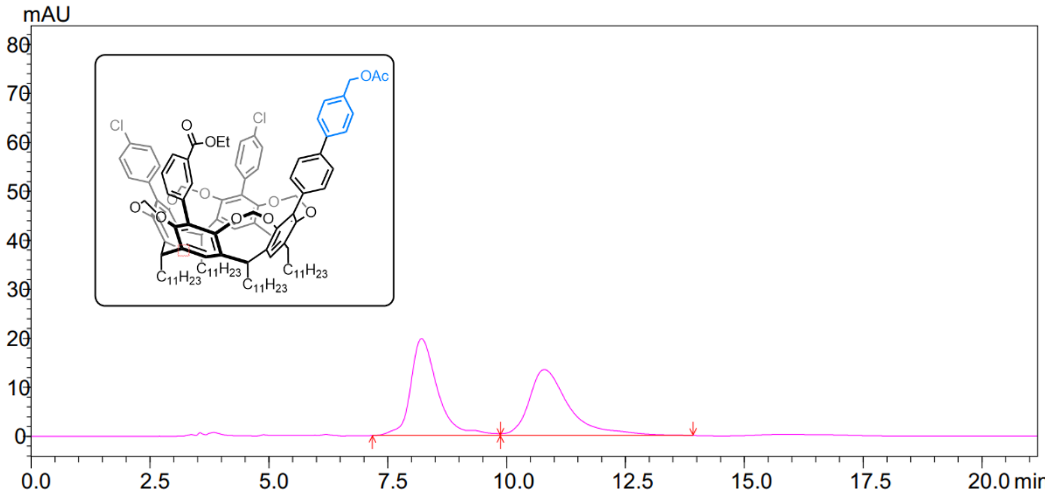

<Peak Table>

PDA Ch1 254nm

Peak#	Ret. Time	Area	Mark	Peak Start	Peak End	Area%
1	8.209	784475	M	7.179	9.867	49.923
2	10.794	786880	V M	9.867	13.920	50.077
Total		1571355				100.000

HPLC analysis of chiral **S8** (er = 93:7, from **Scheme 4**)

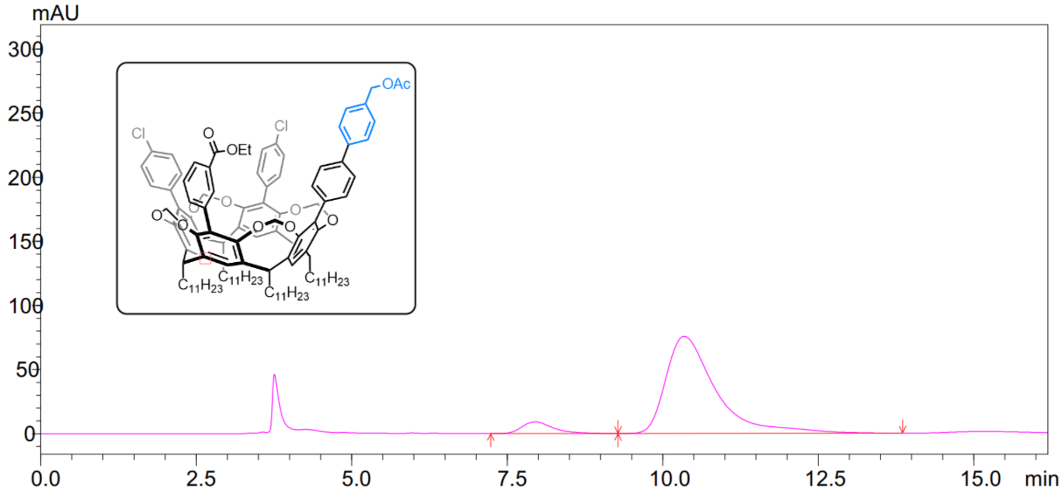

<Peak Table>

PDA Ch1 254nm

Peak#	Ret. Time	Area	Mark	Peak Start	Peak End	Area%
1	7.948	317776	M	7.232	9.280	7.028
2	10.346	4203778	V M	9.280	13.856	92.972
Total		4521554				100.000

HPLC analysis of racemic **S8**

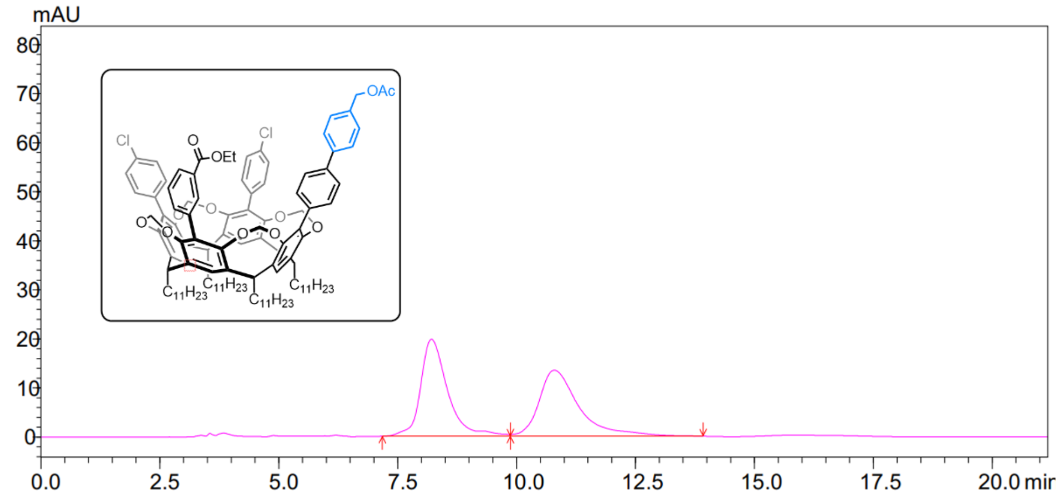

<Peak Table>

PDA Ch1 254nm

Peak#	Ret. Time	Area	Mark	Peak Start	Peak End	Area%
1	8.209	784475	M	7.179	9.867	49.923
2	10.794	786880	V M	9.867	13.920	50.077
Total		1571355				100.000

HPLC analysis of chiral **S8** (er = 98:2, **Scheme 5**)

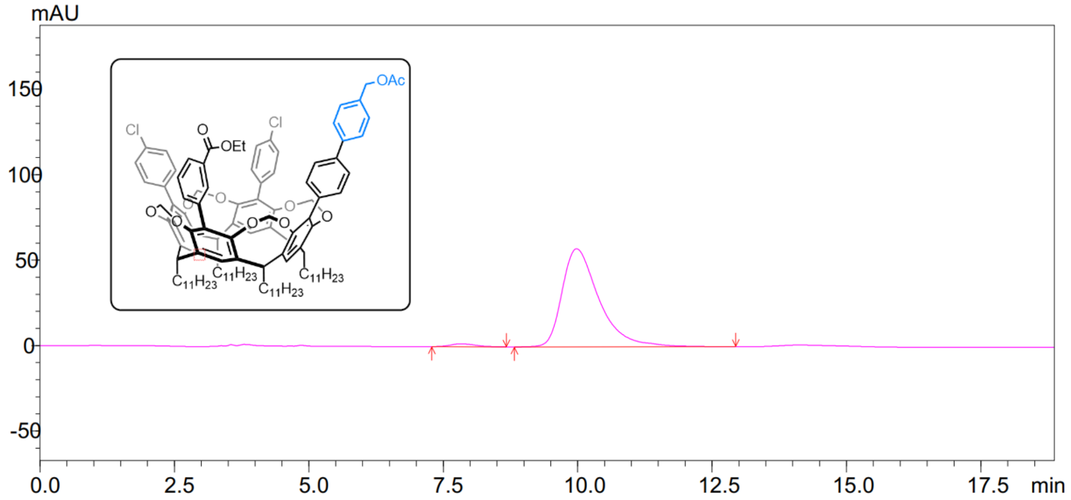

<Peak Table>

PDA Ch1 254nm

Peak#	Ret. Time	Area	Mark	Peak Start	Peak End	Area%
1	7.834	53267	M	7.285	8.672	1.894
2	9.980	2758451	M	8.821	12.939	98.106
Total		2811718				100.000

HPLC analysis of racemic **17**

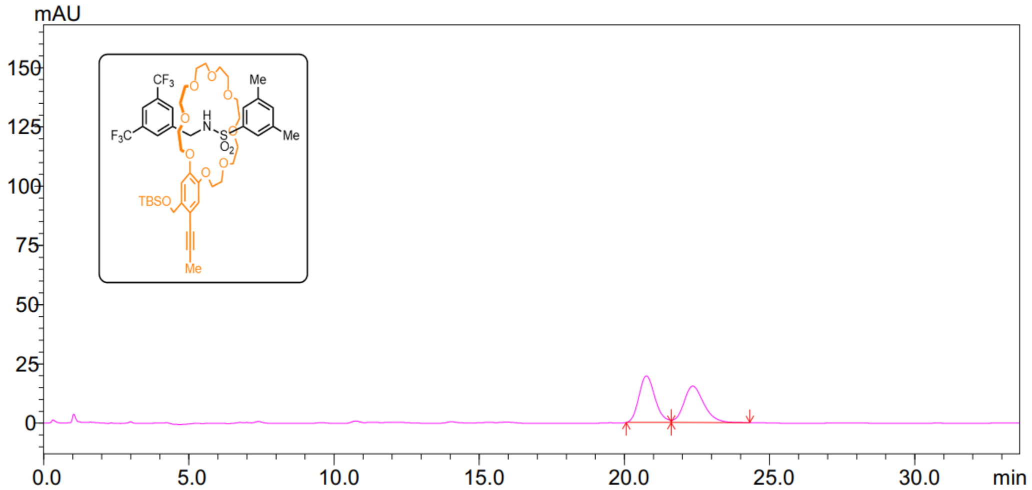

<Peak Table>

PDA Ch1 254nm

Ret. Time	Area	Height	Mark	Peak Start	Peak End	Area%
20.757	757778	19687	M	20.064	21.611	50.567
22.354	740794	15423	V M	21.611	24.320	49.433
	1498572	35110				100.000

HPLC analysis of chiral **17** (er = 90:10, **from Scheme 4**)

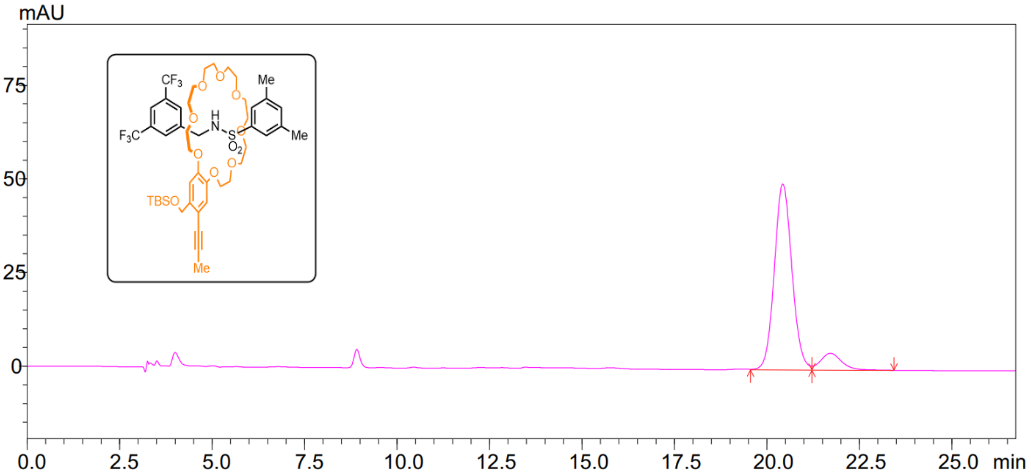

<Peak Table>

PDA Ch1 254nm

Peak#	Ret. Time	Area	Mark	Peak Start	Peak End	Area%
1	20.428	1644976	M	19.552	21.216	90.181
2	21.714	179114	V M	21.216	23.435	9.819
Total		1824090				100.000

HPLC analysis of racemic **17**

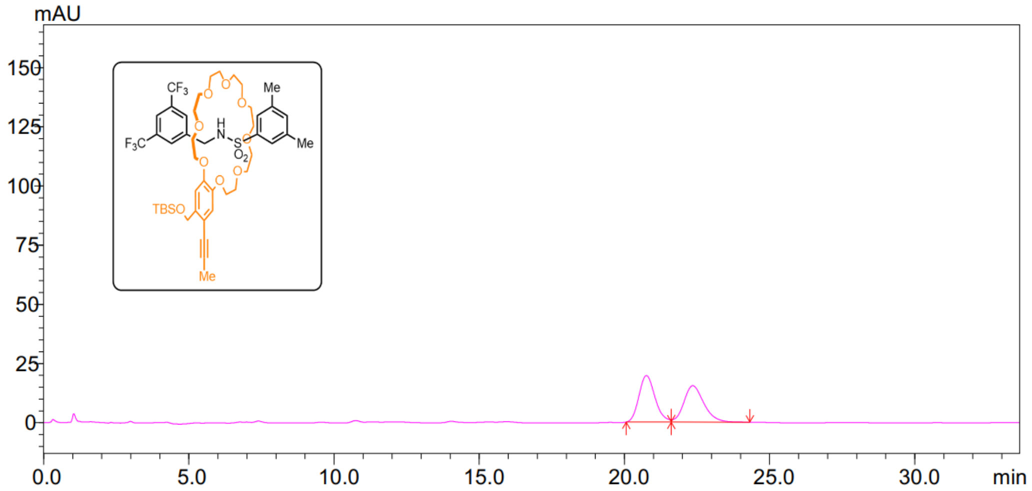

<Peak Table>

PDA Ch1 254nm

Ret. Time	Area	Height	Mark	Peak Start	Peak End	Area%
20.757	757778	19687	M	20.064	21.611	50.567
22.354	740794	15423	V M	21.611	24.320	49.433
	1498572	35110				100.000

HPLC analysis of chiral **17** (er = 93:7, **from Scheme 5**)

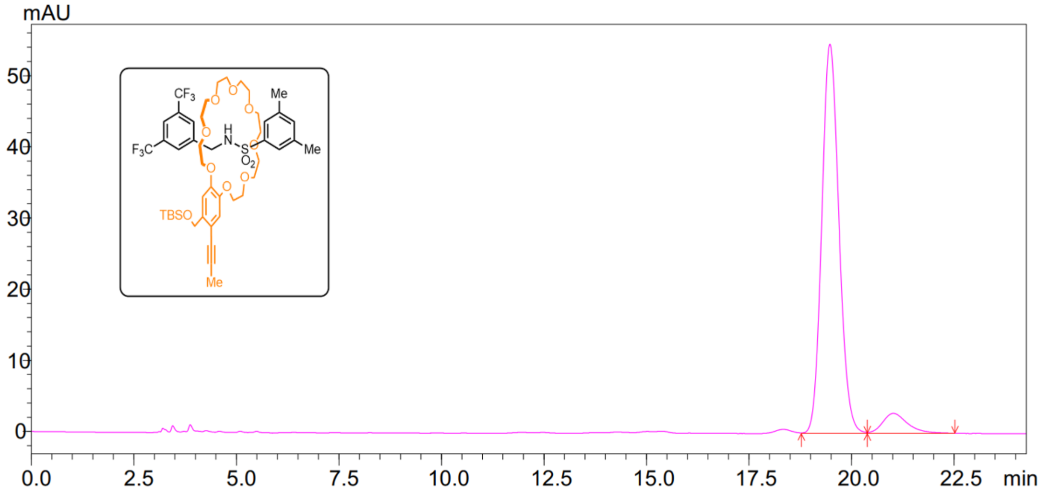

<Peak Table>

PDA Ch1 254nm

Peak#	Ret. Time	Area	Mark	Peak Start	Peak End	Area%
1	19.470	1597706	M	18.773	20.384	93.048
2	21.021	119366	V M	20.384	22.517	6.952
Total		1717072				100.000

HPLC analysis of racemic **23**

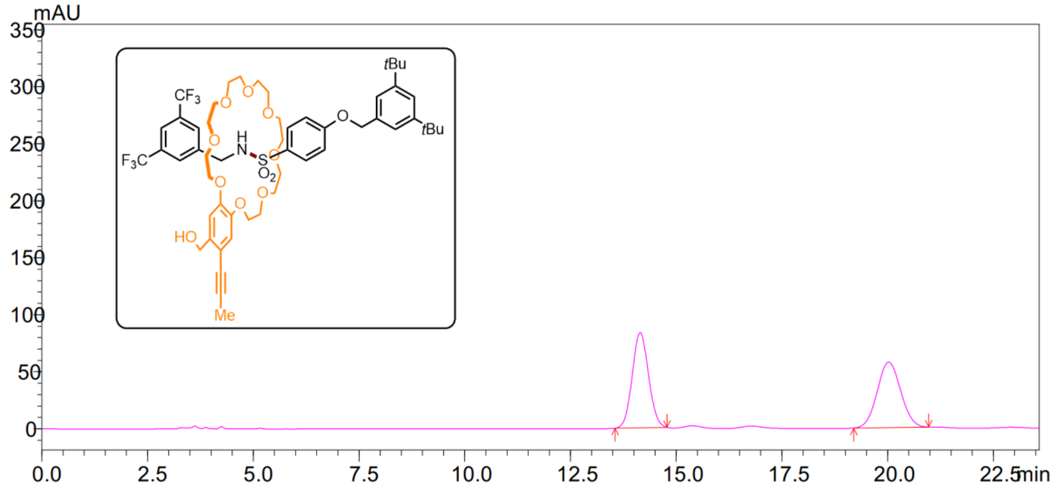

<Peak Table>

PDA Ch1 254nm

Peak#	Ret. Time	Area	Mark	Peak Start	Peak End	Area%
1	14.148	2242579	M	13.557	14.784	50.240
2	20.024	2221181	M	19.200	20.971	49.760
Total		4463760				100.000

HPLC analysis of chiral **23**

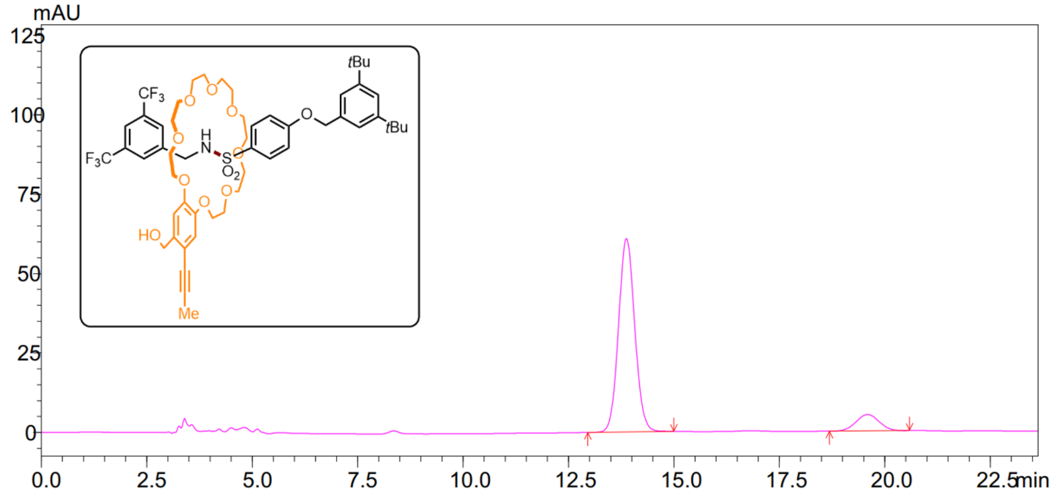

<Peak Table>

PDA Ch1 254nm

Peak#	Ret. Time	Area	Mark	Peak Start	Peak End	Area%
1	13.873	1633156	M	12.960	14.997	89.321
2	19.591	195264	M	18.688	20.587	10.679
Total		1828420				100.000

HPLC analysis of racemic **24**

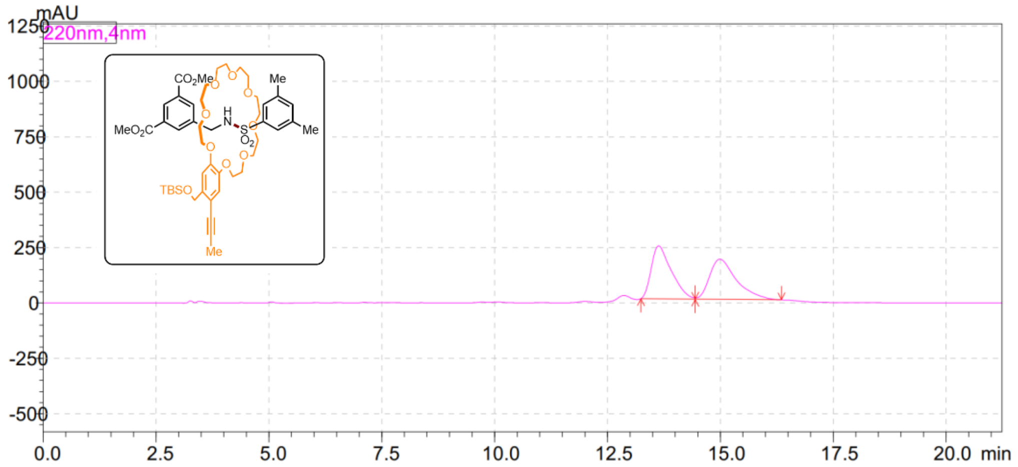

<Peak Table>

PDA Ch1 220nm

Peak#	Ret. Time	Area	Mark	Peak Start	Peak End	Area%
1	13.632	7407560	M	13.237	14.443	49.622
2	14.989	7520391	V M	14.443	16.352	50.378
Total		14927951				100.000

HPLC analysis of chiral **24**

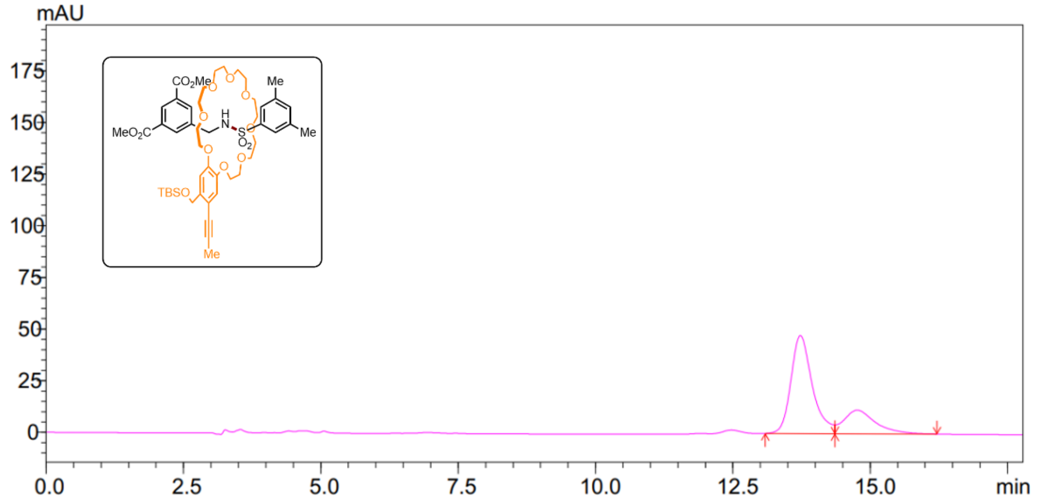

<Peak Table>

PDA Ch1 220nm

Peak#	Ret. Time	Area	Mark	Peak Start	Peak End	Area%
1	13.728	4481896	M	13.248	14.336	78.319
2	14.741	1240728	V M	14.336	15.339	21.681
Total		5722623				100.000

HPLC analysis of racemic **25**

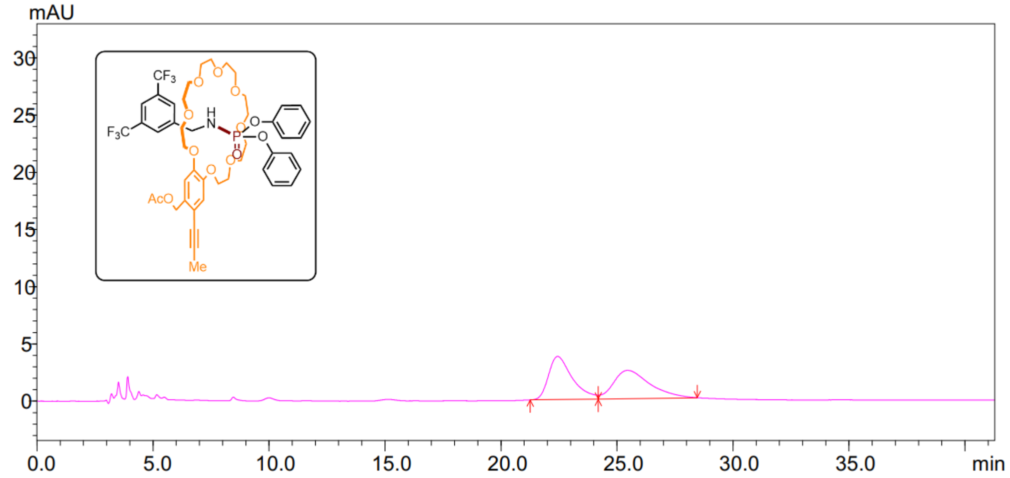

<Peak Table>

PDA Ch1 254nm

Peak#	Ret. Time	Area	Mark	Peak Start	Peak End	Area%
1	22.442	268938	M	21.259	24.192	49.864
2	25.463	270404	V M	24.192	28.459	50.136
Total		539342				100.000

HPLC analysis of chiral **25**

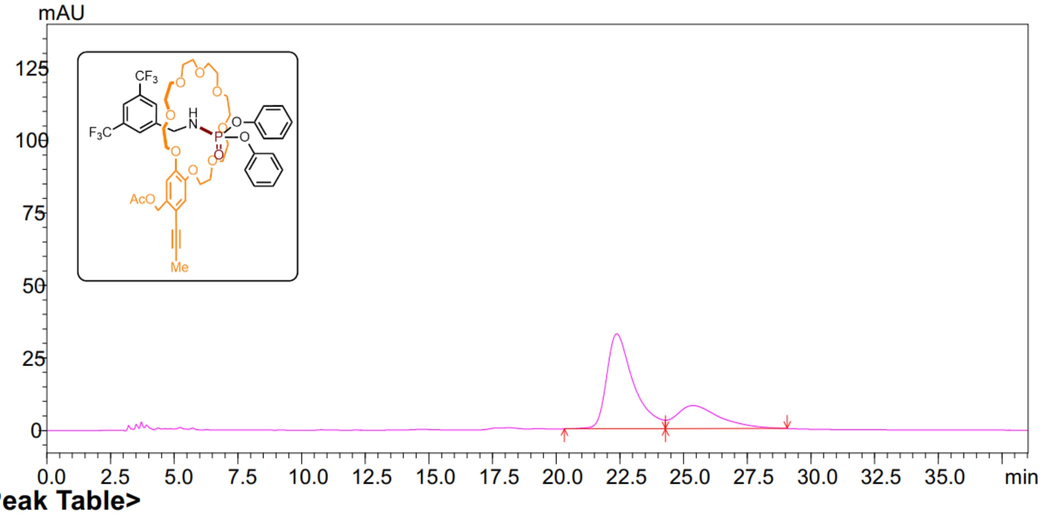

<Peak Table>

PDA Ch1 254nm

Peak#	Ret. Time	Area	Mark	Peak Start	Peak End	Area%
1	22.376	2403818	M	20.320	24.288	71.690
2	25.365	949277	V M	24.288	29.067	28.310
Total		3353095				100.000

HPLC analysis of racemic **26**

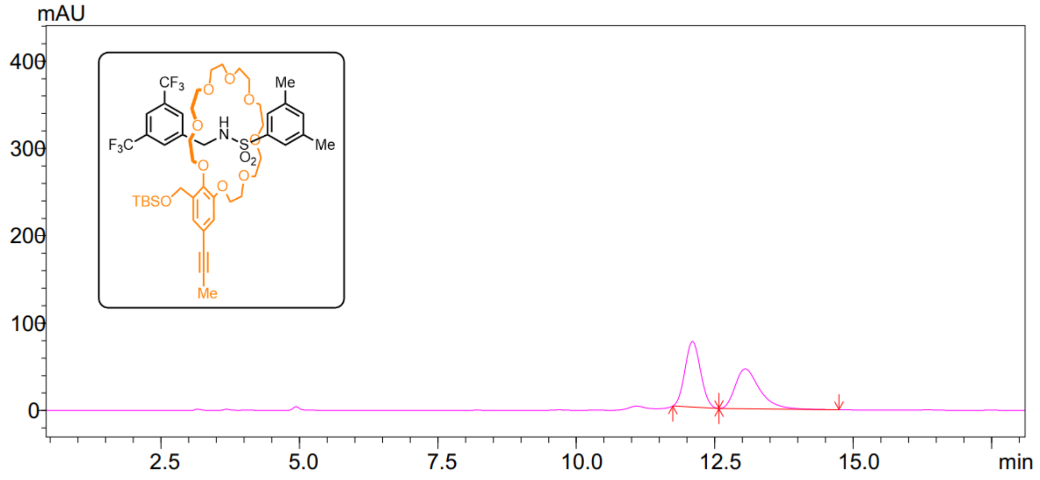

<Peak Table>

PDA Ch1 254nm

Peak#	Ret. Time	Area	Mark	Peak Start	Peak End	Area%
1	12.098	1466232	M	11.744	12.576	51.099
2	13.049	1403177	M	12.576	14.741	48.901
Total		2869409				100.000

HPLC analysis of chiral **26**

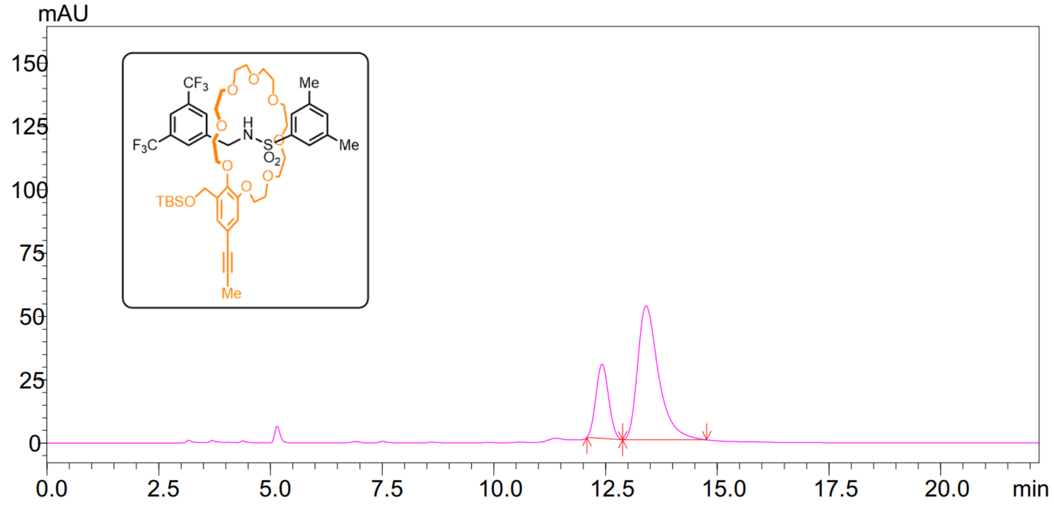

<Peak Table>

PDA Ch1 254nm

Peak#	Ret. Time	Area	Mark	Peak Start	Peak End	Area%
1	12.422	575710	M	12.085	12.885	25.001
2	13.406	1727036	M	12.885	14.763	74.999
Total		2302746				100.000

HPLC analysis of racemic **31**

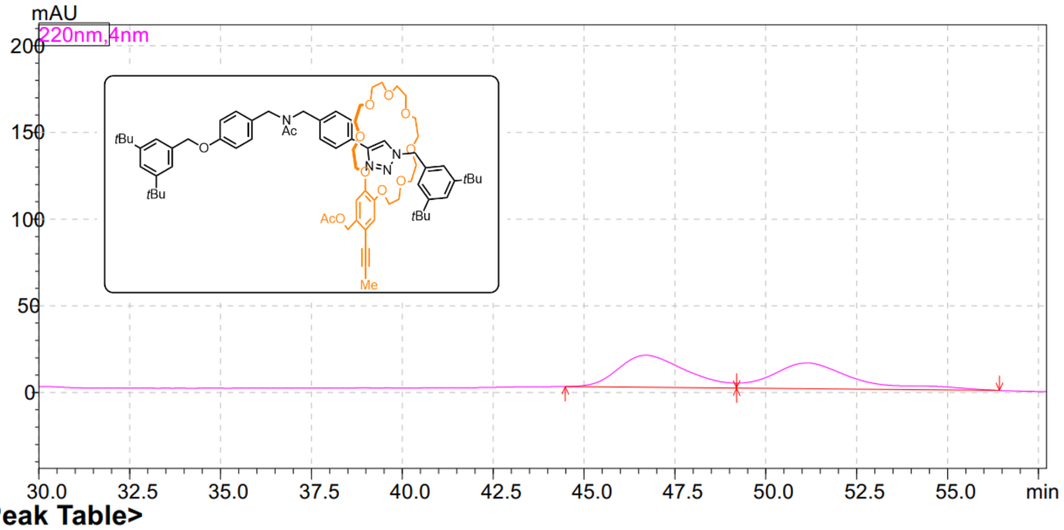

<Peak Table>

PDA Ch1 220nm

Peak#	Ret. Time	Area	Mark	Peak Start	Peak End	Area%
1	46.701	2408103	M	44.480	49.195	50.627
2	51.140	2348497	V M	49.195	56.427	49.373
Total		4756600				100.000

HPLC analysis of chiral **31**

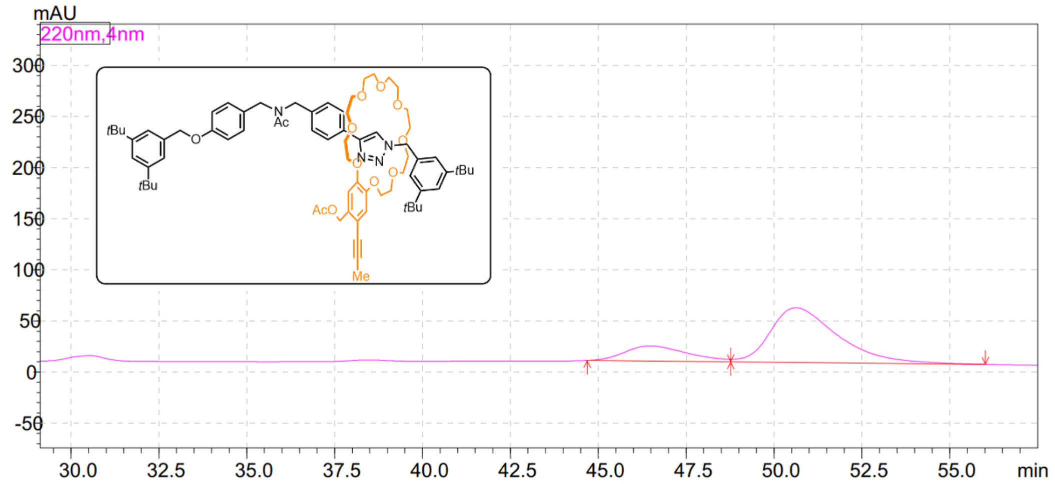

<Peak Table>

PDA Ch1 220nm

Peak#	Ret. Time	Area	Mark	Peak Start	Peak End	Area%
1	46.489	1835308	M	44.683	48.768	20.403
2	50.625	7159931	V M	48.768	56.011	79.597
Total		8995239				100.000

HPLC analysis of racemic **33**

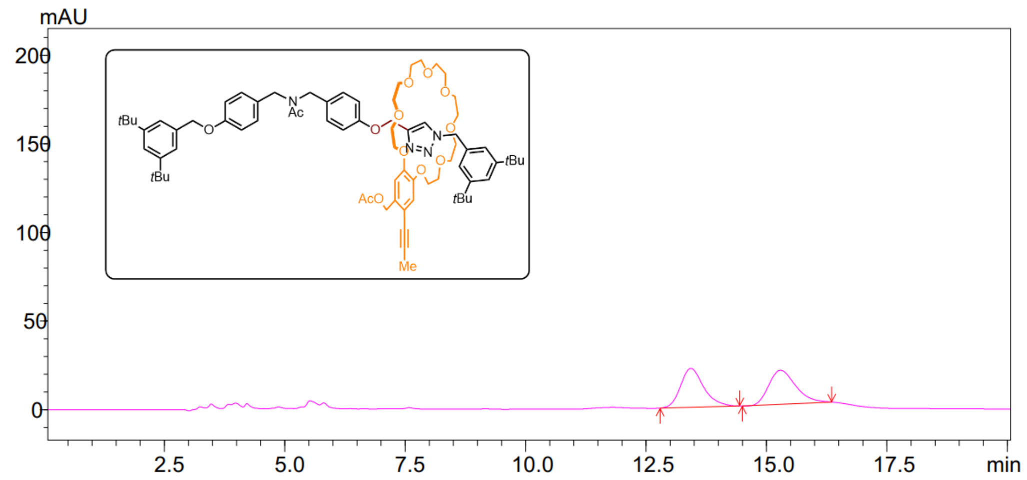

<Peak Table>

PDA Ch1 254nm

Peak#	Ret. Time	Area	Mark	Peak Start	Peak End	Area%
1	13.429	736507	M	12.789	14.443	49.675
2	15.290	746144	M	14.496	16.352	50.325
Total		1482651				100.000

HPLC analysis of chiral **33** (er = 80:20, three steps from **13**, **Scheme 6**)

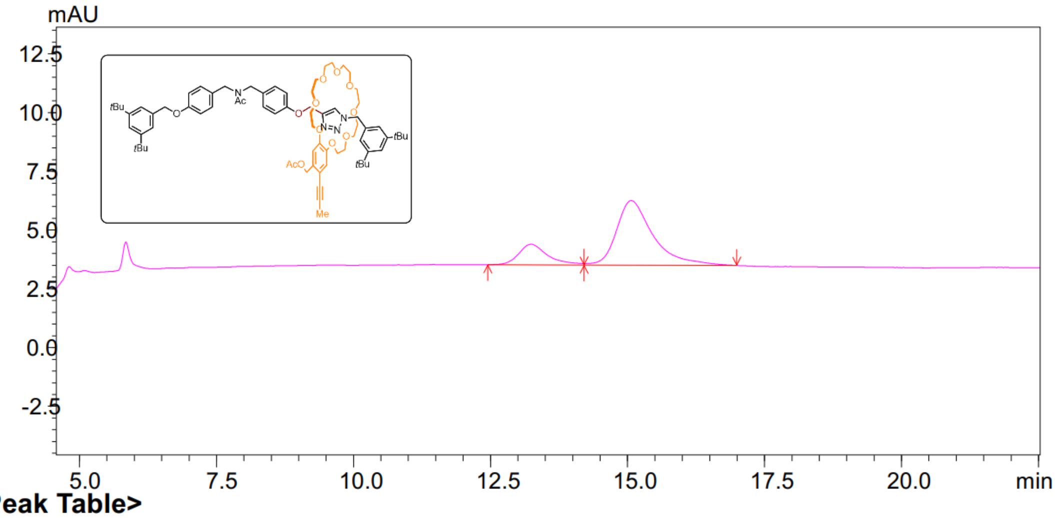

<Peak Table>

PDA Ch1 254nm

Peak#	Ret. Time	Area	Mark	Peak Start	Peak End	Area%
1	13.238	32573	M	12.448	14.208	20.283
2	15.068	128015	V M	14.208	16.992	79.717
Total		160587				100.000

HPLC analysis of racemic **33**

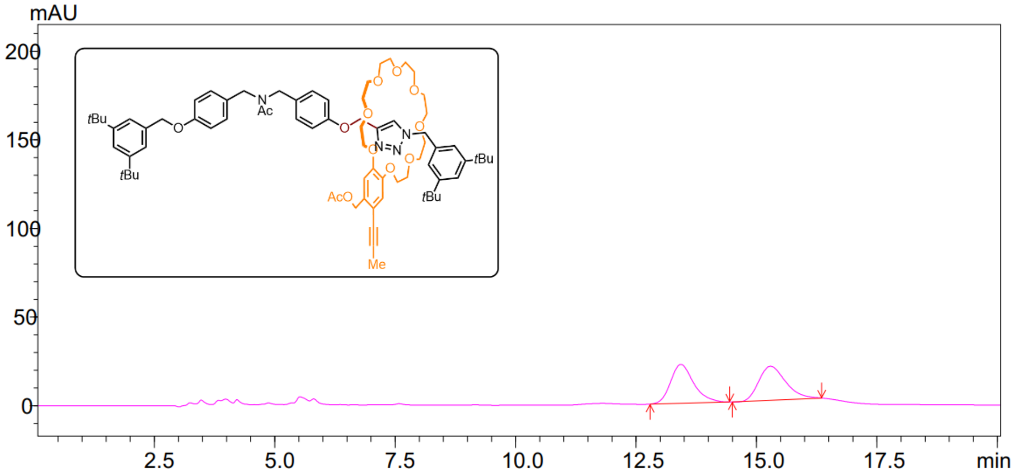

<Peak Table>

PDA Ch1 254nm

Peak#	Ret. Time	Area	Mark	Peak Start	Peak End	Area%
1	13.429	736507	M	12.789	14.443	49.675
2	15.290	746144	M	14.496	16.352	50.325
Total		1482651				100.000

HPLC analysis of chiral **33** (er = 81:19, three steps from **12**, **Scheme 6**)

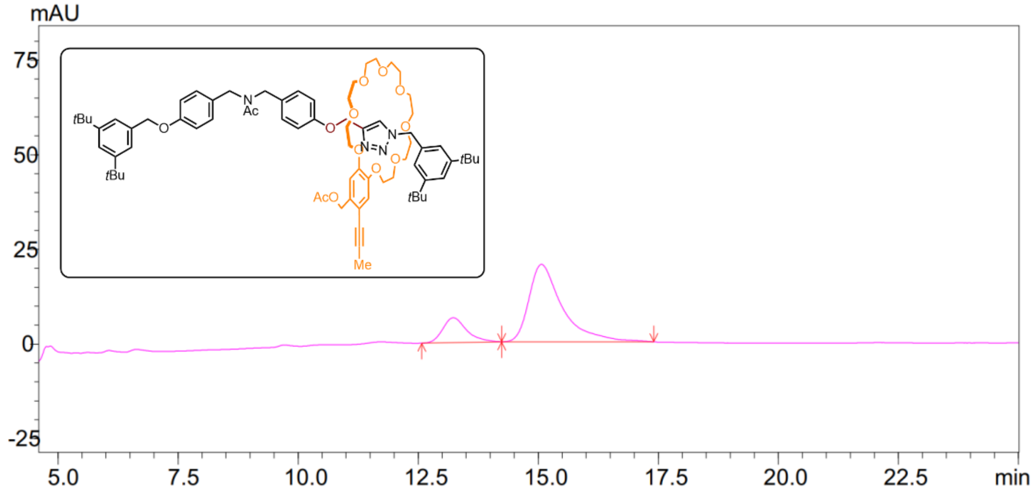

<Peak Table>

PDA Ch1 254nm

Peak#	Ret. Time	Area	Mark	Peak Start	Peak End	Area%
1	13.232	46885	M	12.576	14.240	18.719
2	15.069	203577	M	14.261	16.565	81.281
Total		250462				100.000

X-ray crystallography data and CCDC deposition number

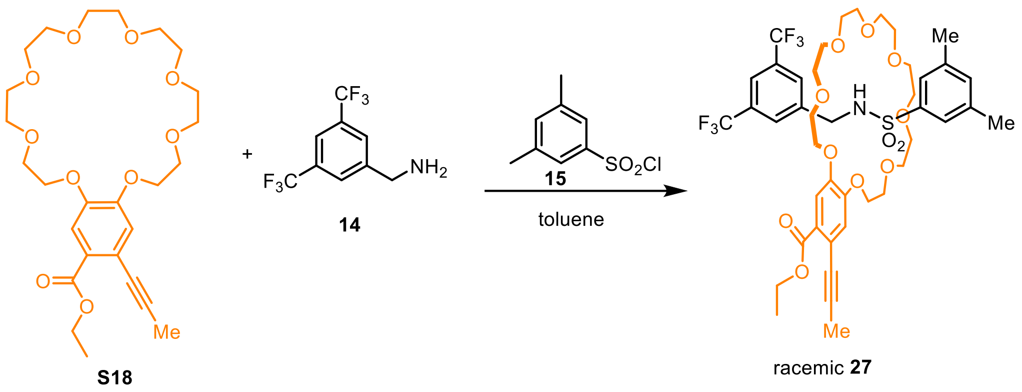

The preparation of racemic **27**: To a solution of **S18** (6 mg, 0.0118 mmol) in 1 mL dry toluene was added **14** (11 mg, 0.045 mmol, 4 equiv.) and **15** (5 mg, 0.0245 mmol). The reaction stirred at room temperature for overnight. The reaction mixture was diluted with 10 mL ethyl acetate and washed with saturated NaCl. The combined organic layers were dried with Na_2SO_4 and concentrated *in vacuo* to afford the crude product. The crude product purified by flash chromatography using ethyl acetate/hexane(1:1) as eluent to give racemic sample **27** as light-yellow solid.

The resolution of racemic **27** by chiral HPLC: Daicel Chiralpak IF, hexane/*iso*-propanol = 80: 20, 1.0 mL/min, temperature = 40 °C, λ = 272 nm, retention time: 21.2 min and 30.9 min.

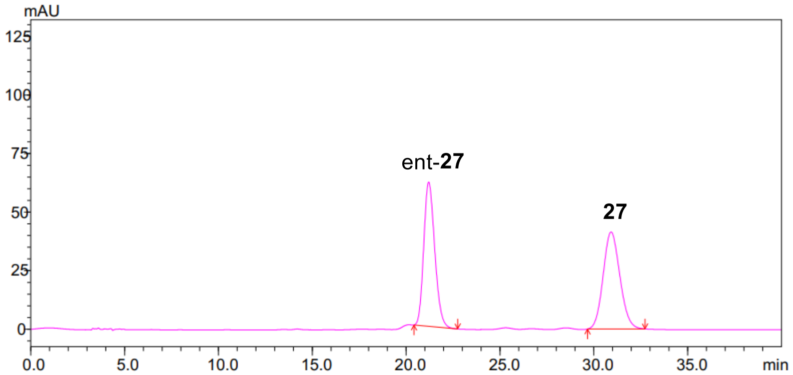

<Peak Table>

PDA Ch1 272nm

Peak#	Ret. Time	Area	Mark	Peak Start	Peak End	Area%
1	21.204	2507364	M	20.416	22.752	49.384
2	30.922	2569926	M	29.664	32.725	50.616
Total		5077290				100.000

Conditions for crystallization: 1.6 mg **27** (retention time = 30.9 min) was isolated by chiral HPLC under the optimized HPLC conditions. The crystal of **27** was obtained by slow evaporation of its solution in isopropanol.

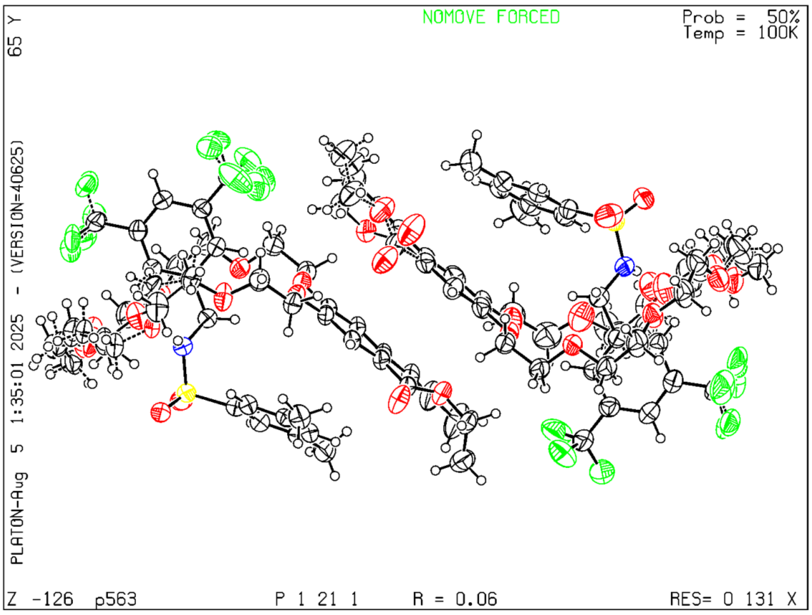

Deposition number of crystal of **27**: 2481669

Crystal data and structure refinement

Identification code	p563	
Empirical formula	C43 H53 F6 N O12 S	
Formula weight	921.92	
Temperature	100.00 K	
Wavelength	1.54178 Å	
Crystal system	Monoclinic	
Space group	P 1 21 1	
Unit cell dimensions	a = 8.9863(5) Å	a= 90°.
	b = 20.4401(10) Å	b= 92.491(3)°.
	c = 24.4363(11) Å	g = 90°.
Volume	4484.2(4) $Å^3$	
Z	4	
Density (calculated)	1.366 Mg/m^3	
Absorption coefficient	1.399 mm^{-1}	
F(000)	1936	
Crystal size	0.1 x 0.06 x 0.051 mm^3	
Theta range for data collection	2.819 to 66.596°.	
Index ranges	-10<=h<=10, -24<=k<=24, -29<=l<=29	
Reflections collected	168171	
Independent reflections	15860 [R(int) = 0.1228]	
Completeness to theta = 66.596°	100.0 %	
Absorption correction	Semi-empirical from equivalents	
Max. and min. transmission	0.7062 and 0.6205	
Refinement method	Full-matrix least-squares on F^2	
Data / restraints / parameters	15860 / 1023 / 1426	
Goodness-of-fit on F^2	1.020	
Final R indices [I>2sigma(I)]	R1 = 0.0609, wR2 = 0.1613	
R indices (all data)	R1 = 0.0788, wR2 = 0.1790	
Absolute structure parameter	-0.011(11)	
Extinction coefficient	n/a	
Largest diff. peak and hole	0.725 and -0.203 e.$Å^{-3}$	

The chemical correlation between 17 and 27

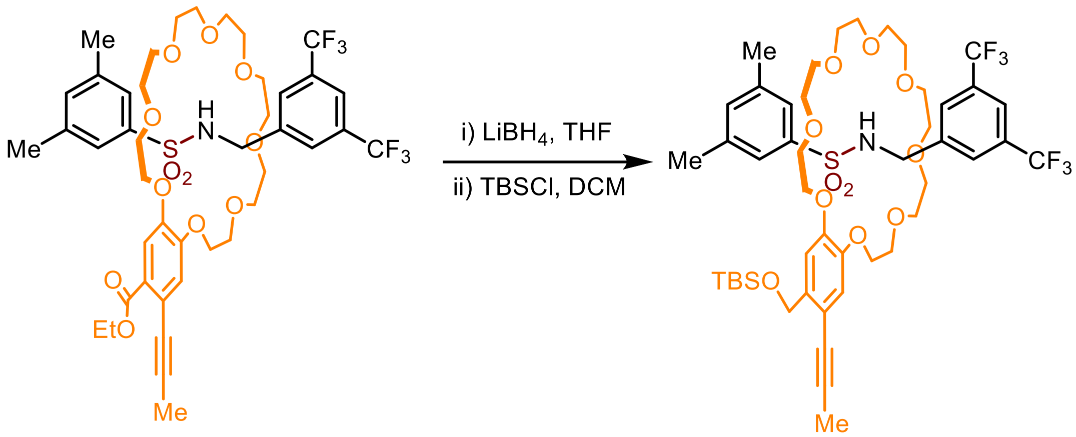

27 same as the sample for X-ray **28**

Procedure: under N_2 atmosphere, to a solution of **27** (1.2 mg, 0.0013 mmol) in 0.2 mL dry THF was added two drops of 2M lithium borohydride in THF. The reaction stirred at room temperature for overnight. The solvent was removed under reduced pressure and dissolved into 1 mL DCM. imidazole (1 mg, 10 equiv.) and TBSCl (2.5 mg, 10 equiv.) was added into the reaction mixture. The solution was stirred at room temperature for 4 hours. The reaction mixture was diluted with 10 mL ethyl acetate and washed with saturated NaCl. The combined organic layers were dried with Na_2SO_4 and concentrated *in vacuo* to afford the crude product. Purification of crude product on PTLC (eluent: ethyl acetate/hexane = 1:3) to give **28** (0.9 mg, 70% yield for two steps) as colorless oil.

HPLC analysis of racemic sample of **17** and compound **28**: Daicel Chiralpak IF, hexane/*iso*-propanol = 95: 5, 1.0 mL/min, temperature = 40 ^{o}C, λ = 254 nm, retention time for racemic sample: 20.7 min and 22.3 min. retention time for chiral **28**: 20.2 min (minor) and 21.4 min (major).

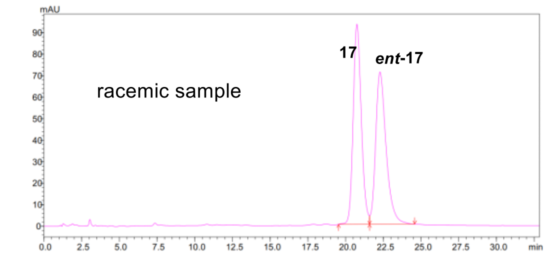

<Peak Table>

PDA Ch1 254nm

Peak#	Ret. Time	Area	Height	Mark	Peak Start	Peak End	Area%
1	20.752	3488993	93038	M	19.531	21.589	50.440
2	22.282	3428160	70830	V M	21.589	24.587	49.560
Total		6917154	163868				100.000

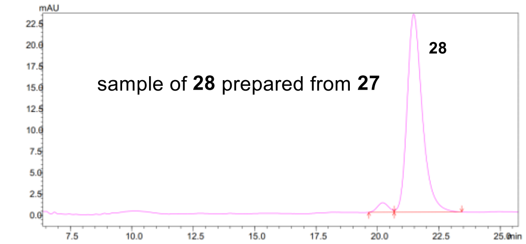

<Peak Table>

PDA Ch1 254nm

Peak#	Ret. Time	Area	Mark	Peak Start	Peak End	Area%
1	20.215	35316	M	19.648	20.683	3.424
2	21.477	996252	V M	20.683	23.435	96.576
Total		1031569				100.000

Therefore, sample **28** should be the same as the minor enantiomer of **17** (i.e., *ent*-**17**) prepared using the method described in the main text. This way, the stereochemistry of the major enantiomer of **17** was determined as opposite to that of sample **28**. The result was consistent with the predicted stereochemical outcome based on the proposed mechanism of chirality transmission from the enantioenriched cavitand template.
